# Efficient Difluoromethylation of sp^3^ Carbon Nucleophiles by Bromodifluoromethylation Reagents with Organic Bases

**DOI:** 10.1002/open.201200033

**Published:** 2012-10-11

**Authors:** Guokai Liu, Xin Wang, Xu Lu, Xiu-Hua Xu, Etsuko Tokunaga, Norio Shibata

**Affiliations:** a Department of Frontier Materials, Graduate School of Engineering, Nagoya Institute of TechnologyGokiso, Showa-ku, Nagoya 466-8555 (Japan) E-mail: nozshiba@nitech.ac.jp

**Keywords:** difluorocabenes, difluoromethylation, electrophilicity, fluorine, regioselectivity

Introduction of a difluoromethyl (CF_2_H) unit into organic molecules is of specific interest in medicinal chemistry and chemical biology, as compounds with a CF_2_H moiety can act as an isostere to molecules having a methanol (CH_2_OH) unit with improved lipophilicity.[1–6] Among several strategies for the synthesis of CF_2_H compounds available, a late-stage difluoromethylation using easy-to-handle reagents under mild conditions is principally advantageous for the synthesis of complex molecules.[7, 9f] Transferring a CF_2_H group from a reagent to a target molecule is key for the reaction, and the reagents are classified according to their nucleophilic, radical, or electrophilic character.[8–10] Electrophilic difluoromethylation through difluorocarbene species has attracted considerable attention, and several methods have been reported.8a–h, j, l Although the difluoromethylation of heteroatom-centered nucleophiles such as oxygen, sulfur and nitrogen nucleophiles is well studied,[11], 8g, j, l mild and efficient difluoromethylation methods for carbon-centered nucleophiles are relatively scarce.8i, k–m, 12d In 2007, Prakash and co-workers developed a new electrophilic difluoromethylating reagent, *S*-(difluoromethyl)diarylsulfonium tetrafluoroborate.8i This reagent is effective for the introduction of a CF_2_H group into a wide range of heteroatom-centered nucleophiles, but it failed to transfer to carbon nucleophiles. Besides, they point out the drawback of *S*-(difluoromethyl)diarylsulfonium tetrafluoroborate being its slow decomposition over time even at low temperatures.8m Lately, Prakash group designed a novel electrophilic difluoromethylating protocol employing in situ prepared *N*,*N*-dimethyl-*S*-difluoromethyl-*S*-phenylsulfoximinium salt as a robust electrophilic difluoromethylating reagent.8m The reagent has exhibited good reactivity toward a broad scope of nucleophiles (N, P, S, and O nucleophiles), but no example was shown for carbon nucleophiles. *N*-Tosyl-*S*-difluoromethyl-*S*-phenylsulfoximine, which was developed as a difluorocarbene precursor by Hu and co-workers in 2009, is effective for transferring a CF_2_H group to both heteroatom and carbon nucleophiles.8l However, for carbon nucleophiles, only a limited number of phenylacetylene derivatives was examined as substrates for difluoromethylation. As part of our research program towards the enantioselective synthesis of biologically attractive fluoro-organic compounds,[12, 13] we required electrophilic difluoromethylation reagents reactive enough for sp^3^ carbon nucleophiles, which provide the CF_2_H compounds with an asymmetric carbon center. In 2010, Xiao et al. for the first time reported symmetrical *S*-(bromodifluoromethyl)diphenylsulfonium salts.8q Recently, we reported the efficient synthesis of a series of unsymmetrical *S*-(bromodifluoromethyl)diarylsulfonium salts **1** which are effective for electrophilic bromodifluoromethylation (^+^CF_2_Br) of terminal alkynes in response to *n*BuLi.8p We disclose herein that the same regents **1** can be used as electrophilic difluoromethylation reagents for sp^3^ carbon nucleophiles mediated by organic bases (Scheme 1). Allylic difluoromethylation of dicyanoalkylidenes proceeds nicely by **1** in the presence of a P_1_ base to give CF_2_H products with a quaternary carbon center in high to excellent yields. A wide range of β-ketoesters are also efficiently reacted with **1**, mediated by 1,8-diazabicyclo[5.4.0]undec-7-ene (DBU )to provide carbon—CF_2_H compounds as major products with a small amount of oxygen—CF_2_H products in high to excellent yields. In addition to the high yields of the carbon—CF_2_H products, the use of reagents **1** having CF_2_Br moieties as a CF_2_H source is of further advantage, as the CF_2_Br-reagents **1** are quite stable due to the lack of an acidic hydrogen atom.8i, l, m

**Scheme 1:**
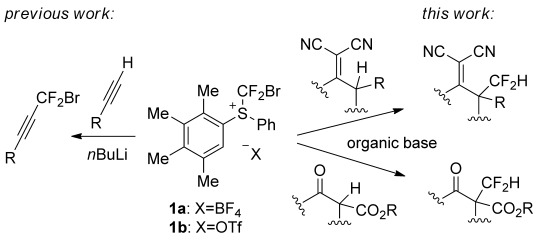
Transferring CF_2_Br or CF_2_H into carbon centers by 1.

Initially, we attempted an electrophilic bromodifluoromethylation of dicyanoalkylidene **2 a** with **1 a** under similar reaction conditions as previously described for trifluoromethylation.13a, c Namely, **2 a** was treated with **1 a** in the presence of P_1_ in acetonitrile at −43 °C for 1 h. Interestingly, CF_2_H compound **3 a** was predominantly obtained in 45 %, instead of predictable CF_2_Br compound **4 a** (Table 1, Entry 1). This result spurred us to use **1 a** as an electrophilic CF_2_H-transfering reagent. We next investigated the effects of base for difluoromethylation of **2 a**. Base P_2_ gave a similar yield of 43 % (Entry 2), but the yield decreased to 11 % using DBU (Entry 3). No reaction took place with an inorganic base (i.e., K_2_CO_3_; Entry 4). Increasing the amount of **1 a** to 1.5 equivalents did not improve the yield of **3 a** (46 %; Entry 5). Because **2 a** was completely consumed in most cases according to TLC analysis, we next carefully examined the ratio of **2 a**, **1 a** and P_1_. A good yield of 67 % was observed at a ratio of 2.0:1.0:2.0 of **2 a**/**1 a**/P_1_ (the yield based on **1 a**; Entry 6), and the yield of **3 a** was further improved to 77 % using triflate **1 b** (Entry 7). When the reaction temperature was lowered to −75 °C in dichloromethane, **3 a** was obtained in 81 % (Entry 8). At a ratio of 2.0/1.0/1.2 of **2 a**/**1 b**/P_1_, 66 % of **3 a** was obtained (Entry 9). It should be mentioned that no bromination product was isolated in all cases in contrast to the results for the difluoromethylation of β-ketoesters **5** (see below).

**Table 1:** Difluoromethylation of dicyanoalkylidene 2 a[a]

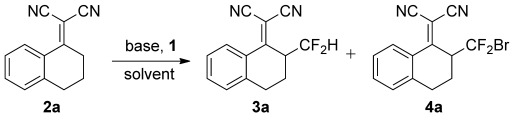						

Entry	1	Base[b]	2 a/1/Base [equiv]	Solvent	T [°C]	Yield 3 a/4 a[%]
1	**1 a**	P_1_	1.0/1.1/2.0	CH_3_CN	−42	45/<5
2	**1 a**	P_2_	1.0/1.1/2.0	CH_3_CN	−42	44/<5
3	**1 a**	DBU	1.0/1.1/2.0	CH_3_CN	−42	11/0
4	**1 a**	K_2_CO_3_	1.0/1.1/2.0	CH_3_CN	−42	NR
5	**1 a**	P_1_	1.0/1.5/2.0	CH_3_CN	−42	46/7
6	**1 a**	P_1_	2.0/1.0/2.0	CH_3_CN	−42	67/10
7	**1 b**	P_1_	2.0/1.0/2.0	CH_3_CN	−42	77/9
8	**1 b**	P_1_	2.0/1.0/2.0	CH_2_Cl_2_	−75	81/0
9	**1 b**	P_1_	2.0/1.0/1.2	CH_2_Cl_2_	−75	66/0

[a] The yields determined by ^19^F NMR are based on substrate (Entries 1–5) or reagent (Entries 6–9).

[b] P_1_: *tert*-butyliminotri(pyrrolidino)phosphorane, P_2_: tetramethyl(tris(dimethylamino)phosphoranylidene)phosphorictria-mid-Et-imin, DBU: 1,8-diazabicyclo[5.4.0]undec-7-ene.

With the optimized reaction conditions in hand (**2**/**1 b**/P_1_=2.0:1.0:2.0), we screened a variety of substrates **2** (Scheme 2). All the benzodicyanoalkylidenes afforded the corresponding allylic CF_2_H compounds **3 b**–**e** in good to excellent yields, and the hetero-cyclodicyanoalkylidenes **2 f** and **2 g** were also transferred efficiently into **3 f** and **3 g** in 76 % and 92 %, respectively, and nonaromatic substrate **2 i** gave allylic CF_2_H compound **3 i** in 55 %. We next investigated the reaction of acyclic dicyanoalkylidenes **2 j**–**r** with **1 b**. The desired CF_2_H compounds **3 j**–**r** were obtained in moderate to excellent yields.

**Scheme 2:**
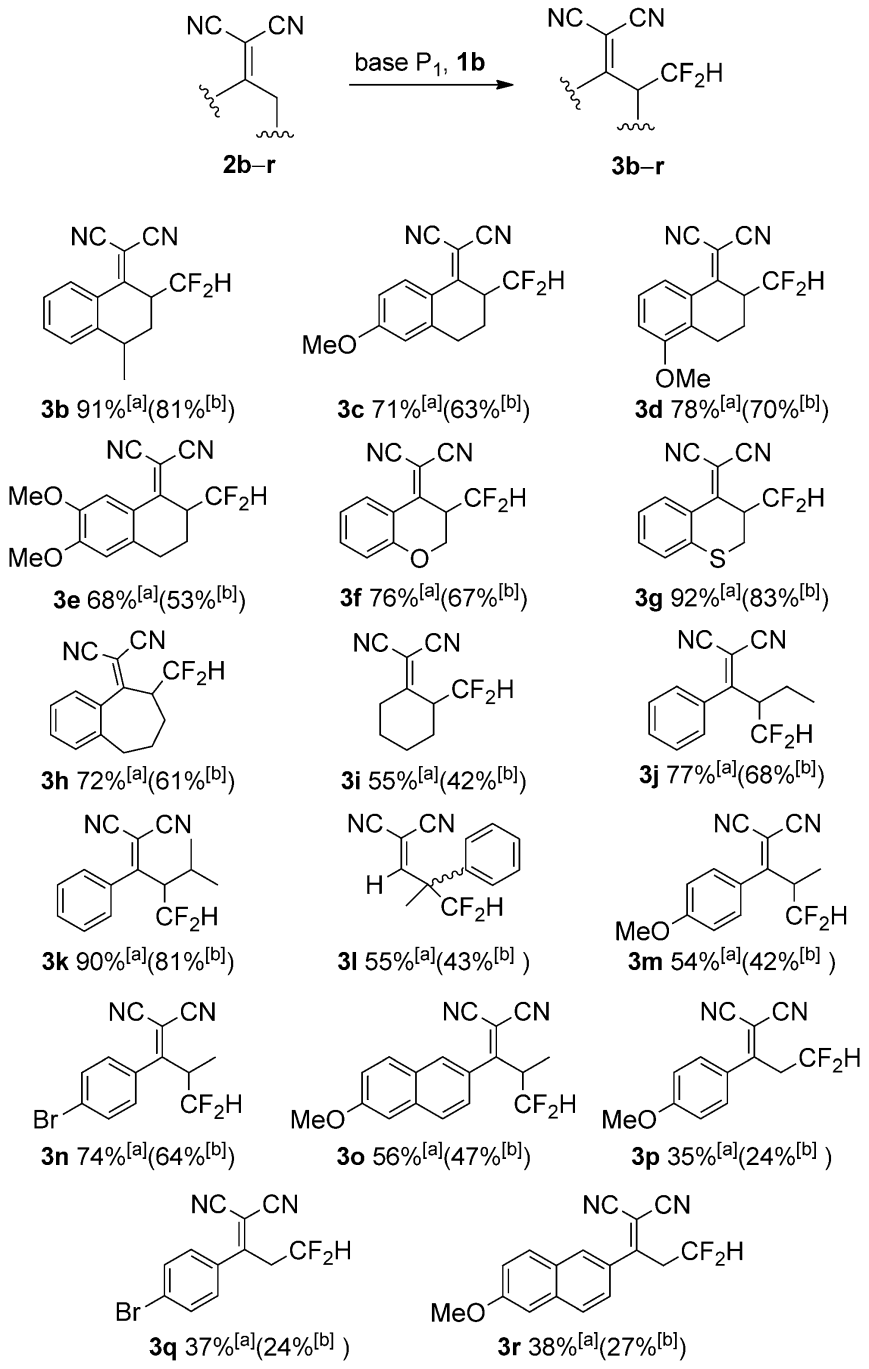
Difluoromethylation of dicyanoalkylidene 2 b–r. *Reagents and conditions*: 2 b–r (2.0 equiv), P_1_ (2.0 equiv), 1 b (1.0 equiv), CH_2_Cl_2_, −75 °C. [a] Determined by ^19^F NMR using trifluorotoluene as the internal standard. [b] Isolated yields.

Difluoromethylation of β-ketoester **5 a** with **1** was next evaluated (Table 2). Based on the results for difluoromethylation of **2** with **1**, we first examined the reaction under the conditions of **5 a**/**1 a**/P1 at a ratio of 2.0:1.0:1.0 in CH_2_Cl_2_ at −75 °C. A mixture of C—CF_2_H product **6 a** and O—CF_2_H product **7 a** was obtained in 51 % (**6 a**/**7 a**=65:35; Entry 1). Optimization of base was next performed (Entries 2–4) and DBU gave a good result of 70 % (**6 a**/**7 a**=69:31; Entry 3). Because approximately 20 % of **1 a** was not consumed according to ^19^F NMR analysis, the conditions were changed to **5 a**/**1 a**/DBU at a ratio of 2.2:1.0:1.3, affording **6 a** and **7 a** in 81 % yield (**6 a**/**7 a**=65:35; Entry 5). The result was further improved using **1 b** instead of **1 a** under the same conditions, as a combined yield of 85 % was achieved with higher C/O regioselectivity (**6 a**/**7 a**=80:20; Entry 6). We also noticed that 82 % of α-brominated product **8 a** was produced (Entry 6). It is should be mentioned that difluoromethylation of **5 a** with *N*-tosyl-*S*-difluoromethyl-*S*-phenylsulfoximine developed by Hu gave a mixture of **6 a** and **7 a** at low yields of 38 % with lower C/O regioselectivity (**6 a**/**7 a**=63:37; data not shown).12d The CF_2_H analogue of **1 a**, Prackash reagent, also gave a mixture of **6 a** and **7 a** in good 80 % yield but with lower C/O regioselectivity (**6 a**/**7 a**=66:34; Entry 7).8i

**Table 2:** Difluoromethylation of β-ketoester 5 a[a]

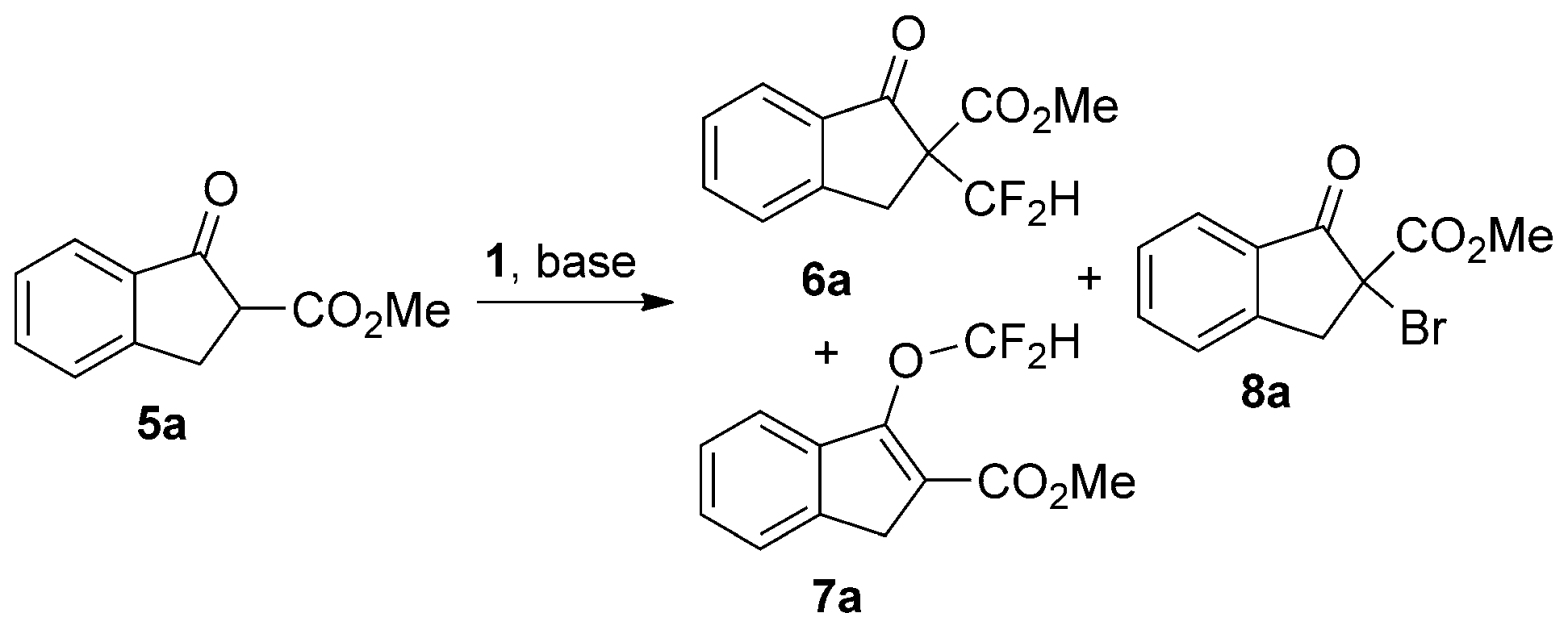						

Entry	1	Base[b]	5 a/Base [equiv]	Yield 6 a and 7 a[%][c]	Ratio 6 a/7 a	Yield 8 a[%][d]
1	**1 a**	P_1_	2.0/1.0	51	65/35	–
2	**1 a**	P_2_	2.0/1.0	47	72/28	–
3	**1 a**	DBU	2.0/1.0	70	69/31	–
4	**1 a**	Cs_2_CO_3_	2.0/1.0	NR	–	NR
5	**1 a**	DBU	2.2/1.3	81	65/35	–
6	**1 b**	DBU	2.2/1.3	85	80/20	82
7	**1 c**[e]	DBU	2.2/1.3	80	66/34	–

[a] *Reagents and conditions*: **5 a**, **1 a**–c (1.0 equiv), base, CH_2_Cl_2_, −75 °C.

[b] P_1_: *tert*-butyliminotri(pyrrolidino)phosphorane, P_2_: tetramethyl-(tris(dimethylamino)phosphoranylidene)phosphorictria-mid-Et-imin, DBU: 1,8-diazabicyclo[5.4.0]undec-7-ene.

[c] Yields were determined using ^19^F NMR based on **1**.

[d] Isolated yield.

[e] **1 c**: Prakash reagent8i (CF_2_H analogue of **1 a**) was used under the same reaction conditions for comparison.

To explore the scope of difluoromethylating β-ketoester **5** with **1 b** under optimum reaction conditions, we carried out experiments with a variety of substrates **5**, including indanone carboxylates, tetralone carboxylates, and other β-ketoesters (Table 3). Methyl indanone carboxylates **5 b**–**f** with a variety of substituents in the aromatic ring proceeded well at providing their corresponding CF_2_H compounds **6 b**–**f** and **7 b**–**f** in good to excellent yields with high C/O regioselectivity (70:30 to 84:16; Entries 1–5). Substrates with an electron-withdrawing group in the aromatic ring gave higher yields than those without substituents or those with an electron-donating group in the aromatic ring (Entries 4 and 5). Ethyl, benzyl, *tert*-butyl, *iso*-propyl and allyl indanone carboxylates **5 g**–**l** also proceed smoothly in good to excellent yields of CF_2_H products **6 g**–**l** and **7 g**–**l** (Entries 6–11). Although tetralone carboxylates **5 m** and **5 n** were less reactive with **1 b** to provide corresponding CF_2_H products **6 m**/**7 m** and **6 n**/**7 n** in moderate yields (49 % and 47 %, respectively; Entries 12 and 13), high C/O regioselectivities were achieved (88:12 and 87:13, respectively; Entries 12 and 13). Benzyl 2-oxocyclopentanecarboxylate **5 o** gave **6 o** and **7 o** in 63 % at a 79:21 ratio (Entry 14). Acyclic β-ketoester **5 p** also proceeded well to provide corresponding CF_2_H compounds **6 p** and **7 p** (Entry 15). It was noteworthy that brominated compounds **8** were isolated with excellent yields in all cases except for **8 o** and **8 p**.

**Table 3:** Difluoromethylation of β-ketoester 5 b–p[a]

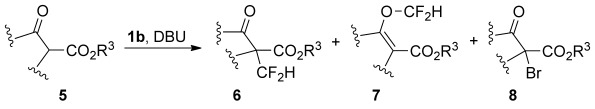									

Entry		β-Ketoester 5					Yield 6 and 7[%][b]	Ratio 6/7[c]	Yield 8[%][d]
			R^1^	R^2^	R^3^	*n*			
1	**5 b**	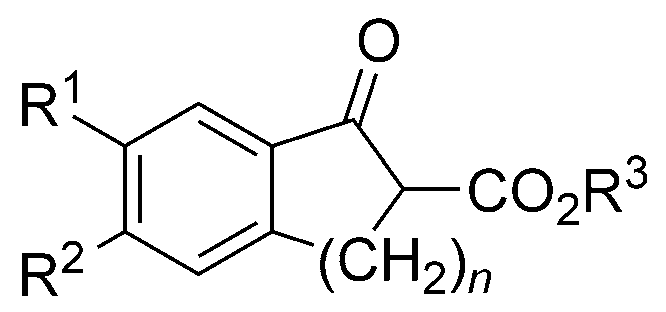	Me	H	Me	1	88 (75)	75:25	95
2	**5 c**		OMe	H	Me	1	89 (78)	75:25	90
3	**5 d**		OMe	OMe	Me	1	89 (80)	84:16	91
4[e]	**5 e**		H	Br	Me	1	quant. (91)	71:29	92
5[e]	**5 f**		H	Cl	Me	1	quant. (88)	70:30	92
6	**5 g**		H	H	Bn	1	84 (76)	72:28	93
7	**5 h**		H	H	Et	1	84 (76)	75:25	92
8	**5 i**		H	H	*t*Bu	1	80 (74)	77:23	96
9	**5 j**		H	H	Allyl	1	82 (75)	73:27	95
10[e]	**5 k**		H	H	*i*Pr	1	74 (60)	77:23	84
11[e]	**5 l**		H	Br	Bn	1	89 (73)	71:29	90
12[f]	**5 m**		H	H	Me	2	49 (32)	88:12	74
13[e,f]	**5 n**		H	H	Bn	2	47 (27)	87:13	72
14[g]	**5 o**	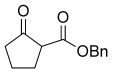	–	–	–	–	63 (47)	79:21	–
15[g,h]	**5 p**	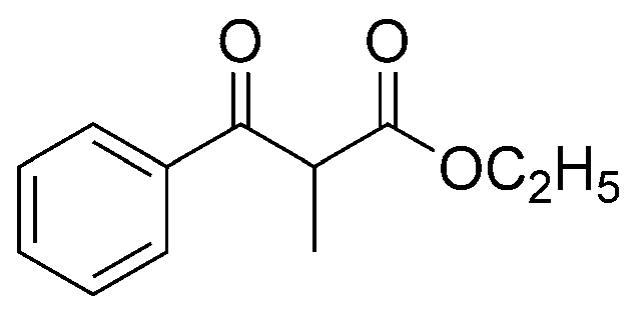	–	–	–	–	56 (41)	74:26	-

[a] *Reagents and conditions*: **5 b**–**p** (2.0–2.2 equiv), **1 b** (1.0 equiv), 1,8-diazabicyclo[5.4.0]undec-7-ene (DBU; 1.0–1.3 equiv), CH_2_Cl_2_, −75 °C. For detailed reaction conditions, see the Supporting Information.

[b] Yields were determined by ^19^F NMR using trifluorotoluene as the internal standard, and the data in parentheses are isolated yields.

[c] Determined using ^19^F NMR.

[d] Isolated yields.

[e] Substrate/DBU/CF_2_Br=2.0:1.0:1.0.

[f] **7** were not separated because of their low yields.

[g] **8** could not be obtained as a pure product.

[h] P_1_ was used instead of DBU.

Based on the experimental results, we propose a plausible mechanism for difluoromethylation with **1** taking into account information from the literature8l, m (Scheme 3). Initially, substrate Sub—H (**2**, **5**) was treated with a base (P_1_ or DBU) to provide Sub^−^[Base—H]^**+**^. Generated Sub^−^ attracts the bromine atom in **1** to give brominated product Sub—Br (**8**) and difluorocarbene (:CF_2_) with PhSC_6_HMe_4_ and X^−^[Base—H]^+^. Difluorocarbene (:CF_2_) reacts with additional Sub to give difluoromethylated products **3, 6** and **7** with base. In the case of difluoromethylation of dicyanoalkylidenes **2**, no bromination product such as **8** was detected and two equivalents of base were required. These facts can be explained, as bromination products of **2** (Sub—Br) are further reacted with base to give salts Sub^−^[Base—Br]^**+**^.

**Scheme 3:**
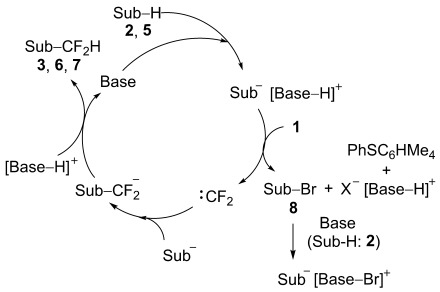
Proposed reaction mechanism.

In conclusion, we have developed a new protocol for electrophilic difluoromethylation of sp^3^ carbon nucleophiles by shelf-stable CF_2_Br reagent **1** through the in situ generation of difluorocarbene induced by an organic base. A wide range of dicyanoalkylidenes **2** and β-ketoesters **5** proceed efficiently to give corresponding C**—**CF_2_H products **3** and **6** in good to excellent yields under mild conditions. Enantioselective difluoromethylation of sp^3^ carbon nucleophiles by **1** is under investigation.

## Experimental Section

**General procedure for electrophilic difluoromethylation of dicyanoalkylidenes**: To a stirred solution of dicyanoalkylidenes (0.20 mmol) in dry CH_2_Cl_2_ (2 mL), a corresponding base (0.20 mmol) was added at −20 °C under an inert atmosphere. After stirring for 20 min at −20 °C, the resulting reaction mixture was further cooled to −75 °C, and reagent **1** (0.10 mmol) was added to the reaction mixture in one portion at the same temperature. The resulting mixture was maintained for 1 h at −75 °C, then warmed to RT naturally. The reaction mixture was concentrated in vacuo, and the residue was subject to column chromatography on silica gel to afford the pure products.

**General procedure for electrophilic difluoromethylation of β-ketoesters**: To a stirred solution of β-ketoesters (0.22 mmol) in dry CH_2_Cl_2_ (2 mL), a corresponding base (0.13 mmol) was added at −75 °C under an inert atmosphere. After stirring for 30 min at −75 °C, reagent **1** (0.10 mmol) was added to the reaction mixture in one portion at the same temperature. The resulting mixture was maintained for 1 h at −75 °C, then warmed to RT naturally. The reaction mixture was concentrated in vacuo and the residue was subject to column chromatography on silica gel to afford the pure products.

## Supplementary material

Detailed facts of importance to specialist readers are published as ”Supporting Information”. Such documents are peer-reviewed, but not copy-edited or typeset. They are made available as submitted by the authors.
