# Supplementary material for: Efficient Difluoromethylation of sp3 Carbon Nucleophiles by Bromodifluoromethylation Reagents with Organic Bases
Source: ChemistryOpen. 2012 Oct 11;1(5):227–31. doi: 10.1002/open.201200033 (PMC3922594; doi:10.1002/open.201200033)

ChemistryOPEN

Supporting Information

© 2012 The Authors. Published by Wiley-VCH Verlag GmbH & Co. KGaA, Weinheim

Efficient Difluoromethylation of sp^3 Carbon Nucleophiles by Bromodifluoromethylation Reagents with Organic Bases

Guokai Liu, Xin Wang, Xu Lu, Xiu-Hua Xu, Etsuko Tokunaga, and Norio Shibata*[a]

open_201200033_sm_miscellaneous_information.pdf

Experimental

General experimental details

All reactions were performed in oven-dried glassware under a positive pressure of nitrogen. Solvents were transferred via syringe and were introduced into the reaction vessels through a rubber septum. All of the reactions were monitored by thin-layer chromatography (TLC) carried out on 0.25 mm Merck silica gel (60-F254). The TLC plates were visualized with UV light and 7% phosphomolybdic acid or $KMnO_4$ in water/heat. Column chromatography was carried out on a column packed with silica gel 60N spherical neutral size 63-210 μm. The ^{1}H NMR (300 MHz) and ^{19}F NMR (282 MHz) spectra for solution in $CDCl_3$, were recorded on a Varian Mercury 300. ^{13}C NMR (150.9 MHz) spectra were recorded on a BRUKER 600 UltraShield^{TR}. Chemical shifts (δ) are expressed in ppm downfield from internal TMS or $CFCl_3$. Mass spectra were recorded on a SHIMADZU GCMS-QP5050A (EI-MS) and SHIMAZU LCMS-2010EV (ESI-MS and APCI-MS). Infrared spectra were recorded on a JASCO FT/IR-200 or a JASCO FT/IR-4100 spectrometer.

General procedure for the preparation of dicyanoalkylidenes[1]: A solution of ketone (2 mmol), malononitrile (158mg, 2.4 mmol), ammonium acetate (3 mg, 0.4mmol) and acetic acid (0.125 mL, 2.2 mmol) in toluene (20 mL) were refluxed overnight. The reaction mixture was diluted with toluene, and then washed with water and brine. After drying over Na_2SO_4, the solvent was removed under reduced pressure, and the residue was subject to chromatography on silica gel using hexane and ethyl acetate as an eluent to afford corresponding dicyanoalkylidenes **2**.

All the *β*-ketoesters Substrates (5a–p) were prepared according to the literature procedure.[2]

2-(1-(4-methoxyphenyl)propylidene)malononitrile (2m)

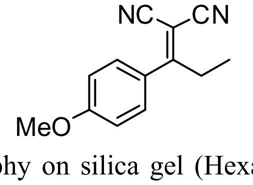

Purified through column chromatography on silica gel (Hexane/EtOAc = 10/1) to give **2m,** white solid, yield: 0.36 g, 90 %**.** ^{1}H NMR (300 MHz, $CDCl_3$) δ 7.55 (d, J = 2.1 Hz, 1H), 7.53 (d, J = 2.1 Hz, 1H), 7.01(d, J = 2.1 Hz, 1H), 6.99 (d, J = 2.1 Hz, 1H), 3.87 (s, 3H), 2.96 (q, J = 7.8 Hz, 2H), 1.12 (t, J = 7.8 Hz, 3H); ^{13}C NMR (150.9MHz, $CDCl_3$) δ 180.6, 163.0, 130.0, 126.6, 114.7, 113.8, 113.2, 81.7, 55.7, 30.7, 13.4; IR (KBr) 3028, 2966,2938, 2839, 2228, 1609, 1577, 1556, 1514, 1461, 1315, 1271, 1242, 1029, 833, 740, 620 cm^{-1} ; m.p.: 55-56 ℃; MS (APCI, *m/z*) 211.0 [M-H]$^{-}$; HRMS (APCI) Calcd. for $C_{13}H_{13}N_2O$ [M+H]$^{+}$: 213.1028, Found: 213.1028.

2-(1-(4-bromophenyl)propylidene)malononitrile (2n)

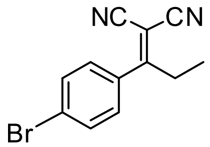

Purified through column chromatography on silica gel (Hexane/EtOAc = 10/1) to give **2n,** light yellow solid, yield: 0.38 g, 76 %. ^{1}H NMR (300 MHz, $CDCl_3$) δ 7.66 (d, J = 8.4 Hz, 2H), 7.36 (d, J = 8.4 Hz, 2H), 2.97 (q, J = 7.8 Hz, 2H), 1.11 (t, J = 7.8 Hz, 3H); ^{13}C NMR (150.9MHz, $CDCl_3$) δ 180.3, 133.5, 132.8, 129.2, 127.0, 112.7, 112.3, 85.0, 31.2, 13.0; IR (KBr) 2983, 2228, 1584, 1485, 1448, 1394, 1071, 1009, 872, 840, 825, 730 cm^{-1} ; m.p.: 113-115 ℃; MS (APCI, *m/z*) 259.0, 261.0 $[M-H]^-$; (APCI) Calcd. for $C_{12}H_{10}N_2Br$ $[M+H]^+$: 261.0027, Found: 261.0025.

2-(1-(6-methoxynaphthalen-2-yl)propylidene)malononitrile (2o)

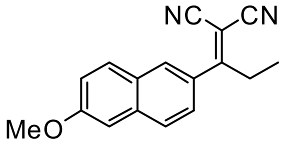

Purified through column chromatography on silica gel (Hexane/EtOAc = 6/1) to give **2o,** light yellow solid, yield: 0.46 g, 92 %. ^{1}H NMR (300 MHz, $CDCl_3$) δ 7.98 (s, 1H), 7.82 (dd, J = 8.4, 2.1 Hz, 2H), 7.51 (dd, J = 8.4, 1.2 Hz, 1H), 7.23 (dd, J = 9.0, 2.4 Hz, 1H), 3.95 (s, 3H), 3.07 (q, J = 7.8 Hz, 2H), 1.15 (t, J = 7.8 Hz, 3H); ^{13}C NMR (150.9MHz, $CDCl_3$) δ 181.4, 160.0, 136.6, 130.8, 129.6, 128.7, 128.1, 128.0, 124.5, 120.6, 113.5, 113.1, 105.9, 83.2, 55.7, 31.0, 13.4; IR (KBr) 2983, 2935, 2229, 1623, 1568, 1482, 1392, 1313, 1267, 1213, 1177, 1020, 901, 853, 824, 726 cm^{-1}; m.p.: 107-109 ℃; MS (APCI, *m/z*) 261.1 $[M-H]^-$; HRMS (APCI) Calcd. for $C_{17}H_{15}N_2O$ $[M+H]^+$: 263.1184, Found: 263.1185.

General procedure for electrophilic difluoromethylation of dicyanoalkylidenes: To a stirred solution of dicyanoalkylidenes (0.20 mmol) in 2 mL dry CH_2Cl_2 was added P_1 (0.20 mmol) at –20 °C under an inert atmosphere. After stirring for 20 min at –20 °C, the resulting reaction mixture was further cooled to –75°C and reagent **1b** (0.10 mmol) was added to reaction mixture in one portion at the same temperature, and the resulting mixture was maintained for 1hr at –75 °C, then warmed to room temperature naturally. The reaction mixture was concentrated under reduced pressure and the residue was subject to column chromatography on silica gel to afford pure desired products (eluent: hexane/ethyl acetate).

2-[3-(Difluoromethyl)-2,3-dihydronaphthalen-4(1*H*)-ylidene]malononitrile

(3a)

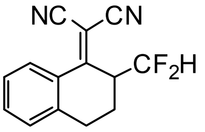

Purified through column chromatography on silica gel (Hexane/EtOAc = 95/5) to give **3a,** white solid, yield: 18.5 mg, 76%**.** ^{1}H NMR (300 MHz, $CDCl_3$) δ 7.54 (t, *J* = 4.5 Hz, 1H), 7.25–7.32 (m, 3H), 6.86(t, *J* = 4.5 Hz, 1H), 6.39 (t, *J* = 54.0 Hz, 1H), 2.80 (t, *J* = 7.5 Hz, 2H), 2.43–2.50 (m, 2H), 3.65 (dd, *J* = 50.1, 17.4 Hz, 2H); ^{19}F NMR (282 MHz, $CDCl_3$) δ –119.1 (d, *J* = 28.2, 2F); ^{13}C NMR (150.9MHz, $CDCl_3$) δ 137.4, 136.7, 129.3, 129.1, 128.6, 127.1, 123.3, 122.6, 110.2 (t, *J* = 3.0 Hz), 110.0 (t, *J* = 256.5 Hz), 47.0 (t, *J* = 25.5 Hz), 27.4, 23.6; IR (KBr) 3064, 2958, 2900, 1489, 1450, 1366, 1291, 1131, 1112, 1089, 1044, 842, 765, 737, 572 cm^{-1} ; m.p.: 102–105 ℃; MS (APCI, *m/z*) 279.0, 281.0 $[M+Cl]^-$; HRMS (APCI) Calcd. for $C_{14}H_{11}N_2F_2$ $[M+H]^+$: 245.0890, Found: 245.0897.

2-[3-(Difluoromethyl)-2,3-dihydro-1-methylnaphthalen-4(1*H*)-ylidene]malononitrile (3b)

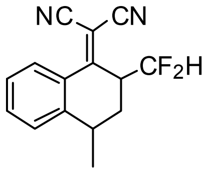

Purified through column chromatography on silica gel (Hexane/EtOAc = 97/3) to give **3b,** white solid, yield: 21.0 mg, 81%**.** ^{1}H NMR (300 MHz, $CDCl_3$) δ 7.58 (d, *J* = 6.9 Hz, 1H), 7.27–7.35 (m, 3H), 6.79 (t, *J* = 5.4 Hz, 1H), 6.42 (t, *J* = 54.0 Hz, 1H), 2.92-3.0 (m, 1H), 2.56–2.66 (m, 1H), 2.27–2.37 (m, 1H); ^{19}F NMR (282 MHz, $CDCl_3$) δ -118.8 (dd, *J* = 273.3, 54.4 Hz, 1F), –119.9 (dd, *J* = 273.3, 54.4 Hz , 1F); ^{13}C NMR (150.9MHz, $CDCl_3$) δ 142.4, 135.2, 129.7, 127.9, 127.7, 127.0, 122.8, 122.6, 110.4 (d, *J* = 4.5 Hz), 110.1, 110.0 (t, *J* = 256.5 Hz), 47.0 (t, *J* = 30.0 Hz), 31.7, 31.2, 19.7; IR (KBr) 3038, 2960, 2927, 1488, 1447, 1371, 1362, 1224, 1115, 1097, 1067, 1016, 883, 812, 766, 752, 725, 573 cm^{-1} ; m.p.: 84–86 ℃; MS (APCI, *m/z*) 291.1 $[M+MeOH+H]^+$; HRMS (APCI) Calcd. for $C_{15}H_{13}N_2F_2$ $[M+H]^+$: 259.1047, Found: 259.1036.

2-[3-(Difluoromethyl)-2,3-dihydro-7-methoxynaphthalen-4(1*H*)-ylidene]malononitrile (3c)

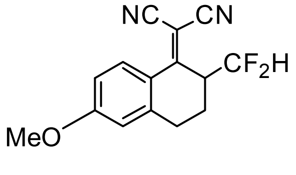

Purified through column chromatography on silica gel (Hexane/EtOAc = 95/5) to give **3c,** white solid, yield: 17.3 mg, 63%. ^{1}H NMR (300 MHz, $CDCl_3$) δ 7.48 (d, *J* = 9.0 Hz, 1H), 6.80 (d, *J* = 6.0 Hz, 2H), 6.70 (t, *J* = 6.0 Hz, 1H), 6.36 (t, *J* = 54.0 Hz, 1H), 3.83 (s, 3H), 2.77 (t, *J* = 7.5 Hz, 2H), 2.40–2.47 (m, 2H); ^{19}F NMR (282 MHz, $CDCl_3$) δ –119.1 (d, *J* = 56.4 Hz, 2F); ^{13}C NMR (150.9MHz, $CDCl_3$) δ 160.0, 139.6, 133.7, 124.1, 123.0, 121.6, 115.4, 111.4, 110.3, 110.1 (t, *J* = 256.5 Hz), 55.6, 47.0 (t, *J* = 30.0 Hz), 28.0, 23.5; IR (KBr) 3028, 1962, 2841, 1611, 1570, 1494, 1431, 1146, 1121, 1103, 1089, 1046, 930, 874, 833, 805, 754, 672, 574 cm^{-1} ; m.p.: 46–48 ℃; MS (APCI, *m/z*) 309.0, 311.0 $[M+Cl]^-$; HRMS (APCI) Calcd. for $C_{15}H_{13}N_2OF_2$ $[M+H]^+$: 275.0996, Found: 275.1004.

2-[3-(Difluoromethyl)-2,3-dihydro-8-methoxynaphthalen-4(1*H*)-ylidene]malononitrile (3d)

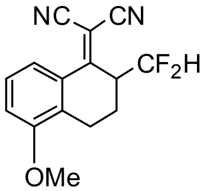

Purified through column chromatography on silica gel (Hexane/EtOAc = 97/3) to give **3d,** white solid, yield: 19.2 mg, 70%. ^{1}H NMR (300 MHz, $CDCl_3$) δ 7.26 (t, *J* = 8.1, 1H), 7.18 (d, *J* = 7.8 Hz, 1H), 6.92 (d, *J* = 6.0 Hz, 1H), 6.87 (t, *J* = 6.0 Hz, 1H), 6.40 (t, *J* = 54.0 Hz, 1H), 3.86 (s, 3H), 2.79 (t, *J* = 9.0 Hz, 2H), 2.38–2.45 (m, 2H); ^{19}F NMR (282 MHz, $CDCl_3$) δ –119.3 (d, *J* = 56.4 Hz, 2F); ^{13}C NMR (150.9MHz, $CDCl_3$) δ 156.9, 136.9, 129.6, 127.3, 125.7, 123.2, 115.2, 111.7, 110.3, 110.0 (t, *J* = 256.5 Hz), 55.8, 47.2 (t, *J* = 30.0 Hz), 23.1, 19.1; IR (KBr) 2946, 2891, 2844, 1576, 1477, 1457, 1440, 1356, 1270, 1141, 1119, 1031, 835, 784, 744, 730 cm^{-1} ; m.p.: 63–65 ℃; MS (APCI, *m/z*) 275.0 $[M+H]^+$, 307.1 $[M+MeOH+H]^+$; HRMS (APCI) Calcd. for $C_{15}H_{13}N_2OF_2$ $[M+H]^+$: 275.0996, Found: 275.0997.

2-[3-(Difluoromethyl)-2,3-dihydro-6,7-dimethoxynaphthalen-4(1*H*)-ylidene] malononitrile (3e)

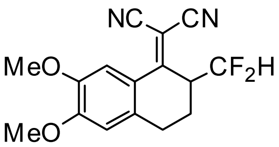

Purified through column chromatography on silica gel (Hexane/EtOAc = 6/1) to give **3e,** yellow solid, yield: 16.0 mg, 53%. ^{1}H NMR (300 MHz, $CDCl_3$) δ 7.12(s, 1H), 6.77 (s, 1H), 6.71 (d, *J* = 6.0 Hz, 1H), 6.33 (t, *J* = 54.0 Hz, 1H), 3.90 (s, 3H), 3.90 (s, 3H), 2.72 (t, *J* = 9.0 Hz, 2H), 2.39–2.46 (m, 2H); ^{19}F NMR (282 MHz, $CDCl_3$) δ –118.6 (d, *J* = 56.4 Hz, 2F); ^{13}C NMR (150.9MHz, $CDCl_3$) δ

149.4, 147.5, 134.0, 130.8, 123.0, 121.1, 112.2, 110.4 (t, J = 256.5 Hz), 110.4 (t, J = 4.5 Hz), 56.5, 56.2, 46.9 (t, J = 30.0 Hz), 27.2, 23.7; IR (KBr) cm^{-1} 3026, 2962, 2835, 1574, 1515, 1465, 1371, 1332, 1271, 1221, 1151, 1115, 1083, 1035, 857, 782, 749, 726 cm^{-1}; m.p.: 125–127 ℃; MS (APCI, *m/z*) 337.1 $[M+MeOH+H]^+$; HRMS (APCI) Calcd. for $C_{16}H_{15}N_2O_2F_2$ $[M+H]^+$: 305.1102, Found: 305.1106.

2-(3-(difluoromethyl)chroman-4-ylidene)malononitrile (3f)

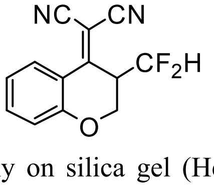

Purified through column chromatography on silica gel (Hexane/EtOAc = 90/10) to give **3f,** light yellow solid, yield: 16.5 mg, 67%**.** ^{1}H NMR (300 MHz, $CDCl_3$) δ 7.50 (d, J = 7.8 Hz, 1H), 7.30 (t, J = 9.0 Hz, 1H), 6.96-7.06 (m, 2H), 6.52 (d, J = 2.4 Hz, 1H), 6.34 (t, J = 54.0 Hz, 1H), 4.86 (d, J = 3.3, 2H); ^{19}F NMR (282 MHz, $CDCl_3$) δ −118.6 (d, J = 54.4 Hz, 2F); ^{13}C NMR (150.9MHz, $CDCl_3$) δ 154.8, 131.8, 128.3, 123.0, 122.3, 121.2, 118.0, 117.6, 109.8 (t, J = 271.6 Hz), 109.4 (t, J = 3.0 Hz), 64.7, 46.0 (t, J = 25.6 Hz); IR (KBr) 3040, 2856, 1604, 1491, 1459, 1448, 1354, 1235, 1121, 1107, 1092, 1060, 942, 765, 625, 573 cm^{-1} ; m.p.: 101–103 ℃; MS (APCI, *m/z*) 281.0, 283.0 $[M+Cl]^-$; HRMS (APCI) Calcd. for $C_{13}H_9N_2OF_2$ $[M+H]^+$: 247.0683, Found: 247.0677.

2-(3-(difluoromethyl)thiochroman-4-ylidene)malononitrile (3g)

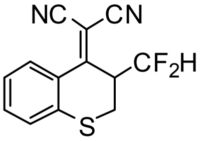

Purified through column chromatography on silica gel (Hexane/EtOAc = 94/6) to give **3g,** yellow solid, yield: 22.0 mg, 83%**.** ^{1}H NMR (300 MHz, $CDCl_3$) δ 7.69–7.72 (m, 1H), 7.47–7.49 (m, 1H), 7.24–7.31 (m, 2H), 6.87 (t, J = 6.0 Hz, 1H), 6.24 (t, J = 54.0 Hz, 1H), 3.38 (d, J = 6.0 Hz, 2H); ^{19}F NMR (282 MHz, $CDCl_3$) δ −118.9 (d, J = 54.4 Hz, 2F); ^{13}C NMR (150.9MHz, $CDCl_3$) δ 135.9, 130.1, 129.7, 129.4, 128.5, 126.4, 125.0, 124.6, 110.2 (t, J = 256.5 Hz), 110.0 (t, J = 3.0 Hz), 47.3 (t, J = 25.6 Hz), 25.0; IR (KBr) 3060, 2206, 1588, 1465, 1433, 1369, 1122, 1111, 1090, 876, 814, 764, 730 cm^{-1} ; m.p.: 99 –101 ℃; MS (APCI, *m/z*) 297.0, 299.0 $[M+Cl]^-$; HRMS (APCI) Calcd. for $C_{13}H_9N_2F_2S$ $[M+H]^+$: 263.0455, Found: 263.0439.

2-(6-(difluoromethyl)-6,7,8,9-tetrahydro-5H-benzo[7]annulen-5-ylidene)malononitrile (3h)

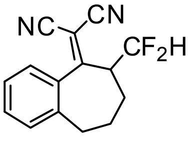

Purified through column chromatography on silica gel (Hexane/EtOAc = 99/1) to give **3h,** white solid, yield: 15.6 mg, 61%**.** ^{1}H NMR (300 MHz, $CDCl_3$) δ 7.66–7.69 (m, 1H), 7.30–7.37 (m, 3H), 7.00 (t, *J* = 7.8 Hz, 1H), 5.87 (t, *J* = 54.0 Hz, 1H), 2.53 (t, *J* = 6.0 Hz, 2H), 2.11-2.18 (m, 2H), 1.95–2.02 (m, 2H); ^{19}F NMR (282 MHz, $CDCl_3$) δ –120.8 (d, *J* = 56.4 Hz, 2F); ^{13}C NMR (150.9MHz, $CDCl_3$) δ 142.1, 138.8, 133.0, 130.1, 129.8, 127.2, 126.6, 125.3, 111.1 (t, *J* = 256.5 Hz), 110.5, 47.9 (t, *J* = 30.0 Hz), 33.8, 31.6, 24.9; IR (KBr) 3059, 2978, 2948, 2870, 1451, 1358, 1259, 1132, 1104, 1062, 1042, 887, 770, 741, 524 cm^{-1} ; m.p.: 65–68 ℃; MS (APCI, *m/z*) 293.0, 295.0 $[M+Cl]^-$; HRMS (APCI) Calcd. for $C_{15}H_{13}N_2F_2$ $[M+H]^+$: 259.1047, Found: 259.1048.

2-(2-(difluoromethyl)cyclohexylidene)malononitrile (3i)

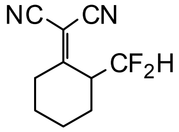

Purified through column chromatography on silica gel (Hexane/EtOAc = 95/5) to give **3i,** white solid, yield: 8.2 mg, 42%**.** ^{1}H NMR (300 MHz, $CDCl_3$) δ 6.40–6.43 (m, 1H), 5.94 (t, *J* = 54.0 Hz, 1H), 2.22–2.26 (m, 4H), 1.73–1.80 (m, 2H), 1.61–1.69 (m, 2H); ^{19}F NMR (282 MHz, $CDCl_3$) δ –118.8 (d, *J* = 54.0 Hz, 2F); ^{13}C NMR (150.9MHz, $CDCl_3$) δ 134.6, 123.3, 111.4 (t, *J* = 256.5 Hz), 109.9 (t, *J* = 3.0 Hz), 48.9 (t, *J* = 24.0 Hz), 25.9, 25.7, 22.3, 21.1; IR (KBr) 2936, 2287, 1726, 1437, 1368, 1118, 1094, 925, 753, 714 cm^{-1} ; m.p.: 40–42 ℃; MS (APCI, *m/z*) 229.1 $[M+MeOH+H]^+$; HRMS (APCI) Calcd. for $C_{10}H_9N_2F_2$ $[M-H]^-$: 195.0734, Found: 195.0748.

2-(2-(difluoromethyl)-1-phenylbutylidene)malononitrile (3j)

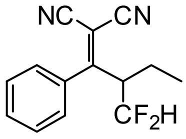

Purified through column chromatography on silica gel (Hexane/EtOAc = 95/5) to give **3j,** white solid, yield: 16.8 mg, 68%**.** ^{1}H NMR (300 MHz, $CDCl_3$) δ 7.45–7.47 (m, 3H), 7.22–7.26 (m, 2H), 6.52 (t, *J* = 6.0 Hz, 1H), 5.76 (t, *J* = 54.0 Hz, 1H), 1.88–1.98 (m, 2H), 0.98 (t, *J* = 6.0 Hz, 3H); ^{19}F NMR (282 MHz, $CDCl_3$) δ –121.8 (d, *J* = 54.0 Hz, 2F); ^{13}C NMR (150.9MHz, $CDCl_3$) δ 142.1, 132.3, 130.0, 129.8, 129.5, 125.6, 110.8 (t, *J* = 256.5 Hz), 109.9 (t, *J* = 3.0 Hz), 49.4 (t, *J* = 24.0 Hz), 23.0, 13.4; IR (KBr) 3035, 2973, 2875, 1494, 1461, 1367, 1123, 1105, 1082, 1033, 886, 761, 750, 705, 572 cm^{-1} ; m.p.: 32–34 ℃; MS (APCI, *m/z*) 279.1 $[M+MeOH+H]^+$; HRMS (APCI) Calcd. for $C_{14}H_{13}N_2F_2$ $[M+H]^+$: 247.1047, Found: 247.1044.

2-(2-(difluoromethyl)-3-methyl-1-phenylbutylidene)malononitrile (3k)

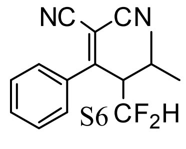

Purified through column chromatography on silica gel (Hexane/EtOAc = 98/2) to give **3k,** white solid, yield: 21.0 mg, 81%**.** ^{1}H NMR (300 MHz, $CDCl_3$) δ 7.44–7.47 (m, 3H), 7.21–7.24 (m, 2H), 6.30 (d, *J* = 9.0 Hz, 1H), 5.75 (t, *J* = 54.0 Hz, 1H), 2.11–2.18 (m, 1H), 0.98 (s , 3H), 0.96 (s , 3H); ^{19}F NMR (282 MHz, $CDCl_3$) δ –121.8 (d, *J* = 56.4 Hz, 2F); ^{13}C NMR (150.9MHz, $CDCl_3$) δ 147.1, 132.5, 130.0, 129.8, 129.5, 124.1, 110.8 (t, *J* = 256.5 Hz), 109.9, 49.4(t, *J* = 3.0 Hz), 29.2, 22.2; IR (KBr) 2964, 1467, 1365, 1119, 1108, 1062, 772, 750, 710, 576 cm^{-1} ; m.p.: 38–40 ℃; MS (APCI, *m/z*) 293.1 $[M+MeOH+H]^+$; HRMS (APCI) Calcd. for $C_{15}H_{15}N_2F_2$ $[M+H]^+$: 261.1203, Found: 261.1205.

2-(3,3-difluoro-2-methyl-2-phenylpropylidene)malononitrile (3l)

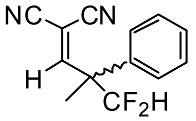

Purified through column chromatography on silica gel (Hexane/EtOAc = 99/1) to give **3l,** light yellow solid, yield: 10.0 mg, 43%**.** ^{1}H NMR (300 MHz, $CDCl_3$) δ 7.40 (s, 5H), 6.17 (t, *J* = 54.0 Hz, 1H), 5.60 (s, 1H), 2.47 (s, 3H); ^{19}F NMR (282 MHz, $CDCl_3$) δ –121.5 (d, *J* = 56.4 Hz, 2F); ^{13}C NMR (150.9MHz, $CDCl_3$) δ 151.1, 140.7, 129.6, 129.0, 126.2, 111.5 (t, *J* = 256.5 Hz), 40.2 (t, *J* = 25.6 Hz), 18.7; IR (KBr) 3064, 2259, 1628, 1497, 1446, 1366, 1268, 1141, 1118, 1092, 1021, 773, 758, 692 cm^{-1} ; m.p.: 37–39 ℃; MS (APCI, *m/z*) 265.1 $[M+MeOH+H]^+$; HRMS (APCI) Calcd. for $C_{13}H_{11}N_2F_2$ $[M+H]^+$: 233.0890, Found: 233.0907.

2-(3,3-difluoro-1-(4-methoxyphenyl)-2-methylpropylidene)malononitrile (3m)

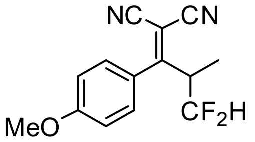

Purified through column chromatography on silica gel (Hexane/EtOAc = 95/5) to give **3m,** white solid, yield: 11.0 mg, 42%**.** ^{1}H NMR (300 MHz, $CDCl_3$) δ 7.15 (d, *J* = 8.7 Hz, 2H), 6.98 (d, *J* = 8.7 Hz, 2H), 6.61 (q, *J* = 6.0 Hz, 1H), 5.76 (t, *J* = 54.0 Hz, 1H), 3.84 (s, 3H), 1.62 (d, *J* = 6.0 Hz, 3H); ^{19}F NMR (282 MHz, $CDCl_3$) δ –122.0 (d, *J* = 53.6 Hz, 2F); ^{13}C NMR (150.9MHz, $CDCl_3$) δ 160.5, 135.6, 131.4, 126.7, 124.0, 115.0, 110.9 (t, *J* = 256.5 Hz), 110.0 (t, *J* = 3.0 Hz), 55.5, 49.6 (t, *J* = 25.6 Hz), 15.4; IR (KBr) 3021, 2964, 2840, 1608, 1512, 1456, 1360, 1288, 1248, 1177, 1140, 1097, 1075, 1027, 841, 753, 632 cm^{-1} ; m.p.: 50–53 ℃; MS (APCI, *m/z*) 295.1 $[M+MeOH+H]^+$; HRMS (APCI) Calcd. for $C_{14}H_{13}N_2OF_2$ $[M+H]^+$: 263.0996, Found: 263.0992.

2-(1-(4-bromophenyl)-3,3-difluoro-2-methylpropylidene)malononitrile (3n)

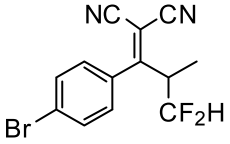

Purified through column chromatography on silica gel (Hexane/EtOAc = 97/3) to give **3n,** light yellow solid, yield: 20.0 mg, 64%**.** ^{1}H NMR (300 MHz, $CDCl_3$) δ 7.62 (d, *J* = 8.7 Hz, 2H), 7.12 (d, *J* = 8.7 Hz, 2H), 6.65 (q, *J* = 6.9 Hz, 1H), 5.77 (t, *J* = 54.0 Hz, 1H), 1.63 (d, *J* = 6.0 Hz, 3H); ^{19}F NMR (282 MHz, $CDCl_3$) δ –121.3 (d, *J* = 53.6 Hz, 2F); ^{13}C NMR (150.9MHz, $CDCl_3$) δ 136.3, 132.9, 131.7, 131.1, 125.9, 124.4, 110.7 (t, *J* = 256.5 Hz), 109.7 (t, *J* = 3.0 Hz), 49.1 (t, *J* = 25.6 Hz), 15.5; IR (KBr) 3004, 1587, 1488, 1439, 1358, 1130, 1116, 1074, 1038, 1013, 858, 842, 820, 752, 730 cm^{-1} ; m.p.: 101–104 ℃; MS (APCI, *m/z*) 343.0, 345.0 $[M+MeOH+H]^+$; HRMS (APCI) Calcd. for $C_{13}H_{10}N_2F_2Br$ $[M+H]^+$: 310.9995, Found: 310.9999.

2-(3,3-difluoro-1-(6-methoxynaphthalen-2-yl)-2-methylpropylidene)malononitrile (3o)

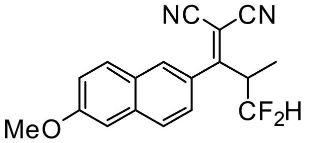

Purified through column chromatography on silica gel (Hexane/EtOAc = 95/5) to give **3o,** light yellow solid, yield: 14.7 mg, 47%**.** ^{1}H NMR (300 MHz, $CDCl_3$) δ 7.83 (d, *J* = 9.0 Hz, 2H), 7.77 (d, *J* = 9.0 Hz, 2H), 7.65 (s, 1H), 7.16–7.27 (m, 3H), 6.70 (q, *J* = 6.9 Hz, 1H), 5.80 (t, *J* = 54.0 Hz, 1H), 3.95 (s, 3H), 1.64 (d, *J* = 6.0 Hz, 3H); ^{19}F NMR (282 MHz, $CDCl_3$) δ –121.8 (d, *J* = 56.4 Hz, 2F); ^{13}C NMR (150.9MHz, $CDCl_3$) δ 159.0, 135.7, 134.9, 129.9, 129.5, 128.8, 128.3, 127.4, 127.1, 127.0, 120.2, 110.9 (t, *J* = 256.5 Hz), 110.0, 105.8, 55.6, 49.6 (t, *J* = 25.6 Hz), 15.5; IR (KBr) 3007, 1628, 1604, 1483, 1391, 1264, 1226, 1197, 1132, 1106, 1074, 1027, 851, 839, 753 cm^{-1} ; m.p.: 69–71 ℃; MS (APCI, *m/z*) 345.1 $[M+MeOH+H]^+$; HRMS (APCI) Calcd. for $C_{18}H_{15}N_2OF_2$ $[M+H]^+$: 313.1152, Found: 313.1147.

2-(3,3-difluoro-1-(4-methoxyphenyl)propylidene)malononitrile (3p)

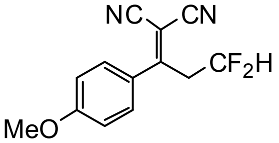

Purified through column chromatography on silica gel (Hexane/EtOAc = 98/2) to give **3p,** white solid, yield: 6.0 mg, 24%**.** ^{1}H NMR (300 MHz, $CDCl_3$) δ 7.31–7.34 (m, 2H), 6.93-6.97 (m, 2H), 6.08

(s, 1H), 5.86 (t, J = 54.0 Hz, 1H), 5.69 (s, 1H), 3.84 (s, 3H); ^{19}F NMR (282 MHz, $CDCl_3$) δ −121.1 (d, J = 56.4 Hz, 2F); ^{13}C NMR (150.9MHz, $CDCl_3$) δ 160.9, 134.7, 130.2, 126.6, 125.0, 114.7, 110.8 (t, J = 256.5 Hz), 109.8 (t, J = 3.0 Hz), 55.6, 48.5 (t, J = 30.0 Hz); IR (KBr) 2963, 1604, 1514, 1448, 1347, 1294, 1251, 1188, 1138, 1116, 1030, 945, 841, 756 cm^{-1} ; m.p.: 41–43 ℃; MS (APCI, *m/z*) 281.1 $[M+MeOH+H]^+$; HRMS (APCI) Calcd. for $C_{13}H_{11}N_2OF_2$ $[M+H]^+$: 249.0839, Found: 249.0831.

2-(1-(4-bromophenyl)-3,3-difluoropropylidene)malononitrile (3q)

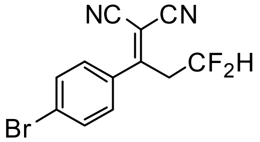

Purified through column chromatography on silica gel (Hexane/EtOAc = 98/2) to give **3q,** light yellow solid, yield: 7.0 mg, 24%**.** ^{1}H NMR (300 MHz, $CDCl_3$) δ 7.59 (d, J = 8.7 Hz, 2H), 7.27 (d, J = 8.7 Hz, 2H), 6.16 (s, 1H), 5.87 (t, J = 54.0 Hz, 1H), 5.75 (s, 1H); ^{19}F NMR (282 MHz, $CDCl_3$) δ −120.3 (d, J = 56.4 Hz, 2F); ^{13}C NMR (150.9MHz, $CDCl_3$) δ 134.1, 133.4, 132.6, 130.5, 126.2, 124.8, 110.7 (t, J = 256.5 Hz), 109.5 (t, J = 3.0 Hz), 48.1 (t, J = 30.0 Hz); IR (KBr) 3004, 1923, 1640, 1587, 1359, 1129, 1119, 1082, 1070, 1048, 1012, 946, 838, 750, 734 cm^{-1} ; m.p.: 54–56 ℃; MS (APCI, *m/z*) 328.9, 331.0 $[M+MeOH+H]^+$; HRMS (APCI) Calcd. for $C_{12}H_8N_2F_2Br$ $[M+H]^+$: 296.9839, Found: 296.9840.

2-(3,3-difluoro-1-(6-methoxynaphthalen-2-yl)propylidene)malononitrile (3r)

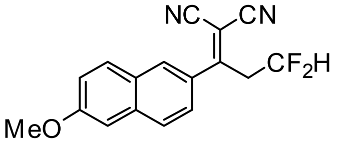

Purified through column chromatography on silica gel (Hexane/EtOAc = 95/5) to give **3r,** white solid, yield: 8.0 mg, 27%**.** ^{1}H NMR (300 MHz, $CDCl_3$) δ 7.76–7.81 (m, 3H), 7.43 (d, J = 8.7 Hz, 1H), 7.21–7.24 (m, 2H), 7.43 (d, J = 8.7 Hz, 1H), 6.19 (s, 1H), 5.90 (t, J = 54.0 Hz, 1H), 5.80 (s, 1H), 3.95 (s, 3H); ^{19}F NMR (282 MHz, $CDCl_3$) δ −120.9 (d, J = 56.4 Hz, 2F); ^{13}C NMR (150.9MHz, $CDCl_3$) δ 159.1, 135.2, 135.1, 130.0, 129.5, 128.5, 128.4, 128.0, 126.2, 125.5, 120.4, 110.8 (t, J = 256.5 Hz), 109.8, 105.7, 55.6, 48.5 (t, J = 25.6 Hz); IR (KBr) 2971, 1631, 1604, 1500, 1488, 1392, 1362, 1273, 1248, 1208, 1165, 1119, 1029, 956, 857, 822, 766, 755 cm^{-1} ; m.p.: 100–102 ℃; MS (APCI, *m/z*) 331.1 $[M+MeOH+H]^+$; HRMS (APCI) Calcd. for $C_{17}H_{13}N_2OF_2$ $[M+H]^+$: 299.0996, Found: 299.0998.

General procedure for electrophilic difluoromethylation of β-ketoesters:

To a stirred solution of β-ketoesters (0.22 mmol) in 2 mL dry CH_2Cl_2 was added corresponding base (0.13 mmol) at −75 °C under an inert atmosphere. After stirring for 30 min at −75 °C, reagent **1b** (0.10 mmol) was added to reaction mixture in one portion at the same temperature, and the resulting mixture was maintained for 1hr at −75 °C, then warmed to room temperature naturally. The reaction mixture was concentrated under reduced pressure and the residue was subject to column chromatography on silica gel to afford pure desired products (eluent: hexane/ethyl acetate).

Methyl 2-(difluoromethyl)-1-oxo-2,3-dihydro-1H-indene-2-carboxylate (6a).[3a]

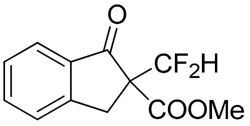

Purified through column chromatography on silica gel (Hexane/EtOAc = 98/2, 95/5) to give **6a, 7a** and **8a.**

Yollow semisolid, 14.0 mg, 58%. ^{1}H NMR (300 MHz, $CDCl_3$) δ 7.78 (d, J = 7.5 Hz, 1H), 7.66–7.71 (m, 1H), 7.57 (d, J = 7.8 Hz,1H), 7.43 (t, J = 7.5 Hz, 1H), 6.60 (t, J = 55.2 Hz, 1H), 3.80 (s, 3H), 3.73, 3.56 (ABq, J_{AB} = 17.4 Hz, 2H); ^{19}F NMR (282 MHz, $CDCl_3$) δ −126.5 (dd, J = 287.1, 54.4 Hz, 1F), −129.8 (dd, J = 287.1, 54.4 Hz, 1F); ^{13}C NMR (150.9MHz, $CDCl_3$) δ 195.9 (d, J = 7.5 Hz), 166.6 (d, J = 12.0 Hz), 154.0, 136.3, 134.1 (d, J = 3.0 Hz), 128.3, 126.6, 125.5, 115.6 (dd, J = 247.5, 241.4 Hz), 64.7 (dd, J = 25.6, 21.1 Hz), 53.6, 30.0 (d, J = 3.0 Hz); IR (neat) 3007, 2958, 1748, 1720, 1606, 1590, 1465, 1213, 1076, 1046, 758 cm^{-1} ; MS (ESI, *m/z*) 263.0 $[M+Na]^+$; HRMS (ESI) Calcd. for $C_{12}H_{10}F_2O_3Na$ $[M+Na]^+$: 263.0496, Found: 263.0503.

Methyl 3-(difluoromethoxy)-1H-indene-2-carboxylate (7a).[3a]

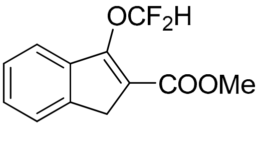

Yollow solid, 4.0 mg, 17%. ^{1}H NMR (300 MHz, $CDCl_3$) δ 7.63–7.66 (m, 1H), 7.39–7.49 (m, 3H, partly overlapping signal), 7.18 (t, J = 75.0 Hz, 1H, partly overlapping signal), 3.86 (s, 3H), 3.73 (s, 2H); ^{19}F NMR (282 MHz, $CDCl_3$) δ −82.2 (d, J = 74.4 Hz); ^{13}C NMR (150.9MHz, $CDCl_3$) δ 164.1, 156.2, 141.6, 138.6, 129.4, 127.4, 124.5, 121.2, 117.0, 116.7 (t, J = 262.1 Hz), 51.9, 36.2; IR (KBr) 3094, 2954, 1708, 1616, 1598, 1578, 1469, 1400, 1354, 1266, 1152, 1099, 761 cm^{-1}; m.p.: 60–61 ℃; MS (ESI, *m/z*) 263.3 $[M+Na]^+$; HRMS (ESI) Calcd. for $C_{12}H_{10}F_2O_3Na$ $[M+Na]^+$: 263.0496, Found: 263.0502.

methyl 2-bromo-1-oxo-2,3-dihydro-1H-indene-2-carboxylate (8a)

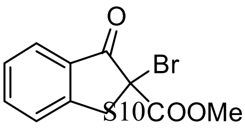

White solid, 22mg, 82%.^{1}H NMR (300 MHz, $CDCl_3$) δ 7.88 (d, *J* = 9.0 Hz, 1H), 7.71 (t, *J* = 7.5 Hz, 1H), 7.45–7.50 (m, 2H), 4.23, 3.69 (ABq, J_{AB} = 18.0 Hz, 2H), 3.84 (s, 3H); ^{13}C NMR (150.9MHz, $CDCl_3$) δ 195.2, 167.8, 150.3, 136.6, 132.4, 128.8, 126.5, 126.2, 58.3, 54.5, 44.0; IR (KBr) 2957, 1751, 1718, 1604, 1589, 1467, 1427, 1276, 1245, 1216, 1179, 1064, 1030, 1012, 878, 781, 756, 685, 639 cm^{-1} ; m.p.: 68–70 ℃; MS (ESI, *m/z*) 291.2, 292.8 $[M+Na]^+$; HRMS (ESI) Calcd. for $C_{11}H_9O_3NaBr$ $[M+Na]^+$: 290.9633, Found: 290.9637.

Methyl 2-(difluoromethyl)-6-methyl-1-oxo-2,3-dihydro-1H-indene-2-carboxylate (6b)

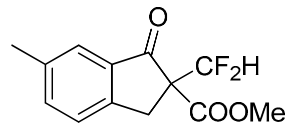

Purified through column chromatography on silica gel (Hexane/EtOAc = 99/1, 98/2) to give **6b, 7b** and **8b.**

White solid, 15.0 mg, 59 %. ^{1}H NMR (300 MHz, $CDCl_3$) δ 7.57 (s, 1H), 7.47 (dd, *J* = 16.2, 8.7 Hz, 2H), 6.58 (t, *J* = 55.5 Hz, 1H), 3.78 (s, 3H), 3.66, 3.50 (ABq, J_{AB} = 16.8 Hz, 2H), 2.41 (s, 3H); ^{19}F NMR (282 MHz, $CDCl_3$) δ –126.5 (dd, *J* = 287.9, 54.3 Hz, 1F), –130.0 (dd, *J* = 287.9, 54.3 Hz, 1F); ^{13}C NMR (150.9MHz, $CDCl_3$) δ 195.9 (d, *J* = 6.0 Hz), 166.7 (d, *J* = 12.1 Hz), 151.5, 138.4, 137.6, 134.3 (d, *J* = 10.6 Hz), 115.6 (dd, *J* = 247.5, 241.4 Hz), 65.0 (dd, *J* = 24.1, 21.1 Hz), 53.5, 29.7, 21.1; IR (KBr) 3031, 2959, 1617, 1583, 1494, 1381, 1284, 1212, 1078, 1042, 824 cm^{-1}; m.p.: 64–66 ℃ ; MS (ESI, *m/z*) 277.0 $[M+Na]^+$; HRMS (ESI) Calcd. for $C_{13}H_{12}F_2O_3Na$ $[M+Na]^+$:277.0652, Found: 277.0661.

Methyl 3-(difluoromethoxy)-5-methyl-1H-indene-2-carboxylate (7b)

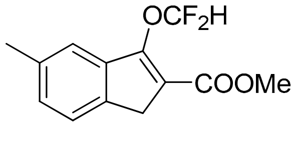

White solid, 4.0 mg, 16%. ^{1}H NMR (300 MHz, $CDCl_3$) δ 7.44 (s, 1H), 7.23–7.37 (m, *2*H), 7.17 (t, *J* = 75.0 Hz, 1H) , 3.85 (s, 3H), 3.67 (s, 2H), 2.43 (s, 3H); ^{19}F NMR (282 MHz, $CDCl_3$) δ –82.2 (d, *J* = 75.0 Hz); ^{13}C NMR (150.9MHz, $CDCl_3$) δ 164.1, 156.3 (d, *J* = 4.5 Hz), 138.8 (d, *J* = 15.1 Hz), 137.4, 130.5, 124.2, 121.5, 117.1, 116.7 (t, *J* = 262.6 Hz), 51.9, 35.8, 21.6; IR (KBr) 2952, 1704, 1623, 1601, 1578, 1436, 1355, 1255, 1200, 1145, 1025, 823 cm^{-1}; m.p.: 62–64 ℃; MS (ESI, *m/z*) 277.2 $[M+Na]^+$; HRMS (ESI) Calcd. for $C_{13}H_{12}F_2O_3Na$ $[M+Na]^+$:277.0652, Found: 277.0679.

methyl 2-bromo-6-methyl-1-oxo-2,3-dihydro-1H-indene-2-carboxylate (8b)

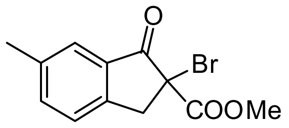

Light yellow solid 27.0 mg, 95%. ^{1}H NMR (300 MHz, $CDCl_3$) δ 7.66 (s, 1H), 7.52 (d, J = 7.2 Hz, 1H), 7.35 (d, J = 7.2 Hz, 1H), 4.17, 3.63 (ABq, J_{AB} = 18.0 Hz, 2H), 3.83 (s, 3H), 2.43 (s, 3H); ^{13}C NMR (150.9MHz, $CDCl_3$) δ 195.3, 167.9, 147.7, 139.0, 137.8, 132.5, 126.1, 126.0, 58.8, 54.4, 43.7 21.3; IR (KBr) 3415, 2954, 2921, 1762, 1715, 1617, 1582, 1492, 1435, 1414, 1278, 1250, 1222, 1181, 1152, 1120, 1036, 1010, 940, 823, 725, 689, 644 cm^{-1}; m.p.: 80–82 ℃; MS (ESI, *m/z*) 305.4, 307.2 $[M+Na]^+$; HRMS (ESI) Calcd. for $C_{12}H_{11}O_3NaBr$ $[M+Na]^+$: 304.9789, Found: 304.9792.

Methyl 2-(difluoromethyl)-6-methoxy-1-oxo-2,3-dihydro-1H-indene-2-carboxylate (6c)

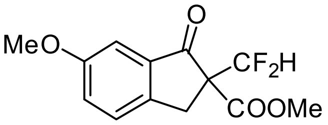

Purified through column chromatography on silica gel (Hexane/EtOAc = 98/2, 95/5) to give **6c, 7c** and **8c.**

White solid, 16.0 mg, 59%. ^{1}H NMR (300 MHz, $CDCl_3$) δ 7.45 (d, J = 8.1 Hz, 1H), 7.27 (dd, J = 8.1, 2.4 Hz, 1H), 7.18 (d, J = 2.4 Hz, 1H), 6.58 (t, J = 54.9 Hz, 1H), 3.84 (s, 3H), 3.79 (s, 3H), 3.63, 3.47 (ABq, J_{AB} = 17.4 Hz, 2H); ^{19}F NMR (282 MHz, $CDCl_3$) δ –126.5 (dd, J = 287.9, 54.4 Hz, 1F), –130.0 (dd, J = 287.9, 54.4 Hz, 1F); ^{13}C NMR (150.9MHz, $CDCl_3$) δ 195.8 (d, J = 7.5 Hz), 166.6 (d, J = 12.1 Hz), 160.0, 147.1, 135.3 (d, J = 4.5 Hz), 127.3, 126.0, 115.5 (dd, J = 247.5, 239.9 Hz), 106.2, 65.4 (dd, J = 24.1, 21.1 Hz), 55.8, 53.6, 29.4; IR (KBr) 3041, 2957, 2834, 1743, 1709, 1617, 1492, 1451, 1434, 1313, 1279, 1200, 1074, 845, 763 cm^{-1}; m.p.: 77–78 ℃; MS (ESI, *m/z*) 293.1 $[M+Na]^+$; HRMS (ESI) Calcd. for $C_{13}H_{12}F_2O_4Na$ $[M+Na]^+$:293.0601, Found: 293.0617.

Methyl 3-(difluoromethoxy)-5-methoxy-1H-indene-2-carboxylate (7c)

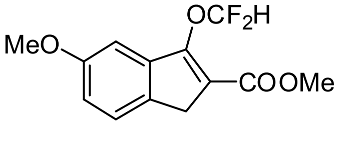

White solid, 5.0 mg, 19%.^{1}H NMR (300 MHz, $CDCl_3$) δ 7.36 (d, J = 8.4 Hz, 1H), 7.12 (d, J = 2.4 Hz, 1H), 7.00 (dd, J = 8.4, 2.4 Hz, 1H), 7.19 (t, J = 75.0 Hz, 1H), 3.87 (s, 3H), 3.85 (s, 3H), 3.66 (s, 2H); ^{19}F NMR (282 MHz, $CDCl_3$) δ –82.2 (d, J = 75.3 Hz); ^{13}C NMR (150.9MHz, $CDCl_3$) δ 195.8 (d, J = 7.5 Hz), 166.6 (d, J = 12.1 Hz), 160.0 (d, J = 15.1 Hz), 147.1, 135.3 (d, J = 4.5 Hz), 127.3,

126.0, 115.5 (dd, J = 247.5, 239.9 Hz), 106.2, 65.4 (dd, J = 24.1, 21.1 Hz), 55.8, 53.6, 29.4; IR (KBr) 2949, 1706, 1605, 1580, 1487, 1356, 1260, 1231, 1129, 1082, 808, 745 cm^{-1}; m.p.: 62–64 ℃; MS (ESI, *m/z*) 293.2 $[M+Na]^+$; HRMS (ESI) Calcd. for $C_{13}H_{12}F_2O_4Na$ $[M+Na]^+$:293.0601, Found: 293.0603.

Methyl 2-bromo-6-methoxy-1-oxo-2,3-dihydro-1H-indene-2-carboxylate (8c)

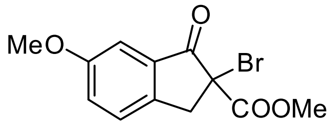

Red solid, 27.0 mg, 90%. ^1H NMR (300 MHz, $CDCl_3$) δ 7.27–7.37 (m, 3H), 4.14, 3.61 (ABq, J_{AB} = 18.0 Hz, 2H), 3.86 (s, 3H), 3.83 (s, 3H); ^{13}C NMR (150.9MHz, $CDCl_3$) δ 195.2, 167.8, 160.4, 143.2, 133.6, 127.2, 126.2, 106.9, 59.0, 55.9, 54.4, 43.4; IR (KBr) 2957, 1751, 1712, 1617, 1491, 1434, 1299, 1281, 1241, 1228, 1173, 1020, 852, 741 cm^{-1}; m.p.: 77–78 ℃; MS (ESI, *m/z*) 321.2, 323.3 $[M+Na]^+$; HRMS (ESI) Calcd. for $C_{12}H_{11}O_4NaBr$ $[M+Na]^+$:320.9738, Found: 320.9742.

Methyl 2-(difluoromethyl)-5,6-dimethoxy-1-oxo-2,3-dihydro-1H-indene-2-Carboxylate (6d)

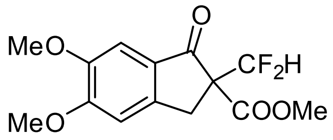

Purified through column chromatography on silica gel (Hexane/EtOAc = 98/2, 95/5) to give **6d, 7d** and **8d.**

White solid, 19.0 mg, 63%. ^1H NMR (300 MHz, $CDCl_3$) δ 7.16 (s, 1H), 6.97 (s, 1H), 6.58 (t, J = 57.0 Hz, 1H), 4.01 (s, 3H), 3.91 (s, 3H), 3.80 (s, 3H), 3.62, 3.45 (ABq, J_{AB} = 17.4 Hz, 2H); ^{19}F NMR (282 MHz, $CDCl_3$) δ −126.7 (dd, J = 286.8, 55.3 Hz, 1F), −130.2 (dd, J = 286.8, 55.3 Hz, 1F); ^{13}C NMR (150.9MHz, $CDCl_3$) δ 194.2 (d, J = 7.5 Hz), 167.0 (d, J = 12.1 Hz), 156.9, 150.2, 150.1, 126.8 (d, J = 4.5 Hz), 115.7 (dd, J = 247.5, 241.4 Hz), 107.4, 105.3, 65.1 (dd, J = 24.1, 21.1 Hz), 56.6, 56.3, 53.6, 29.8; IR (KBr) 2966, 1742, 1692, 1591, 1505, 1465, 1439, 1373, 1324, 1284, 1268, 1250, 1127, 1081, 1047, 855, 771, 715 cm^{-1}; m.p.: 105–106 ℃; MS (ESI, *m/z*) 323.2 $[M+Na]^+$; HRMS (ESI) Calcd. for $C_{14}H_{14}F_2O_5Na$ $[M+Na]^+$:323.0707, Found: 323.0727.

Methyl 3-(difluoromethoxy)-5,6-dimethoxy-1H-indene-2-carboxylate (7d)

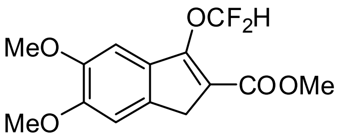

White solid, 5.0 mg, 17%. ^1H NMR (300 MHz, $CDCl_3$) δ 7.23 (t, J = 75.0 Hz, 1H), 7.09 (s, 1H), 7.00

(s, 1H), 3.95 (s, 3H), 3.94 (s, 3H), 3.84 (s, 3H), 3.65 (s, 2H); ^{19}F NMR (282 MHz, $CDCl_3$) δ -82.3 (d, J = 75.3 Hz); ^{13}C NMR (150.9MHz, $CDCl_3$) δ 164.1, 156.7, 151.5, 149.4, 135.5, 131.1, 116.8 (t, J = 261.1 Hz), 114.8, 107.5, 103.3, 56.4 (d, J = 4.5 Hz), 51.8, 36.0; IR (KBr) 3007, 2956, 1701, 1573, 1499, 1438, 1397, 1371, 1330, 1245, 1223, 1210, 1184, 1149, 1125, 1105, 1074, 1057, 1006, 864 cm^{-1}; m.p.: 130–132 ℃; MS (ESI, *m/z*) 323.5 $[M+Na]^+$; Calcd. for $C_{14}H_{14}F_2O_5Na$ $[M+Na]^+$:323.0707, Found: 323.0713.

Methyl 2-bromo-5,6-dimethoxy-1-oxo-2,3-dihydro-1H-indene-2-carboxylate (8d)

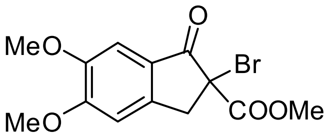

Brown solid, 30.0 mg, 19%.^{1}H NMR (300 MHz, $CDCl_3$) δ 7.24 (s, 1H), 6.86 (s, 1H), 4.00 (s, 3H), 3.93 (s, 3H), 4.15, 3.60 (ABq, J_{AB} = 17.7 Hz, 2H), 3.83 (s, 3H); ^{13}C NMR (150.9MHz, $CDCl_3$) δ 193.8, 168.0, 157.2, 150.5, 146.2, 125.0, 107.2, 106.0, 58.9, 56.7, 56.4, 54.4, 43.8; IR (KBr) 3078, 2954, 1740, 1719, 1701, 1590, 1505, 1467, 1440, 1314, 1265, 1224, 1035, 1008, 857 cm^{-1}; m.p.: 120–122 ℃; MS (ESI, *m/z*) 351.4, 353.4 $[M+Na]^+$; Calcd. for $C_{13}H_{13}O_5NaBr$ $[M+Na]^+$:350.9844, Found: 350.9867.

Methyl 5-bromo-2-(difluoromethyl)-1-oxo-2,3-dihydro-1H-indene-2-carboxylate (6e)

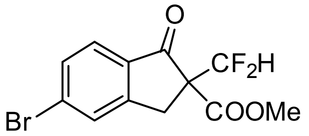

Purified through column chromatography on silica gel (Hexane/EtOAc = 98/2, 95/5) to give **6e, 7e** and **8e.**

White solid, 20.0 mg, 63%. ^{1}H NMR (300 MHz, $CDCl_3$) δ 7.76 (s, 1H), 7.60 (dd, J = 19.8, 8.4 Hz, 2H), 6.58 (t, J = 55.2 Hz, 1H), 3.80 (s, 3H), 3.71, 3.54 (ABq, J_{AB} = 18.0 Hz, 2H); ^{19}F NMR (282 MHz, $CDCl_3$) δ −126.4 (dd, J = 287.9, 54.4 Hz, 1F), −129.6 (dd, J = 288.2, 55.5 Hz, 1F); ^{13}C NMR (150.9MHz, $CDCl_3$) δ 194.7 (d, J = 6.0 Hz), 166.1 (d, J = 12.1 Hz), 155.4, 133.0 (d, J = 3.0 Hz), 132.0 (d, J = 7.5 Hz), 130.0, 126.5, 115.3 (dd, J = 247.5, 239.9 Hz), 64.8-64.7 (m), 53.7, 29.7; IR (KBr) 2965, 1752, 1714, 1598, 1576, 1432, 1323, 1272, 1261, 1167, 1082, 827 cm^{-1}; m.p.: 87–88 ℃; MS (ESI, *m/z*) 341.3 $[M+Na]^+$; HRMS (ESI) Calcd. for $C_{12}H_9F_2O_3NaBr$ $[M+Na]^+$:340.9601, Found: 340.9610.

Methyl 6-bromo-3-(difluoromethoxy)-1H-indene-2-carboxylate (7e)

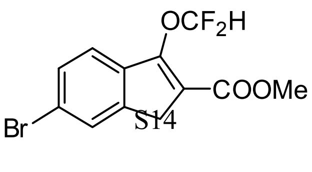

White solid, 9.0 mg, 28%. ^{1}H NMR (300 MHz, $CDCl_3$) δ 7.63 (s, 1H), 7.52 (dd, *J* = 19.5, 8.4 Hz, 2H), 7.19 (t, *J* = 75.3 Hz, 1H), 3.85 (s, 3H), 3.70 (s, 2H); ^{19}F NMR (282 MHz, $CDCl_3$) δ –82.5 (d, *J* = 74.2 Hz); ^{13}C NMR (150.9MHz, $CDCl_3$) δ 163.8, 155.4, 143.3, 137.6, 130.8, 127.9, 124.0, 122.5, 117.1, 116.5 (t, *J* = 262.6 Hz), 52.1, 36.0; IR (KBr) 3030, 2954, 2927, 1610, 1593, 1568, 1434, 1360, 1323, 1255, 1126, 1085, 1046, 835 cm^{-1}; m.p.: 83–85 ℃; MS (ESI, *m/z*) 341.5 $[M+Na]^+$; HRMS (ESI) Calcd. for $C_{12}H_9F_2O_3NaBr$ $[M+Na]^+$:340.9601, Found: 340.9600.

Methyl 2,5-dibromo-1-oxo-2,3-dihydro-1H-indene-2-carboxylate (8e)

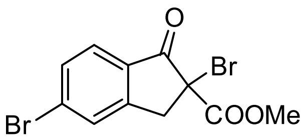

Yellow solid, 32.0 mg, 92%. ^{1}H NMR (300 MHz, $CDCl_3$) δ 7.73 (d, *J* = 8.1 Hz, 1H), 7.66 (s, 1H), 7.62 (d, *J* = 8.1 Hz, 1H), 4.21 (d, *J* = 18.3 Hz, 1H), 3.84 (s, 3H), 3.66 (d, *J* = 18.3 Hz, 1H); ^{13}C NMR (150.9MHz, $CDCl_3$) δ 194.0, 167.3, 151.8, 132.5, 132.2, 131.2, 129.8, 127.2, 57.8, 54.6, 43.6; IR (KBr) 2955, 1759, 1722, 1597, 1580, 1434, 1424, 1315, 1263, 1242, 1210, 1176, 1075, 1056, 1033, 1007, 946, 890, 853, 838, 807, 774, 739, 712, 643, 583 cm^{-1}; m.p.: 110–111 ℃; MS (ESI, *m/z*) 369.4, 371.1, 373.3 $[M+Na]^+$; HRMS (ESI) Calcd. for $C_{11}H_8O_3NaBr_2$ $[M+Na]^+$: 368.8738, Found: 368.8741.

Methyl 5-chloro-2-(difluoromethyl)-1-oxo-2,3-dihydro-1H-indene-2-carboxylate (6f)

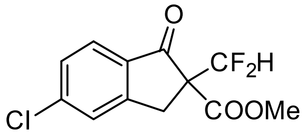

Purified through column chromatography on silica gel (Hexane/EtOAc = 98/2, 95/5) to give **6f, 7f** and **8f.**

White solid, 17.0 mg, 62%. ^{1}H NMR (300 MHz, $CDCl_3$) δ 7.71 (d, *J* = 8.1 Hz, 1H), 7.57 (s, 1H), 7.41 (d, *J* = 8.4 Hz, 1H), 6.58 (t, *J* = 55.2 Hz, 1H), 3.80 (s, 3H), 3.70, 3.53 (ABq, J_{AB} = 18.0 Hz, 2H); ^{19}F NMR (282 MHz, $CDCl_3$) δ –126.4 (dd, *J* = 286.8, 54.4 Hz, 1F), –129.6 (dd, *J* = 287.9, 55.3 Hz, 1F); ^{13}C NMR (150.9MHz, $CDCl_3$) δ 194.4 (d, *J* = 6.0 Hz), 166.2 (d, *J* = 12.1 Hz), 155.3, 143.2, 132.6 (d, *J* = 4.5 Hz), 129.2, 126.9, 126.5, 115.3 (dd, *J* = 247.5, 241.4 Hz), 64.8-64.7 (t, *J* = 21.1 Hz), 53.7, 29.7; IR (KBr) 2965, 1752, 1714, 1600, 1579, 1430, 1375, 1326, 1274, 1257, 1082, 1045, 834, 707 cm^{-1}; m.p.: 88–89 ℃; MS (ESI, *m/z*) 297.1 $[M+Na]^+$; HRMS (ESI) Calcd. for $C_{12}H_9F_2O_3NaCl$ $[M+Na]^+$:297.0106, Found: 297.0106.

Methyl 6-chloro-3-(difluoromethoxy)-1H-indene-2-carboxylate (7f)

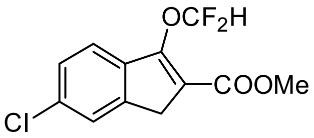

White solid, 7.0 mg, 26%.^{1}H NMR (300 MHz, $CDCl_3$) δ 7.56–7.38 (m, 3H), 7.19 (t, J = 75.0 Hz, 1H), 3.85 (s, 3H), 3.71 (s, 2H); ^{19}F NMR (282 MHz, $CDCl_3$) δ –82.4 (d, J = 75.3 Hz); ^{13}C NMR (150.9MHz, $CDCl_3$) δ 163.8, 155.3, 143.0, 137.2, 135.8, 128.0, 125.0, 122.2, 117.2, 116.5 (t, J = 262.6 Hz), 52.1, 36.0; IR (KBr) 2957, 1702, 1618, 1571, 1434, 1357, 1260, 1123, 1065, 820 cm^{-1}; m.p.: 88–90 ℃;MS (ESI, *m/z*) 297.3 [M+Na]$^+$; HRMS (ESI) Calcd. for $C_{12}H_9F_2O_3NaCl$ [M+Na]$^+$: 297.0106, Found: 297.0114.

Methyl 2-bromo-5-chloro-1-oxo-2,3-dihydro-1H-indene-2-carboxylate (8f known)[3b]

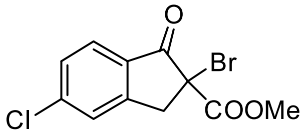

Light yellow solid, 28.0 mg, 92%.^{1}H NMR (300 MHz, $CDCl_3$) δ 7.79 (d, J = 8.1 Hz, 1H), 7.46 (s, 1H), 7.45 (d, J = 8.1 Hz, 1H), 4.20 (d, J = 18.3 Hz, 1H), 3.84 (s, 3H), 3.65 (d, J = 18.3 Hz, 1H); ^{13}C NMR (150.9MHz, $CDCl_3$) δ 193.8, 167.4, 151.7, 143.3, 130.8, 129.7, 127.2, 126.7, 57.9, 54.6, 43.6.

Benzyl 2-(difluoromethyl)-1-oxo-2,3-dihydro-1H-indene-2-carboxylate (6g).

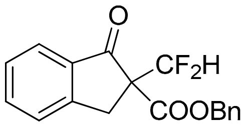

Purified through column chromatography on silica gel (Hexane/EtOAc = 98/2, 95/5) to give **6g, 7g** and **8g.**

Colorless oil, 16.0 mg, 51%.^{1}H NMR (300 MHz, $CDCl_3$) δ 7.78 (d, J = 7.8 Hz, 1H), 7.67–7.70 (m, 1H), 7.61 (d, J = 24.0 Hz, 1H), 7.27–7.45 (m, 6H), 6.60 (t, J = 54.0 Hz, 1H), 3.79 (s, 3H), 5.23 (dd, J = 15.0, 12.6 Hz, 2H), 3.74, 3.56 (ABq, J_{AB} = 17.4 Hz, 2H); ^{19}F NMR (282 MHz, $CDCl_3$) δ –126.3 (dd, J = 287.9, 55.3 Hz, 1F), –129.6 (dd, J = 287.9, 55.3 Hz, 1F); ^{13}C NMR (150.9MHz, $CDCl_3$) δ 195.8 (d, J = 7.5 Hz), 166.0 (d, J = 12.0 Hz), 154.0, 136.4, 135.0, 134.2 (d, J = 4.5 Hz), 128.8, 128.7, 128.3, 128.0, 126.7, 125.5, 115.6 (dd, J = 247.5, 241.4 Hz), 68.2, 65.0 (dd, J = 24.1, 21.1 Hz), 30.1; IR (neat) 3025, 1747, 1721, 1606, 1590, 1498, 1466, 1456, 1373, 1330, 1260, 1213, 1157, 1118, 1076, 1044, 911, 805, 785, 752, 696, 676 cm^{-1} ; MS (ESI, *m/z*) 339.5 [M+Na]$^+$; HRMS (ESI) Calcd. for $C_{18}H_{14}F_2O_3Na$ [M+Na]$^+$: 339.0809, Found: 339.0837.

Benzyl 3-(difluoromethoxy)-1H-indene-2-carboxylate (7g)

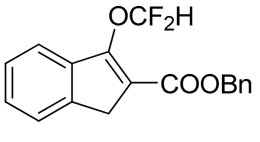

Yellow solid, 8.0 mg, 25%.^{1}H NMR (300 MHz, $CDCl_3$) δ 7.62–7.65 (m, 1H), 7.33–7.45 (m, 8H, partly overlapping signal), 7.16 (t, *J* = 75.0 Hz, 1H, partly overlapping signal), 5.30 (s, 2H), 3.75 (s, 2H); ^{19}F NMR (282 MHz, $CDCl_3$) δ −82.3 (d, *J* = 74.4 Hz); ^{13}C NMR (150.9MHz, $CDCl_3$) δ 163.5, 156.5, 141.8, 138.7, 135.9, 125.5, 128.8, 128.6, 128.4, 127.4, 124.5 121.3, 117.1, 116.6 (t, *J* = 262.6 Hz), 66.6, 36.3; IR (KBr) 3037, 1708, 1620, 1602, 1580, 1452, 1393, 1354, 1305, 1251, 1209, 1158, 1120, 1107, 1095, 1081, 1049, 989, 757, 730, 718, 690 cm^{-1}; m.p.: 45–47 ℃; MS (ESI, *m/z*) 339.6 $[M+Na]^+$; HRMS (ESI) Calcd. for $C_{18}H_{14}F_2O_3Na$ $[M+Na]^+$: 339.0809, Found: 339.0814.

Benzyl 2-bromo-1-oxo-2,3-dihydro-1H-indene-2-carboxylate (8g)

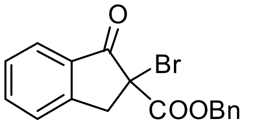

Light yellow oil, 32.0 mg, 93%. ^{1}H NMR (300 MHz, $CDCl_3$) δ 7.87 (d, *J* = 7.5 Hz, 1H), 7.71 (t, *J* = 7.5 Hz, 1H), 7.34–7.49 (m, 7H), 5.26 (d, *J* = 1.5 Hz, 2H), 4.19, 3.59 (ABq, J_{AB} = 18.0 Hz, 2H); ^{13}C NMR (150.9MHz, $CDCl_3$) δ195.1, 167.1, 150.2, 136.5, 135.0, 132.4, 128.8, 128.7 (d, *J* = 16.6 Hz), 128.2, 126.5, 126.2, 69.0, 58.5, 44.0; IR (neat) 3033, 1757, 1726, 1604, 1465, 1426, 1275, 1232, 1212, 1172, 1077, 997, 916, 781, 746, 696 cm^{-1} ; MS (ESI, *m/z*) 367.2, 369.3 $[M+Na]^+$; HRMS (ESI) Calcd. for $C_{17}H_{13}O_3NaBr$ $[M+Na]^+$: 366.9946, Found: 366.9958.

Ethyl 2-(difluoromethyl)-1-oxo-2,3-dihydro-1H-indene-2-carboxylate (6h)

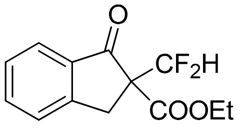

Purified through column chromatography on silica gel (Hexane/EtOAc = 98/2, 95/5) to give **6h, 7h** and **8h.**

Yellow semisolid, 15.0 mg, 59%. ^{1}H NMR (300 MHz, $CDCl_3$) δ 7.78 (d, *J* = 7.8 Hz, 1H), 7.65–7.70 (m, 1H), 7.57 (d, *J* = 7.8 Hz, 1H), 7.42 (t, *J* = 7.8 Hz, 1H), 6.60 (t, *J* = 55.2 Hz, 1H), 4.26 (q, *J* = 7.2 Hz, 2H), 3.72, 3.55 (ABq, J_{AB} = 17.4 Hz, 2H), 1.27 (t, *J* = 6.9 Hz, 3H); ^{19}F NMR (282 MHz, $CDCl_3$) δ −126.6 (dd, *J* = 287.1, 55.6 Hz, 1F), −129.7 (dd, *J* = 287.1, 55.6 Hz, 1F); ^{13}C NMR (150.9MHz, $CDCl_3$) δ 196.0, 166.0 (d, *J* = 12.0 Hz), 154.1, 136.2, 134.2 (d, *J* = 4.5 Hz), 128.2, 126.6, 125.4, 115.6 (t, *J* = 247.5 Hz), 64.8 (t, *J* = 22.6 Hz), 62.8, 30.0; IR (neat) 2985, 2939, 1746, 1721, 1607, 1590, 1433, 1370, 1260, 1076, 757 cm^{-1}; MS (ESI, *m/z*) 277.2 $[M+Na]^+$; HRMS (ESI) Calcd. for

$C_{13}H_{12}F_2O_3Na$ [M+Na]$^+$:277.0652, Found: 277.0663.

Ethyl 3-(difluoromethoxy)-1H-indene-2-carboxylate (7h)

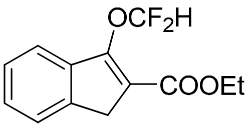

Yellow solid, 5.0 mg, 20%. ^{1}H NMR (300 MHz, $CDCl_3$) δ 7.62–7.65 (m, 1H), 7.41–7.49 (m, 3H, partly overlapping signal), 7.16 (t, *J* = 75.3 Hz, 1H, partly overlapping signal), 4.32 (q, *J* = 7.2 Hz, 2H), 3.73 (s, 2H), 1.37 (t, *J* = 7.2 Hz, 3H); ^{19}F NMR (282 MHz, $CDCl_3$) δ −82.1 (d, *J* = 74.2 Hz); ^{13}C NMR (150.9MHz, $CDCl_3$) δ 164.1, 156.2, 141.6, 138.6, 129.4, 127.4, 124.5, 121.2, 117.0, 116.7 (t, *J* = 262.1 Hz), 51.9, 36.2; IR (KBr) 2982, 2932, 1693, 1617, 1600, 1580, 1478, 1387, 1256, 1164, 1119, 761 cm^{-1}; m.p.: 40–42 ℃; MS (ESI, *m/z*) 277.3 [M+Na]$^+$; HRMS (ESI) Calcd. for $C_{13}H_{12}F_2O_3Na$ [M+Na]$^+$:277.0652, Found: 277.0653.

Ethyl 2-bromo-1-oxo-2,3-dihydro-1H-indene-2-carboxylate (8h Known)[3c]

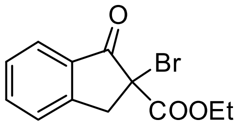

Light yollow oil, 26.0 mg, 92%. ^{1}H NMR (300 MHz, $CDCl_3$) δ 7.87 (d, *J* = 7.8 Hz, 1H), 7.70–7.73 (m, 1H), 7.45–7.49 (m, 2H), 4.29 (dq, *J* = 0.9, 14.4 Hz, 2H), 4.21, 3.68 (ABq, J_{AB} = 18.0 Hz, 2H), 1.29 (t, *J* = 6.9 Hz, 3H).

tert-Butyl 2-(difluoromethyl)-1-oxo-2,3-dihydro-1H-indene-2-carboxylate (6i)

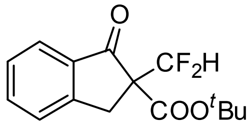

Purified through column chromatography on silica gel (Hexane/EtOAc = 99/1, 98/2) to give **6i, 7i** and **8i.**

Semisolid, 17.0 mg, 60%. ^{1}H NMR (300 MHz, $CDCl_3$) δ 7.77 (d, *J* = 7.8 Hz, 1H), 7.64–7.68 (m, 1H), 7.55 (d, *J* = 7.8 Hz, 1H), 7.38–7.43 (m, 1H), 6.54 (t, *J* = 55.5 Hz, 1H), 3.68, 3.50 (ABq, J_{AB} = 17.4 Hz, 2H), 1.50 (s, 9H); ^{19}F NMR (282 MHz, $CDCl_3$) δ −127.1 (dd, *J* = 285.9, 55.6 Hz, 1F), −129.2 (dd, *J* = 285.9, 55.6 Hz, 1F); ^{13}C NMR (150.9MHz, $CDCl_3$) δ 196.0, 166.0 (d, *J* = 12.0 Hz), 154.1, 136.2, 134.2 (d, *J* = 4.5 Hz), 128.2, 126.6, 125.4, 115.6 (t, *J* = 247.5 Hz), 64.8 (t, *J* = 22.6 Hz), 62.8, 30.0; IR (KBr) 2987, 1711, 1604, 1588, 1465, 1372, 1272, 1147, 1069, 841, 741 cm^{-1}; MS (ESI, *m/z*) 305.6 [M+Na]$^+$; HRMS (ESI) Calcd. for $C_{15}H_{16}F_2O_3Na$ [M+Na]$^+$:305.0965,

Found: 305.0968.

tert-Butyl 3-(difluoromethoxy)-1H-indene-2-carboxylate (7i)

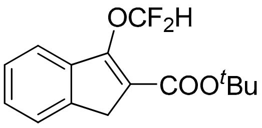

Semisolid, 4.0 mg, 14%. ^{1}H NMR (300 MHz, $CDCl_3$) δ 7.59-7.62 (m, 1H), 7.36-7.59 (m, 3H), 7.08 (t, J = 75.3 Hz, 1H), 3.68 (s, 3H), 1.57 (s, 9H); ^{19}F NMR (282 MHz, $CDCl_3$) δ -82.0 (d, J = 75.0 Hz); ^{13}C NMR (150.9MHz, $CDCl_3$) δ 163.0, 154.8, 141.5, 139.0, 129.0, 127.3, 124.4, 121.0, 120.0, 116.7 (t, J = 261.0 Hz, 1H), 81.8, 36.4, 28.4; IR (KBr) 2985, 2933, 1696, 1601, 1580, 1456, 1369, 1353, 1267, 1164, 1128, 1047, 757 cm^{-1}; MS (ESI, *m/z*) 305.2 $[M+Na]^+$; HRMS (ESI) Calcd. for $C_{15}H_{16}F_2O_3Na$ $[M+Na]^+$:305.0965, Found: 305.0967.

tert-Butyl 2-bromo-1-oxo-2,3-dihydro-1H-indene-2-carboxylate (8i Known)[3d]

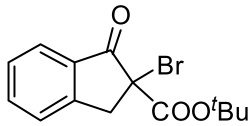

White solid, 30.0 mg, 96%.^{1}H NMR (300 MHz, $CDCl_3$) δ 7.86 (d, J = 7.8 Hz, 1H), 7.67 (d, J = 7.8 Hz, 1H), 7.46 (t, J = 3.0 Hz, 2H), 4.13, 3.66 (ABq, J_{AB} = 18.0 Hz, 2H), 1.46 (t, 9H).

Allyl 2-(difluoromethyl)-1-oxo-2,3-dihydro-1H-indene-2-carboxylate (6j).

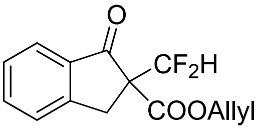

Purified through column chromatography on silica gel (Hexane/EtOAc = 99/1, 98/2) to give **6j, 7j** and **8j.**

Yellow oil, 13.0 mg, 49%. ^{1}H NMR (300 MHz, $CDCl_3$) δ 7.79 (d, J = 7.5 Hz, 1H), 7.68 (t, J = 7.4 Hz, 1H), 7.57 (d, J = 7.5 Hz, 1H), 7.43 (t, J = 7.4 Hz, 1H), 6.62 (t, J = 55.5 Hz, 1H), 5.80–5.91 (m, 1H), 5.23–5.32 (m, 2H), 4.69 (d, J = 5.4 Hz, 2H), 3.74, 3.57 (ABq, J_{AB} = 17.4 Hz, 2H); ^{19}F NMR (282 MHz, $CDCl_3$) δ −126.4 (dd, J = 287.1, 54.4 Hz, 1F), −129.7 (dd, J = 287.1, 54.4 Hz, 1F); ^{13}C NMR (150.9MHz, $CDCl_3$) δ 195.9 (d, J = 6.0 Hz), 165.8 (d, J = 12.1 Hz), 154.1, 136.4, 134.2 (d, J = 3.0 Hz), 131.1, 128.3, 126.7, 125.5, 119.2, 115.6 (dd, J = 247.5, 241.4 Hz), 67.0, 64.9 (t, J = 22.6 Hz), 30.1; IR (neat) 2943, 1748, 1721, 1606, 1590, 1466, 1371, 1256, 1212, 1157, 1119, 1076, 1042, 923, 807, 759, 677 cm^{-1}; MS (ESI, *m/z*) 289.2 $[M+Na]^+$; HRMS (ESI) Calcd. for $C_{14}H_{12}F_2O_3Na$ $[M+Na]^+$: 289.0652, Found: 289.0662.

Allyl 3-(difluoromethoxy)-1H-indene-2-carboxylate (7j)

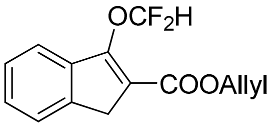

Yellow semisolid, 7.0 mg, 26%.^{1}H NMR (300 MHz, $CDCl_3$) δ 7.65 (d, *J* = 6.0 Hz 1H), 7.39–7.49 (m, 3H, partly overlapping signal), 7.18 (t, *J* = 75.0 Hz, 1H, partly overlapping signal), 5.95–6.06 (m, 1H), 5.40 (d, *J* = 17.4 Hz, 1H), 5.30 (d, *J* = 10.5 Hz, 1H), 4.76 (d, *J* = 5.7 Hz, 1H), 3.75 (s, 2H); ^{19}F NMR (282 MHz, $CDCl_3$) δ –82.0 (d, *J* = 75.0 Hz); ^{13}C NMR (150.9MHz, $CDCl_3$) δ 163.3, 156.4, 141.8, 138.7, 132.1, 129.5, 127.4, 124.5, 121.3, 118.8, 117.2, 116.7 (t, *J* = 262.6 Hz), 65.6, 36.2; IR (neat) 2932, 1706, 1618, 1599, 1578, 1395, 1355, 1321, 1249, 1210, 1121, 1098, 1074, 984, 758, 717 cm^{-1}; MS (ESI, *m/z*) 289.1 $[M+Na]^+$; HRMS (ESI) Calcd. for $C_{14}H_{12}F_2O_3Na$ $[M+Na]^+$: 289.0652, Found: 289.0658.

Allyl 2-bromo-1-oxo-2,3-dihydro-1H-indene-2-carboxylate (8j)

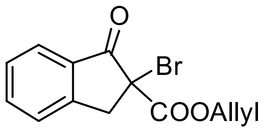

Yellow oil, 28.0 mg, 95%. ^{1}H NMR (300 MHz, $CDCl_3$) δ 7.88 (d, *J* = 7.8 Hz 1H), 7.68–7.786 (m, 1H), 7.45–7.52 (m, 2H) 5.83–5.96 (m, 1H), 5.24–5.38 (m, 2H), 4.70–4.73 (m, 2H), 4.22 (d, *J* = 18.0 Hz, 1H), 3.70 (d, *J* = 18.0 Hz, 1H); ^{13}C NMR (150.9MHz, $CDCl_3$) δ 195.2, 166.9, 150.3, 136.5, 132.4, 131.0, 128.8, 126.5, 126.2, 119.4, 67.9, 58.5, 44.0; IR (neat) 2948, 1757, 1725, 1605, 1465, 1426, 1275, 1233, 1212, 1175, 994, 941, 784, 754, 686 cm^{-1} ; MS (ESI, *m/z*) 295.2, 297.1 $[M+H]^+$; HRMS (ESI) Calcd. for $C_{13}H_{11}O_3NaBr$ $[M+Na]^+$: 316.9789, Found: 316.9795.

***iso*-propyl 2-(difluoromethyl)-1-oxo-2,3-dihydro-1H-indene-2-carboxylate (6k).**

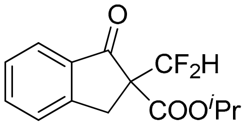

Purified through column chromatography on silica gel (Hexane/EtOAc = 99/1, 98/2) to give **6k, 7k** and **8k.**

Colorless oil, 12.0 mg, 45%. ^{1}H NMR (300 MHz, $CDCl_3$) δ 7.78 (d, *J* = 7.8 Hz, 1H), 7.67 (t, *J* = 7.8 Hz, 1H), 7.56 (d, *J* = 7.8 Hz, 1H), 7.42 (t, *J* = 7.2 Hz, 1H), 6.59 (t, *J* = 55.5 Hz, 1H), 5.07-5.11 (m, 1H), 3.71, 3.53 (ABq, J_{AB} = 17.7 Hz, 2H), 1.23–1.28 (m, 6H); ^{19}F NMR (282 MHz, $CDCl_3$) δ –126.8 (dd, *J* = 287.1, 55.3 Hz, 1F), –129.6 (dd, *J* = 287.1, 55.3 Hz, 1F); ^{13}C NMR (150.9MHz, $CDCl_3$) δ 196.1 (d, *J* = 7.5 Hz), 165.6 (d, *J* = 12.1 Hz), 154.2, 136.2, 134.3 (d, *J* = 4.5 Hz), 128.2, 126.7, 125.4, 115.7 (dd, *J* = 247.5, 241.4 Hz), 70.8, 65.0 (t, *J* = 22.6 Hz), 30.0, 21.7, 21.6; IR (neat) 2984,

1720, 1606, 1467, 1375, 1263, 1213, 1105, 1076, 1040, 914, 759, 676 cm^{-1}; MS (ESI, *m/z*) 291.3 $[M+Na]^+$; HRMS (ESI) Calcd. for $C_{14}H_{14}F_2O_3Na$ $[M+Na]^+$: 291.0809, Found: 291.0838.

iso-Propyl 3-(difluoromethoxy)-1H-indene-2-carboxylate (7k)

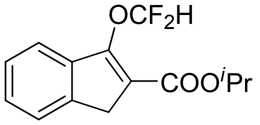

Light yellow solid, 4.0 mg, 15%. ^{1}H NMR (300 MHz, $CDCl_3$) δ 7.61–7.64 (m, 1H), 7.38–7.46(m, 3H, partly overlapping signal), 7.13 (t, *J* = 75.0 Hz, 1H, partly overlapping signal), 5.15–5.23 (m, 1H), 3.72 (s, 2H), 1.36 (s, 3H), 1.34 (s, 3H); ^{19}F NMR (282 MHz, $CDCl_3$) δ –82.0 (d, *J* = 74.2 Hz); ^{13}C NMR (150.9MHz, $CDCl_3$) δ 163.3, 155.7, 141.7, 138.9, 129.2, 127.4, 124.5, 121.1, 118.4, 116.9 (t, *J* = 261.1 Hz), 68.6, 36.3, 22.1; IR (KBr) 2984, 1694, 1618, 1602, 1396, 1355, 1317, 1260, 1209, 1142, 1128, 1112, 1083, 1045, 953, 760 cm^{-1}; m.p.: 41–43 ℃; MS (ESI, *m/z*) 291.2 $[M+Na]^+$; HRMS (ESI) Calcd. for $C_{14}H_{14}F_2O_3Na$ $[M+Na]^+$: 291.0809, Found: 291.0811.

iso-Propyl 2-bromo-1-oxo-2,3-dihydro-1H-indene-2-carboxylate (8k)

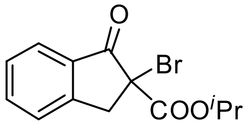

White solid, 25.0 mg, 84%. ^{1}H NMR (300 MHz, $CDCl_3$) δ 7.87 (d, *J* = 7.8 Hz 1H), 7.67–7.72 (m, 1H), 7.44–7.49 (m, 2H) 5.06–5.14 (m, 1H), 4.18 (d, *J* = 18.0 Hz, 1H), 3.67 (d, *J* = 18.0 Hz, 1H), 1.26 (s, 3H), 1.28 (s, 3H); ^{13}C NMR (150.9MHz, $CDCl_3$) δ 195.4, 166.6, 150.4, 136.4, 132.5, 128.7, 126.5, 126.1, 71.8, 59.0, 44.0, 21.6, 21.5; IR (KBr) 2983, 1748, 1720, 1603, 1462, 1378, 1276, 1251, 1214, 1182, 1105, 1078, 996, 830, 748, 694, 638 cm^{-1}; m.p.: 76–78 ℃; MS (ESI, *m/z*) 319.2, 321.2 $[M+Na]^+$; HRMS (ESI) Calcd. for $C_{13}H_{13}O_3NaBr$ $[M+Na]^+$: 318.9946, Found: 318.9961.

Benzyl 5-bromo-2-(difluoromethyl)-1-oxo-2,3-dihydro-1H-indene-2-carboxylate (6l)

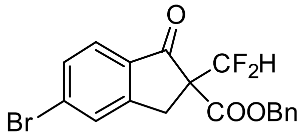

Purified through column chromatography on silica gel (Hexane/EtOAc = 99/1, 98/2) to give **6l, 7l** and **8l.**

Yellow solid, 20.0 mg, 51%. ^{1}H NMR (300 MHz, $CDCl_3$) δ 7.74 (s, 1H), 7.55–7.64 (m, 2H), 7.27–7.37 (m, 5H), 6.60 (t, *J* = 55.0 Hz, 1H), 5.22 (d, *J* = 2.4 Hz, 2H), 3.72, 3.54 (ABq, J_{AB} = 18.0 Hz, 2H); ^{19}F NMR (282 MHz, $CDCl_3$) δ –126.3 (dd, *J* = 287.9, 54.4 Hz, 1F), –129.4 (dd, *J* = 287.9,

54.4 Hz, 1F); ^{13}C NMR (150.9MHz, $CDCl_3$) δ 194.6 (d, *J* = 6.0 Hz), 165.6 (d, *J* = 12.1 Hz), 155.4, 134.8, 133.0 (d, *J* = 3.0 Hz), 132.1, 132.0, 130.1, 128.9, 128.8, 128.1, 126.6, 115.4 (dd, *J* = 247.5, 241.4 Hz), 68.4, 65.0 (dd, *J* = 24.1, 21.1 Hz), 29.7; IR (KBr) 3036, 1596, 1577, 1418, 1374, 1352, 1299, 1213, 1179, 1158, 1104, 1078, 1061, 1048, 948, 910, 872, 840, 830, 750, 697, 688, 591 cm^{-1}; m.p.: 84–86 ℃; MS (ESI, *m/z*) 417.5, 419.3 $[M+Na]^+$; HRMS (ESI) Calcd. for $C_{18}H_{13}F_2O_3NaBr$ $[M+Na]^+$: 416.9914, Found: 416.9919.

Benzyl 6-bromo-3-(difluoromethoxy)-1H-indene-2-carboxylate (7l)

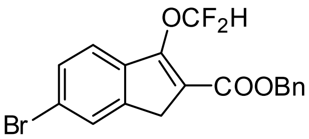

Yellow solid, 9.0 mg, 23%. ^{1}H NMR (300 MHz, $CDCl_3$) δ 7.61 (s, 1H), 7.36–7.56 (m, 7H, partly overlapping signal), 7.16 (t, *J* = 75.0 Hz, 1H, partly overlapping signal), 5.30 (s, 2H), 3.73 (s, 2H); ^{19}F NMR (282 MHz, $CDCl_3$) δ –82.5 (d, *J* = 75.3 Hz); ^{13}C NMR (150.9MHz, $CDCl_3$) δ 163.2, 155.6, 143.4, 137.7, 135.7, 130.8, 128.9, 128.7, 128.5, 127.9, 124.1, 122.5, 117.3, 116.5 (t, *J* = 262.6 Hz), 66.8, 36.1; IR (KBr) 3035, 2923, 1709, 1618, 1598, 1572, 1460, 1396, 1380, 1356, 1321, 1254, 1209, 1186, 1163, 1135, 1107, 1064, 966, 868, 847, 827, 802, 766, 740, 727 cm^{-1}; m.p.: 75–77 ℃; MS (ESI, *m/z*) 417.3, 419.5 $[M+Na]^+$; HRMS (ESI) Calcd. for $C_{18}H_{13}F_2O_3NaBr$ $[M+Na]^+$: 416.9914, Found: 416.9926.

Benzyl 2,5-dibromo-1-oxo-2,3-dihydro-1H-indene-2-carboxylate (8l)

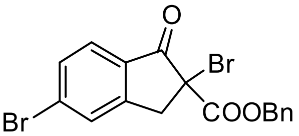

White solid, 38.0 mg, 90%. ^{1}H NMR (300 MHz, $CDCl_3$) δ 7.59–7.73 (m, 3H), 7.29–7.39 (m, 5H), 5.25 (dd, *J* = 13.8, 12.3 Hz, 2H), 4.16 (d, *J* = 18.3 Hz, 1H), 3.64 (d, *J* = 18.3 Hz, 1H); ^{13}C NMR (150.9MHz, $CDCl_3$) δ 193.9, 166.6, 151.7, 134.8, 132.5, 132.1, 131.2, 129.8, 128.8, 128.7, 128.2, 127.2, 69.1, 58.1, 43.5; IR (KBr) 3066, 2950, 1760, 1725, 1595, 1579, 1455, 1420, 1314, 1263, 1229, 1207, 1168, 1076, 1056, 1031, 887, 839, 771, 751, 701, 645 cm^{-1}; m.p.: 76–78 ℃; MS (ESI, *m/z*) 447.0, 449.2 $[M+Na]^+$; HRMS (ESI) Calcd. for $C_{17}H_{12}O_3NaBr_2$ $[M+Na]^+$: 444.9051, Found: 444.9045.

Methyl 2-(difluoromethyl)-1-oxo-1,2,3,4-tetrahydronaphthalene-2-carboxylate (6m)

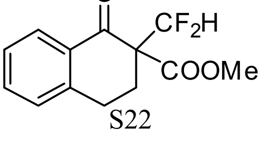

Purified through column chromatography on silica gel (Hexane/EtOAc = 99/1, 98/2) to give **6m** and **8m**, and **7m** was not separated due to its low yield.

Colorless oil, 8.0 mg, 32%. ^{1}H NMR (300 MHz, $CDCl_3$) δ 8.05 (d, *J* = 7.8 Hz, 1H), 7.50–7.56 (m, 1H), 7.34 (d, *J* = 7.8 Hz, 1H), 7.26 (d, *J* = 7.5 Hz, 1H), 6.60 (t, *J* = 55.2 Hz, 1H), 3.75 (s, 3H), 3.27–3.38 (m, 1H), 2.99-3.08 (m, 1H), 2.63–2.71 (m, 1H), 2.43–2.52 (m, 1H); ^{19}F NMR (282 MHz, $CDCl_3$) δ –127.8 (dd, *J* = 283.1, 54.4 Hz, 1F), –132.3 (dd, *J* = 283.1, 54.4 Hz, 1F); ^{13}C NMR (150.9MHz, $CDCl_3$) δ 190.2 (d, *J* = 6.0 Hz), 167.0 (d, *J* = 9.0 Hz), 143.8, 134.8, 131.2 (d, *J* = 3.0 Hz), 129.1, 128.4, 127.2, 116.0 (t, *J* = 246.0 Hz), 61.0 (t, *J* = 21.1 Hz), 53.5, 25.2, 23.3; IR (neat) 2956, 1743, 1686, 1601, 1455, 1305, 1227, 1057, 750 cm^{-1}; MS (ESI, *m/z*) 277.2 $[M+Na]^+$; HRMS (ESI) Calcd. for $C_{13}H_{12}F_2O_3Na$ $[M+Na]^+$: 277.0652, Found: 277.0648.

Methyl 2-bromo-1-oxo-1,2,3,4-tetrahydronaphthalene-2-carboxylate (8m Known)[3b]

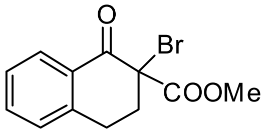

White solid, 21.0 mg, 74%. ^{1}H NMR (300 MHz, $CDCl_3$) δ 8.1 (dd, *J* = 7.8, 0.9 Hz, 1H), 7.54 (td, *J* = 7.5, 1.2 Hz, 1H), 7.37 (t, *J* = 7.8 Hz, 1H), 7.28 (d, *J* = 6.9 Hz, 1H), 3.86 (s, 3H), 3.18–3.29 (m, 1H), 2.94–3.08 (m, 2H), 2.54–2.62 (m, 1H).

Benzyl 2-(difluoromethyl)-1-oxo-1,2,3,4-tetrahydronaphthalene-2-carboxylate (6n)

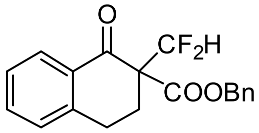

Purified through column chromatography on silica gel (Hexane/EtOAc = 99/1, 98/2) to give **6n** and **8n**, and **7n** was not separated due to its low yield.

Colorless oil, 9.0 mg, 27%. ^{1}H NMR (300 MHz, $CDCl_3$) δ 8.04 (d, *J* = 8.1 Hz, 1H), 7.52 (t, *J* = 7.2 Hz, 1H), 7.19–7.36 (m, 7H), 6.61 (t, *J* = 55.2 Hz, 1H), 5.18 (dd, *J* = 15.0, 12.6 Hz, 2H), 3.20–3.31 (m, 1H), 2.96–3.05 (m, 1H), 2.63–2.71 (m, 1H), 2.41-2.51 (m, 1H); ^{19}F NMR (282 MHz, $CDCl_3$) δ –126.8 (dd, *J* = 284.0, 55.3 Hz, 1F), –131.0 (dd, *J* = 284.0, 55.3 Hz, 1F); ^{13}C NMR (150.9MHz, $CDCl_3$) δ 190.2 (d, *J* = 6.0 Hz), 166.4 (d, *J* = 9.0 Hz), 143.6, 135.0, 134.7, 131.3, 129.1, 128.8, 128.6, 128.4, 127.9, 127.2, 115.9 (t, *J* = 246.0 Hz), 68.0, 61.0 (t, *J* = 21.1 Hz), 53.5, 25.1, 23.4; IR (neat) 2926, 1742, 1686, 1601, 1455, 1355, 1303, 1224, 1162, 1116, 1058, 907, 796, 735, 697 cm^{-1}; MS (ESI, *m/z*) 353.5, $[M+Na]^+$; HRMS (ESI) Calcd. for $C_{19}H_{16}F_2O_3Na$ $[M+Na]^+$: 353.0965, Found: 353.0973.

Benzyl 2-bromo-1-oxo-1,2,3,4-tetrahydronaphthalene-2-carboxylate (8n)

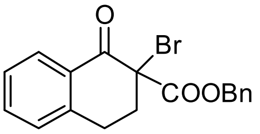

Sticky yellow oil, 26.0 mg, 72%. ^{1}H NMR (300 MHz, $CDCl_3$) δ 8.10 (d, *J* = 7.8 Hz, 1H), 7.53 (td, *J* = 7.8, 1.2 Hz, 1H), 7.23–7.39 (m, 7H), 5.28 (dd, *J* = 22.8, 12.3 Hz, 2H), 3.16–3.25 (m, 1H), 2.93–3.02 (m, 2H), 2.54–2.62 (m, 1H); ^{13}C NMR (150.9MHz, $CDCl_3$) δ 187.6, 167.4, 142.5, 135.1, 134.5, 129.7, 129.2, 128.9, 128.8, 128.6, 128.2, 127.5, 68.8, 65.2, 35.8, 26.9; IR (neat) 3032, 2950, 1758, 1685, 1600, 1455, 1296, 1214, 1180, 1123, 1031, 994, 948, 887, 833, 736, 697, 573 cm^{-1}; MS (ESI, *m/z*) 381.4, 383.3 $[M+Na]^+$; HRMS (ESI) Calcd. for $C_{18}H_{15}O_3NaBr$ $[M+Na]^+$: 381.0102, Found: 381.0105.

Benzyl 1-(difluoromethyl)-2-oxocyclopentanecarboxylate (6o)

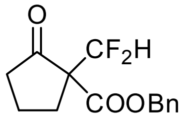

Purified through column chromatography on silica gel (Hexane/EtOAc = 98/2) to give **6o** and **7o**, and **8o** was not separated as a pure product due to its unstability probably.

Colorless oil, 9.0 mg, 34%. ^{1}H NMR (300 MHz, $CDCl_3$) δ 7.30–7.38 (m, 5H), 6.38 (t, *J* = 55.2 Hz, 1H), 5.20 (s, 2H), 2.29–2.62 (m, 4H), 2.07–2.16 (m, 2H); ^{19}F NMR (282 MHz, $CDCl_3$) δ –127.0 (dd, *J* = 287.9, 55.3 Hz, 1F), –128.5 (dd, *J* = 287.9, 55.3 Hz, 1F); ^{13}C NMR (150.9MHz, $CDCl_3$) δ 208.6 (d, *J* = 6.0 Hz), 166.3 (d, *J* = 12.1 Hz), 135.0, 128.9, 128.8, 128.1, 115.6 (dd, *J* = 247.5, 239.9 Hz), 68.1, 64.3 (t, *J* = 21.1 Hz), 38.7 (d, *J* = 3.0 Hz), 26.1, 20.0; IR (neat) 2963, 1761, 1734, 1498, 1455, 1375, 1264, 1236, 1165, 1107, 1067, 1002, 960, 743, 697 cm^{-1}; MS (ESI, *m/z*) 291.2 $[M+Na]^+$; HRMS (ESI) Calcd. for $C_{14}H_{14}F_2O_3Na$ $[M+Na]^+$: 291.0809, Found: 291.0812.

Benzyl 2-(difluoromethoxy)cyclopent-1-enecarboxylate (7o)

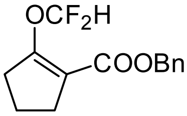

Colorless oil, 3.5 mg, 13%. ^{1}H NMR (300 MHz, $CDCl_3$) δ 7.31–7.38 (m, 5H), 6.60 (t, *J* = 73.5 Hz, 1H), 5.20 (s, 2H), 2.61–2.72 (m, 4H), 1.89-2.00 (m, 2H); ^{19}F NMR (282 MHz, $CDCl_3$) δ –82.1 (d, *J* = 73.3 Hz); ^{13}C NMR (150.9MHz, $CDCl_3$) δ 163.8, 158.7, 136.4, 128.7, 128.2, 128.0, 115.1 (t, *J* = 264.1 Hz), 113.6, 66.0, 33.1, 29.3, 19.5; IR (neat) 2956, 1723, 1658, 1498, 1456, 1385, 1353, 1266, 1217, 1139, 1096, 908, 747, 698 cm^{-1}; MS (ESI, *m/z*) 291.2 $[M+Na]^+$; HRMS (ESI) Calcd. for $C_{14}H_{14}F_2O_3Na$ $[M+Na]^+$: 291.0809, Found: 291.0818.

Ethyl 2-benzoyl-3,3-difluoro-2-methylpropanoate (6p)

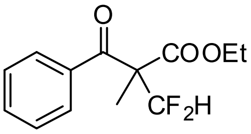

Purified through column chromatography on silica gel (Hexane/EtOAc = 99/1, 98/2) to give **6p** and **7p**, and **8p** was not separated as a pure product due to its unstability probably.

Colorless oil, 7.5 mg, 29%. ^{1}H NMR (300 MHz, $CDCl_3$) δ 7.84–7.86 (m, 2H), 7.55–7.60 (m, 1H), 7.42–7.48 (m, 2H), 6.52 (t, J = 55.0 Hz, 1H), 4.18–4.28 (m, 2H), 1.70 (d, J = 1.5 Hz, 3H), 1.15 (t, J = 6.9 Hz, 3H); ^{19}F NMR (282 MHz, $CDCl_3$) δ –127.2 (dd, J = 282.0, 54.4, Hz, 1F), –130.6 (dd, J = 282.0, 54.4, Hz, 1F); ^{13}C NMR (150.9MHz, $CDCl_3$) δ 193.6 (d, J = 6.0 Hz), 168.5 (d, J = 7.5 Hz), 134.8 (d, J = 3.0 Hz), 133.7, 128.9, 128.8, 115.5 (dd, J = 250.5, 242.5 Hz), 62.8, 62.0 (t, J = 21.1 Hz), 14.1 (q, J = 3.0 Hz), 13.9; IR (neat) 2985, 1749, 1686, 1598, 1449, 1367, 1266, 1233, 1186, 1089, 1066, 978, 798, 697, 651 cm^{-1}; MS (ESI, *m/z*) 279.2 $[M+Na]^+$; HRMS (ESI) Calcd. for $C_{13}H_{14}F_2O_3Na$ $[M+Na]^+$: 279.0809, Found: 279.0817.

(Z)-Ethyl 3-(difluoromethoxy)-2-methyl-3-phenylacrylate (7p)

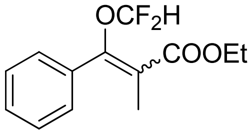

Yellow solid, 3.0 mg, 12%. ^{1}H NMR (300 MHz, $CDCl_3$) δ 7.34–7.42 (m, 5H), 6.06 (t, J = 75.0 Hz, 1H), 3.94 (q, J = 7.2 Hz, 2H), 2.09 (s, 3H), 0.91 (t, J = 7.2 Hz, 3H); ^{19}F NMR (282 MHz, $CDCl_3$) δ –83.7 (d, J = 74.2 Hz); ^{13}C NMR (150.9MHz, $CDCl_3$) δ 168.4, 152.9, 133.0, 129.8, 128.9, 128.5, 118.2, 115.4 (t, J = 258.0 Hz), 60.6, 13.5; IR (KBr) 2984, 2929, 1699, 1654, 1445, 1369, 1312, 1150, 1138, 1089, 1056, 930, 775, 704 cm^{-1}; m.p.: 40–43 ℃; MS (ESI, *m/z*) 279.3 $[M+Na]^+$; HRMS (ESI) Calcd. for $C_{13}H_{14}F_2O_3Na$ $[M+Na]^+$: 279.0809, Found: 279.0811.

References

1. J. J. Sepiol, M. Gora, M. K. Luczynskib, Synlett., 2011, 1383–1386.
2. A. M. R. Smith, D. Billen, K. K. Hii, Chem. Commun., 2009, 3925.
3. a) Y. Nomura, E. Tokunaga, N. Shibata, Angew. Chem. Int. Ed., 2011,50, 1885-1889. b) H. M. Meshram, P. N. Reddy, K. Sadashiv and J. S. Yadav, *Tetrahedron Lett.*, **2005**, *46*, 623–626; c) R. Akula, M. J. Galligan, H. Ibrahim, *Synthesis,* **2011**, 347–351; d) D. A. Nicewicz and D. W. C. MacMillan, *Science (Washington, DC. US),* **2008**, *322*, 77–80.

^{1}H NMR ($CDCl_3$, 300 MHz)

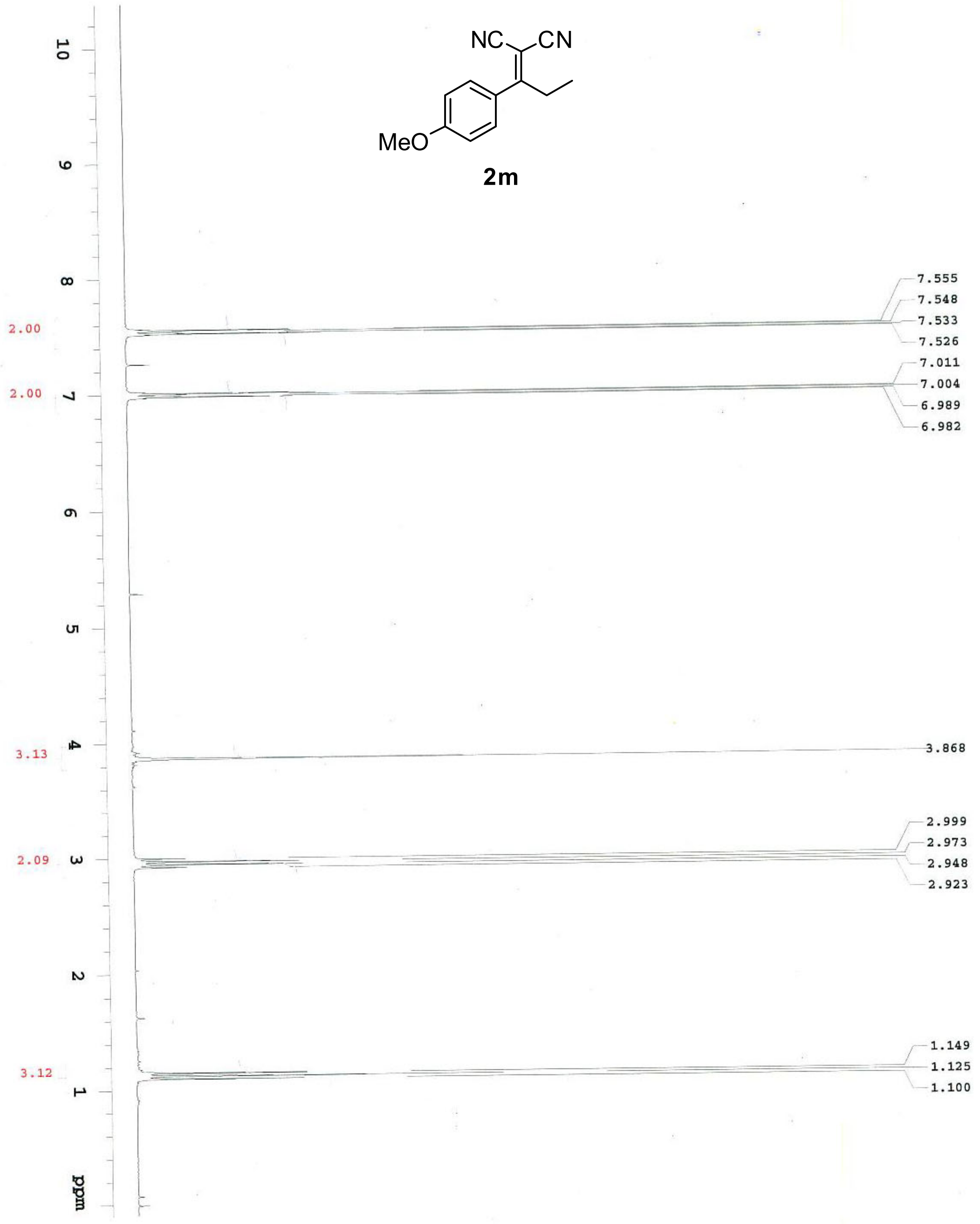

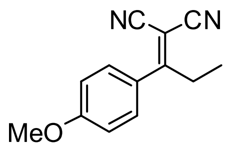

2m

^{13}C NMR ($CDCl_3$, 150.9 MHz)

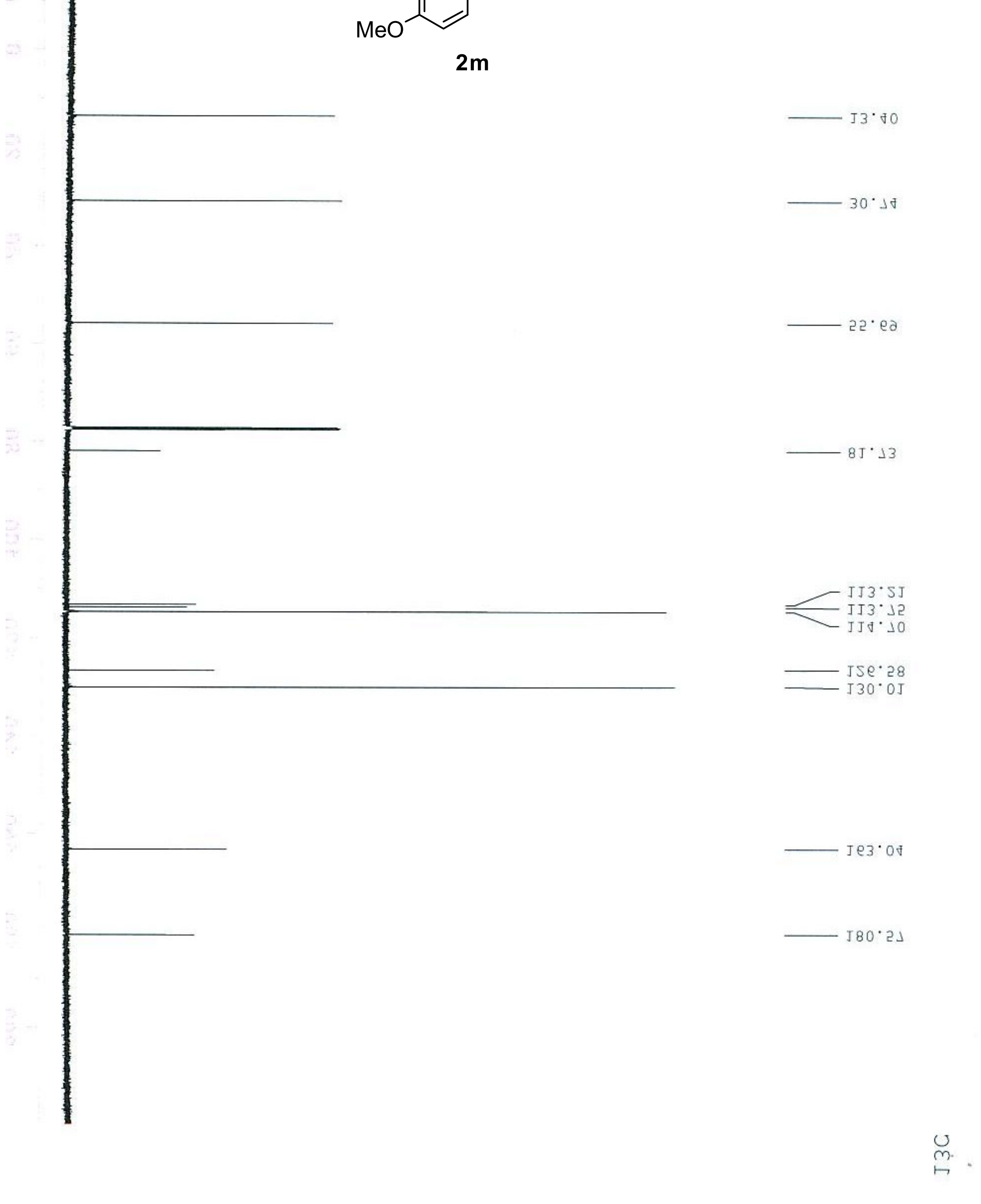

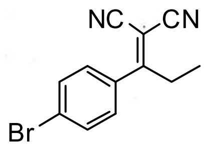

2n

^{1}H NMR ($CDCl_3$, 300 MHz)

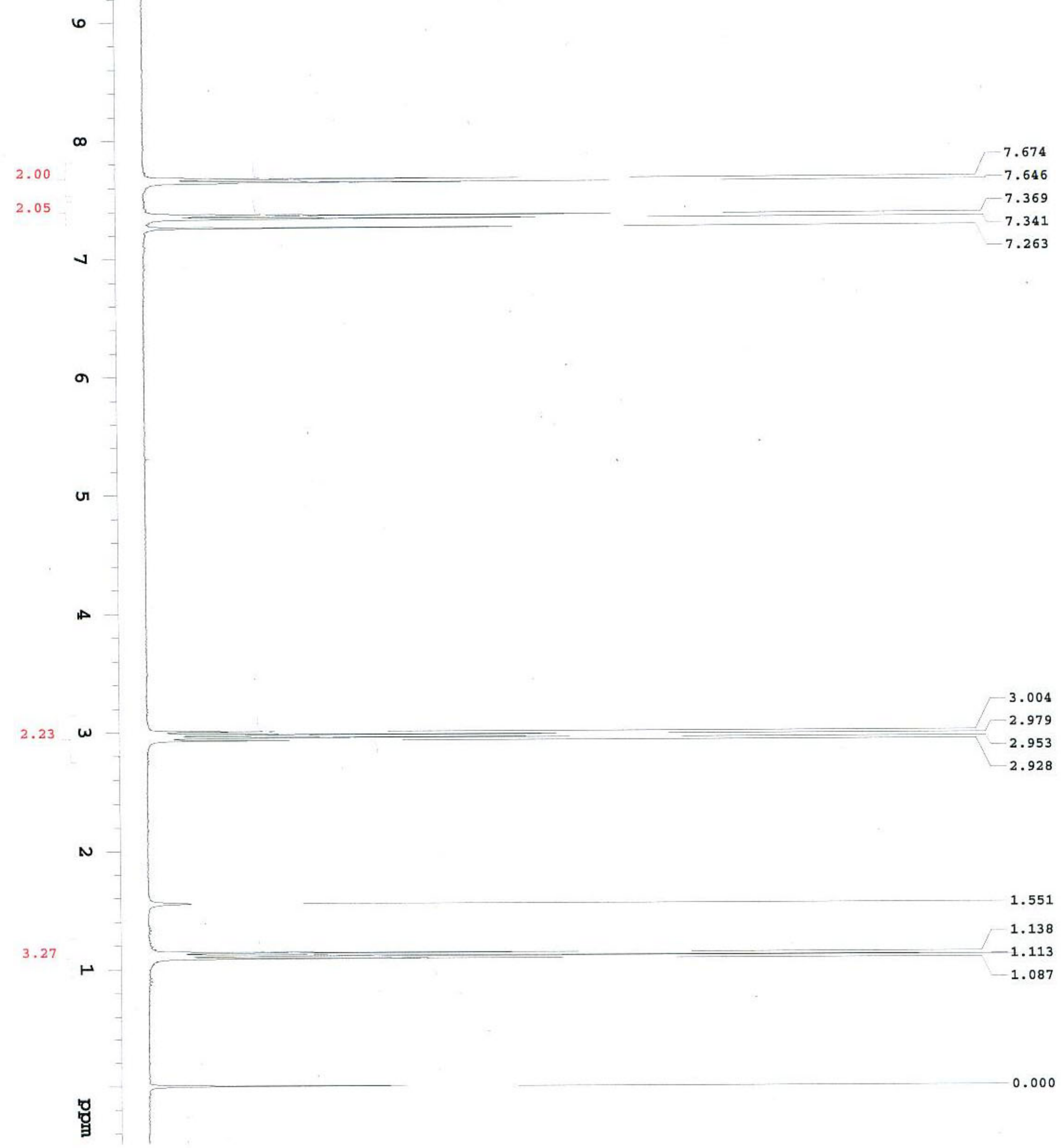

^{13}C NMR ($CDCl_3$, 150.9 MHz)

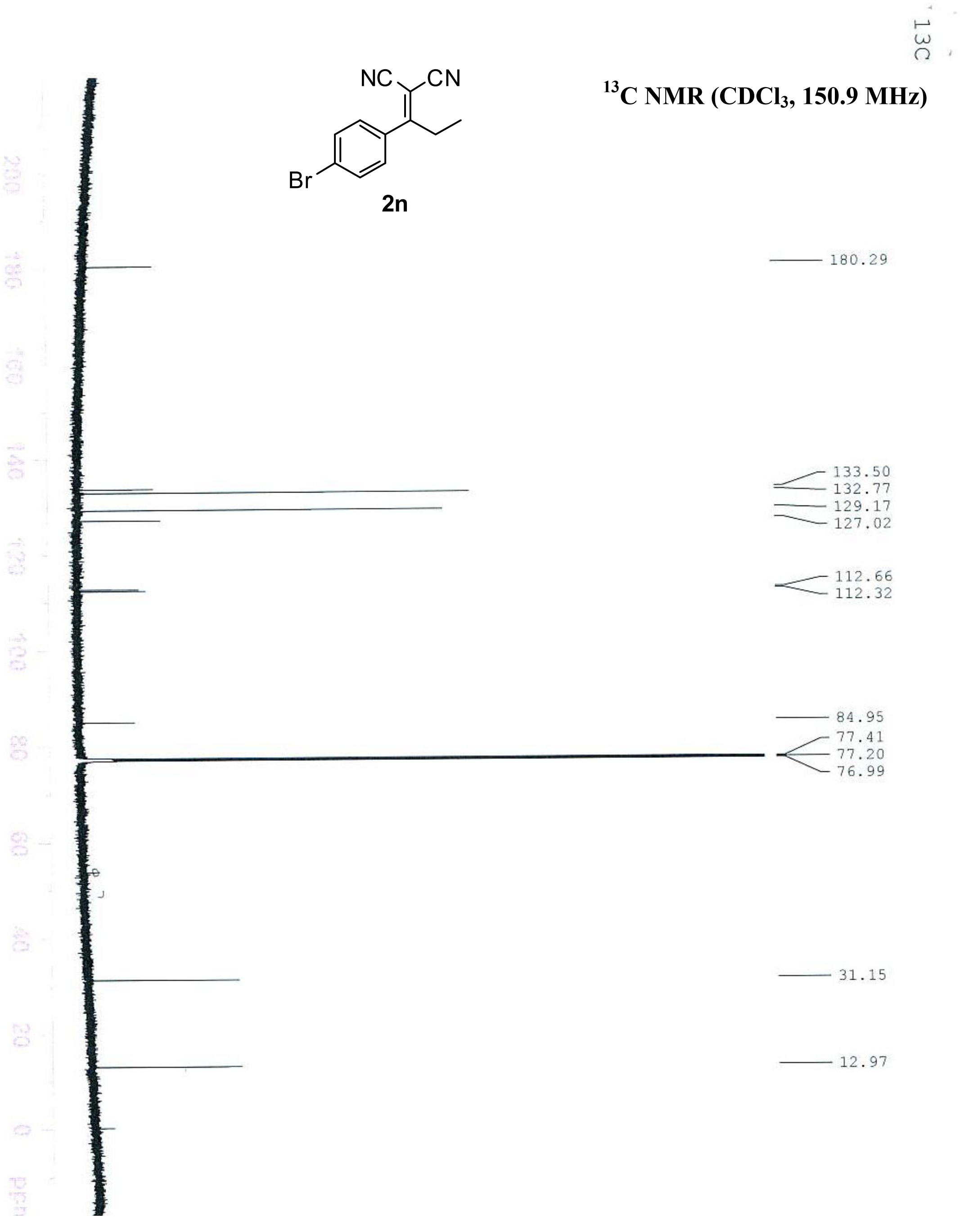

^{1}H NMR ($CDCl_3$, 300 MHz)

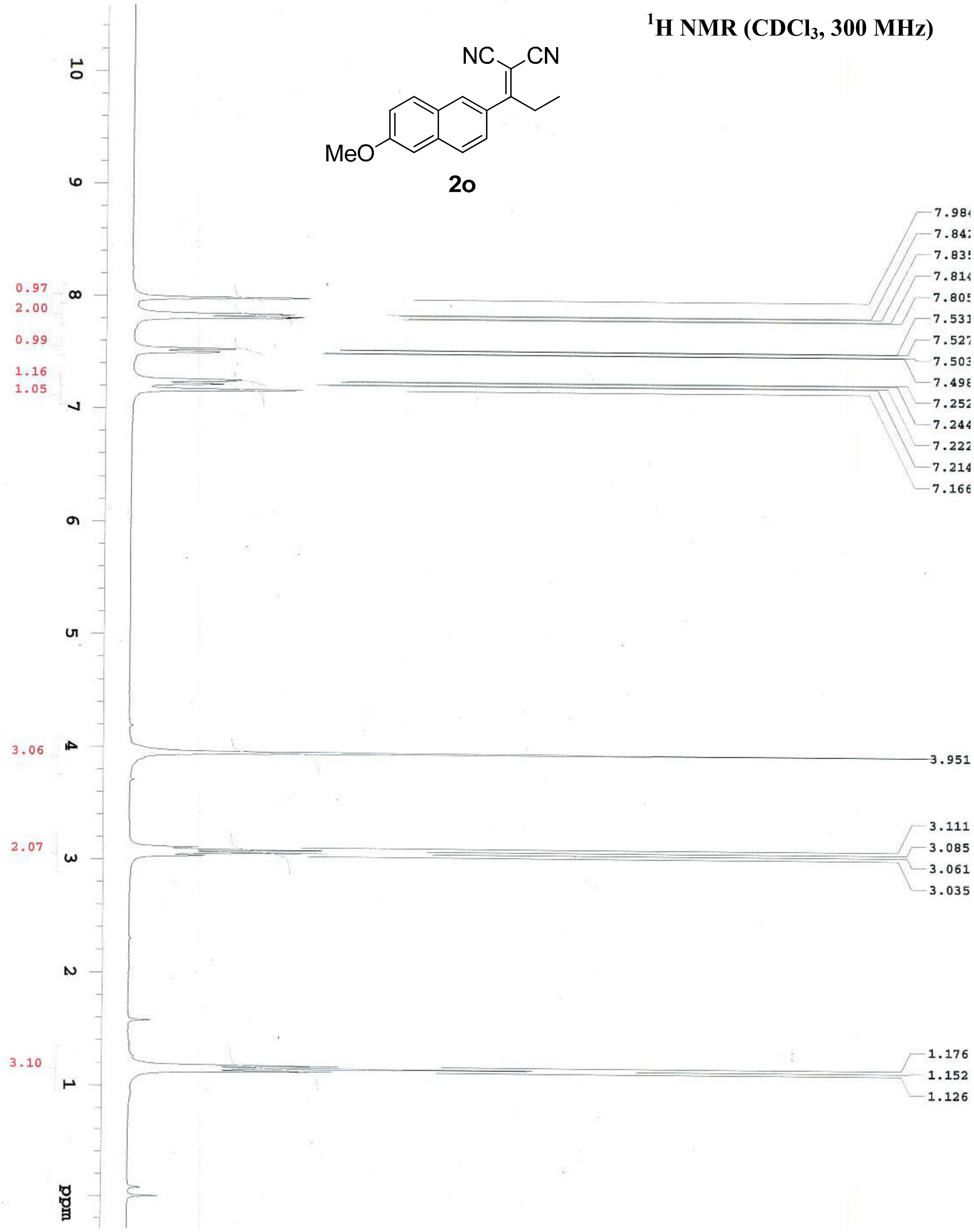

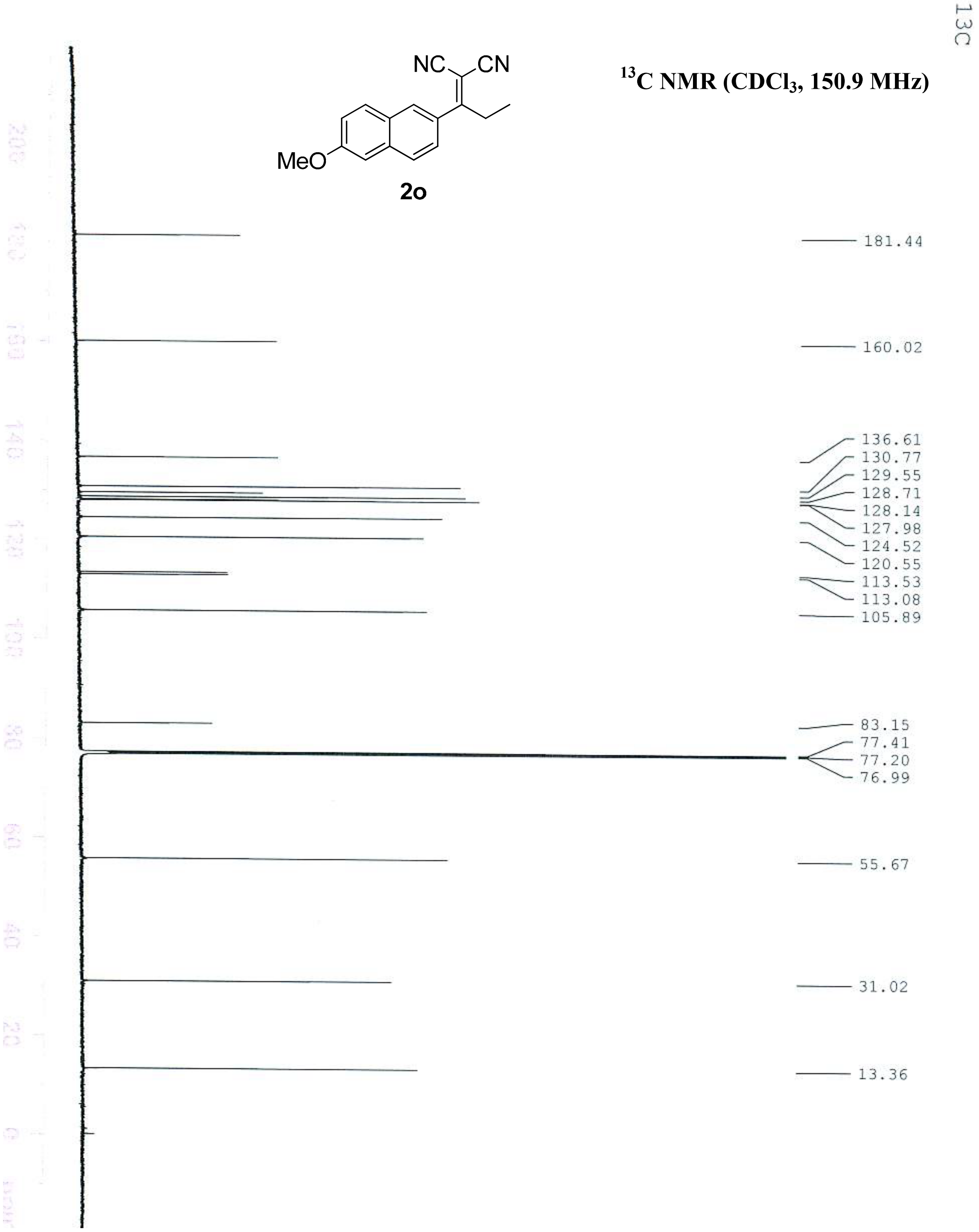

^{13}C NMR ($CDCl_3$, 150.9 MHz)

^{1}H NMR ($CDCl_3$, 300 MHz)

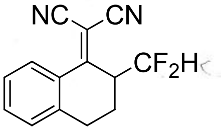

3a

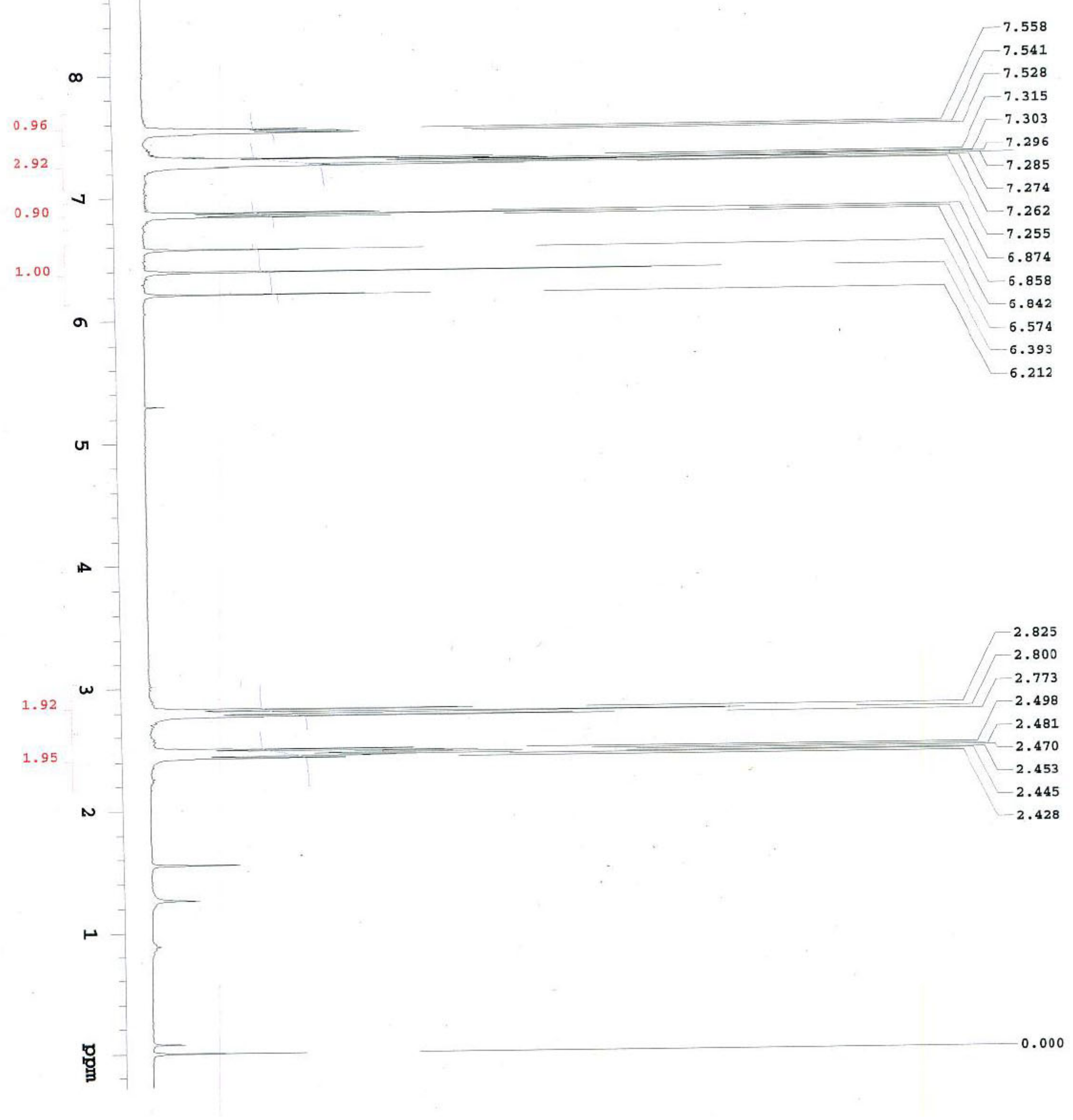

^{19}F NMR (CDCl$_3$, 282 MHz)

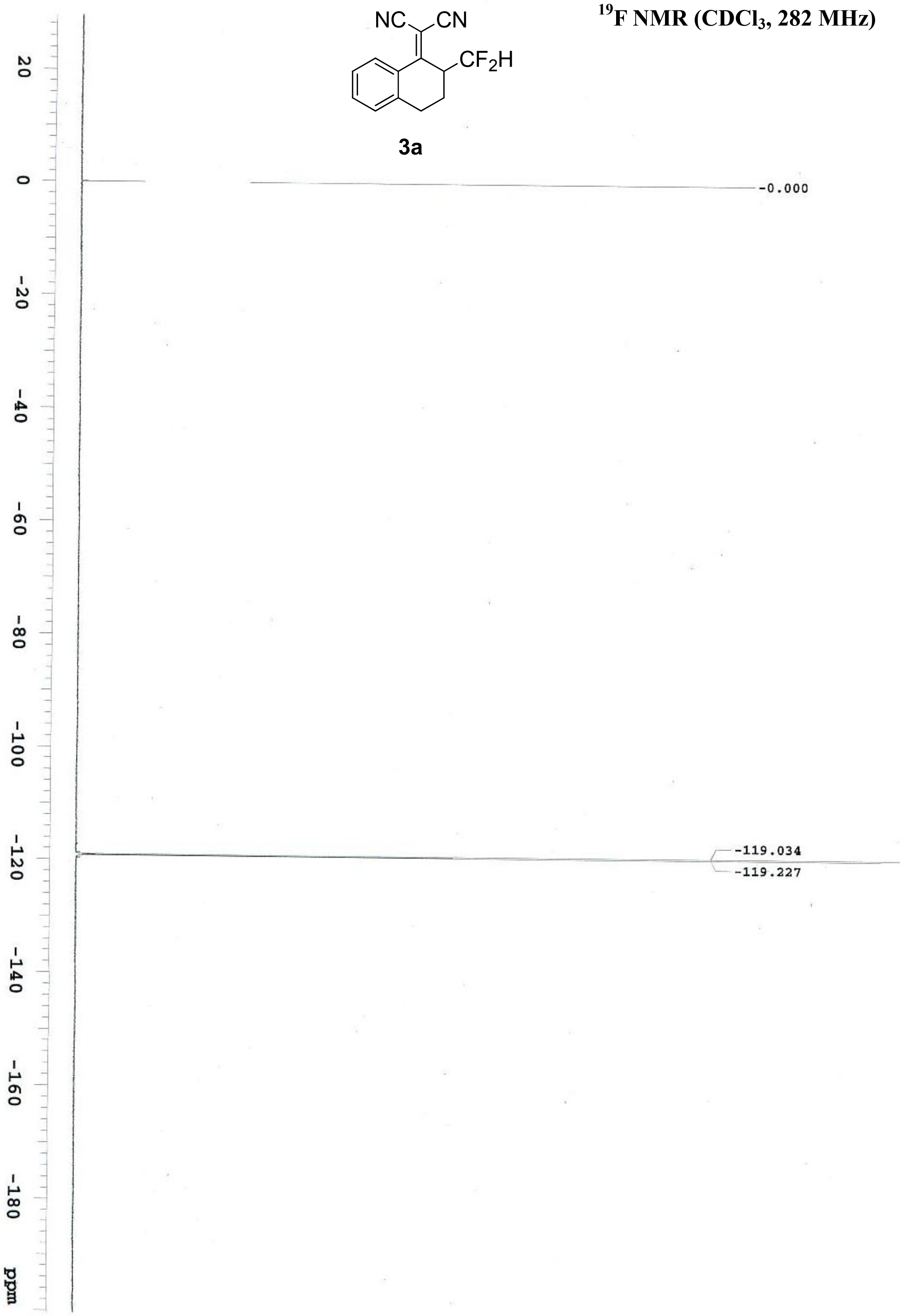

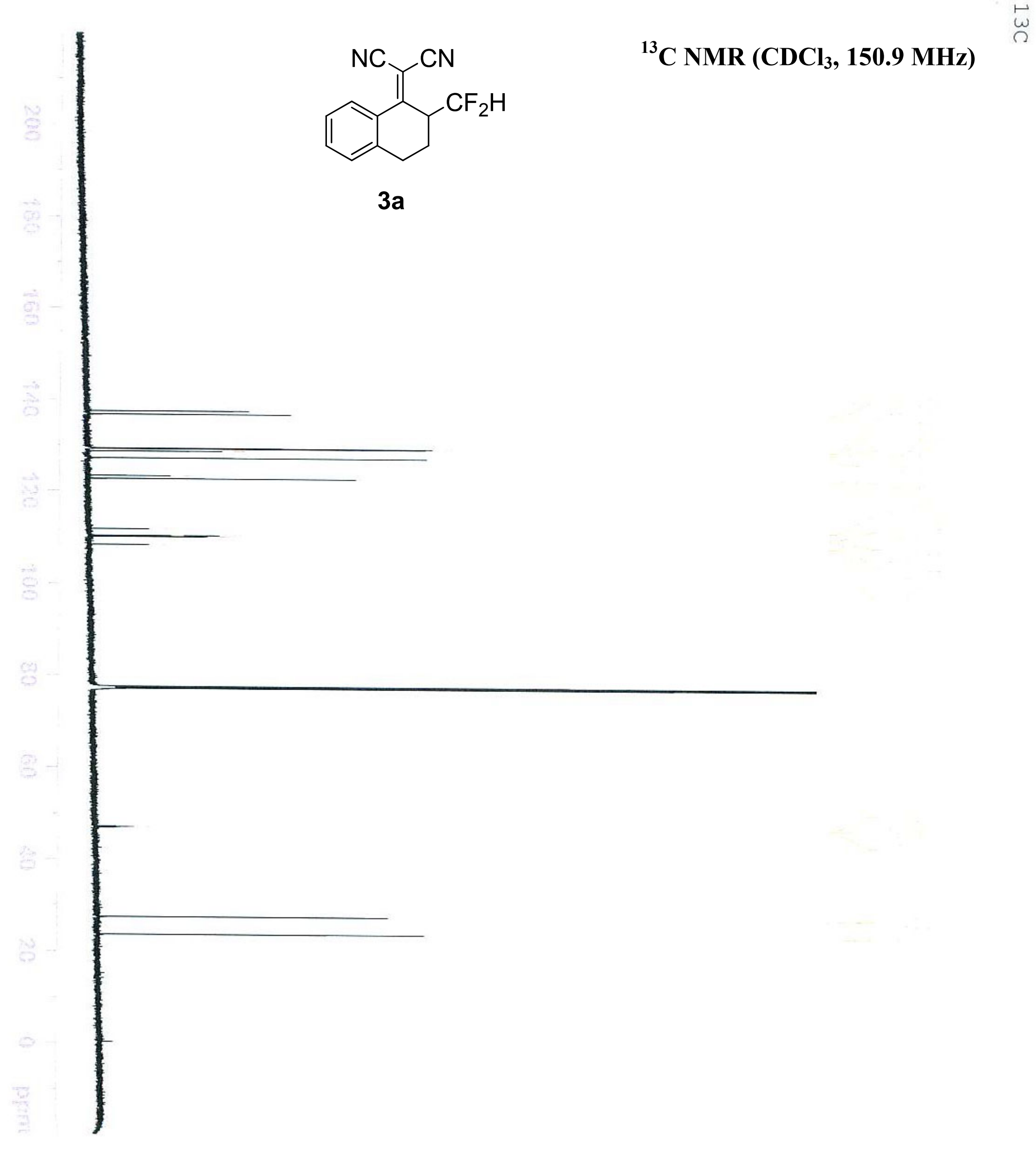

3a

^{13}C NMR ($CDCl_3$, 150.9 MHz)

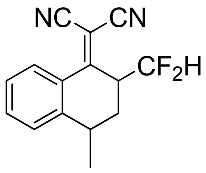

3b

^{1}H NMR ($CDCl_3$, 300 MHz)

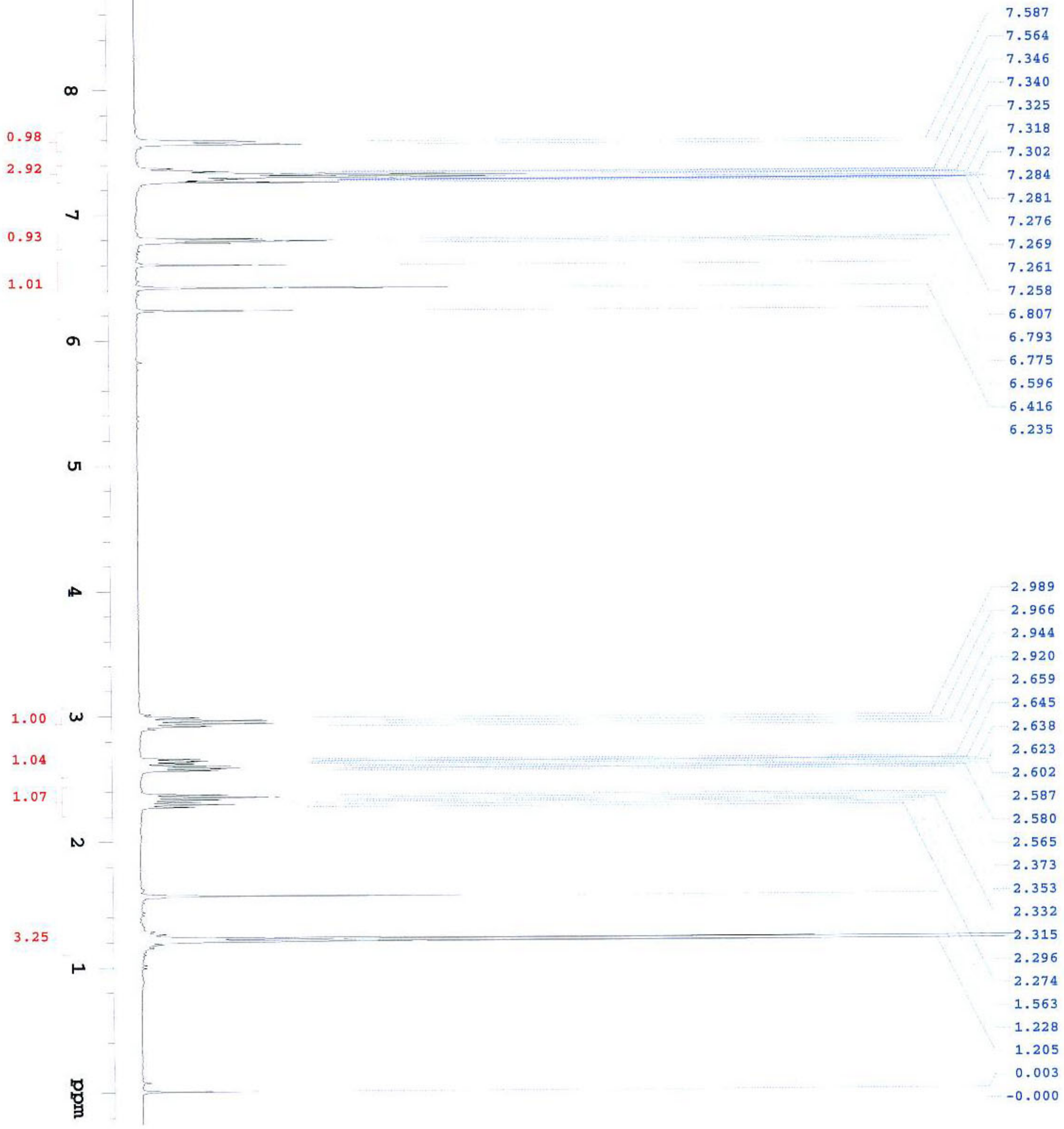

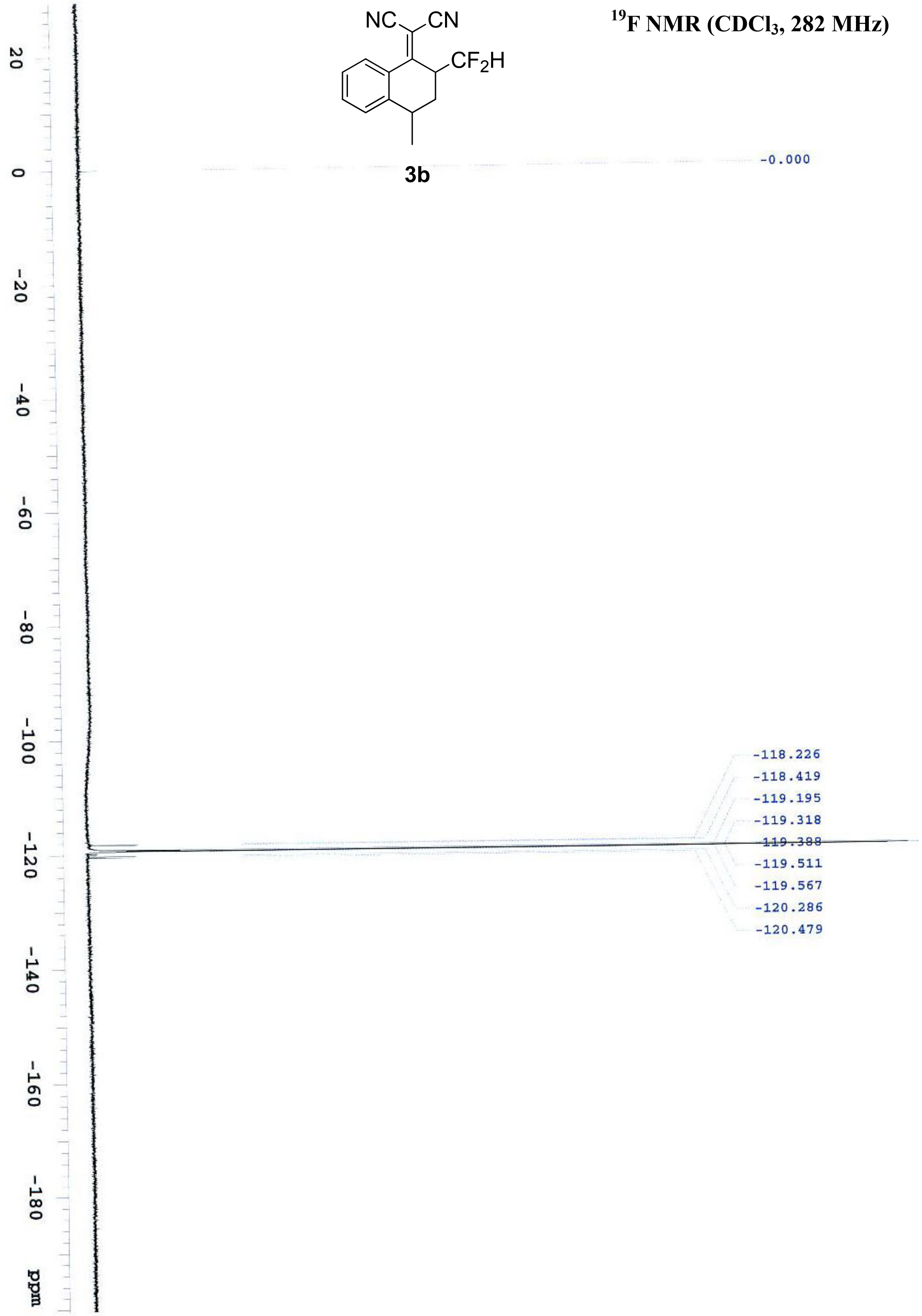

3b

^{19}F NMR ($CDCl_3$, 282 MHz)

^{13}C NMR ($CDCl_3$, 150.9 MHz)

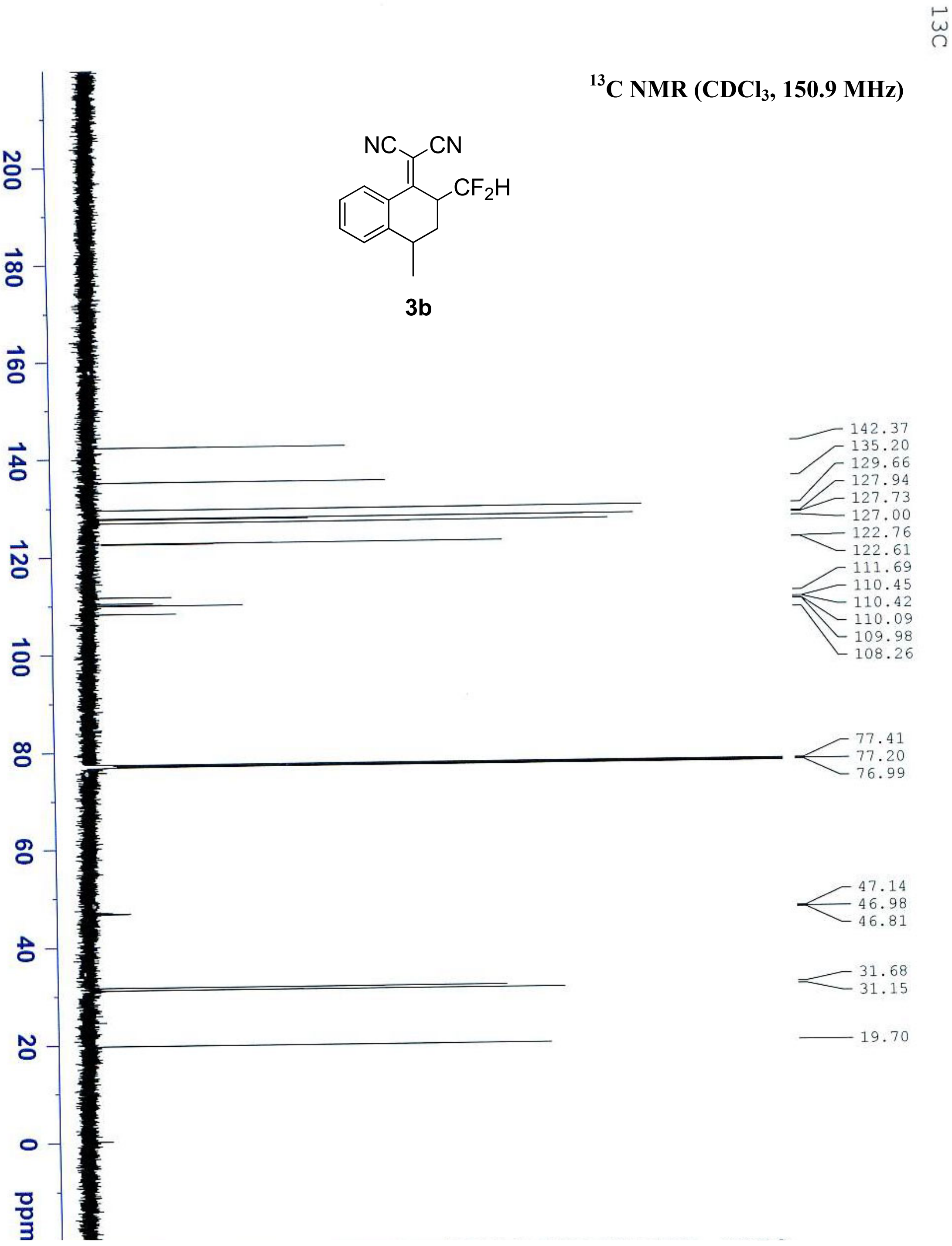

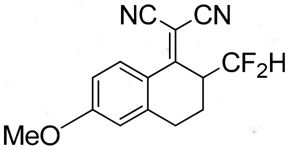

3c

^{1}H NMR ($CDCl_3$, 300 MHz)

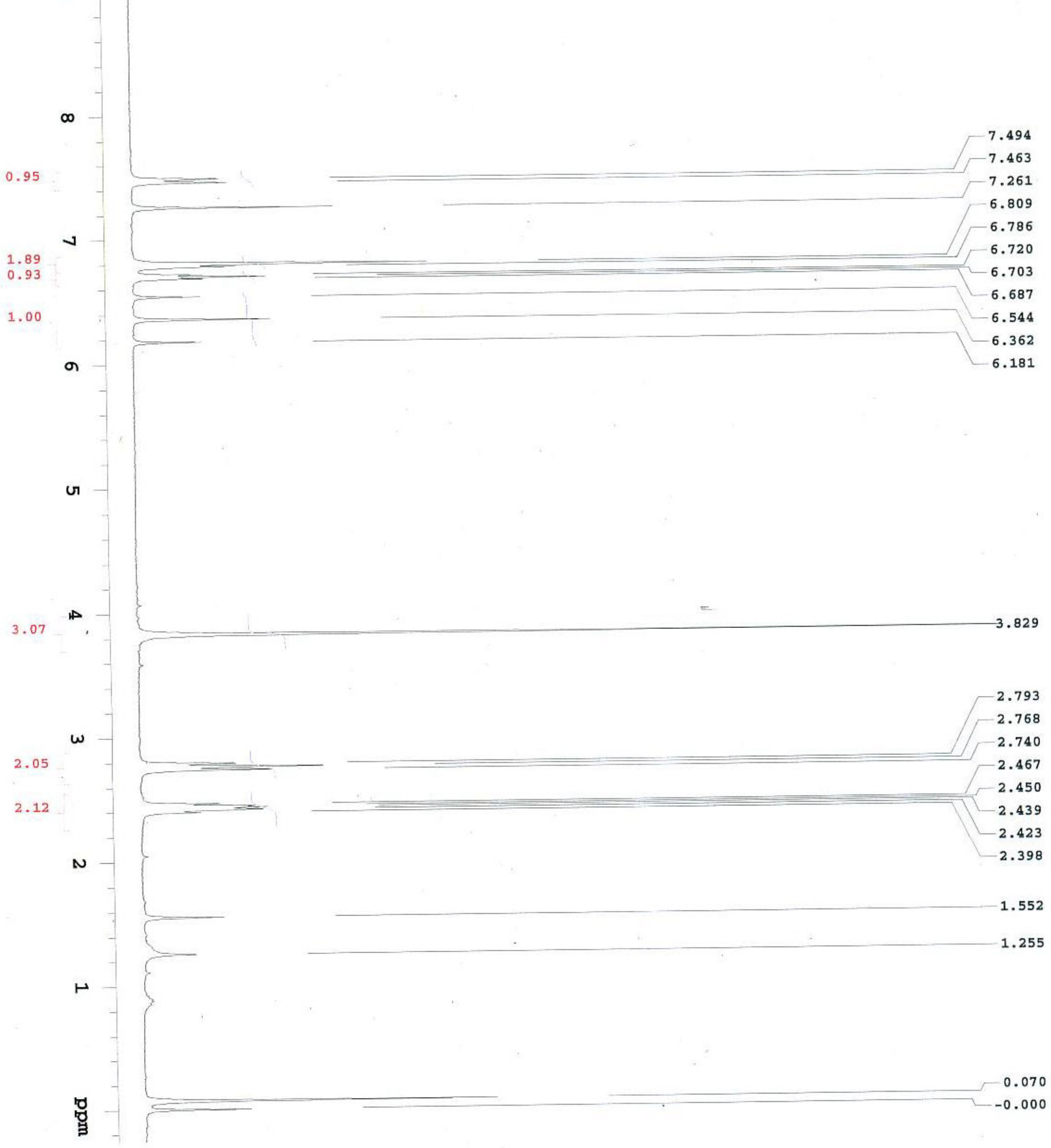

^{19}F NMR ($CDCl_3$, 282 MHz)

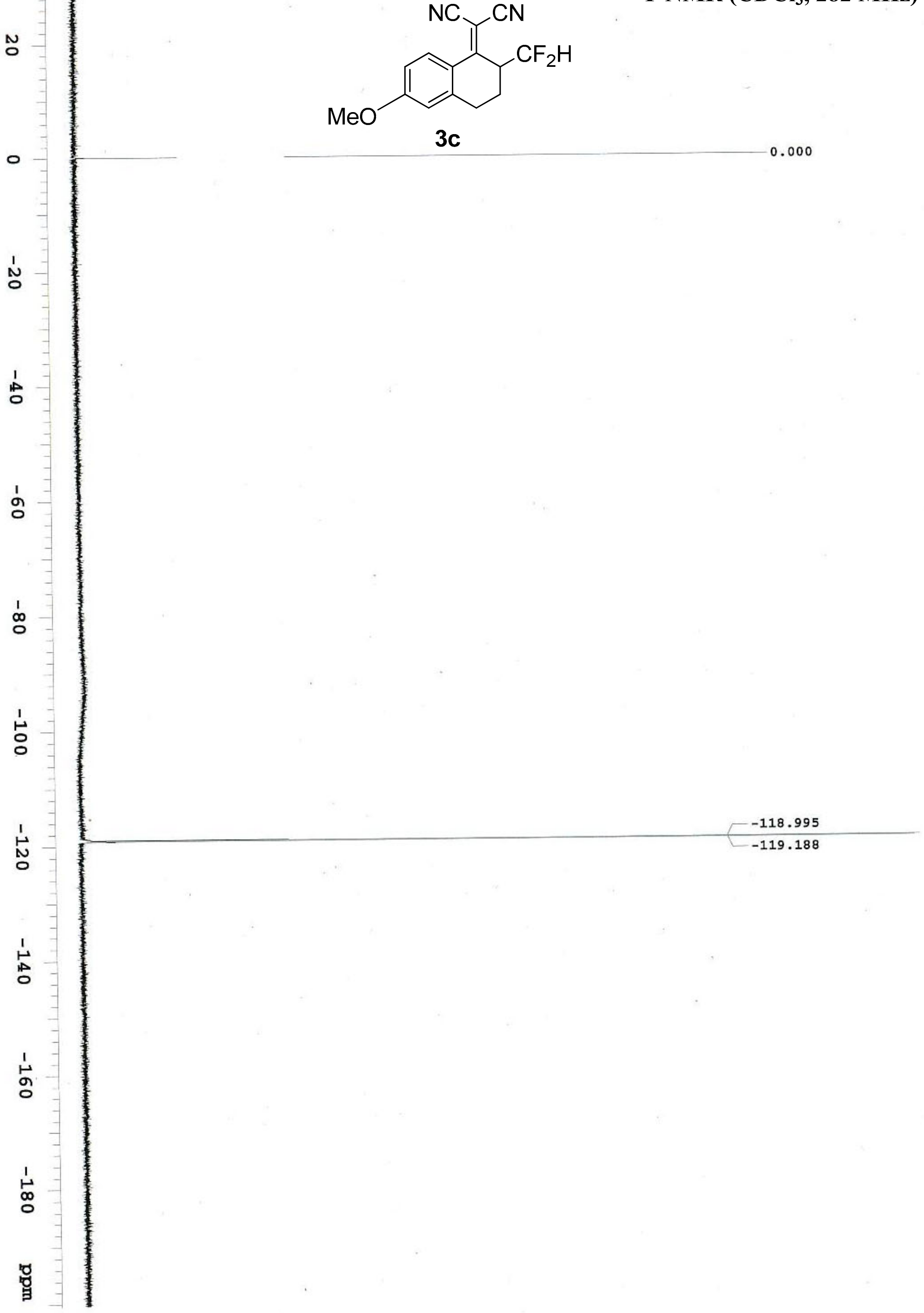

^{13}C NMR ($CDCl_3$, 150.9 MHz)

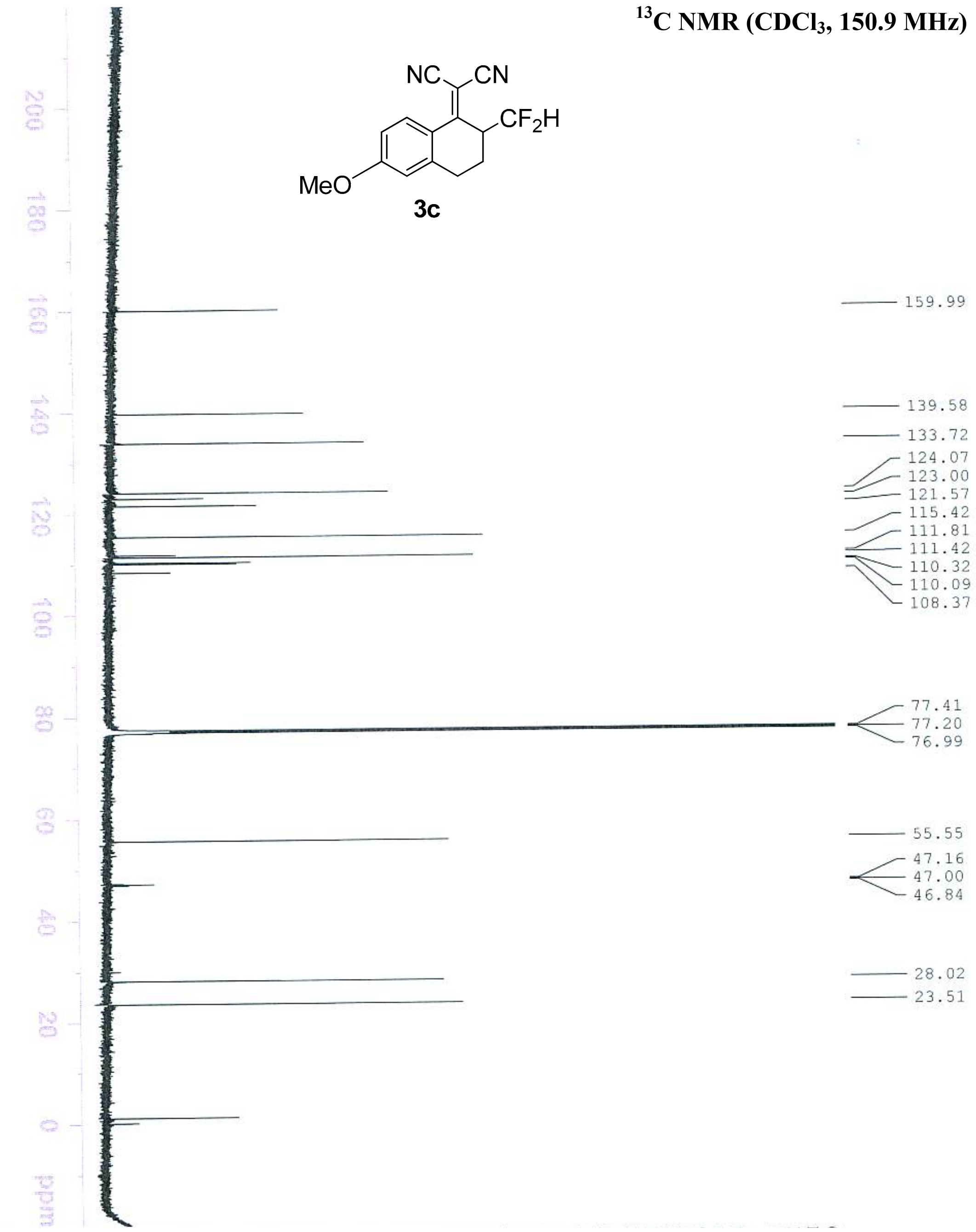

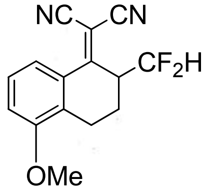

3d

^{1}H NMR ($CDCl_3$, 300 MHz)

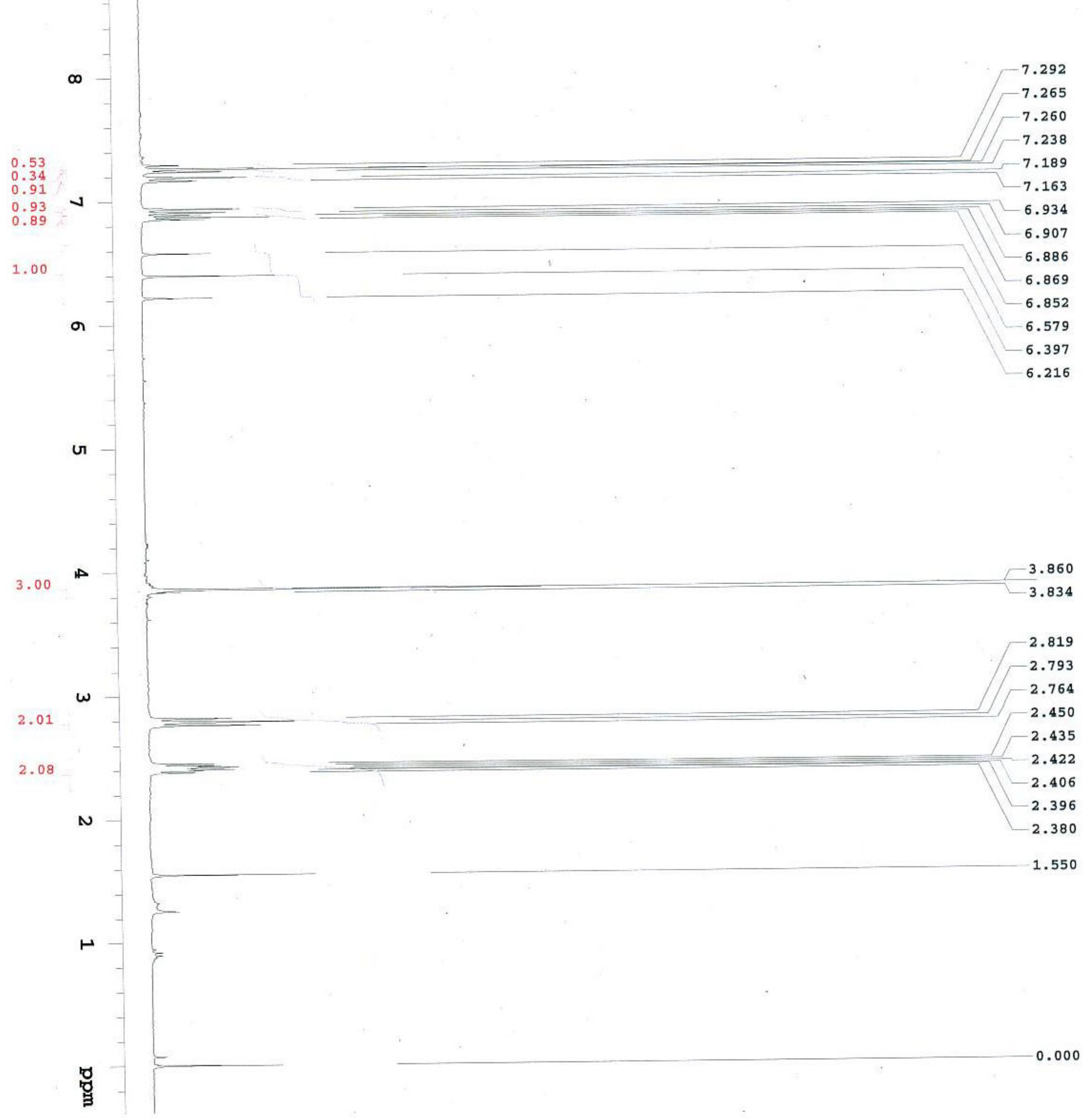

^{19}F NMR ($CDCl_3$, 282 MHz)

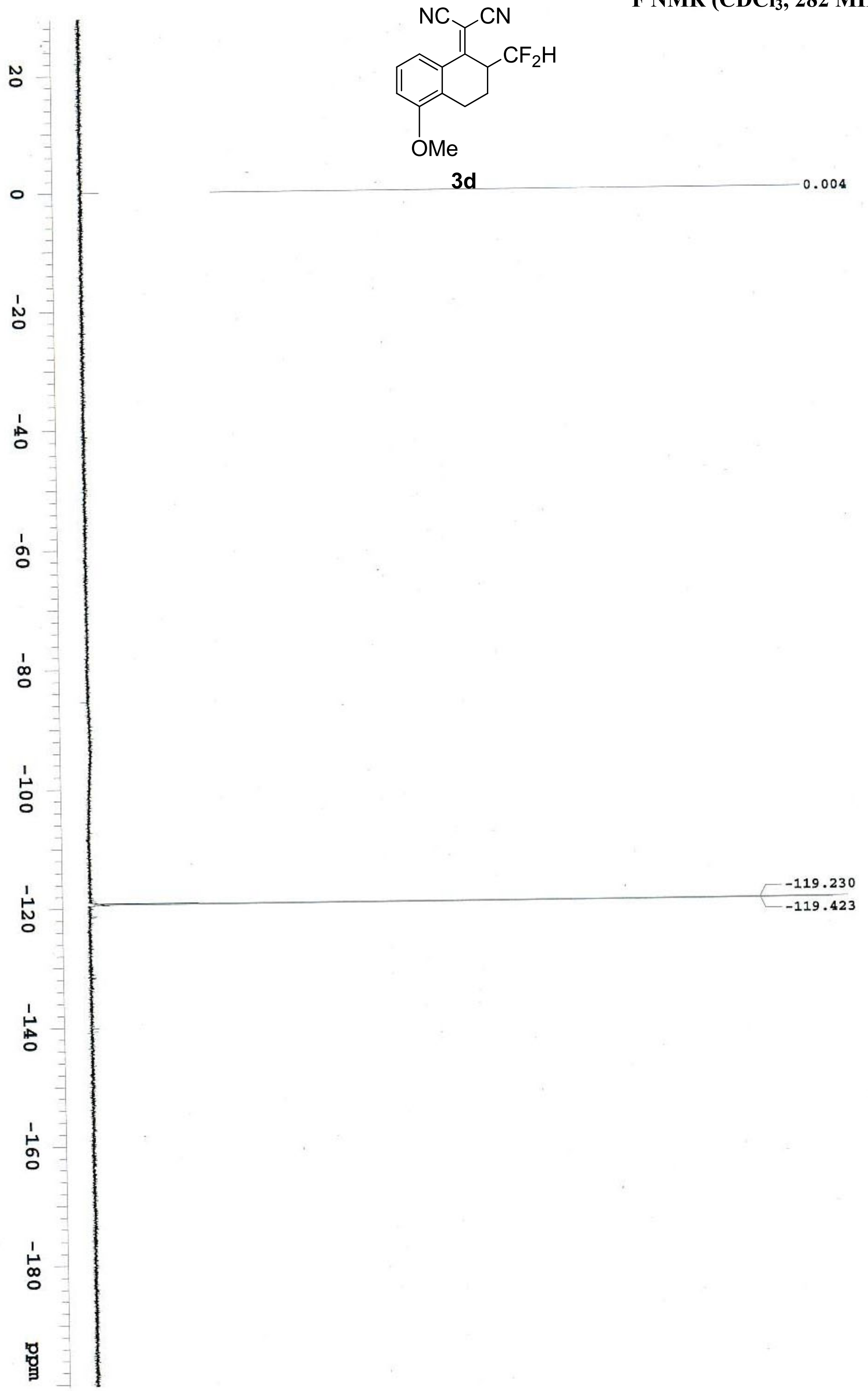

3d

^{13}C NMR ($CDCl_3$, 150.9 MHz)

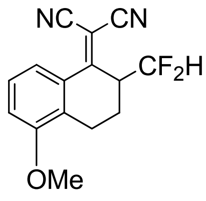

3d

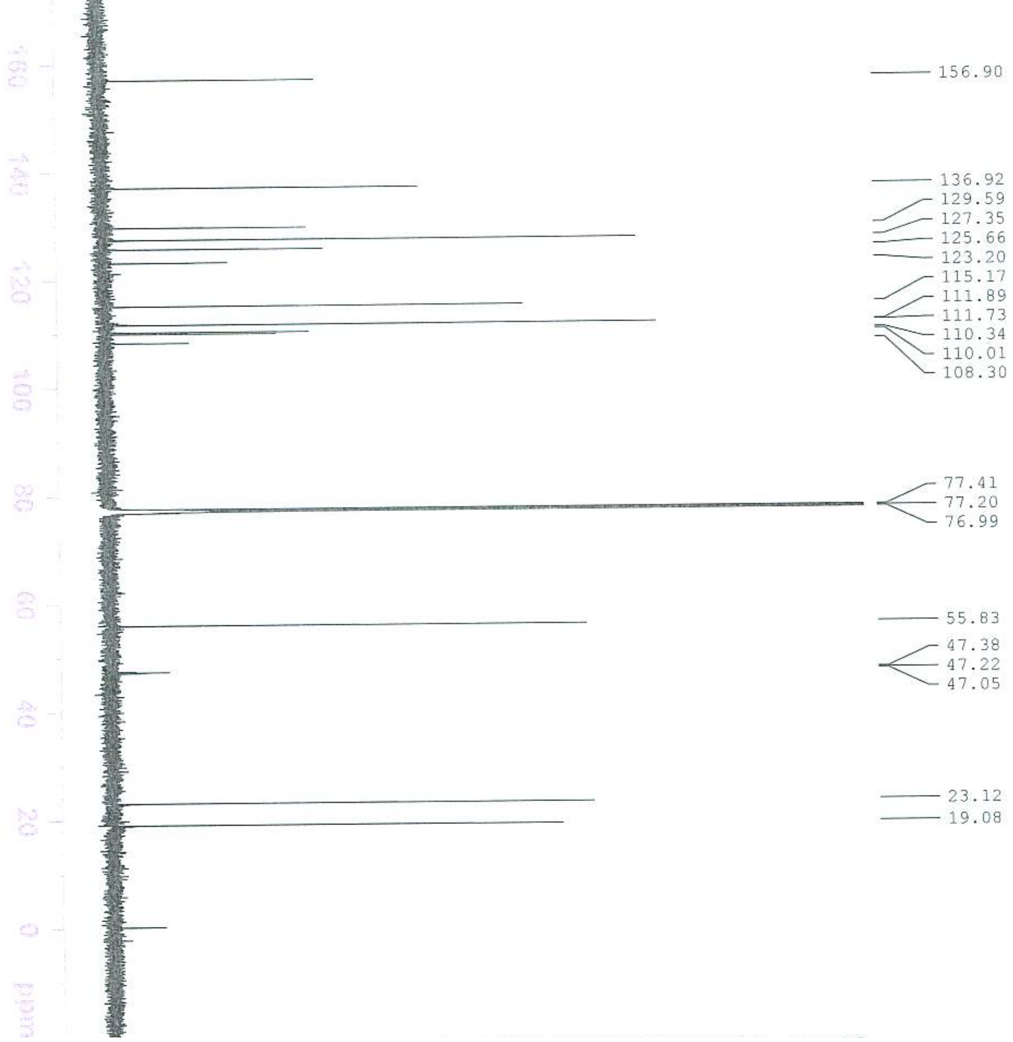

^1H NMR ($CDCl_3$, 300 MHz)

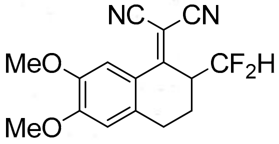

3e

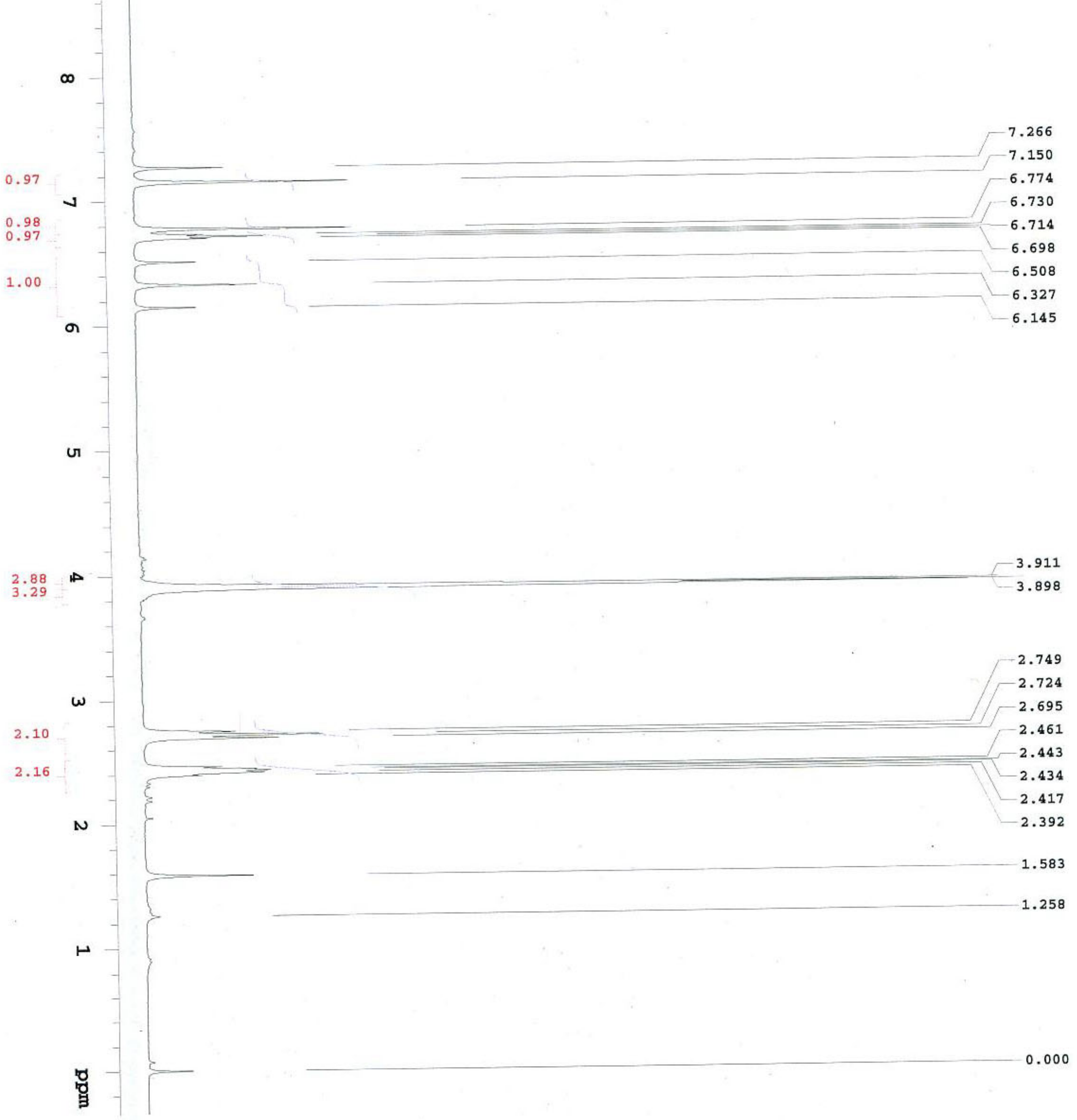

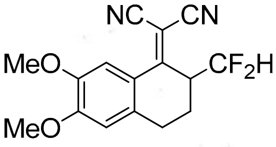

3e

^{19}F NMR (CDCl$_3$, 282 MHz)

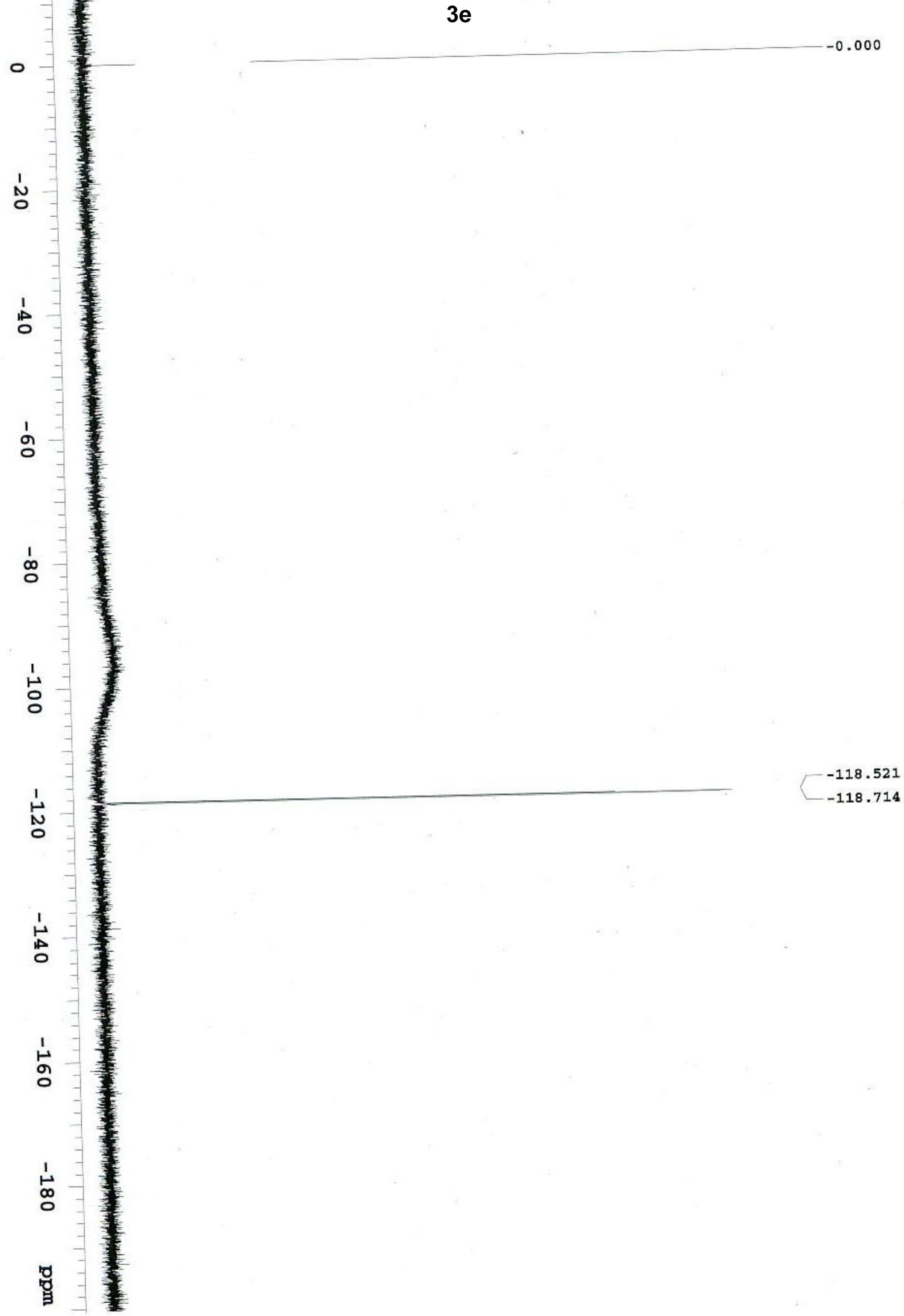

^{13}C NMR ($CDCl_3$, 150.9 MHz)

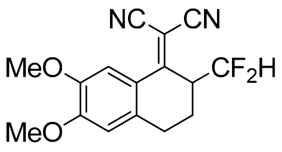

3e

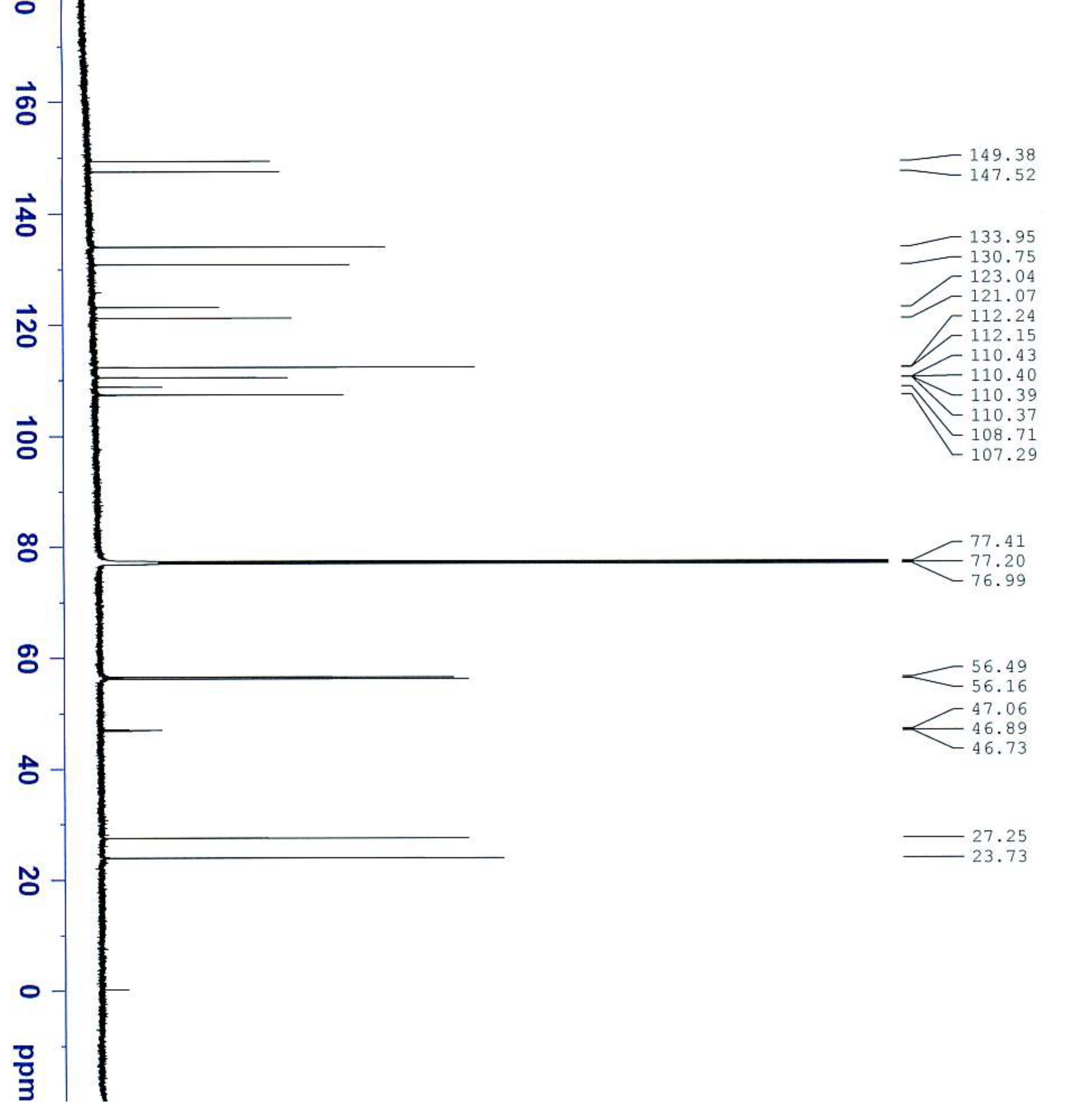

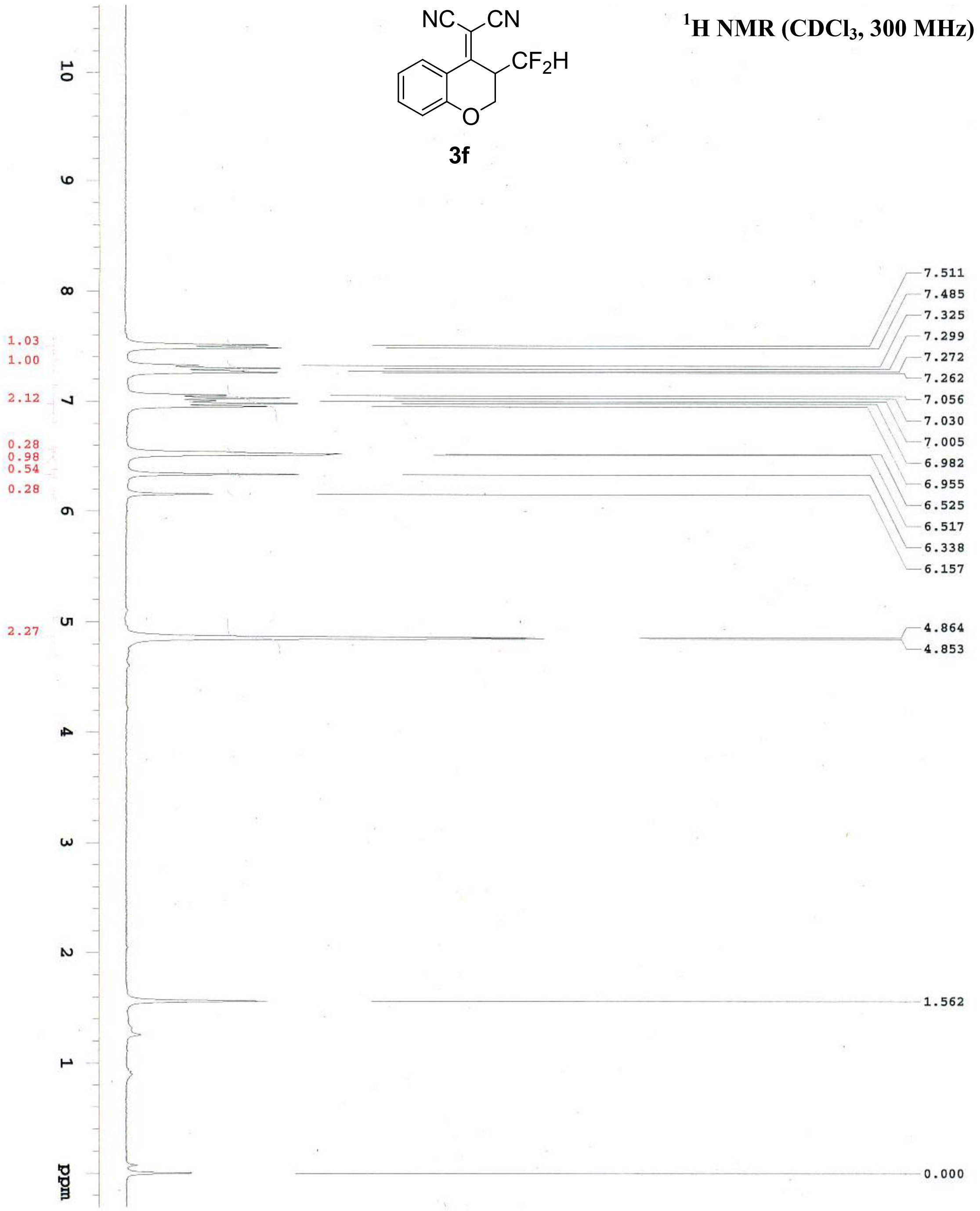
NC CN
CF2H
O
3f
1H NMR (CDCl3, 300 MHz)
7.511
7.485
7.325
7.299
7.272
7.262
7.056
7.030
7.005
6.982
6.955
6.525
6.517
6.338
6.157
4.864
4.853
1.562
0.000
1.03
1.00
2.12
0.28
0.98
0.54
0.28
2.27
10
9
8
7
6
5
4
3
2
1
ppm

^{19}F NMR ($CDCl_3$, 282 MHz)

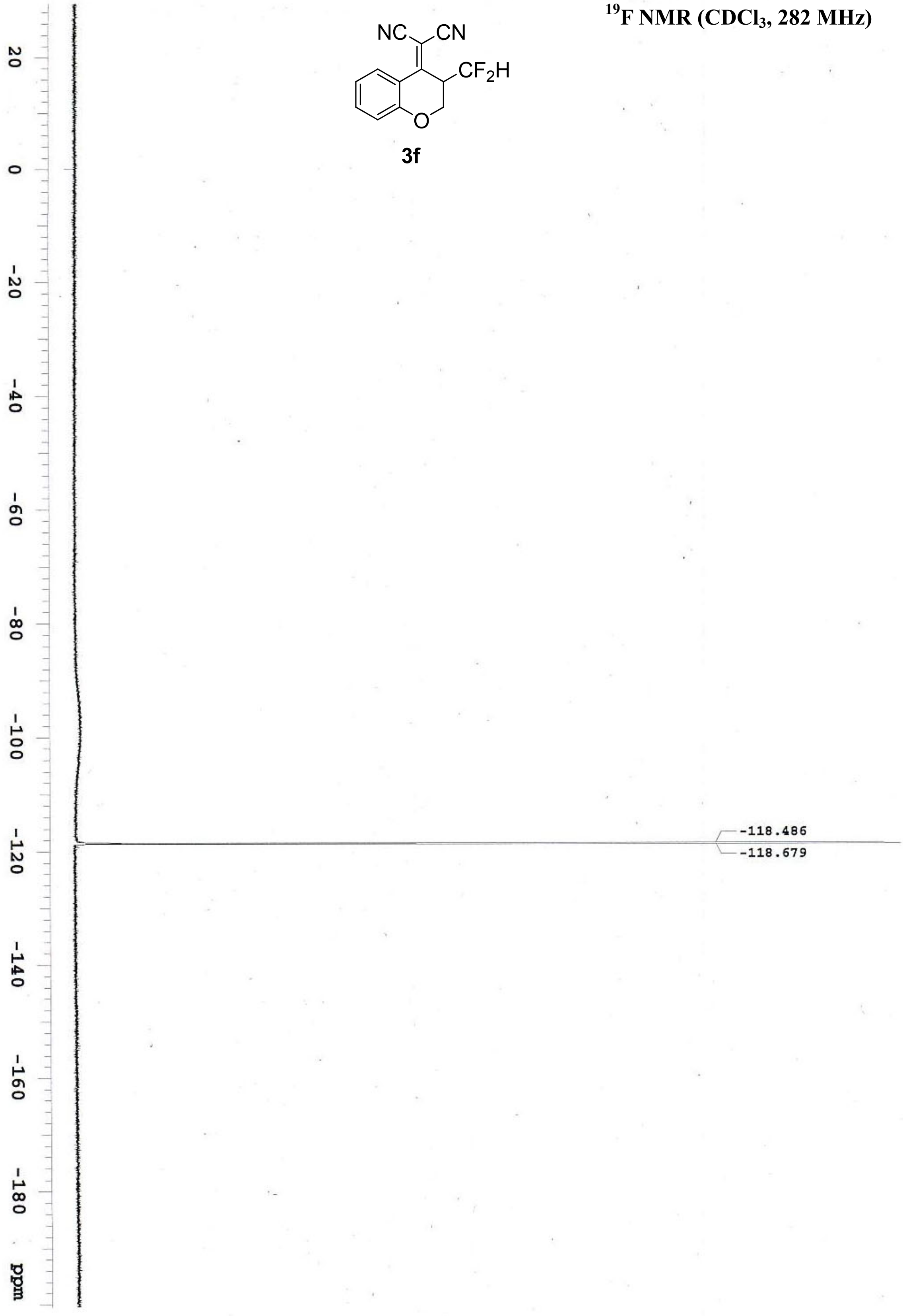

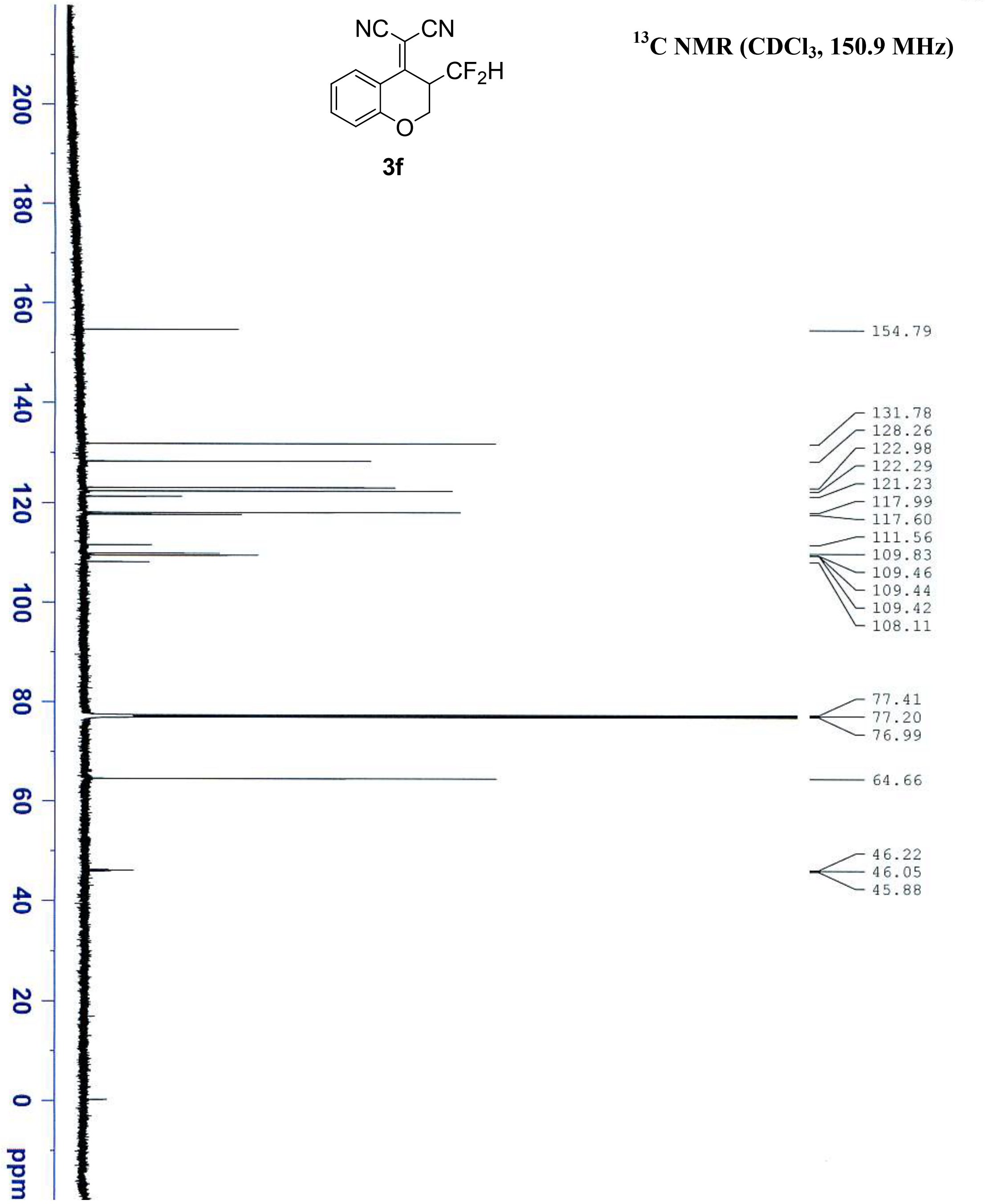

3f

^{13}C NMR ($CDCl_3$, 150.9 MHz)

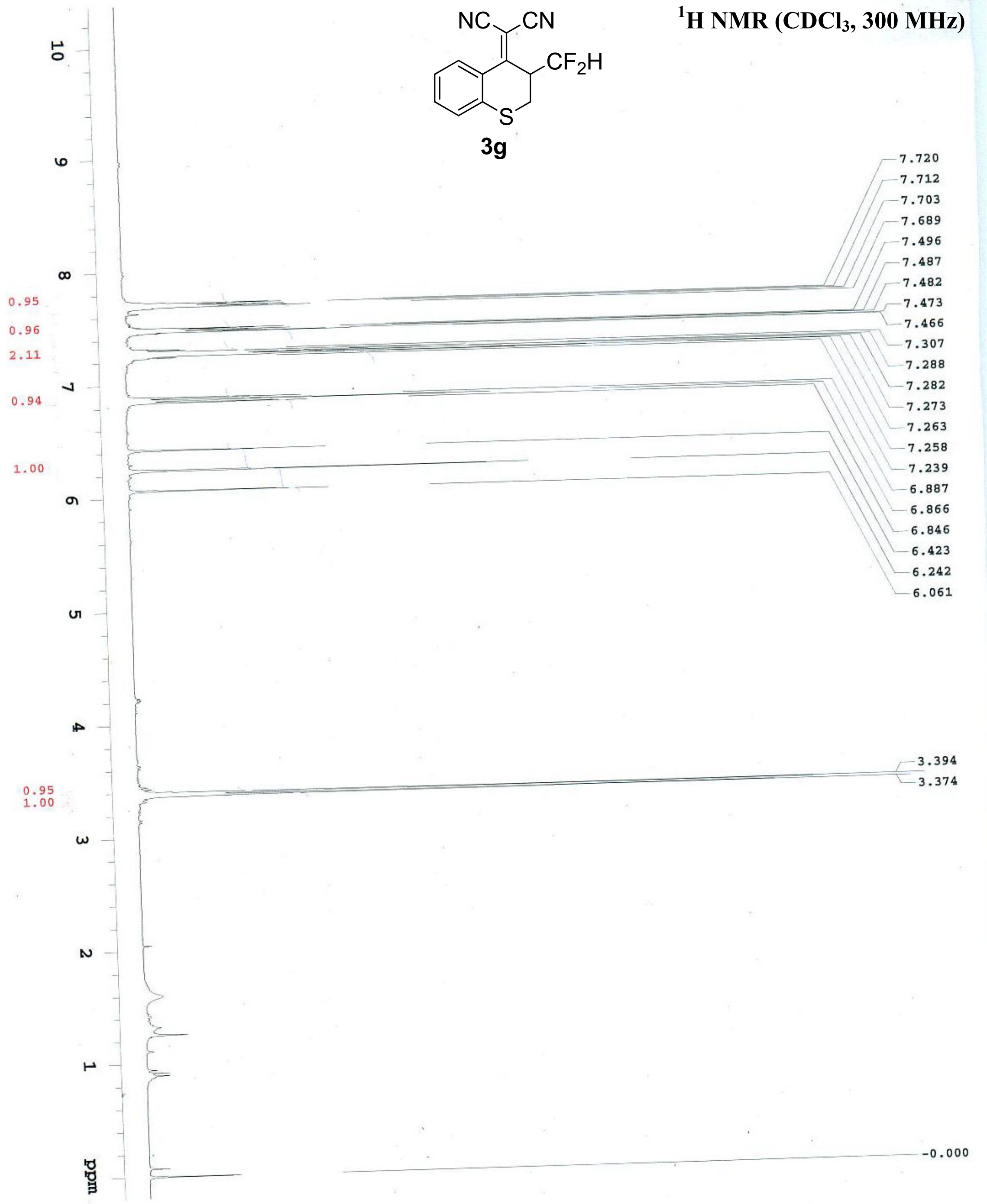

^{1}H NMR ($CDCl_3$, 300 MHz)
NC
CN
CF_2H
S
3g
7.720
7.712
7.703
7.689
7.496
7.487
7.482
7.473
7.466
7.307
7.288
7.282
7.273
7.263
7.258
7.239
6.887
6.866
6.846
6.423
6.242
6.061
3.394
3.374
-0.000
0.95
0.96
2.11
0.94
1.00
0.95
1.00
10
9
8
7
6
5
4
3
2
1
ppm

^{19}F NMR ($CDCl_3$, 282 MHz)

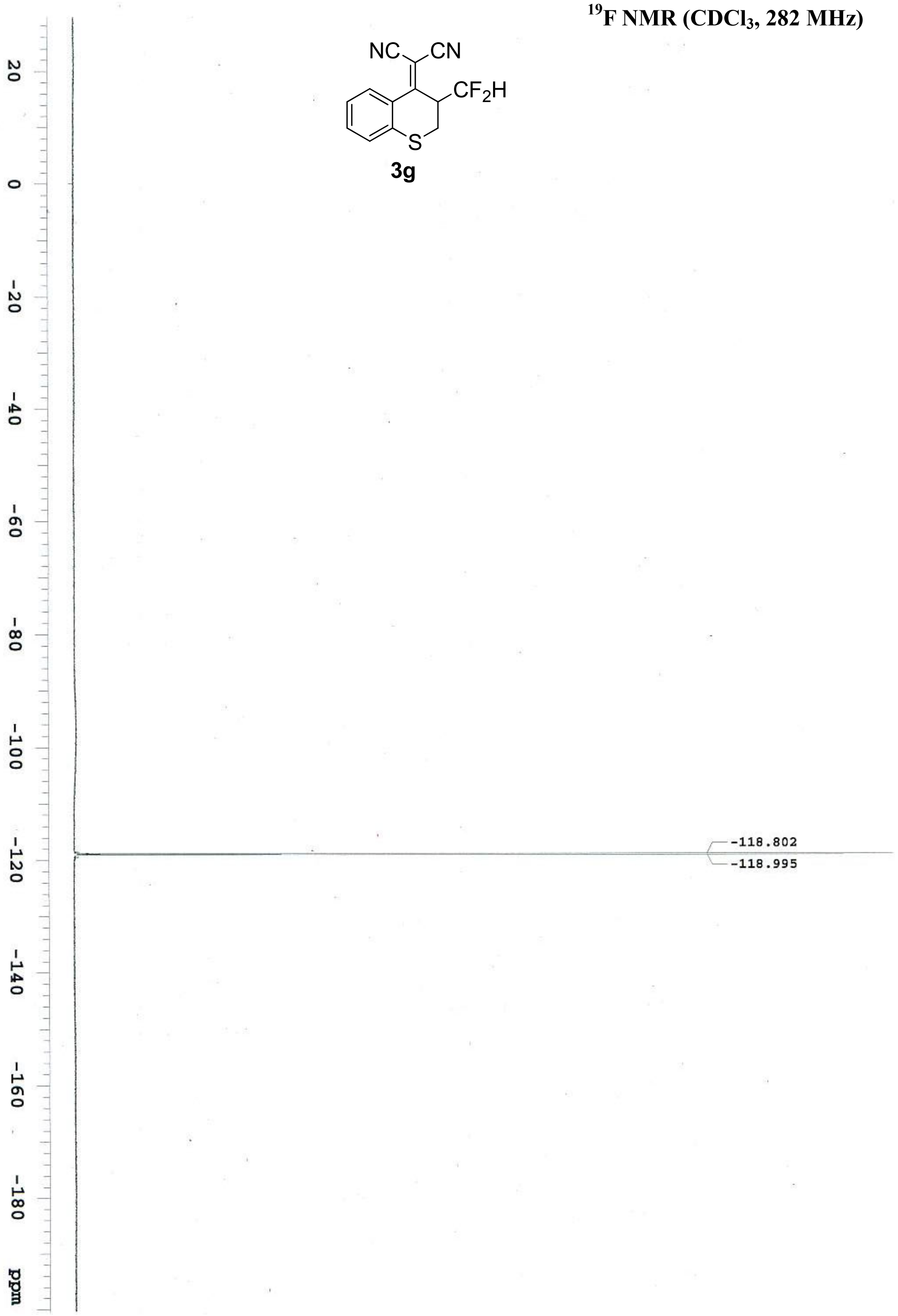

^{13}C NMR ($CDCl_3$, 150.9 MHz)

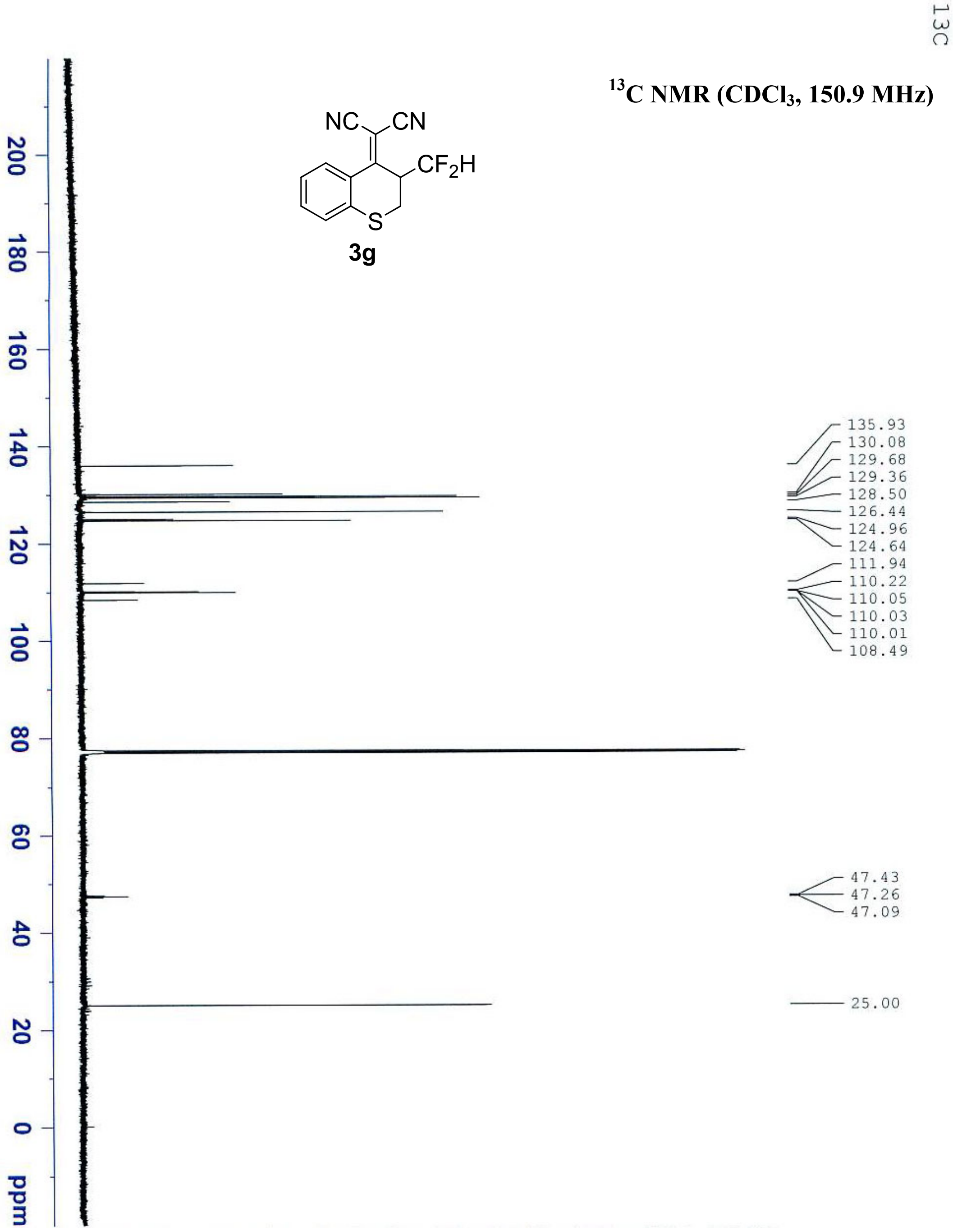

3g

^{1}H NMR ($CDCl_3$, 300 MHz)

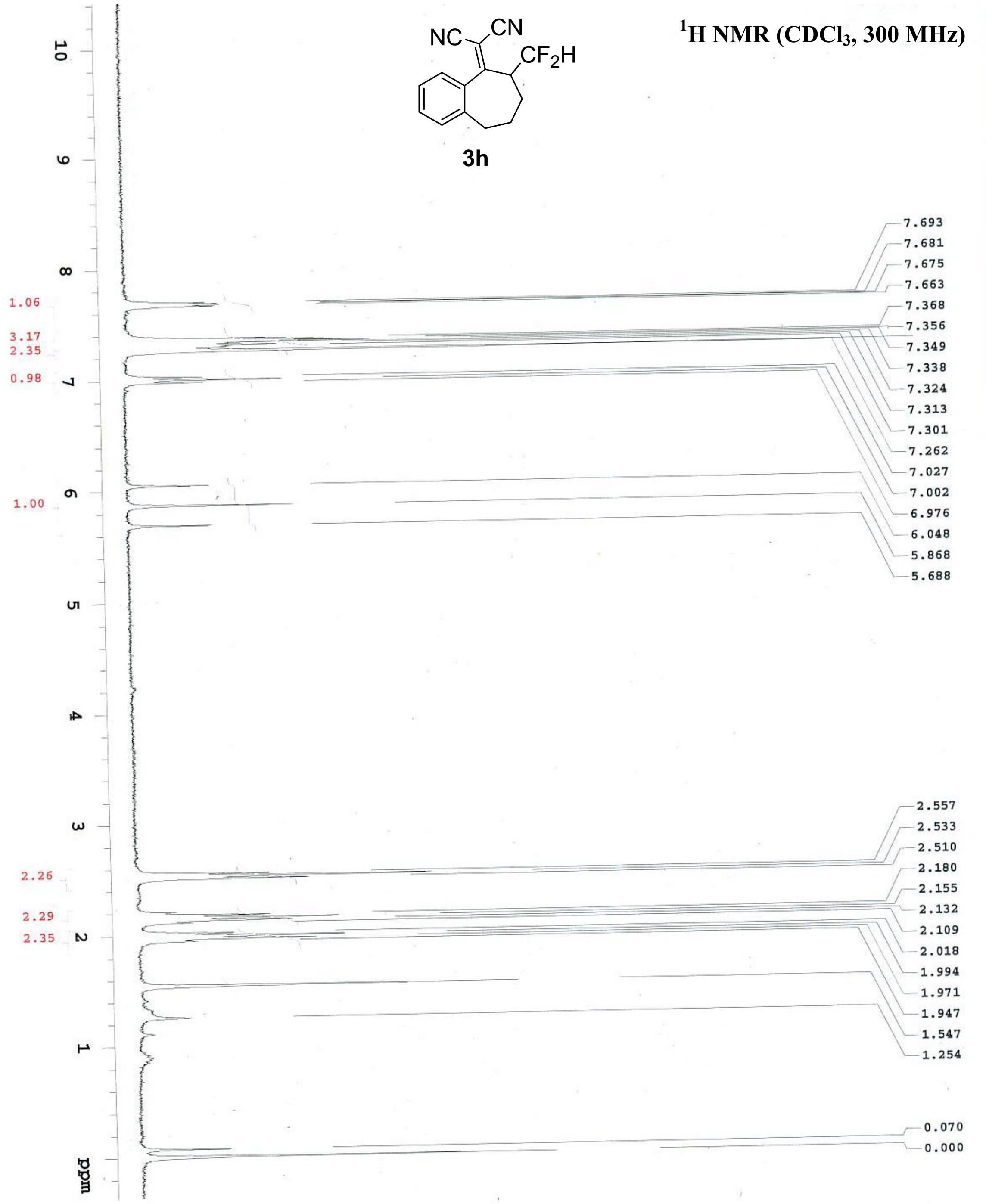

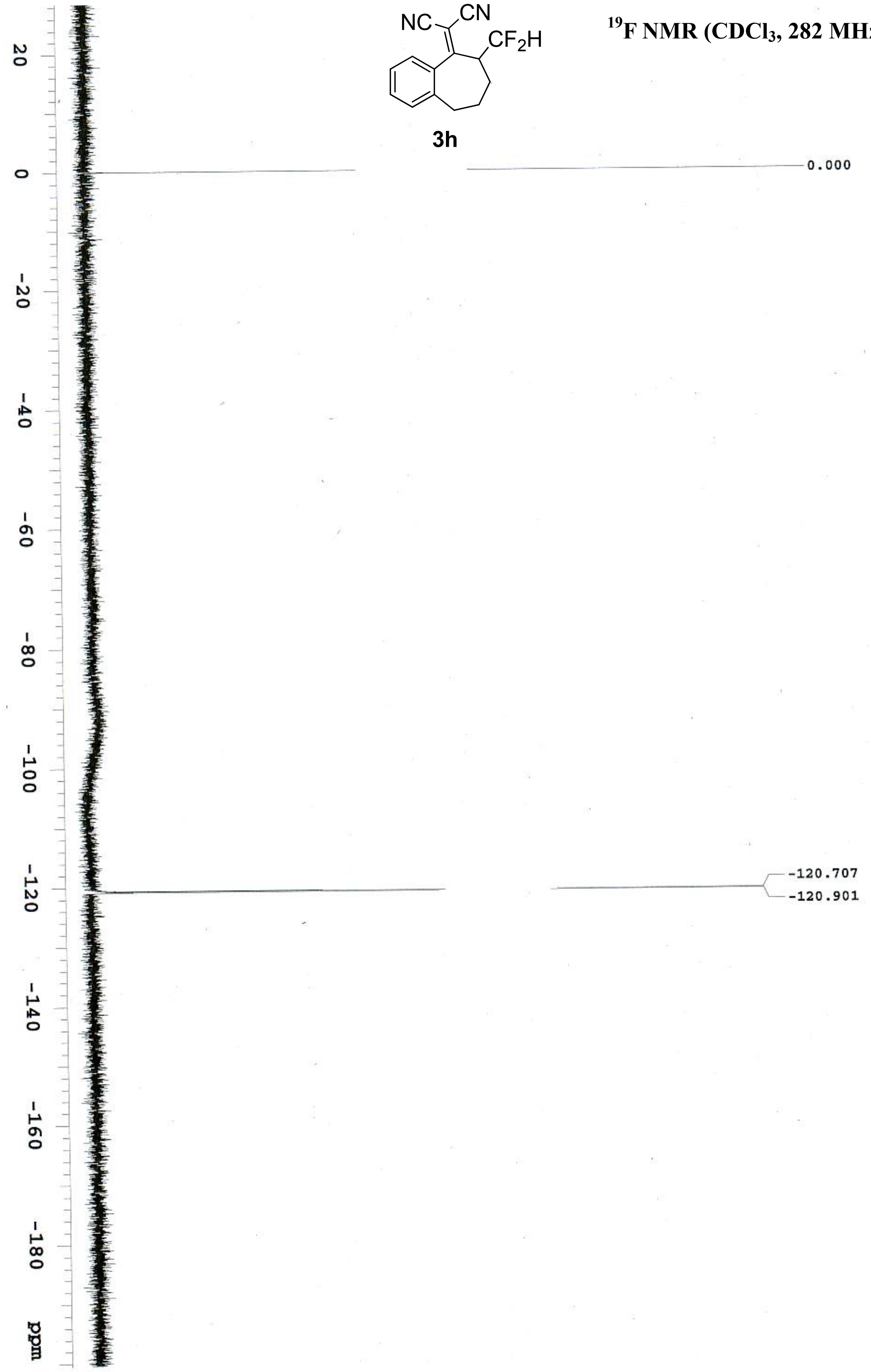

3h

^{19}F NMR (CDCl$_3$, 282 MHz)

^{13}C NMR ($CDCl_3$, 150.9 MHz)

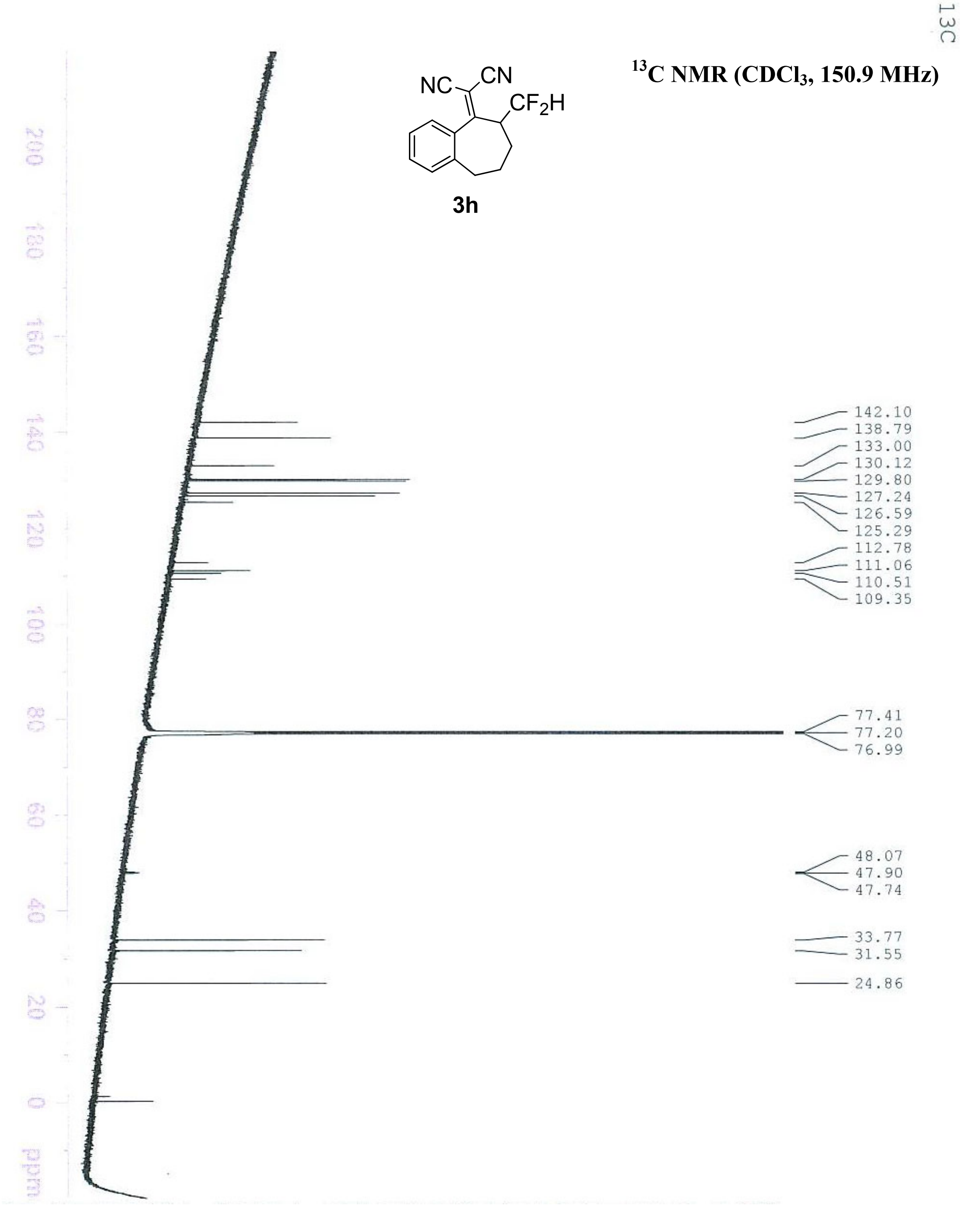

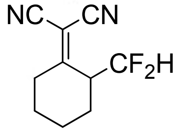

3i

^{1}H NMR ($CDCl_3$, 300 MHz)

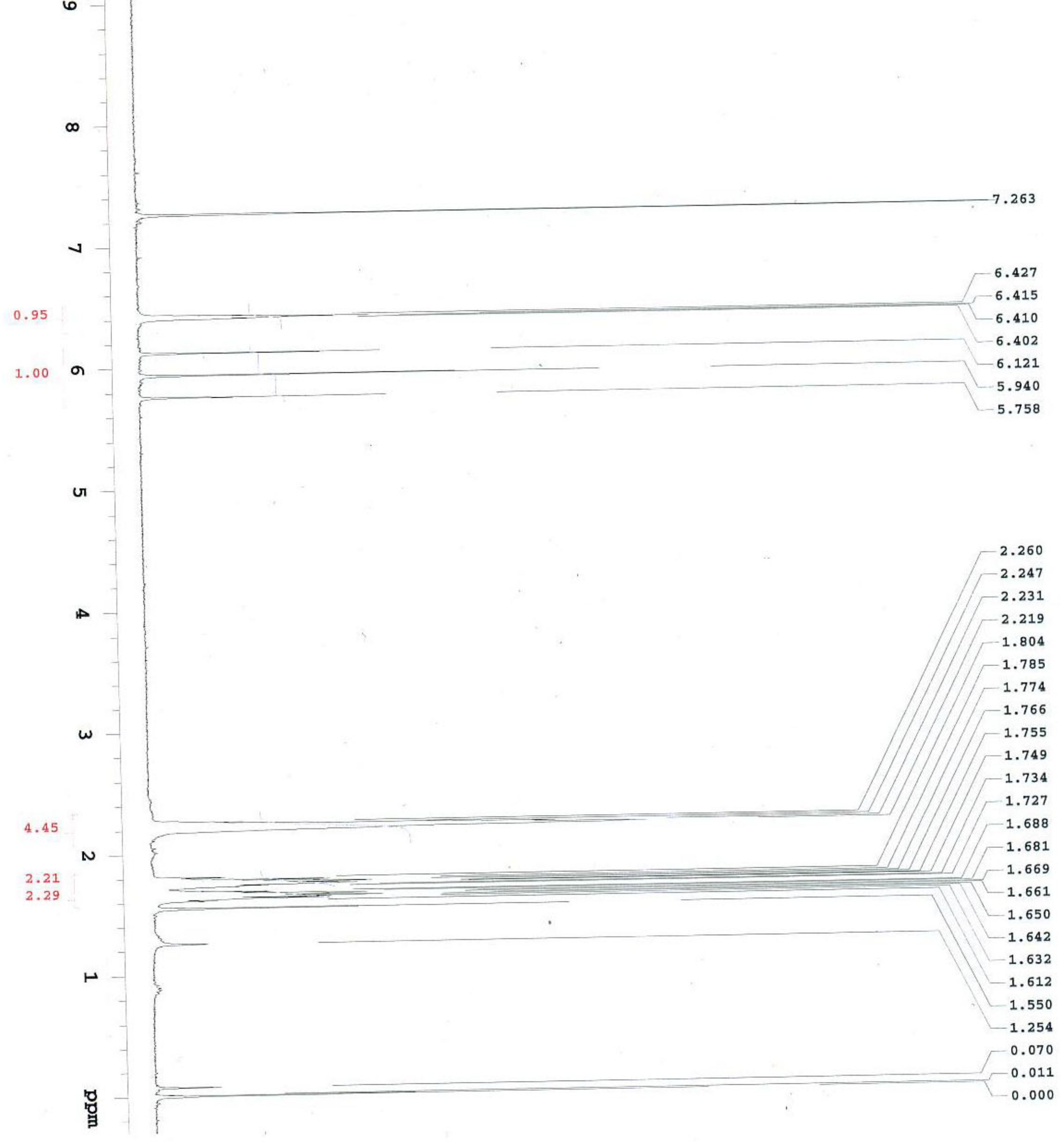

^{19}F NMR ($CDCl_3$, 282 MHz)

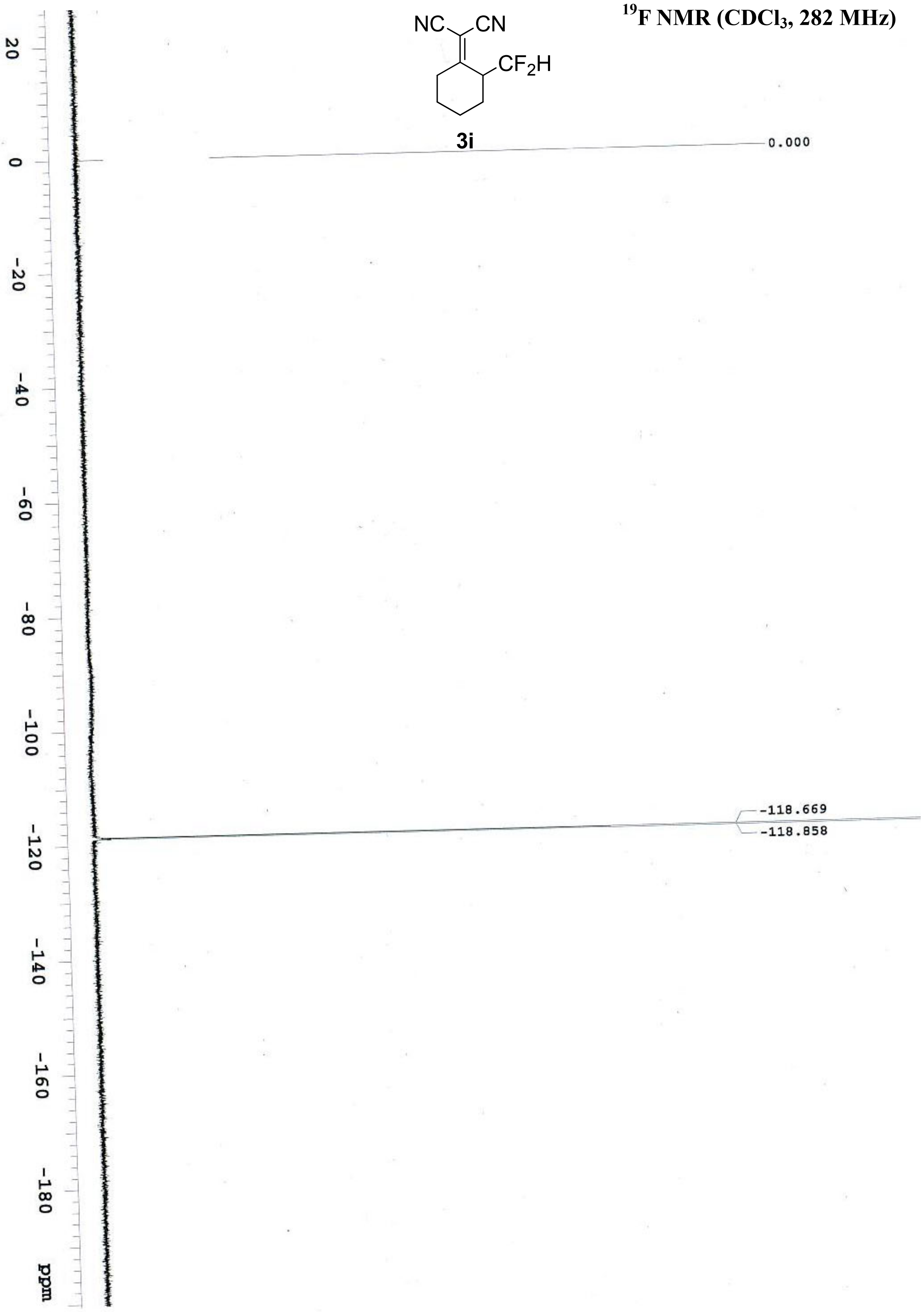

^{13}C NMR (CDCl$_3$, 150.9 MHz)

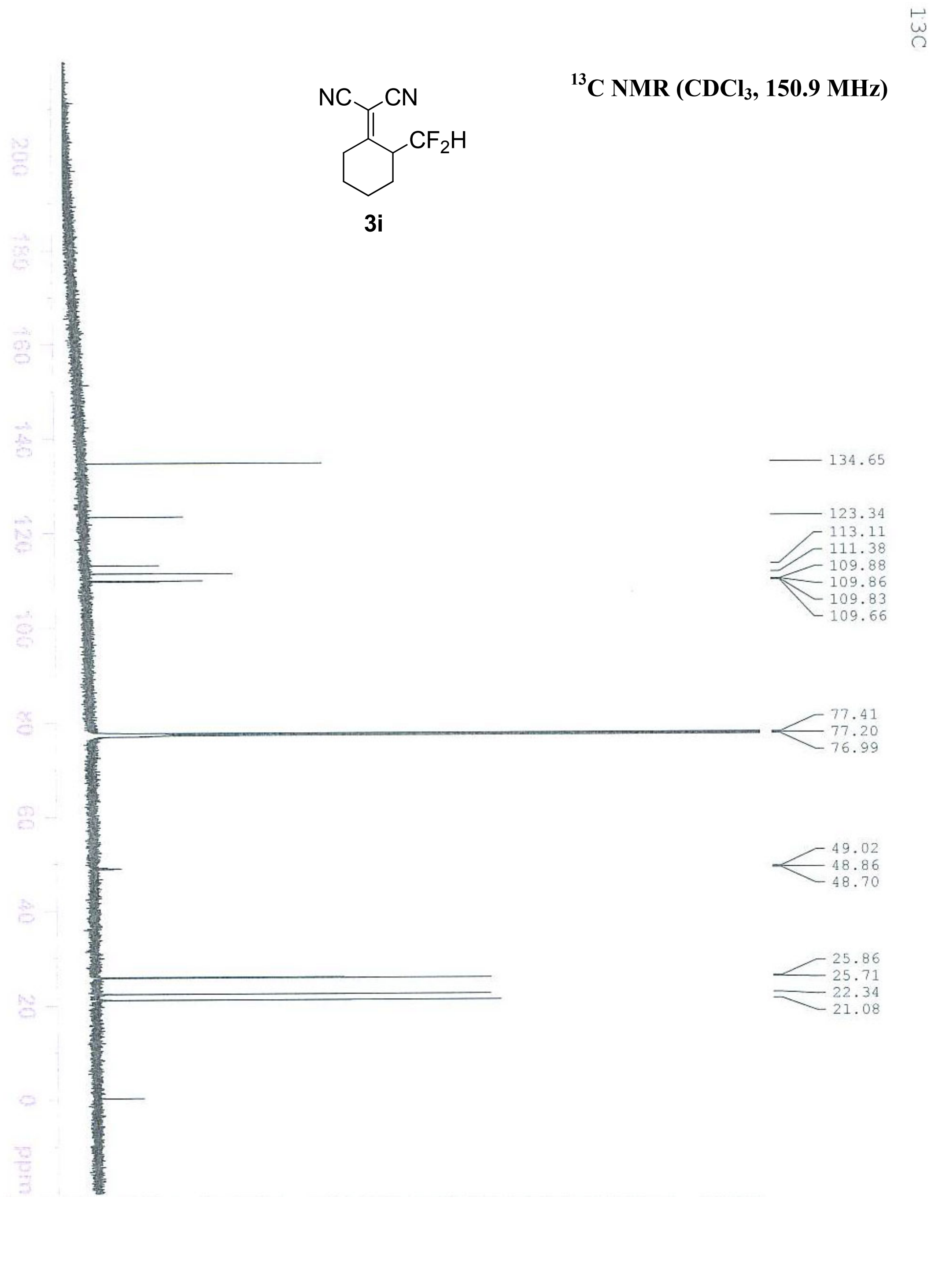

^{1}H NMR ($CDCl_3$, 300 MHz)

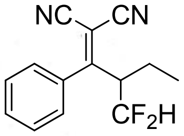

3j

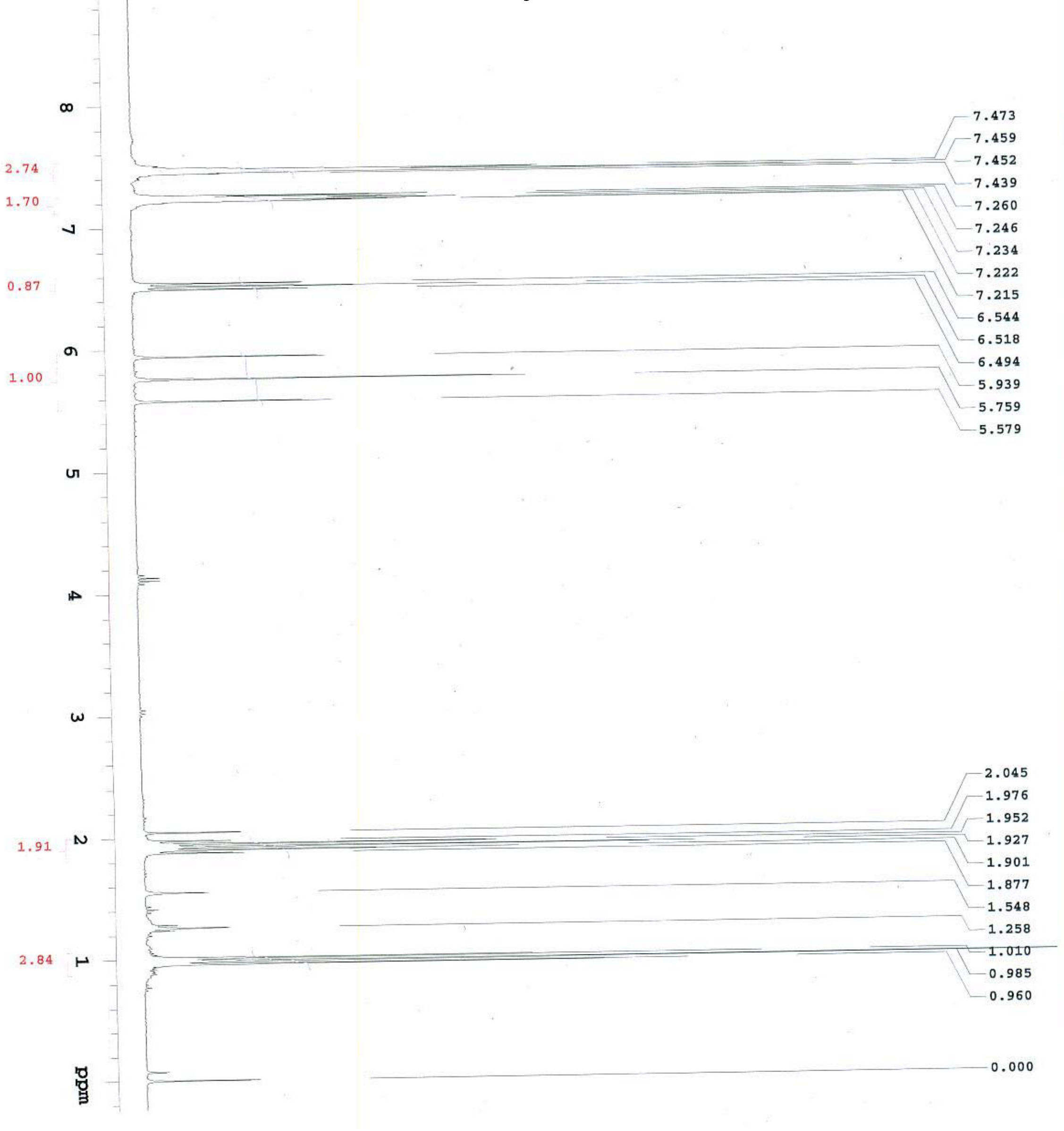

^{19}F NMR ($CDCl_3$, 282 MHz)

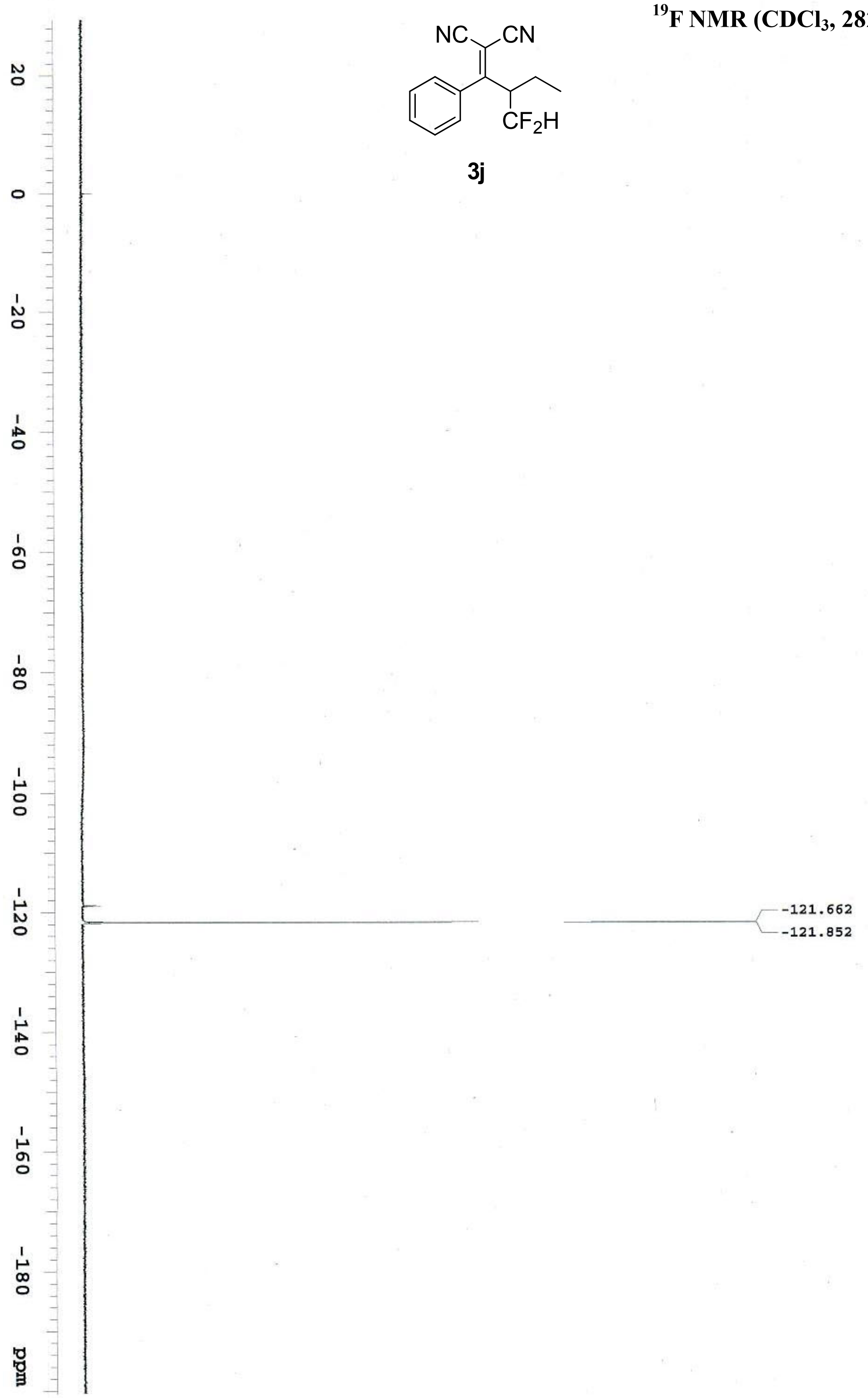

3j

^{13}C NMR ($CDCl_3$, 150.9 MHz)

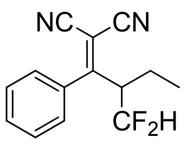

3j

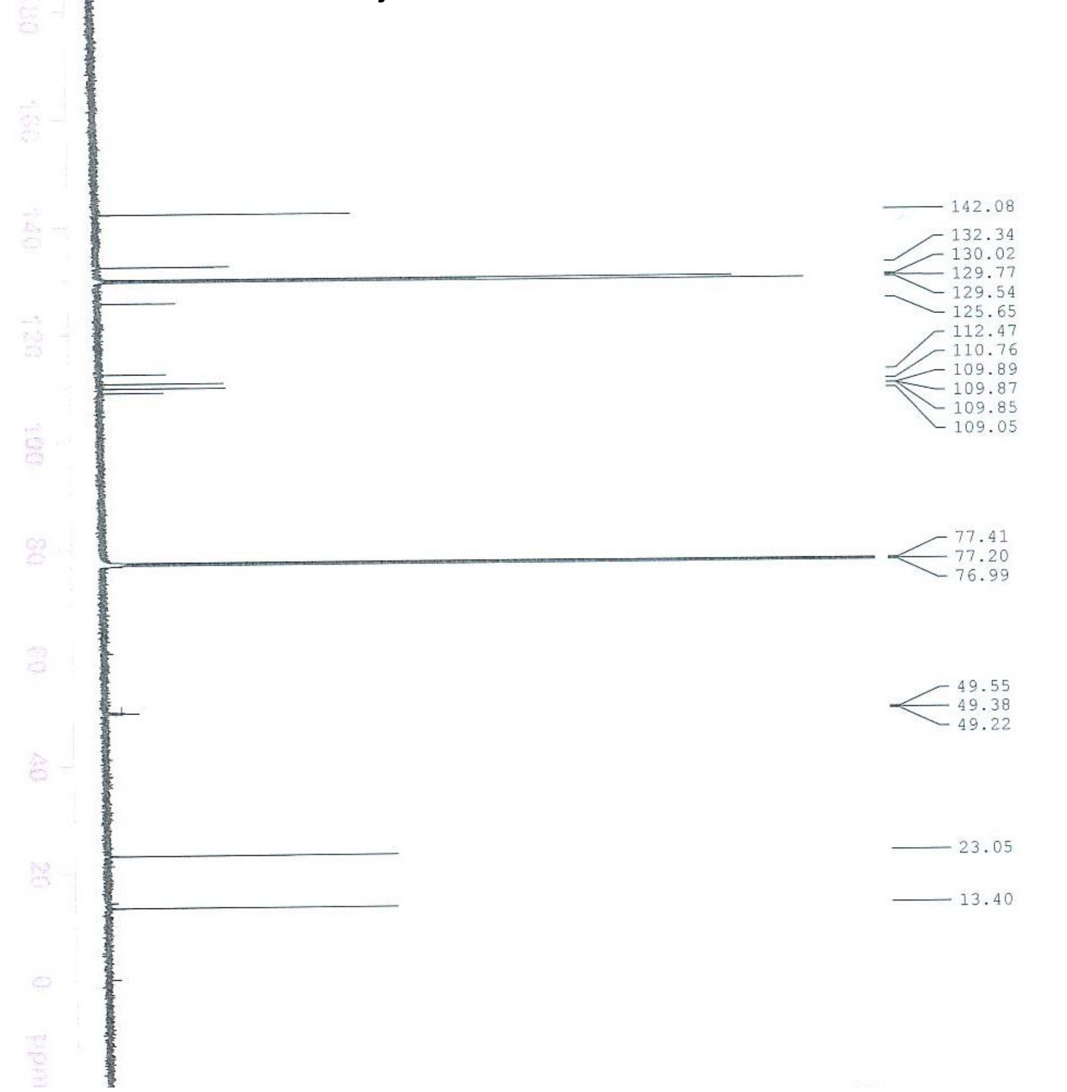

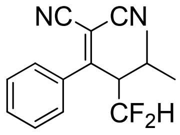

3k

^{1}H NMR ($CDCl_3$, 300 MHz)

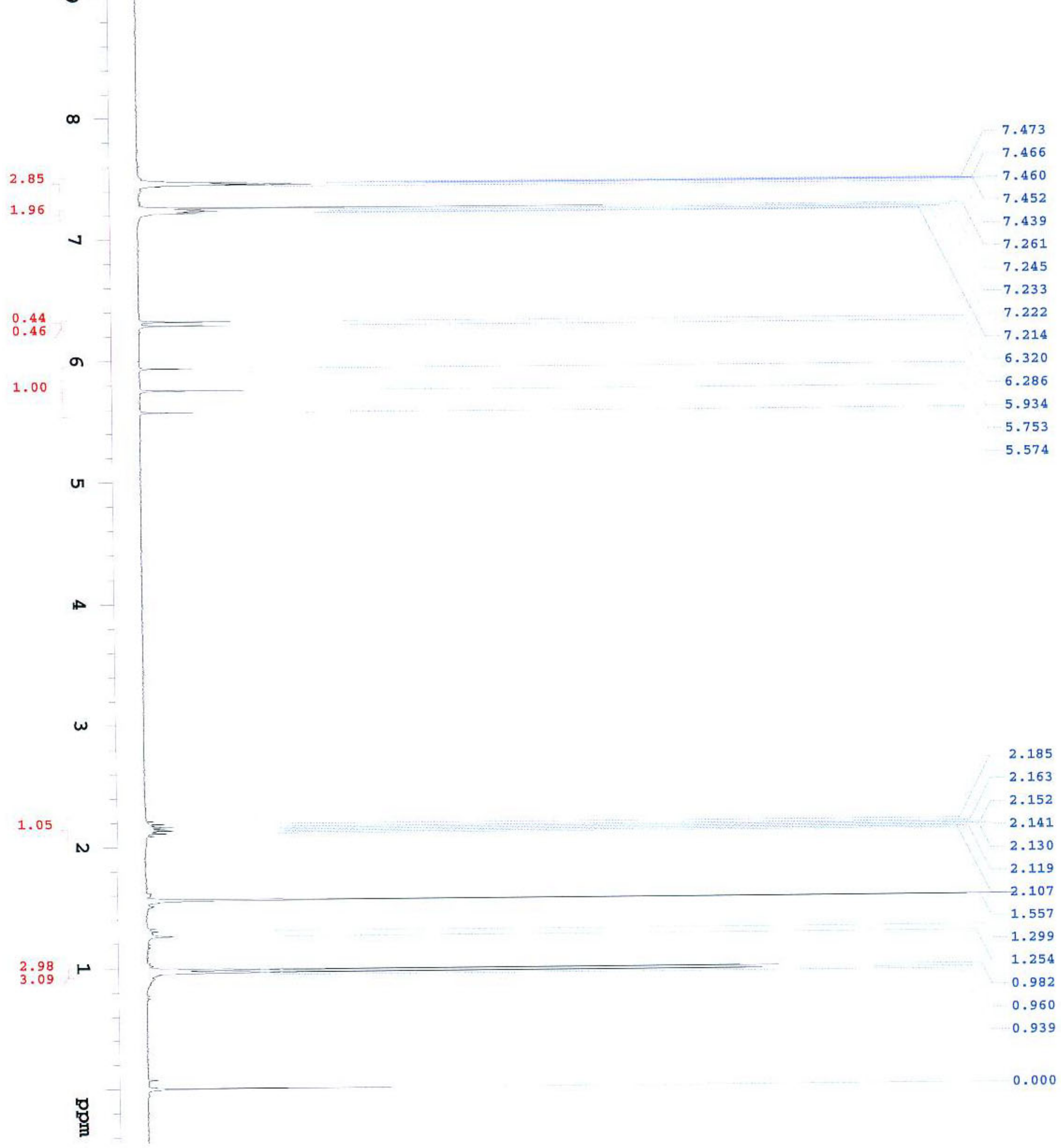

^{19}F NMR ($CDCl_3$, 282 MHz)

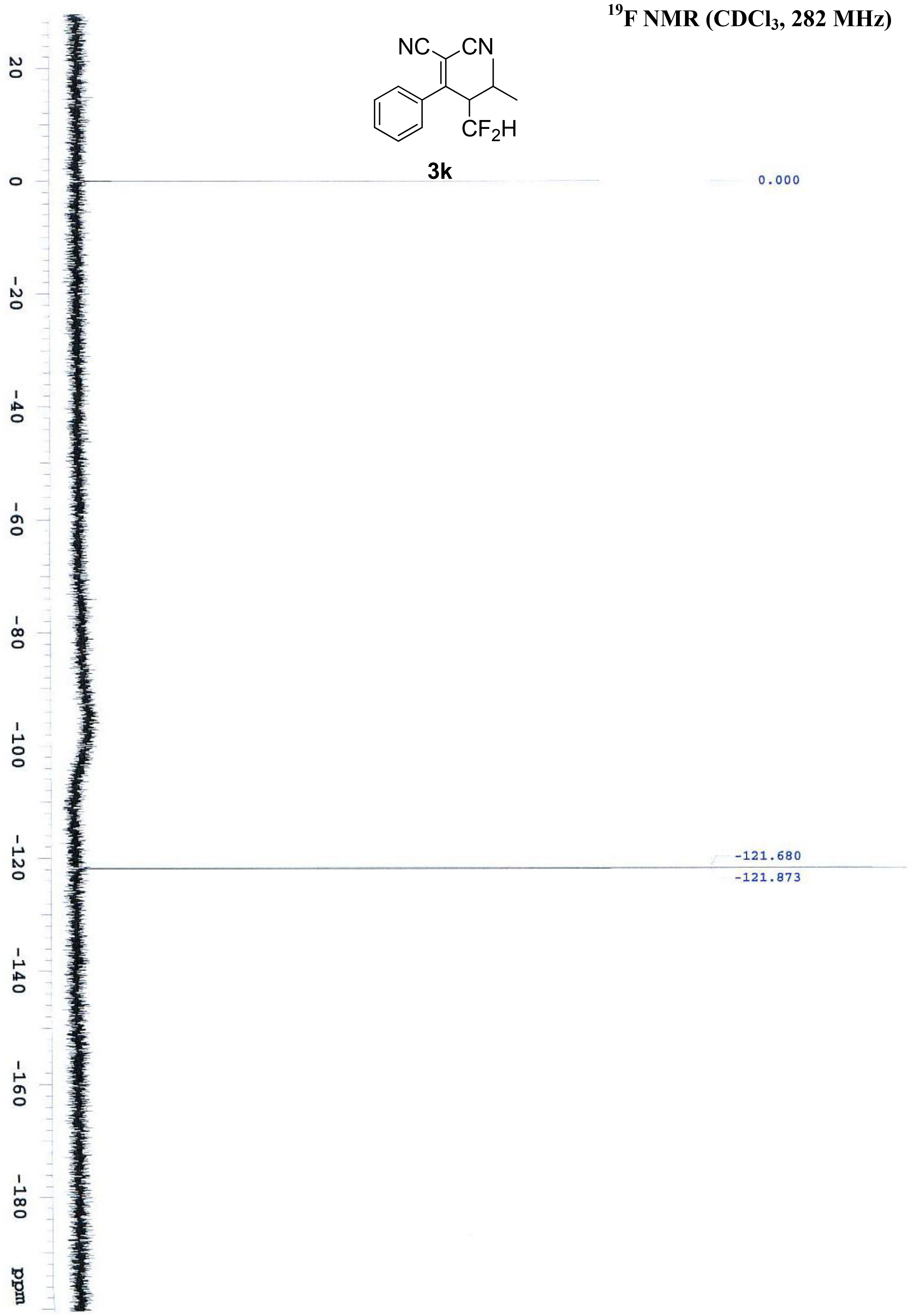

3k

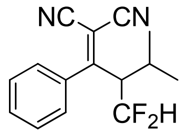

3k

^{13}C NMR (CDCl$_3$, 150.9 MHz)

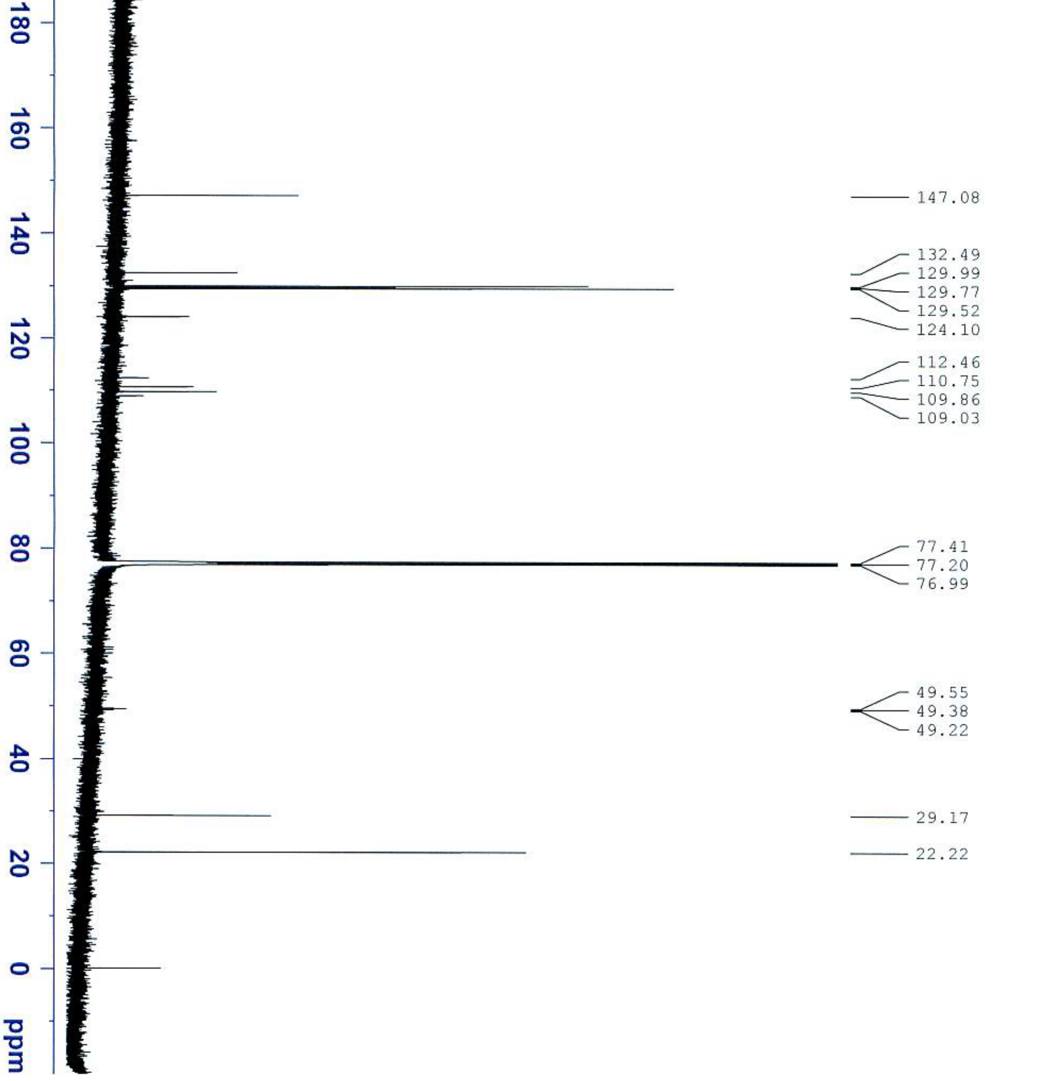

^{1}H NMR ($CDCl_3$, 300 MHz)

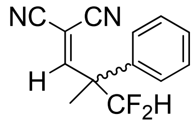

3l

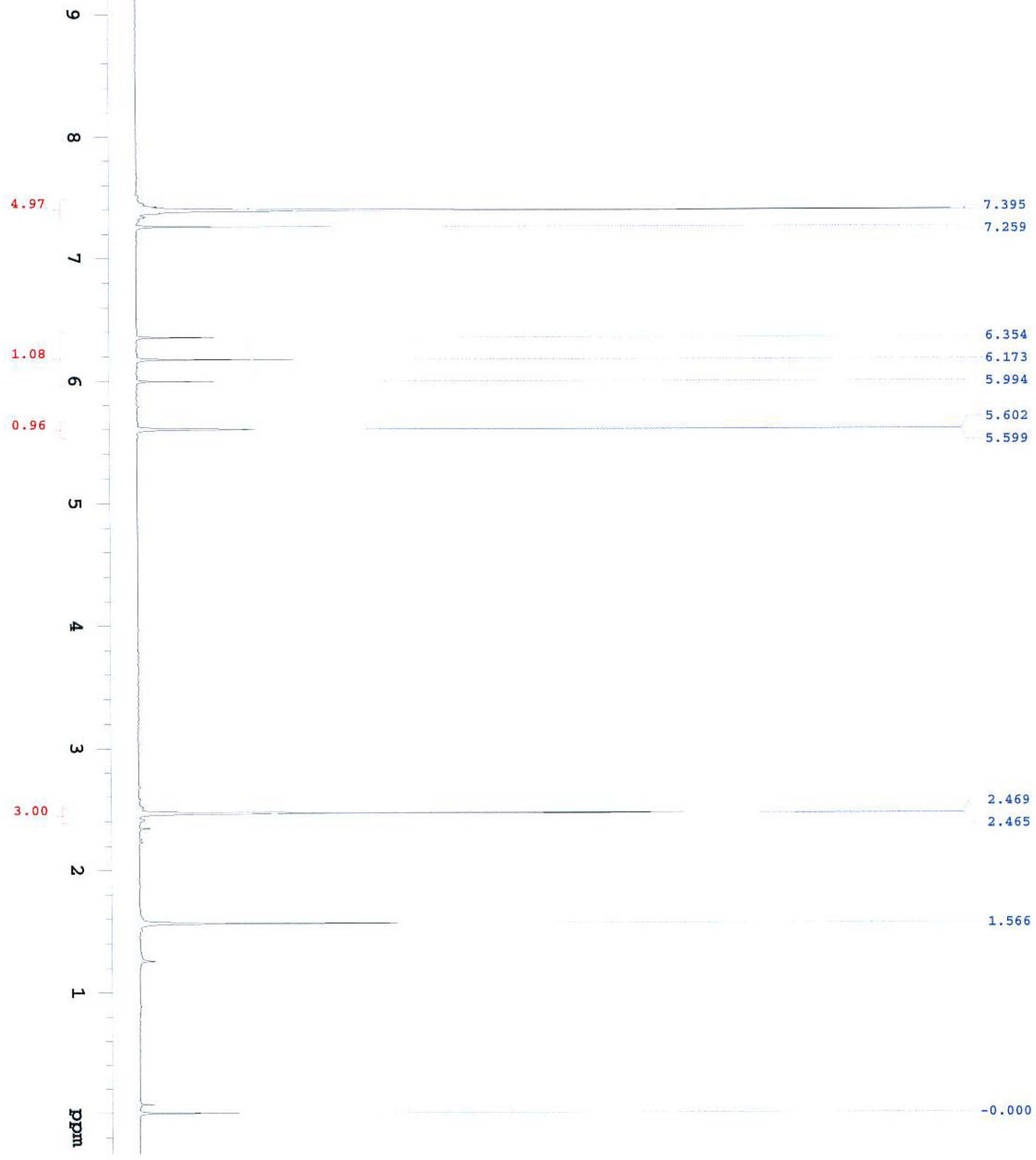

^{19}F NMR ($CDCl_3$, 282 MHz)

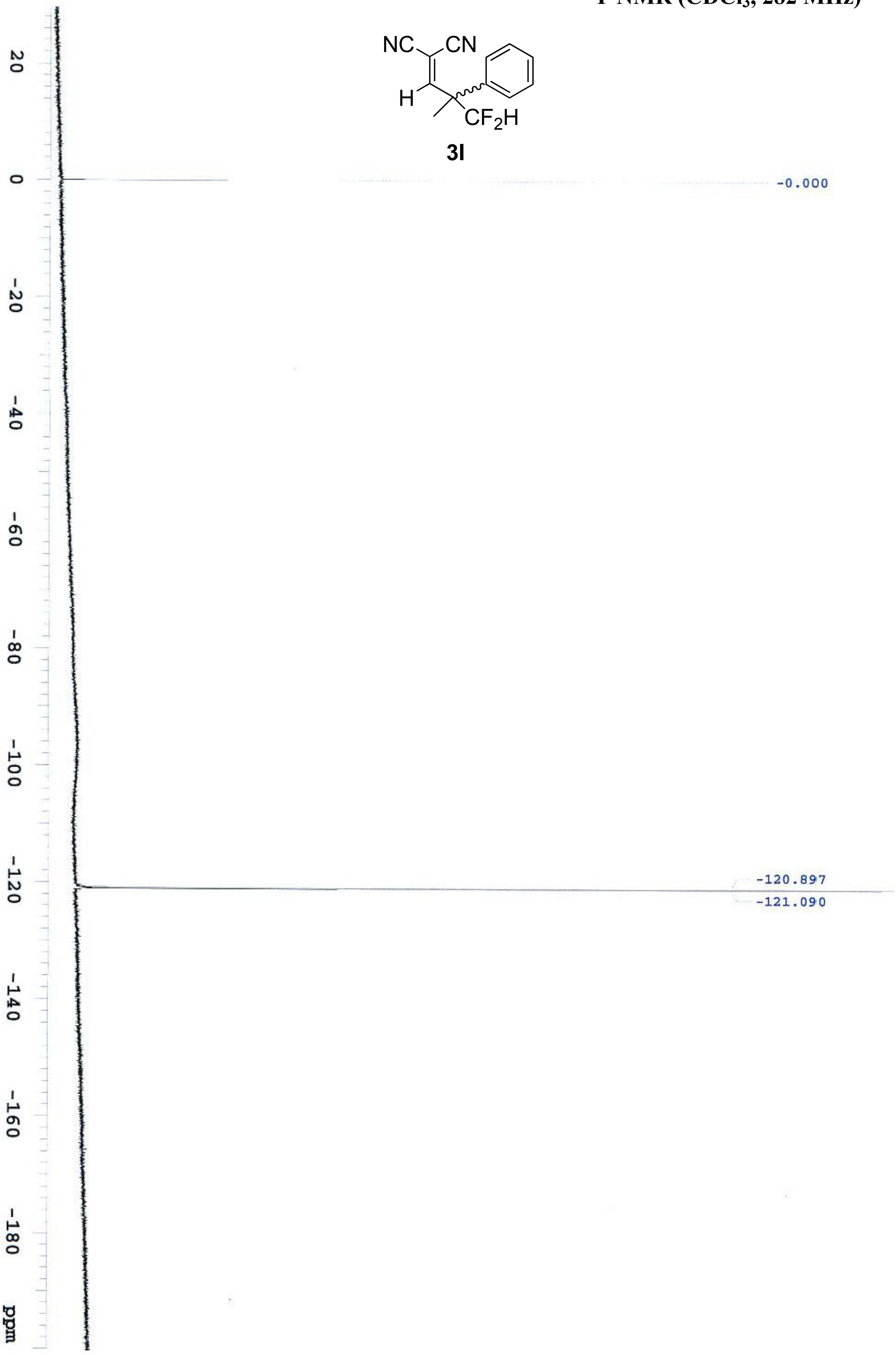

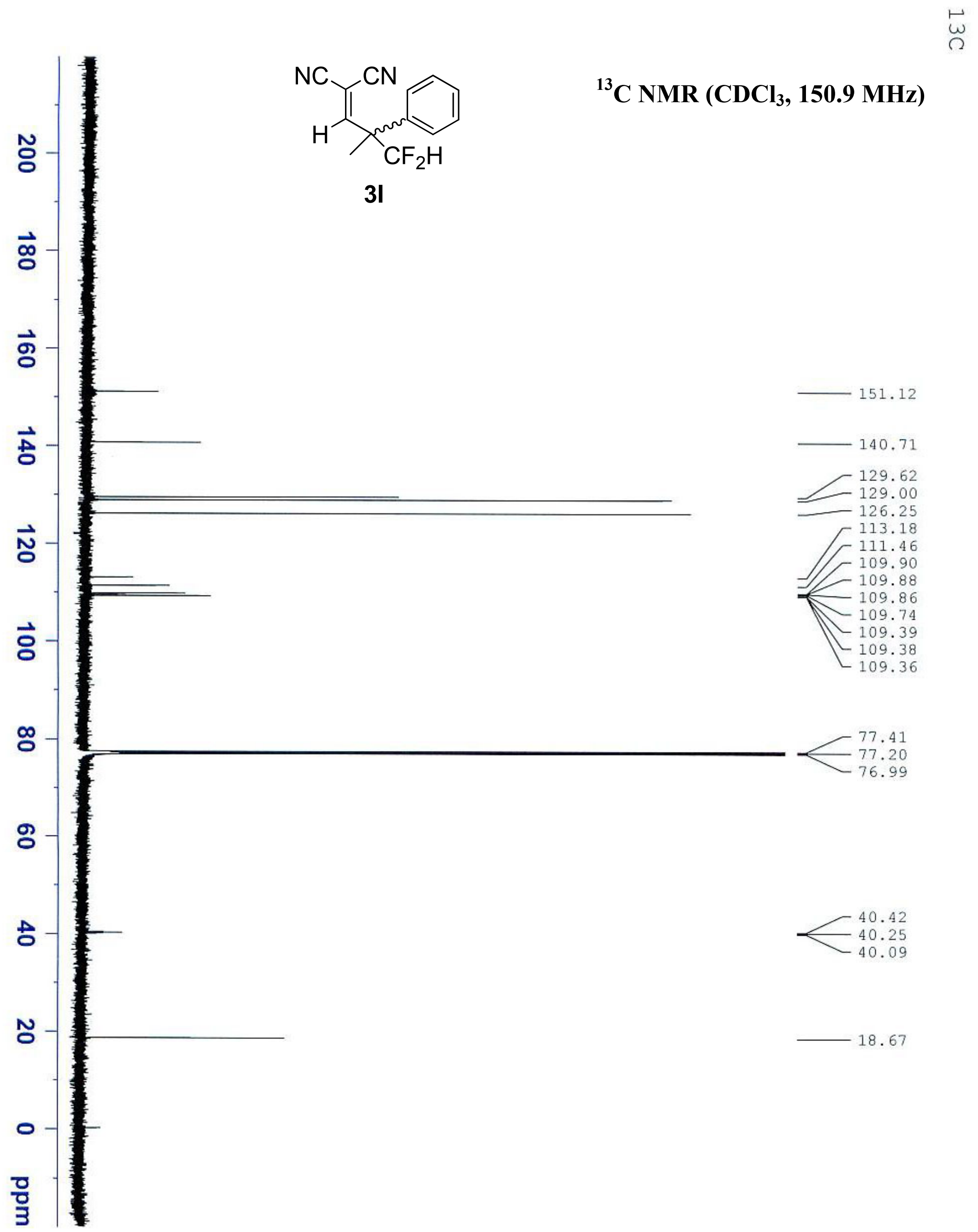
13C
^{13}C NMR (CDCl$_3$, 150.9 MHz)
NC
CN
H
CF$_2$H
3l
151.12
140.71
129.62
129.00
126.25
113.18
111.46
109.90
109.88
109.86
109.74
109.39
109.38
109.36
77.41
77.20
76.99
40.42
40.25
40.09
18.67
200
180
160
140
120
100
80
60
40
20
0
ppm

^{1}H NMR ($CDCl_3$, 300 MHz)

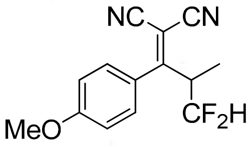

3m

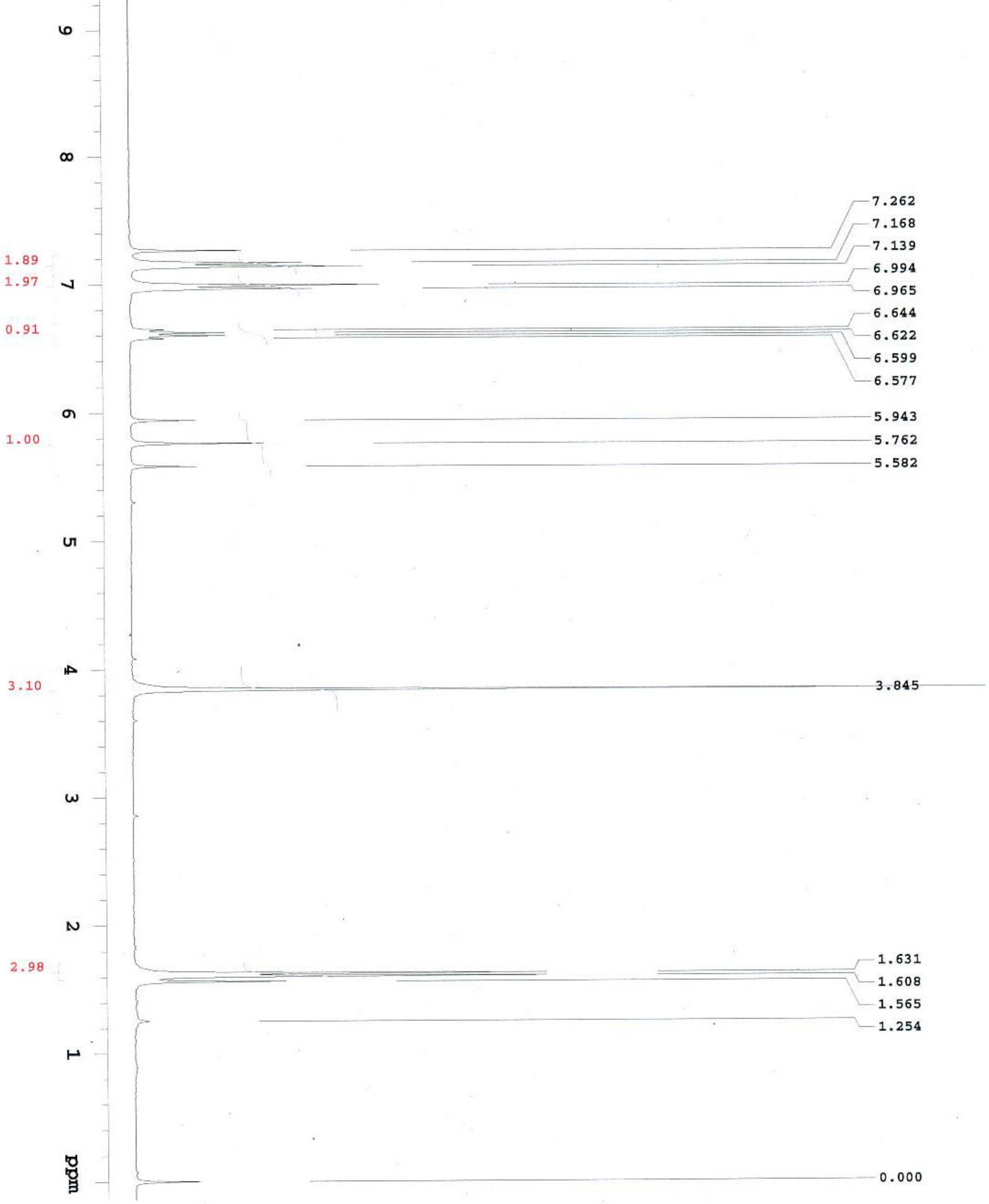

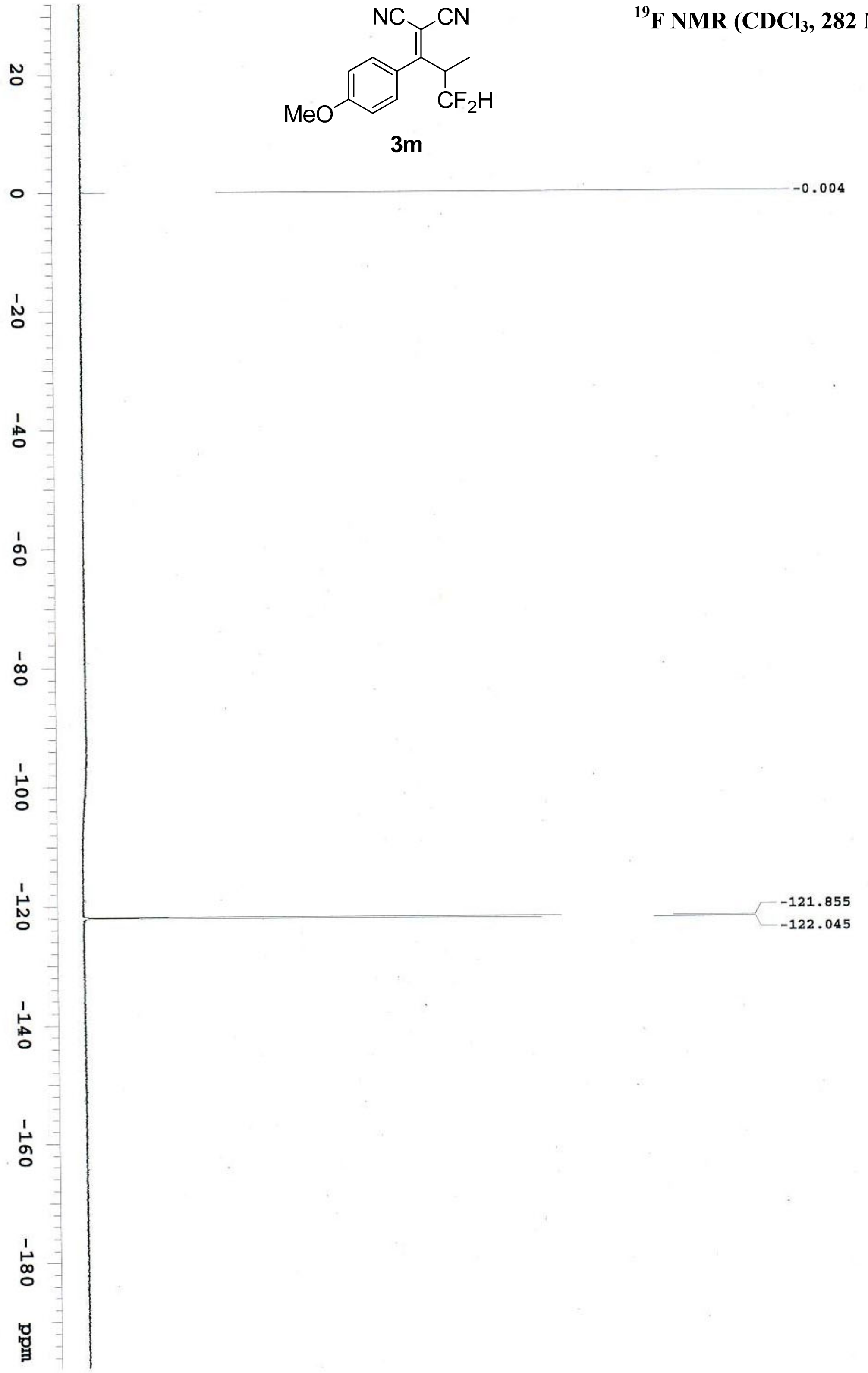

^{19}F NMR (CDCl$_3$, 282 MHz)

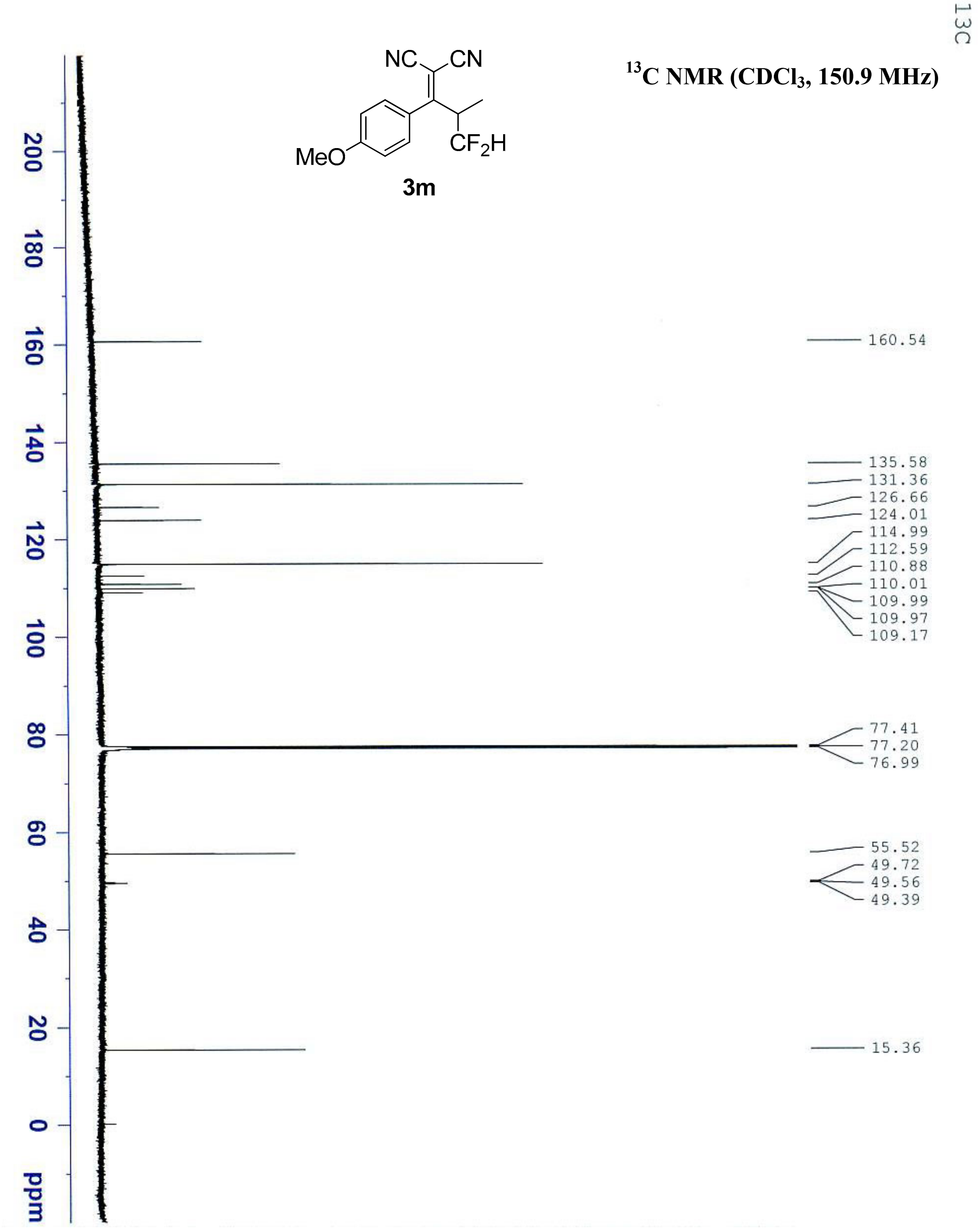
13C
^{13}C NMR (CDCl$_3$, 150.9 MHz)
NC
CN
MeO
CF$_2$H
3m
160.54
135.58
131.36
126.66
124.01
114.99
112.59
110.88
110.01
109.99
109.97
109.17
77.41
77.20
76.99
55.52
49.72
49.56
49.39
15.36
200
180
160
140
120
100
80
60
40
20
0
ppm

^{1}H NMR ($CDCl_3$, 300 MHz)

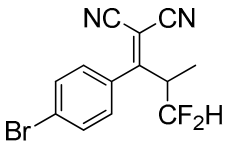

3n

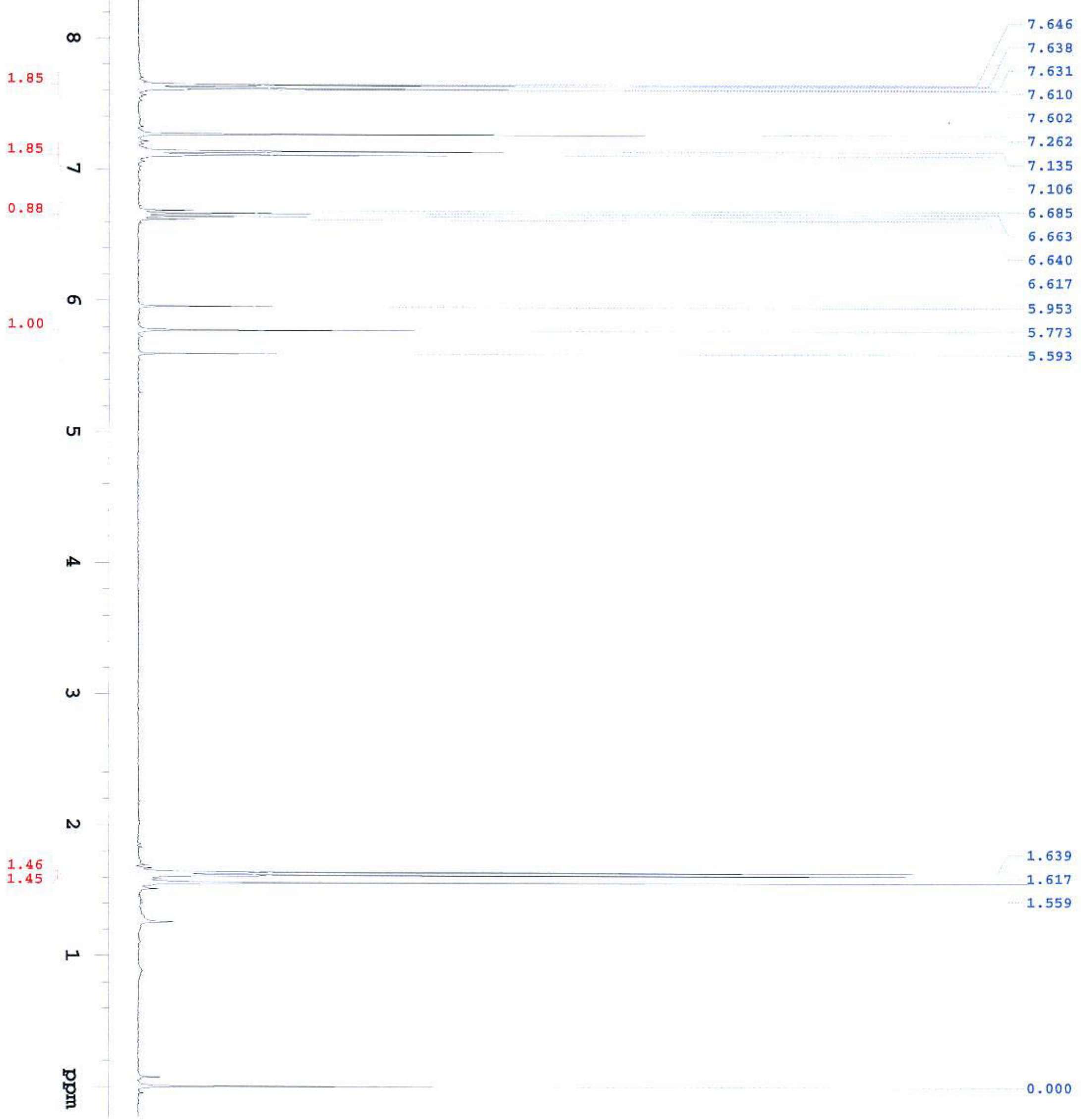

^{19}F NMR ($CDCl_3$, 282 MHz)

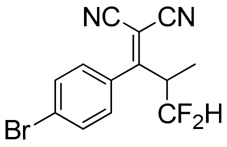

3n

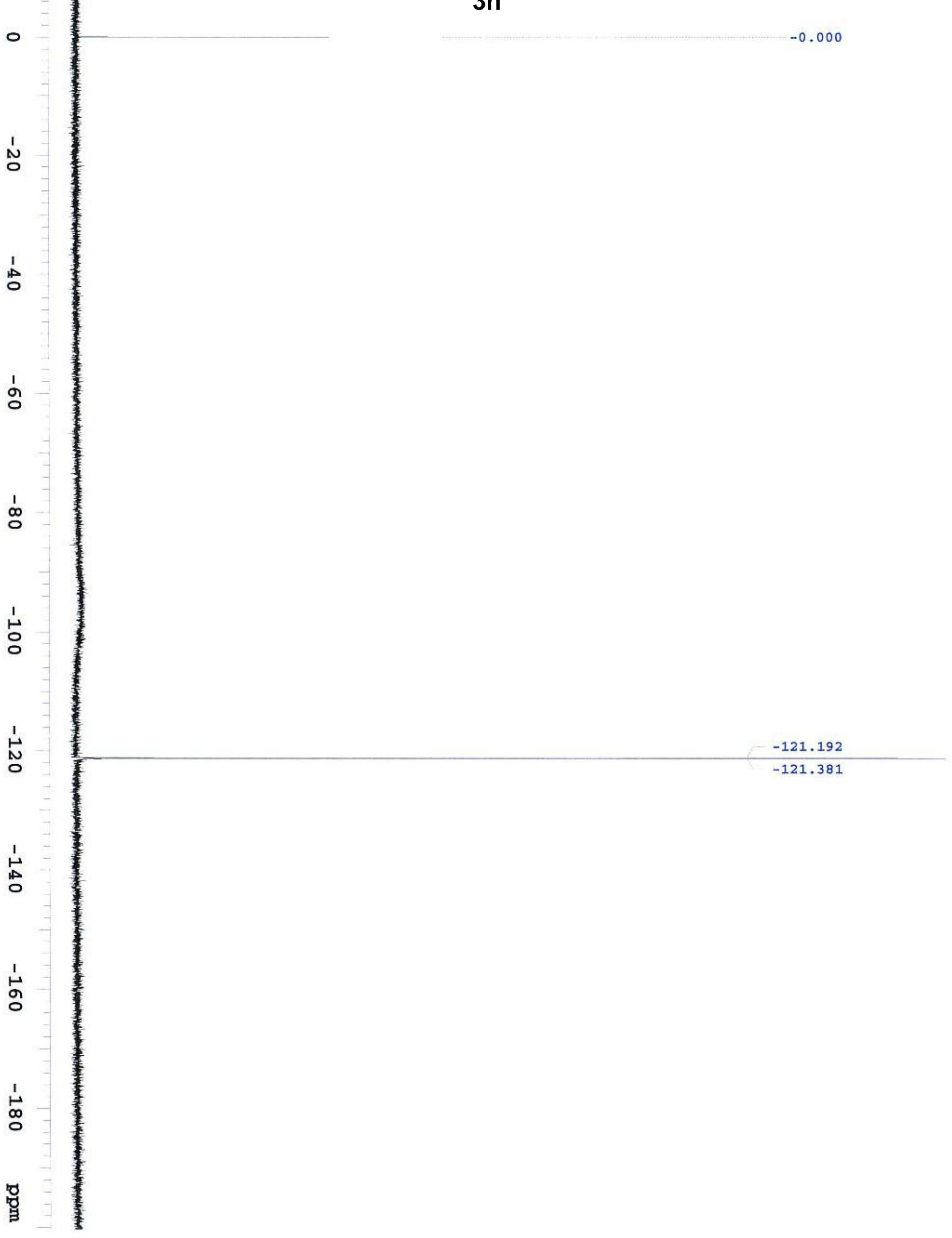

^{13}C NMR (CDCl$_3$, 150.9 MHz)

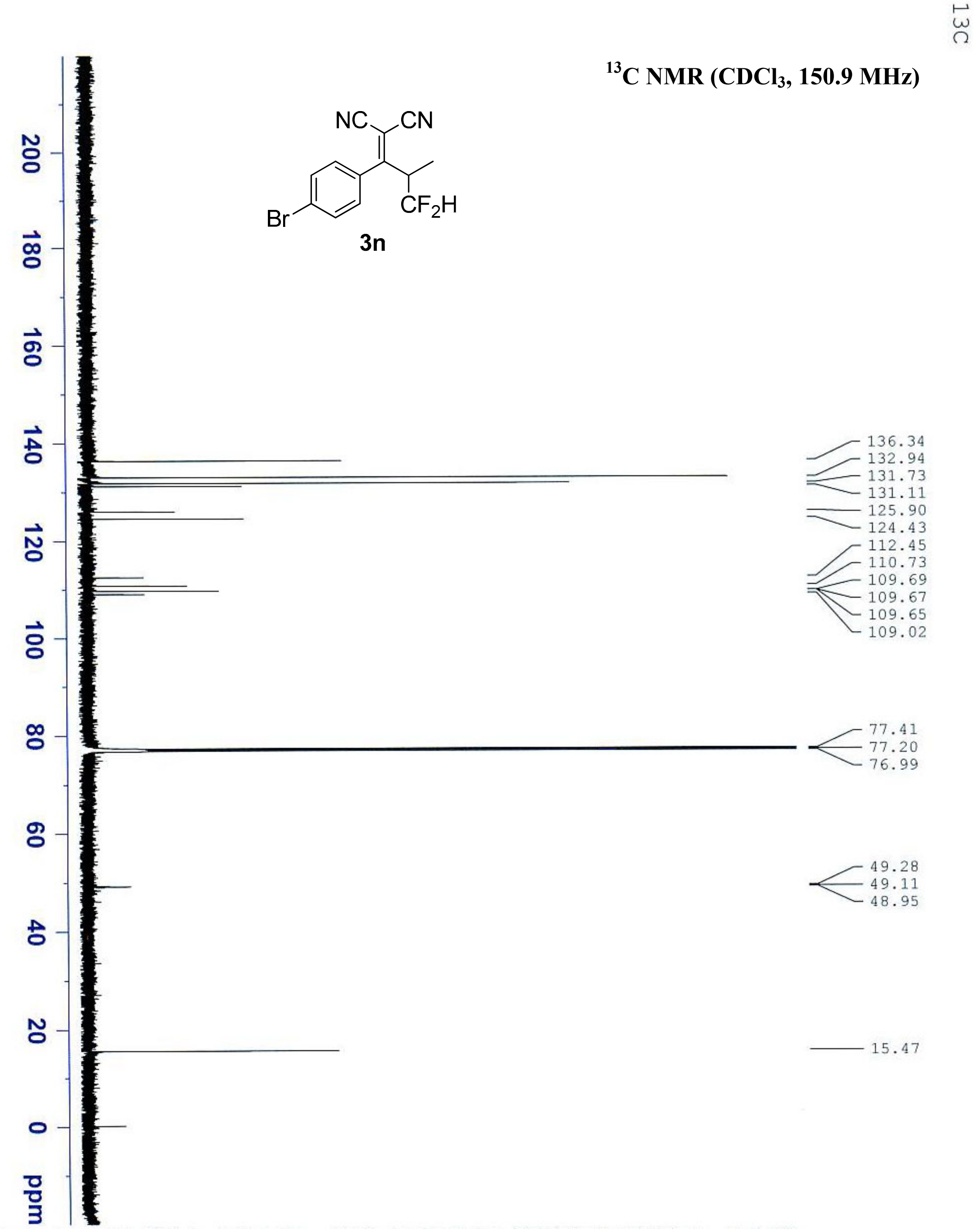

^{1}H NMR ($CDCl_3$, 300 MHz)

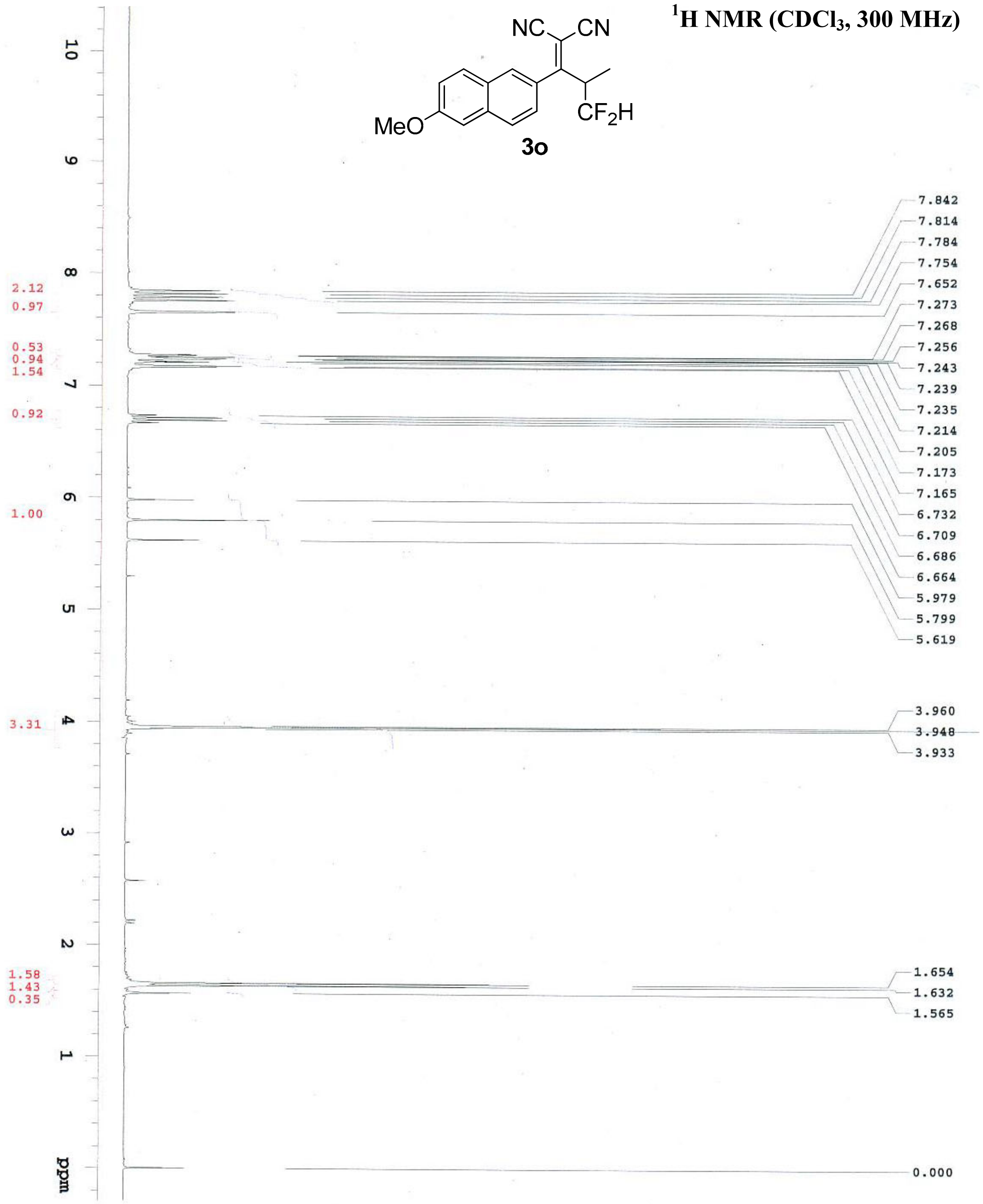

^{19}F NMR ($CDCl_3$, 282 MHz)

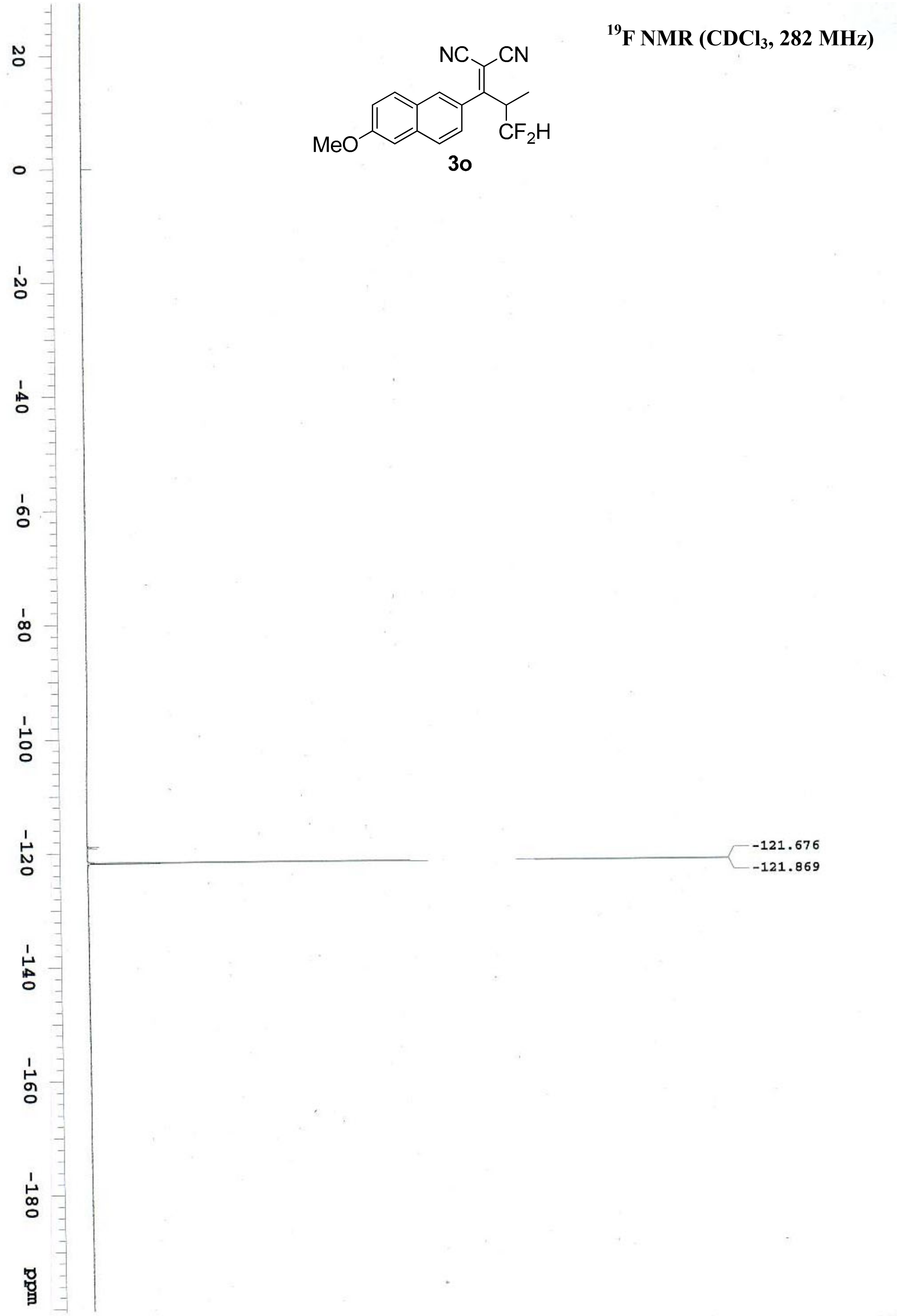

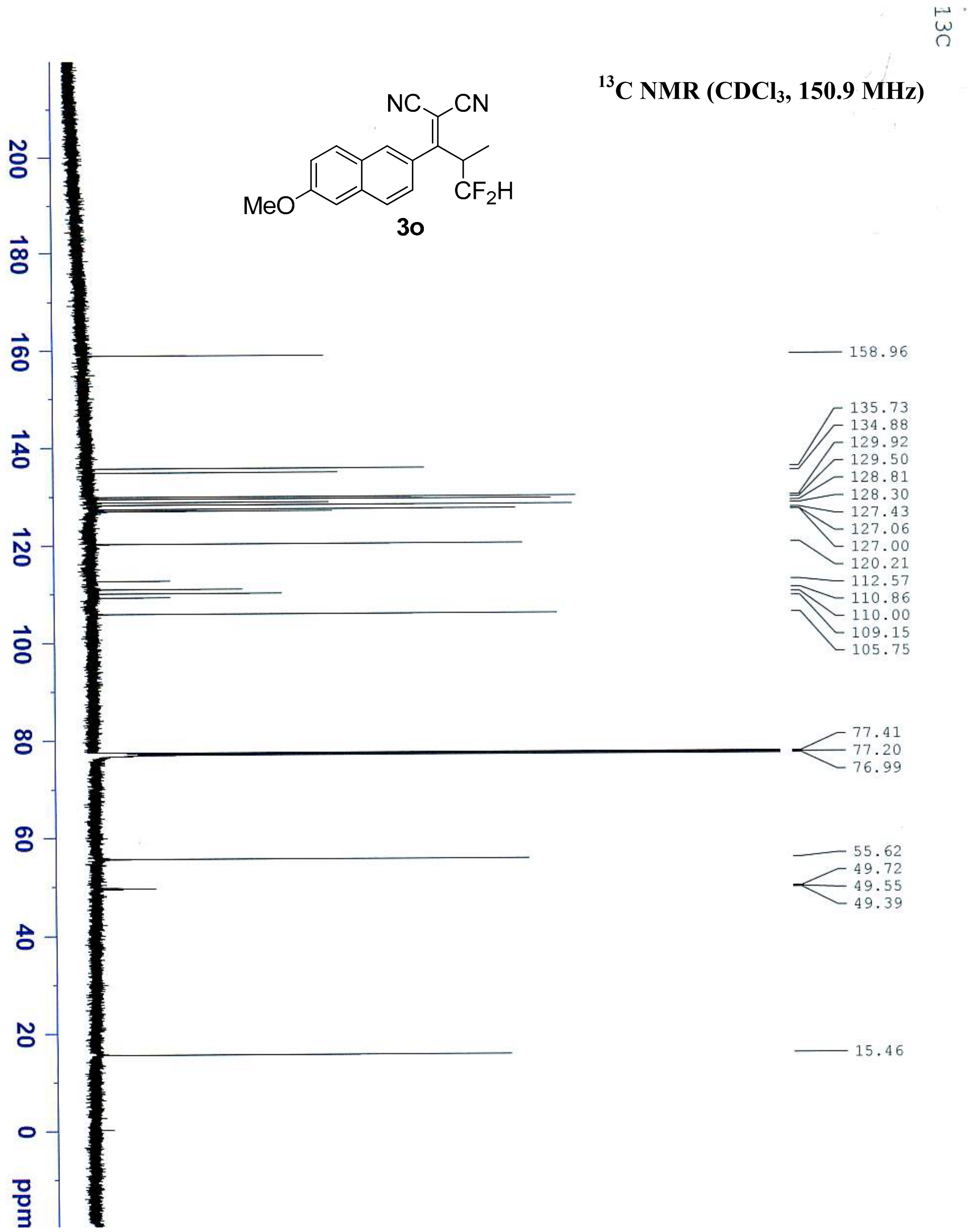

^{13}C NMR ($CDCl_3$, 150.9 MHz)

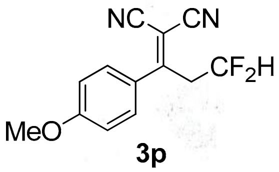

3p

^{1}H NMR ($CDCl_3$, 300 MHz)

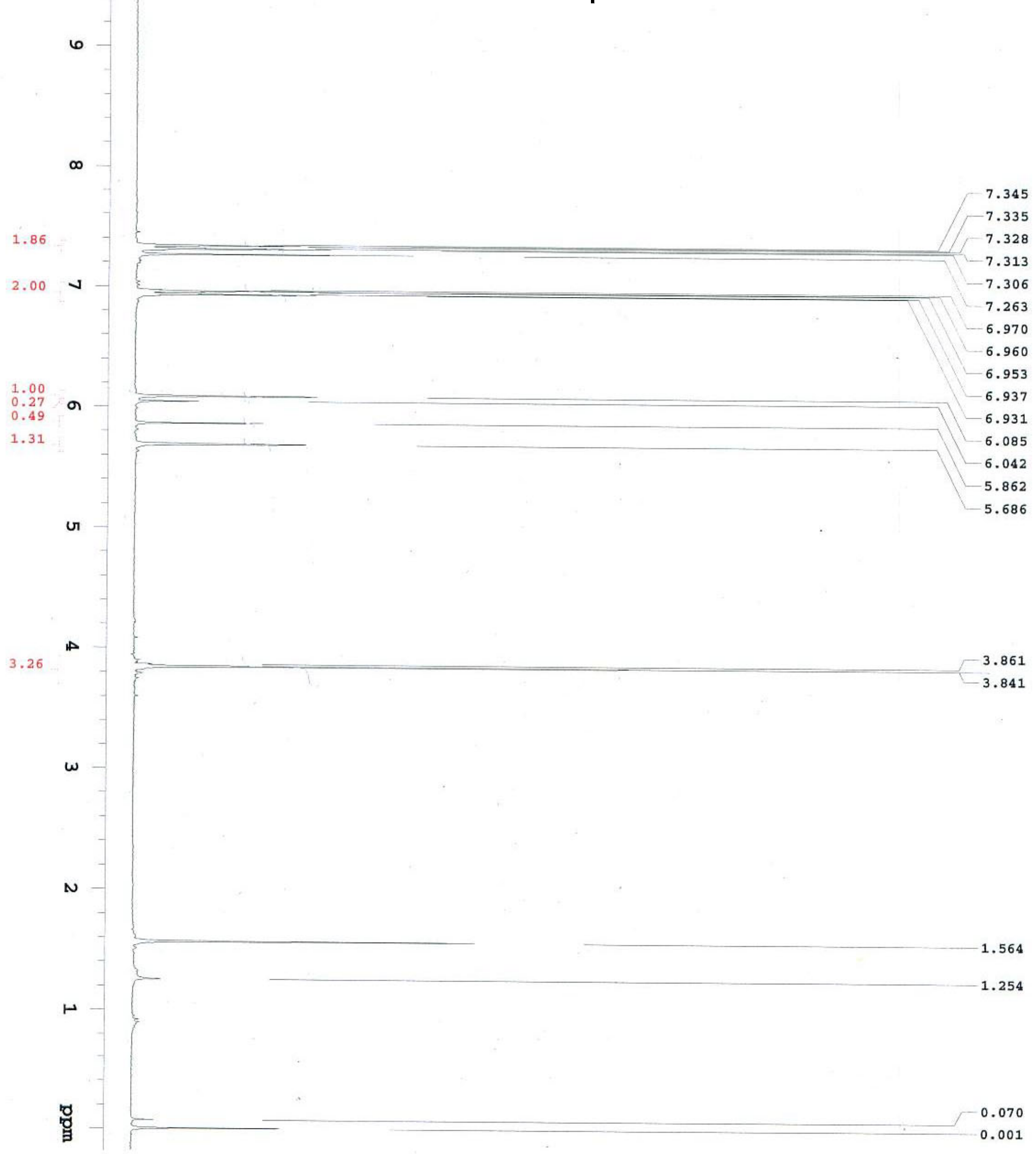

^{19}F NMR ($CDCl_3$, 282 MHz)

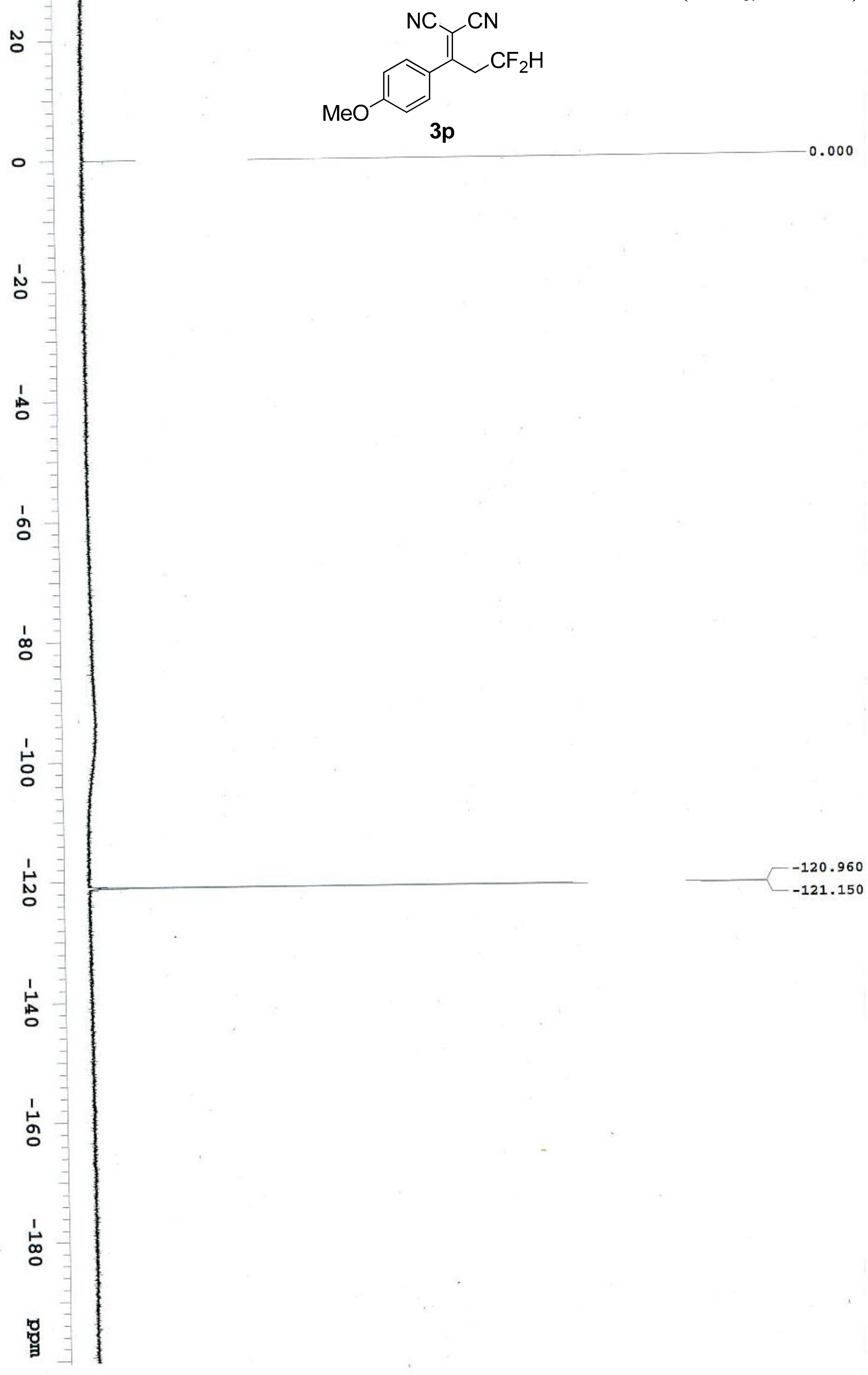

^{13}C NMR ($CDCl_3$, 150.9 MHz)

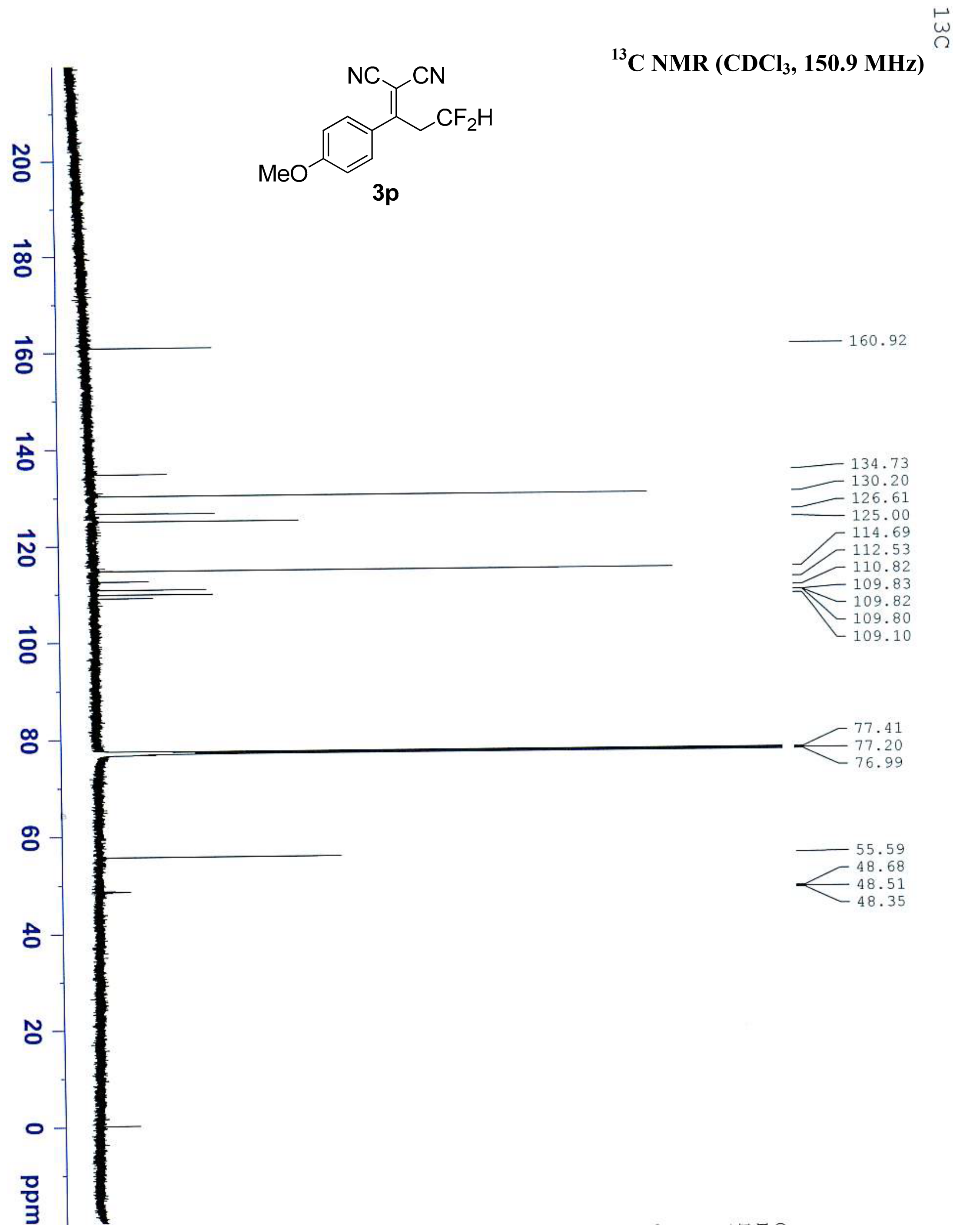

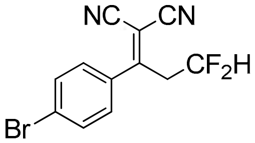

3q

^{1}H NMR ($CDCl_3$, 300 MHz)

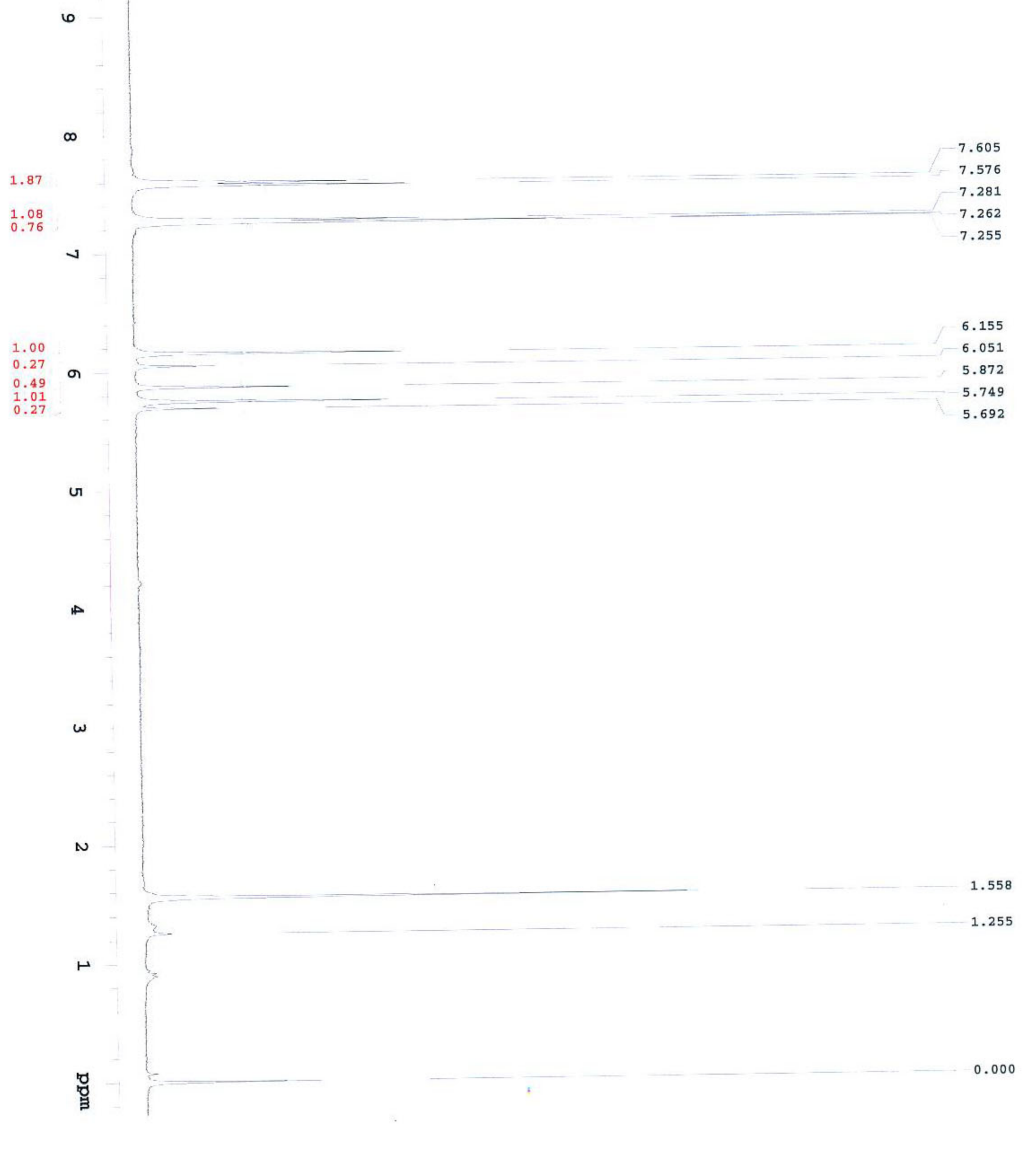

^{19}F NMR ($CDCl_3$, 282 MHz)

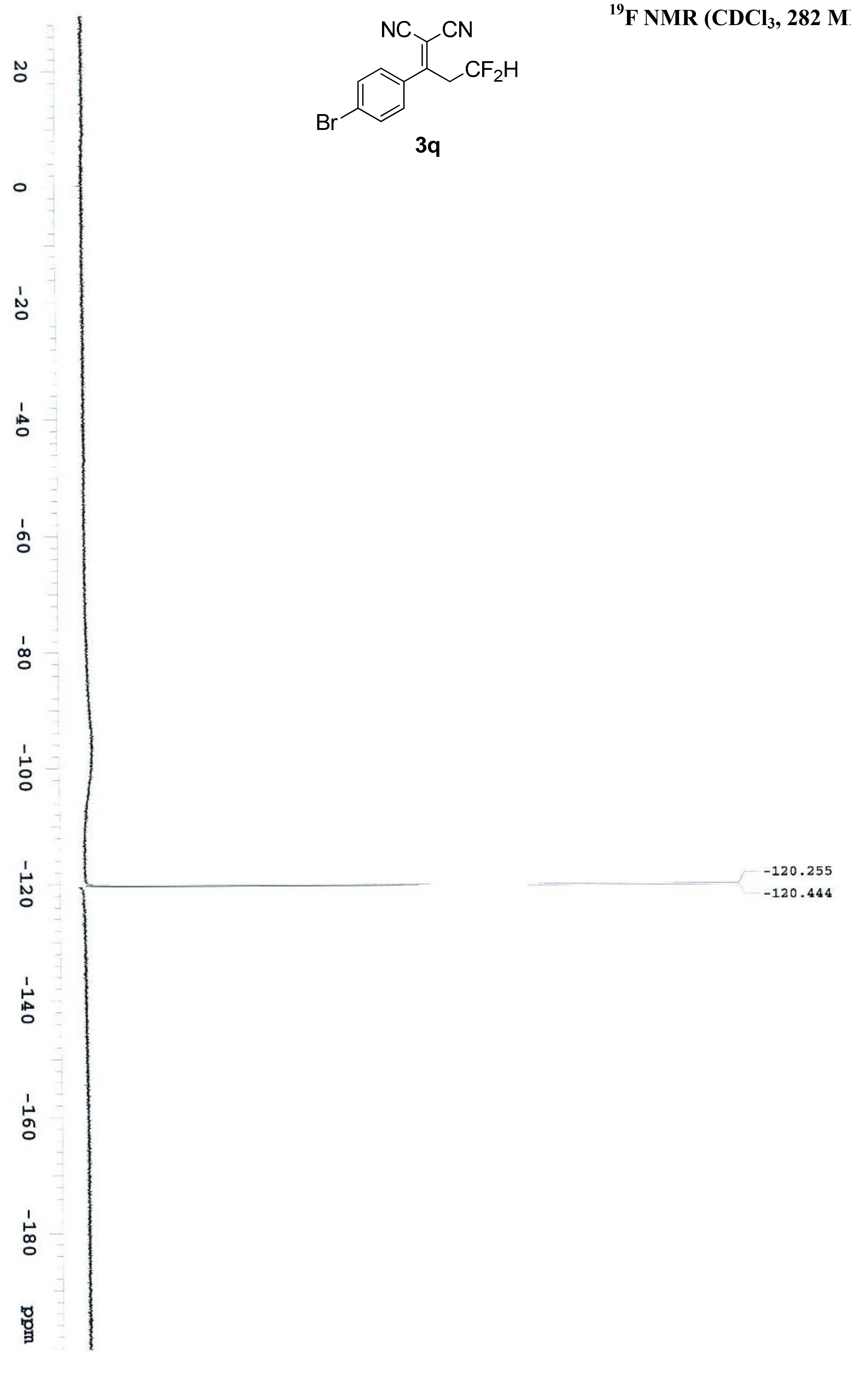

3q

^{13}C NMR ($CDCl_3$, 150.9 MHz)

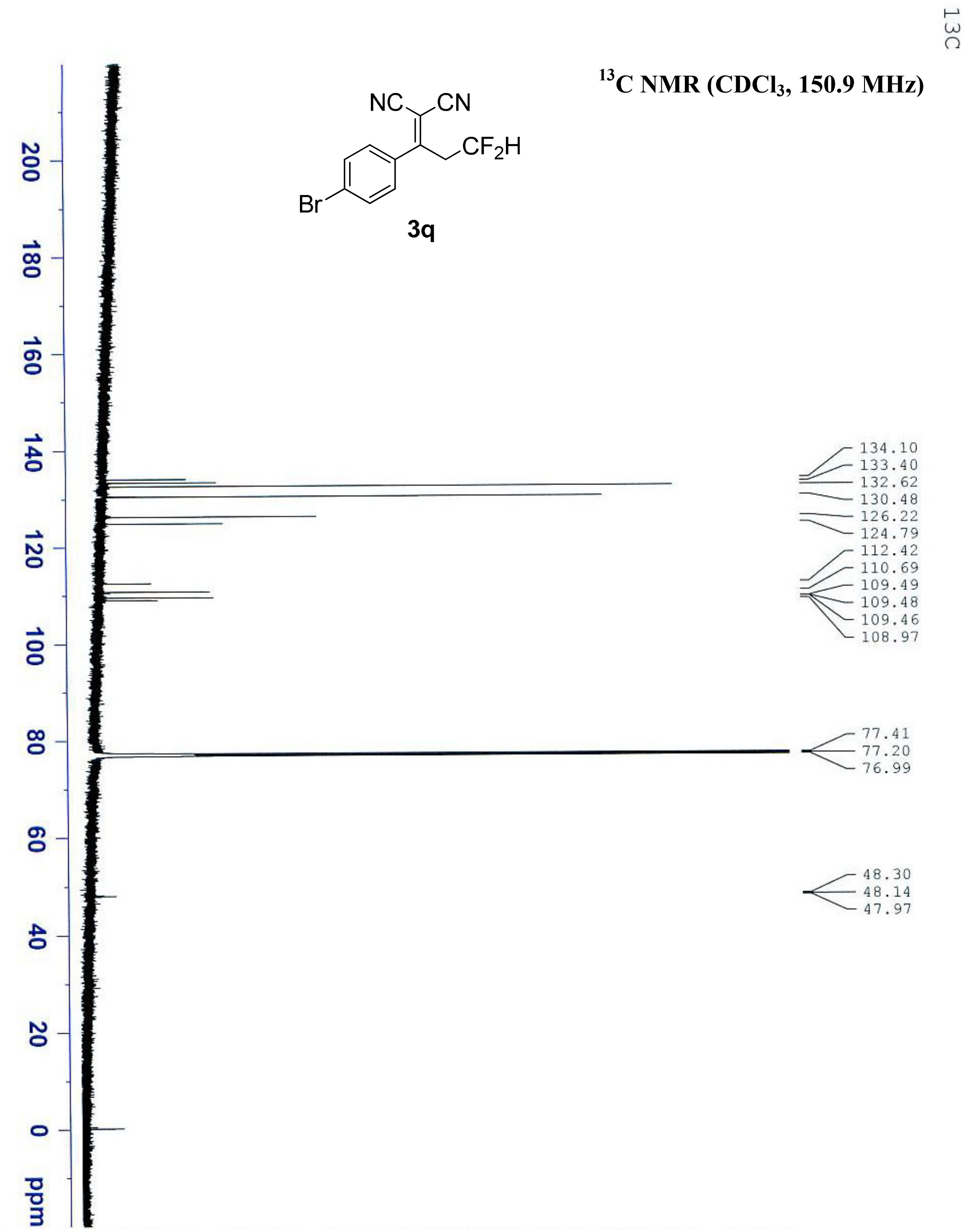

^{1}H NMR ($CDCl_3$, 300 MHz)

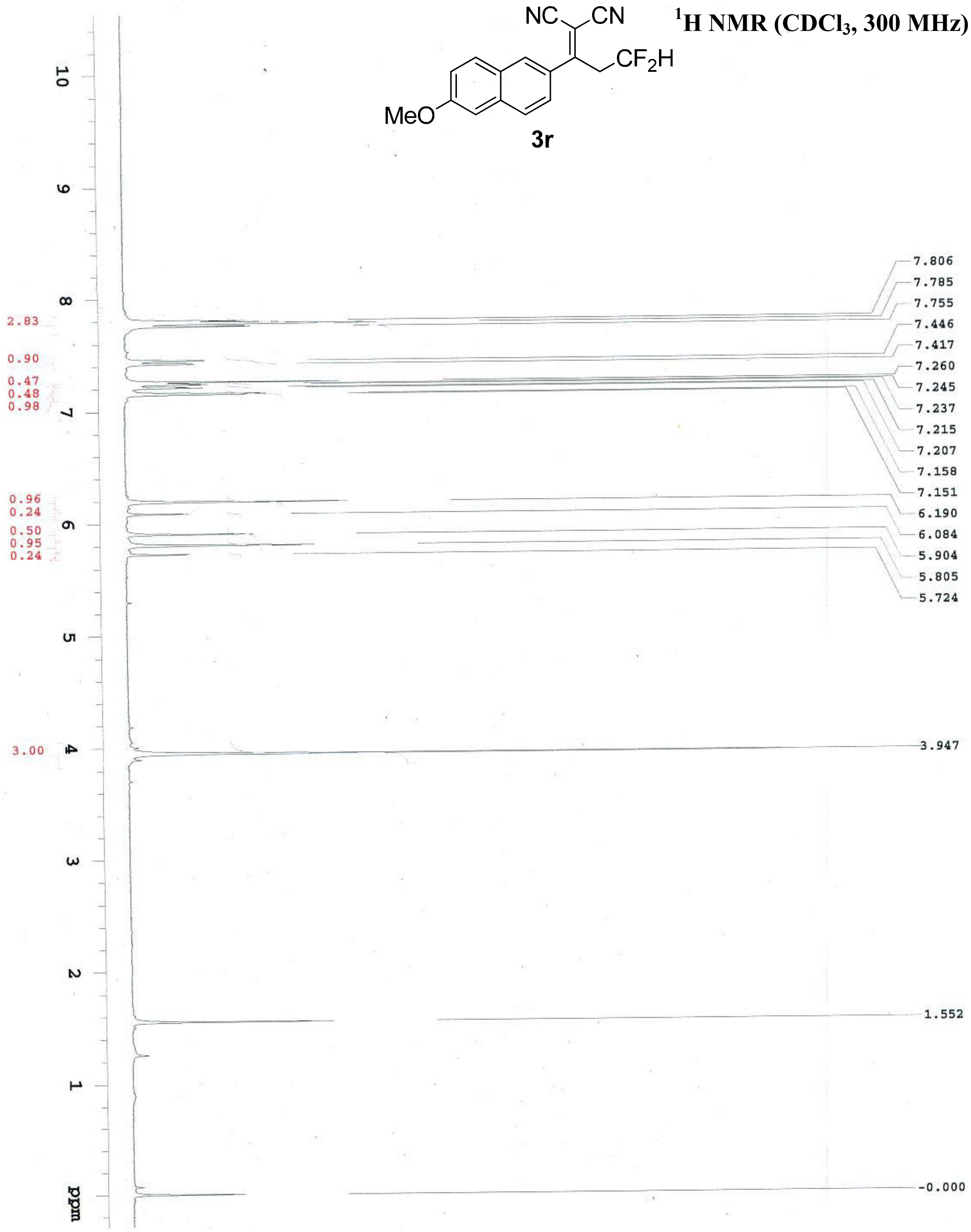

^{19}F NMR ($CDCl_3$, 282 MHz)

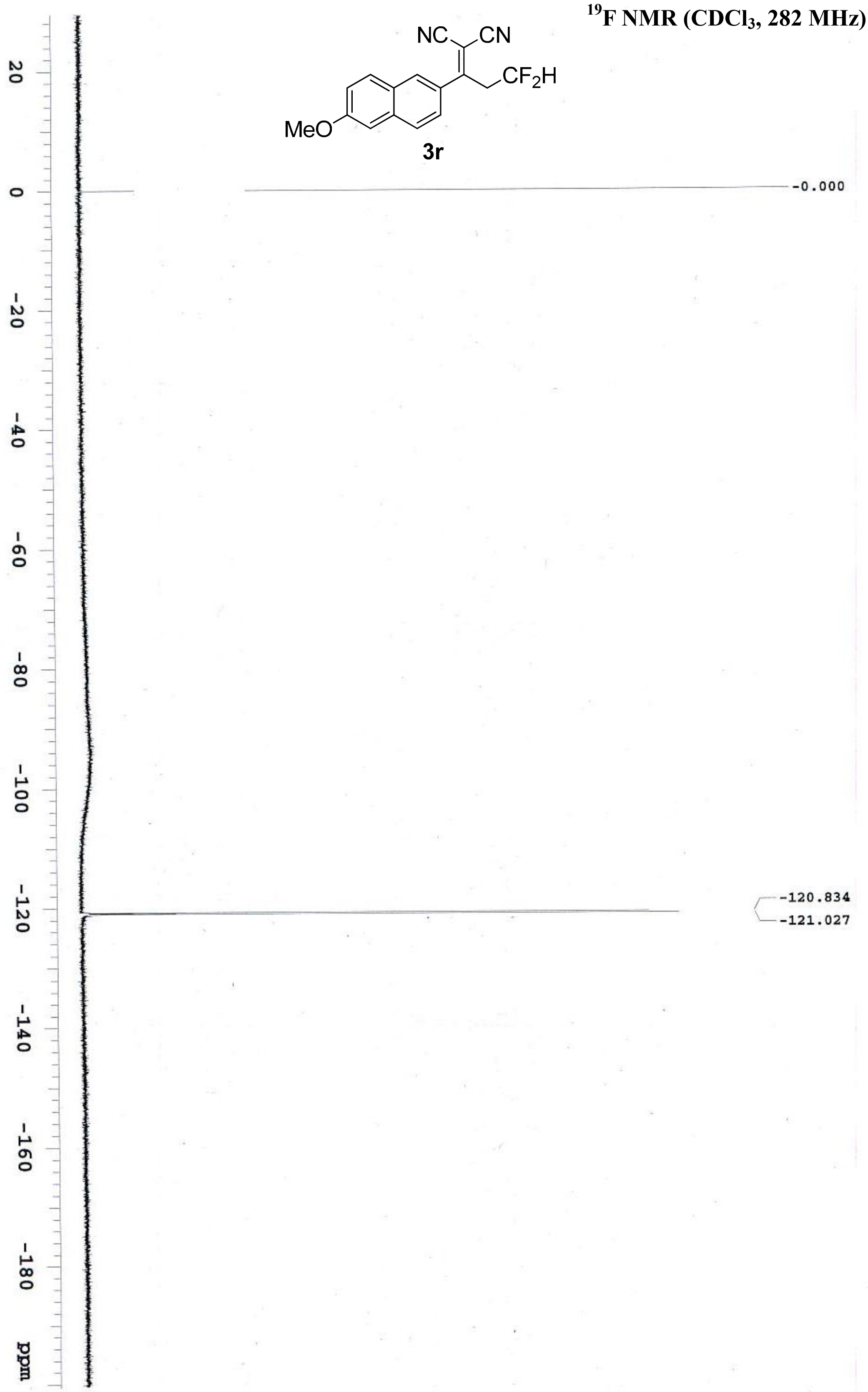

^{13}C NMR ($CDCl_3$, 150.9 MHz)

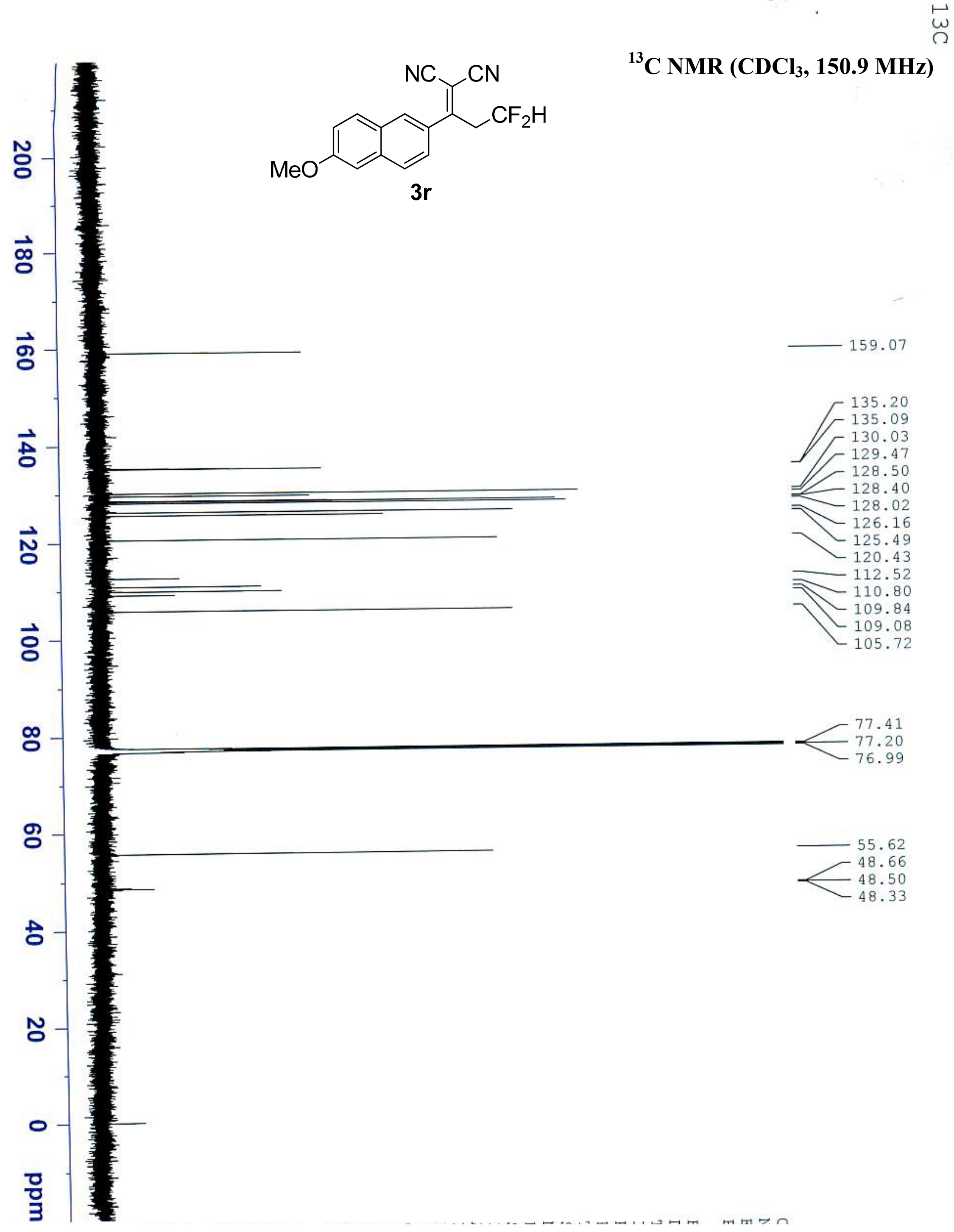

^{1}H NMR ($CDCl_3$, 300 MHz)

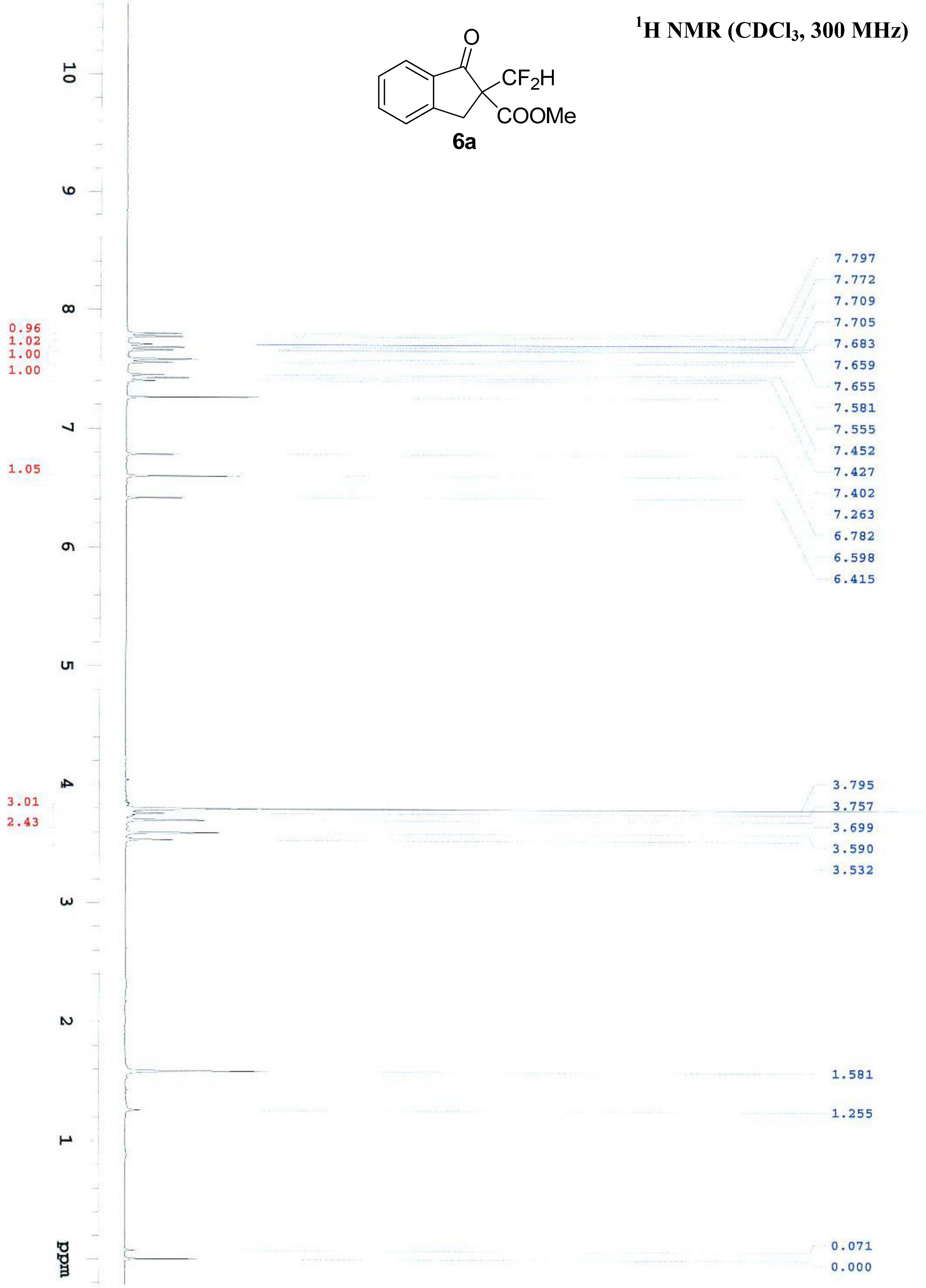

6a

^{19}F NMR ($CDCl_3$, 282 MHz)

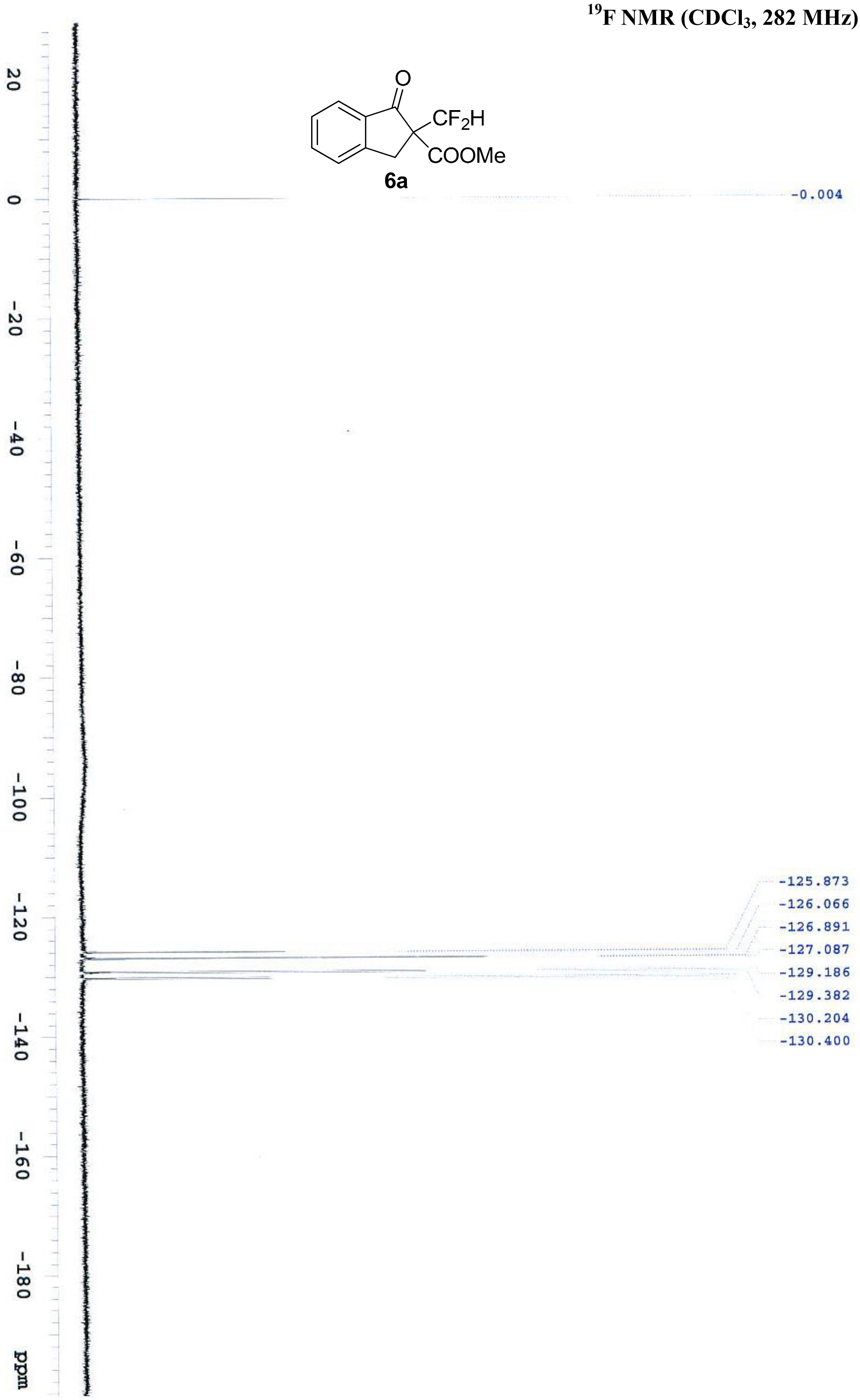

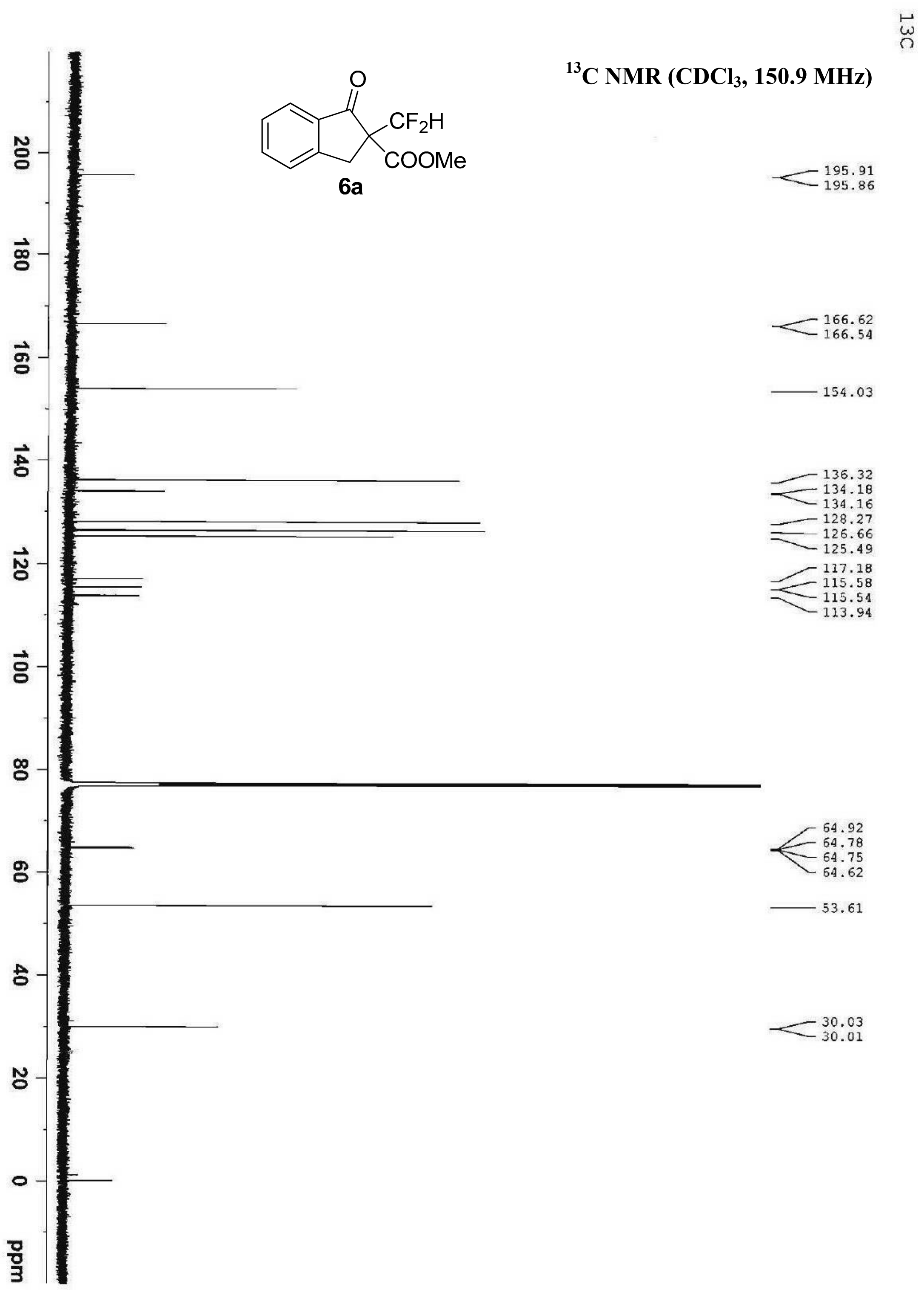

13C
13C NMR (CDCl3, 150.9 MHz)
CF2H
COOMe
6a
195.91
195.86
166.62
166.54
154.03
136.32
134.18
134.16
128.27
126.66
125.49
117.18
115.58
115.54
113.94
64.92
64.78
64.75
64.62
53.61
30.03
30.01
200
180
160
140
120
100
80
60
40
20
0
ppm

^{1}H NMR ($CDCl_3$, 300 MHz)

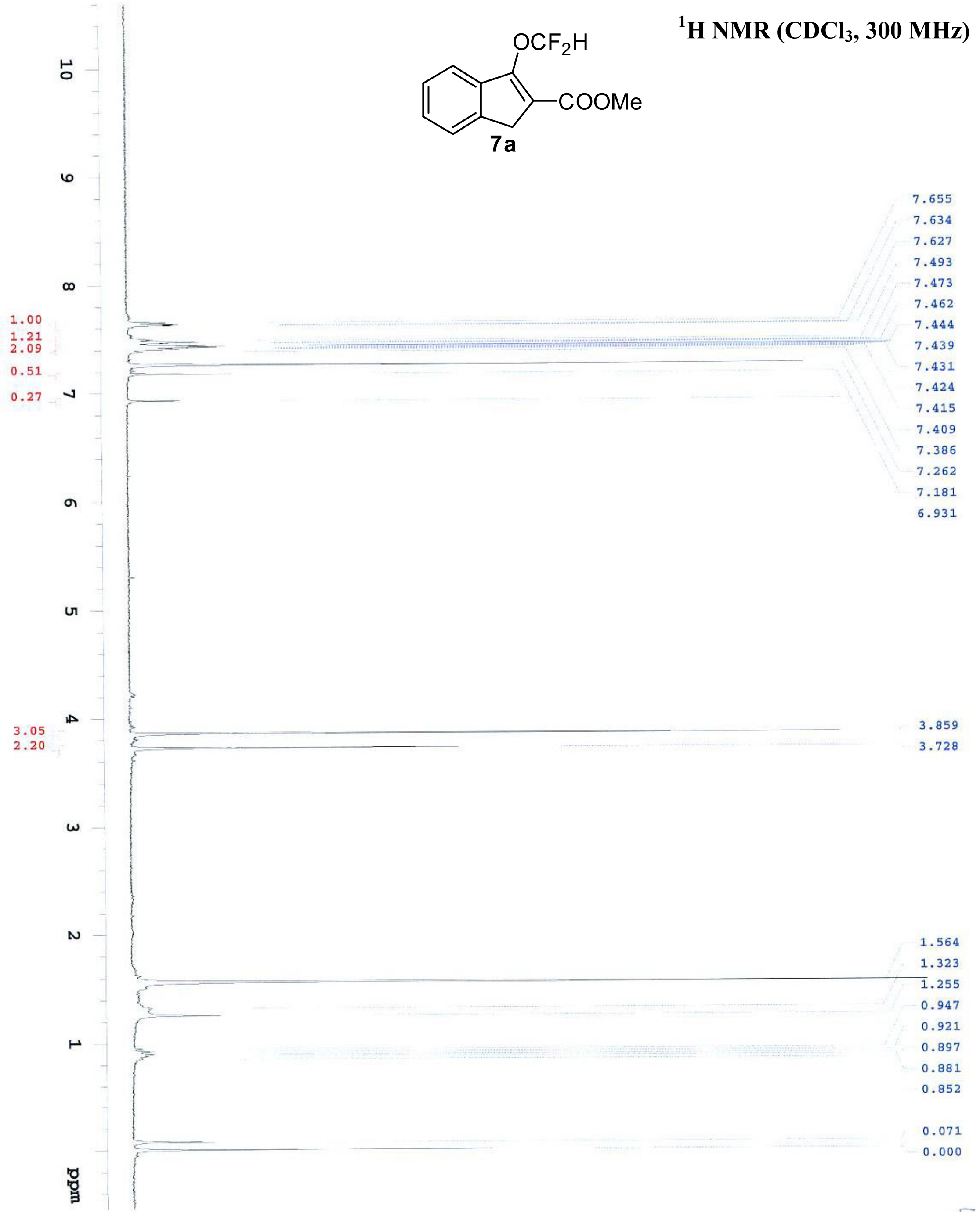

^{19}F NMR ($CDCl_3$, 282 MHz)

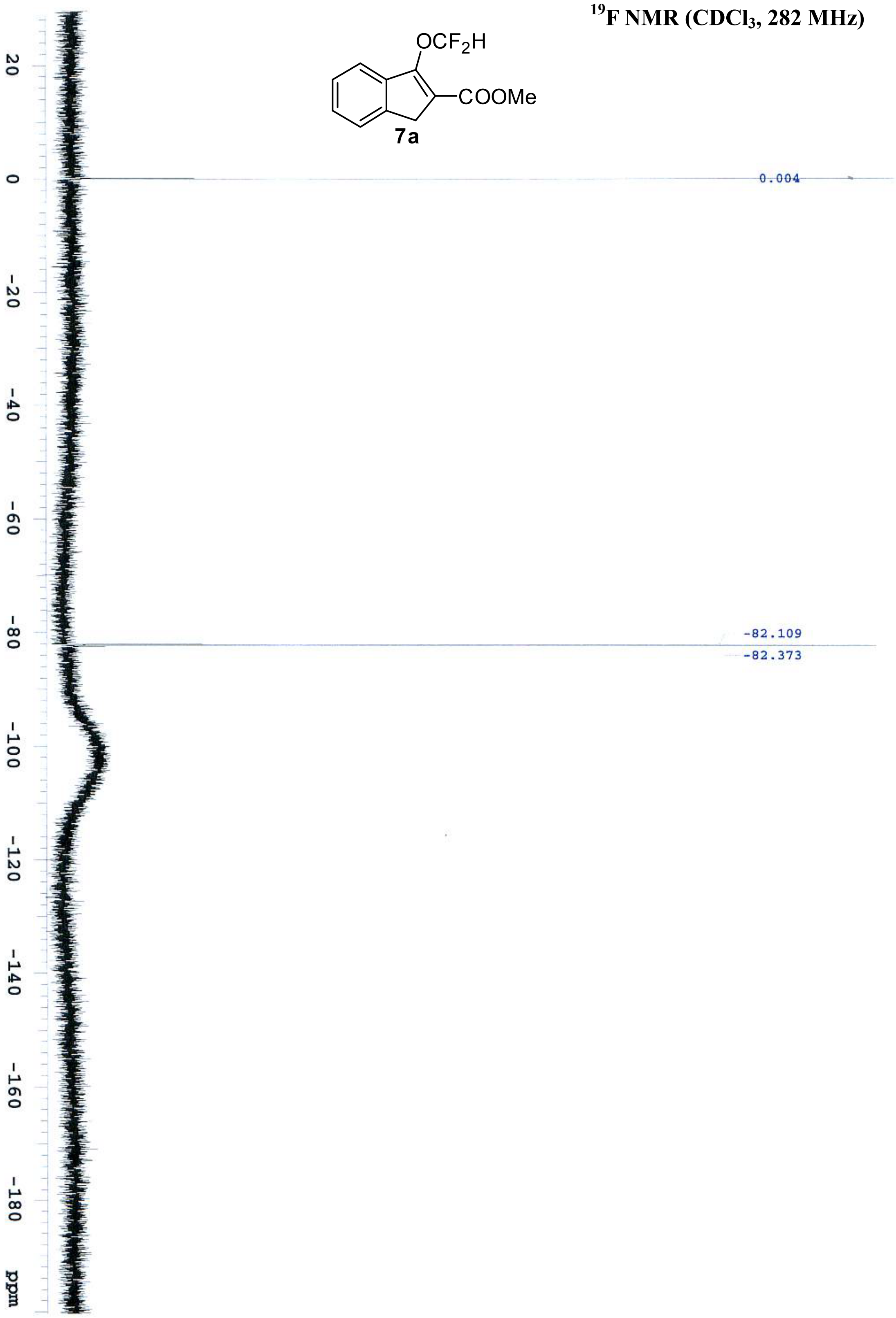

^{13}C NMR ($CDCl_3$, 150.9 MHz)

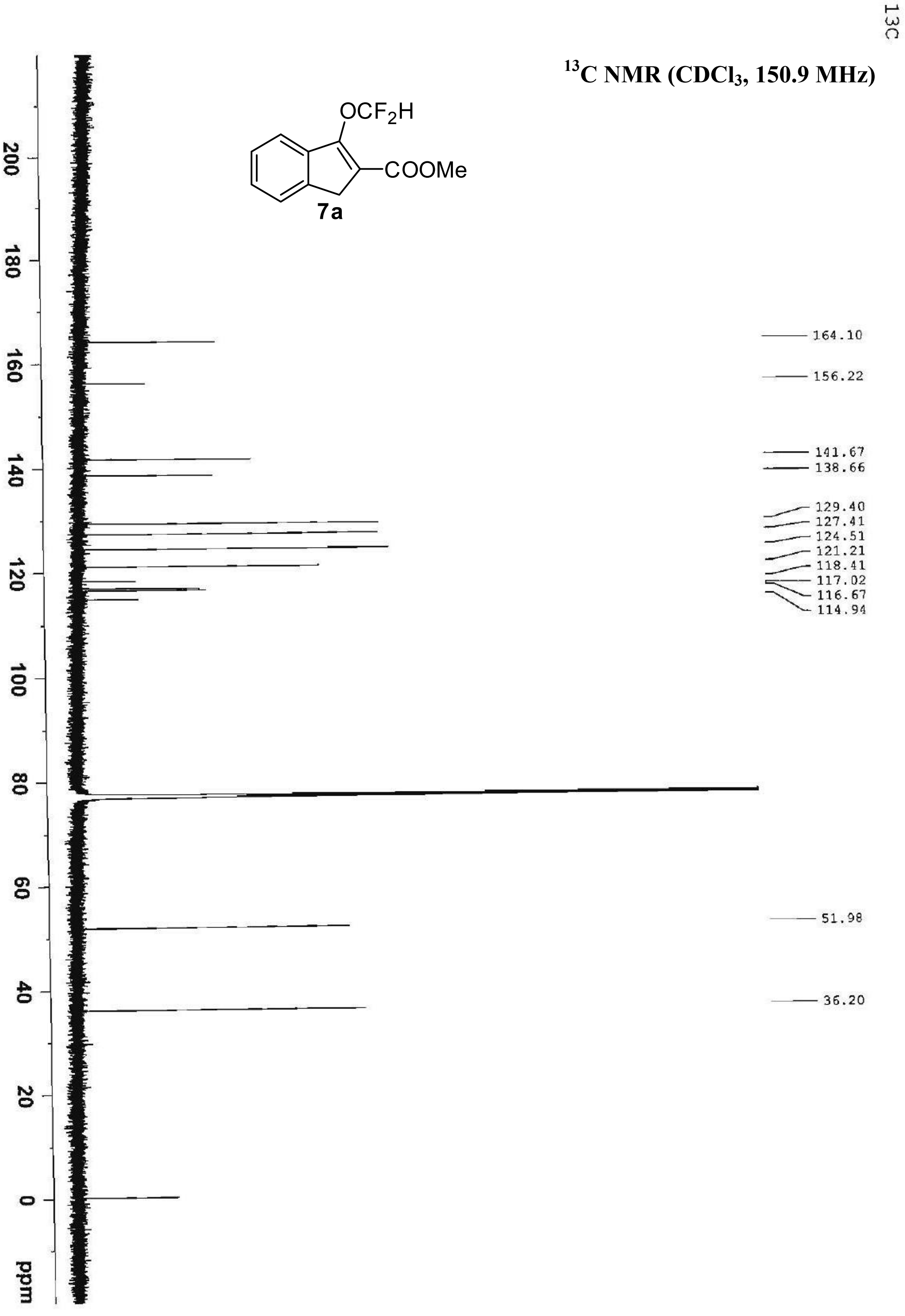

^{1}H NMR ($CDCl_3$, 300 MHz)

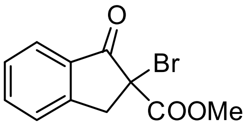

8a

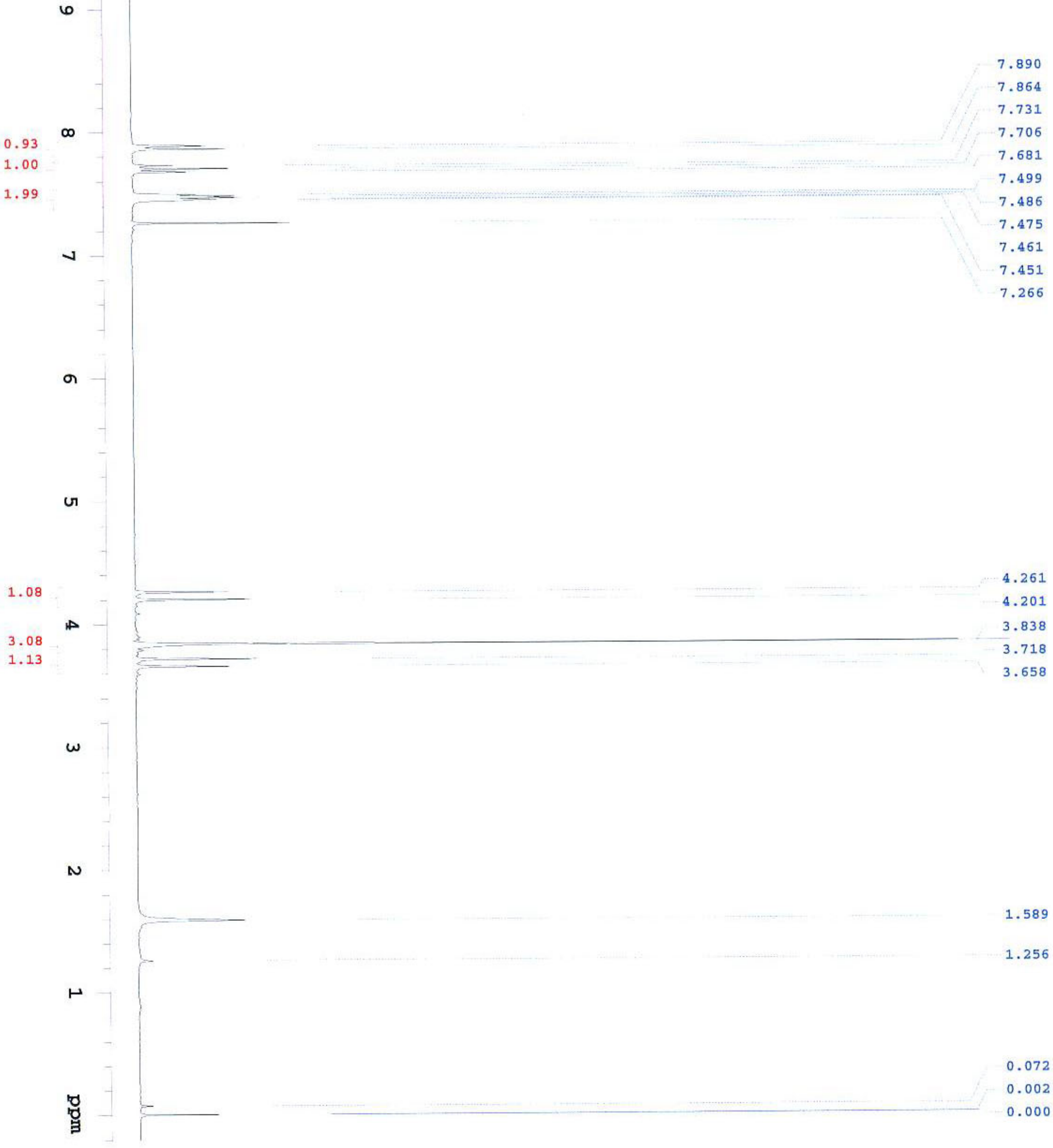

8a

^{13}C NMR ($CDCl_3$, 150.9 MHz)

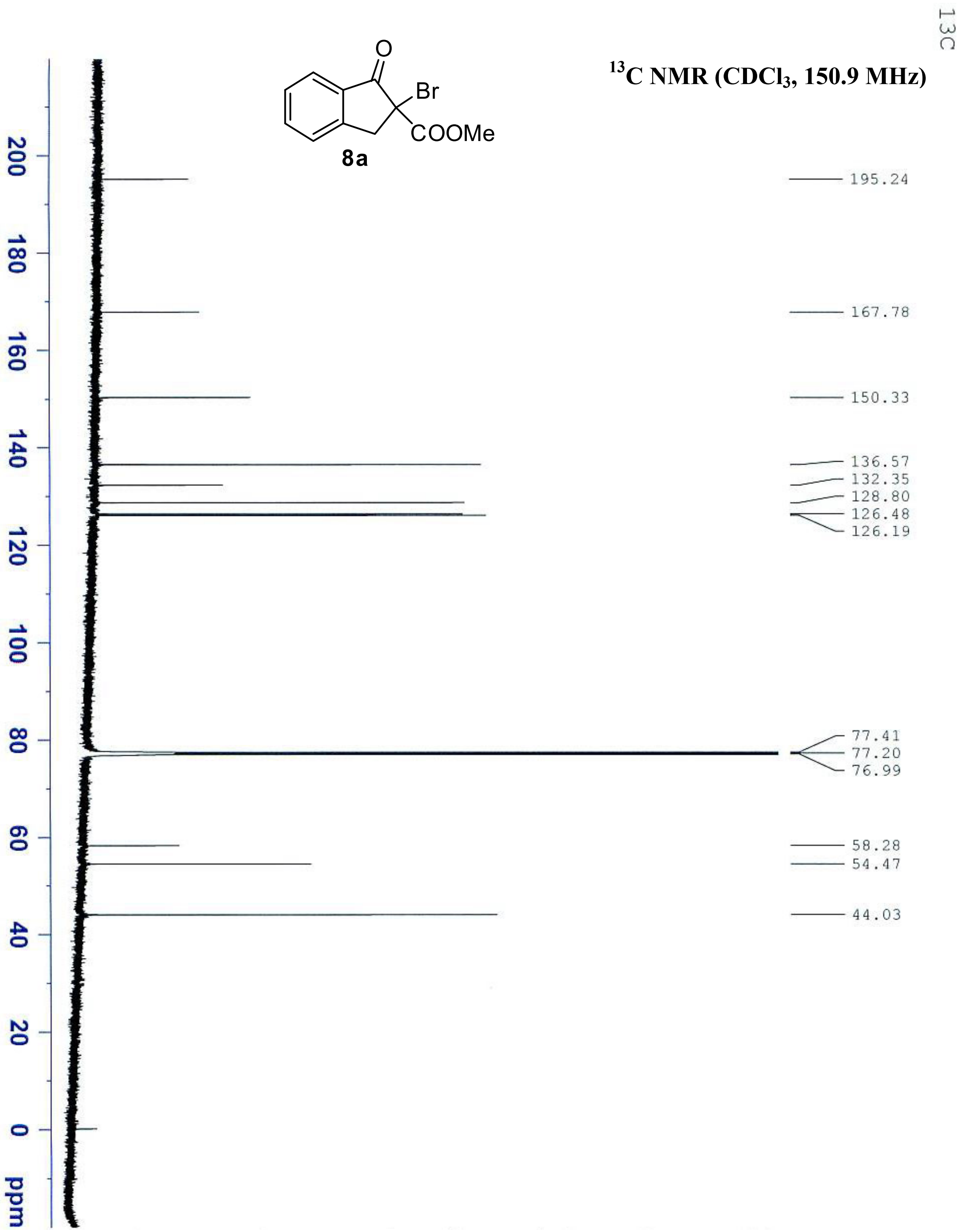

^{1}H NMR ($CDCl_3$, 300 MHz)

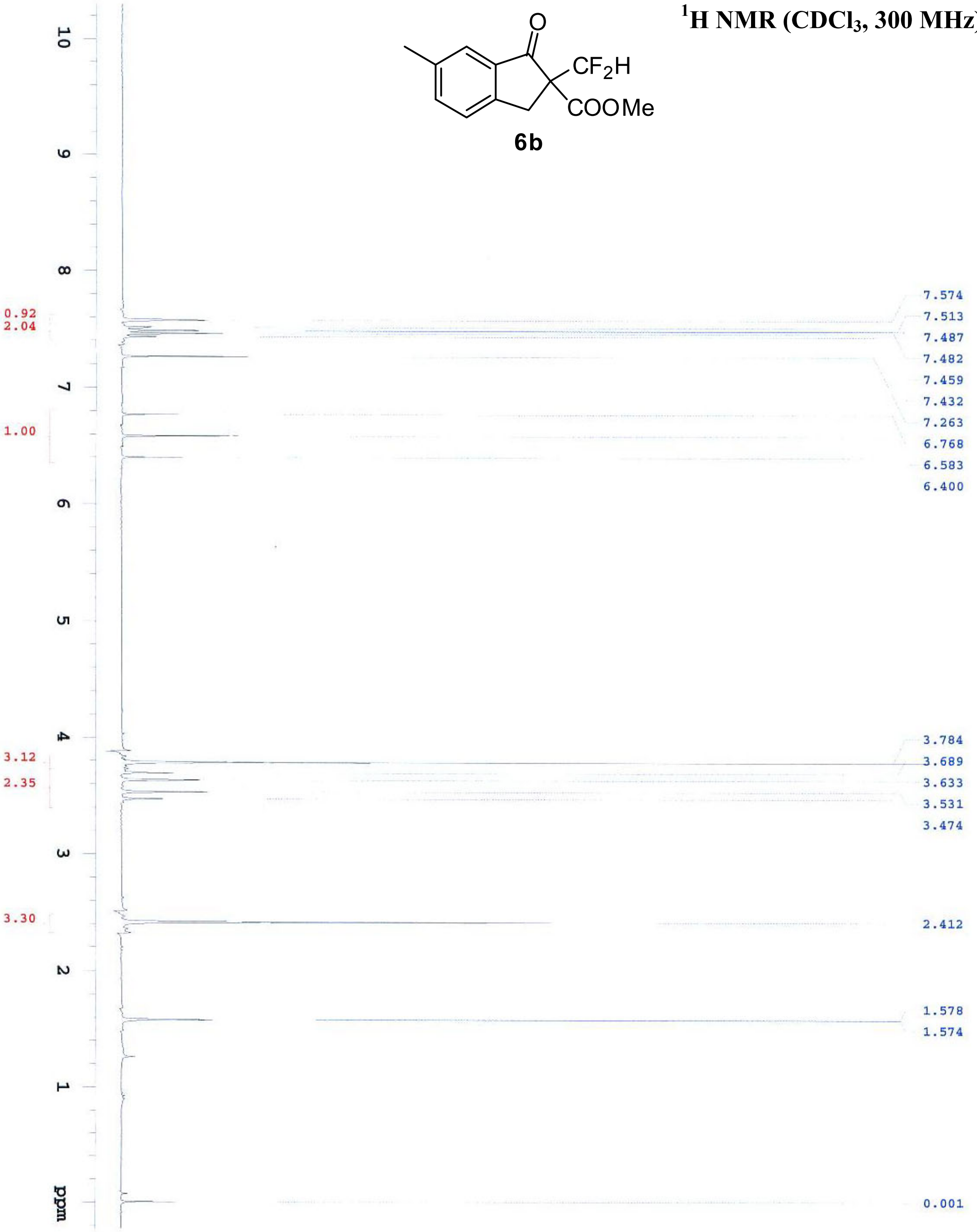

^{19}F NMR ($CDCl_3$, 282 MHz)

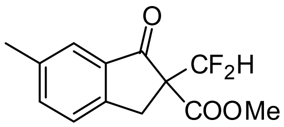

6b

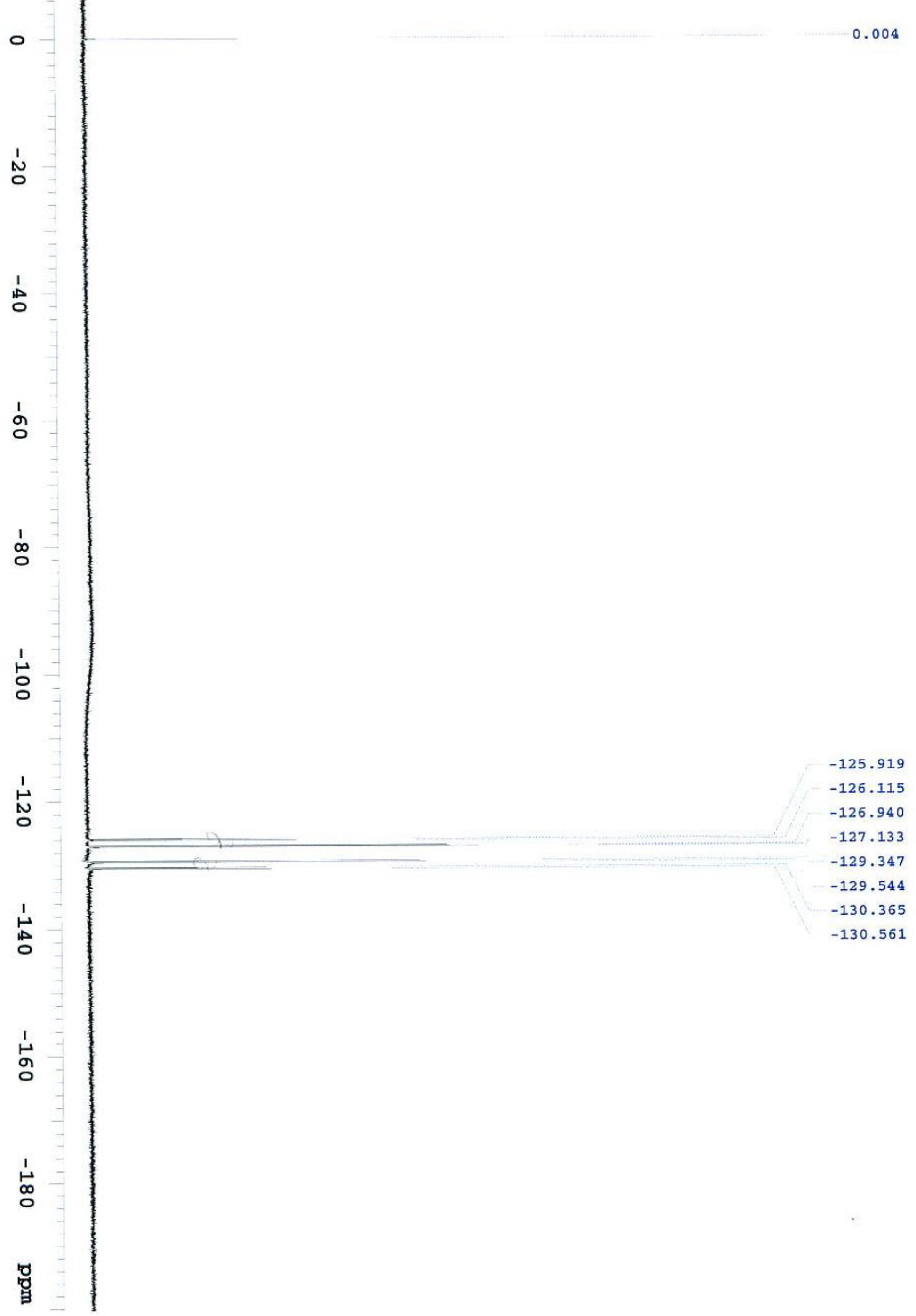

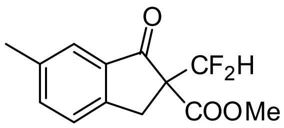

6b

^{13}C NMR ($CDCl_3$, 150.9 MHz)

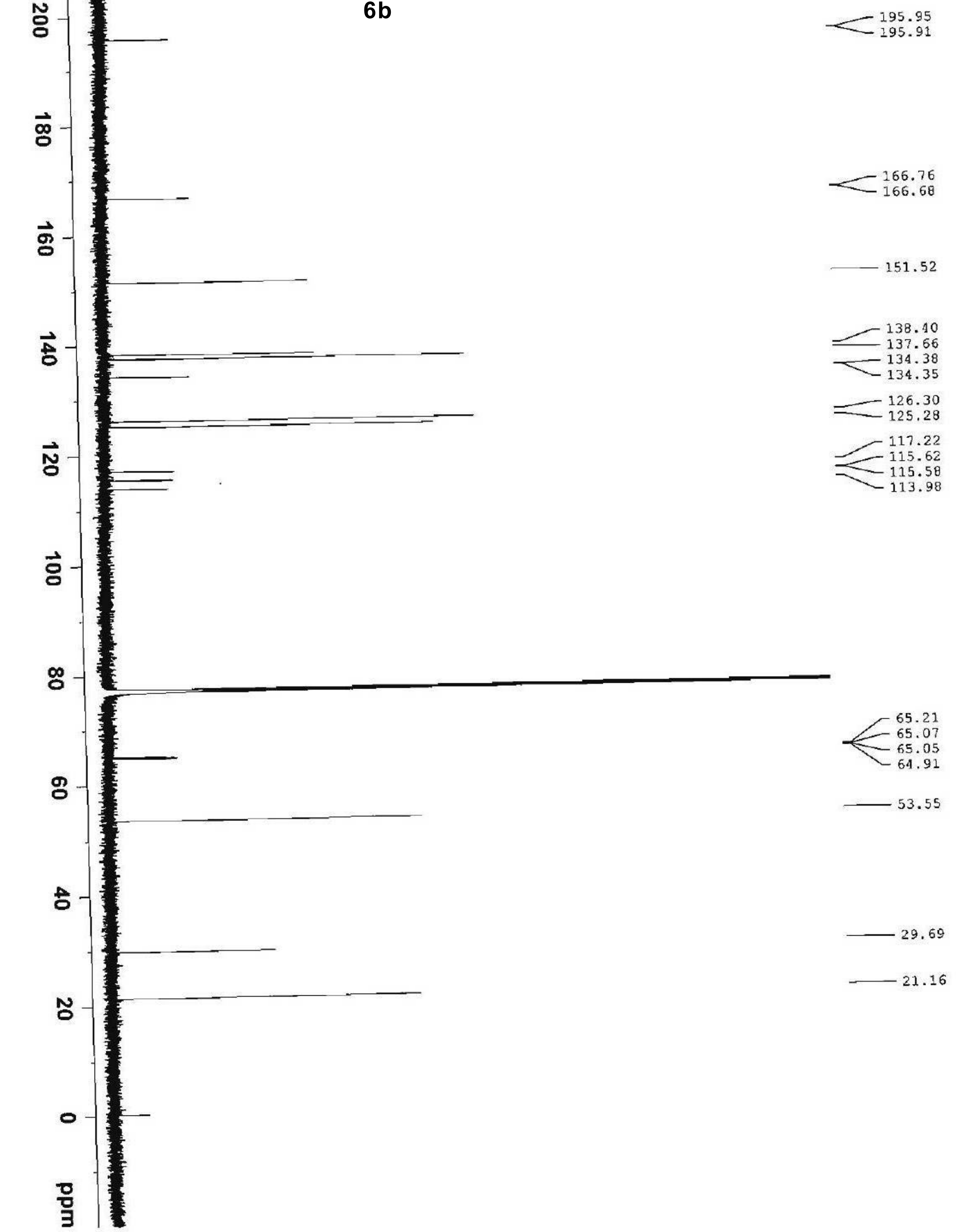

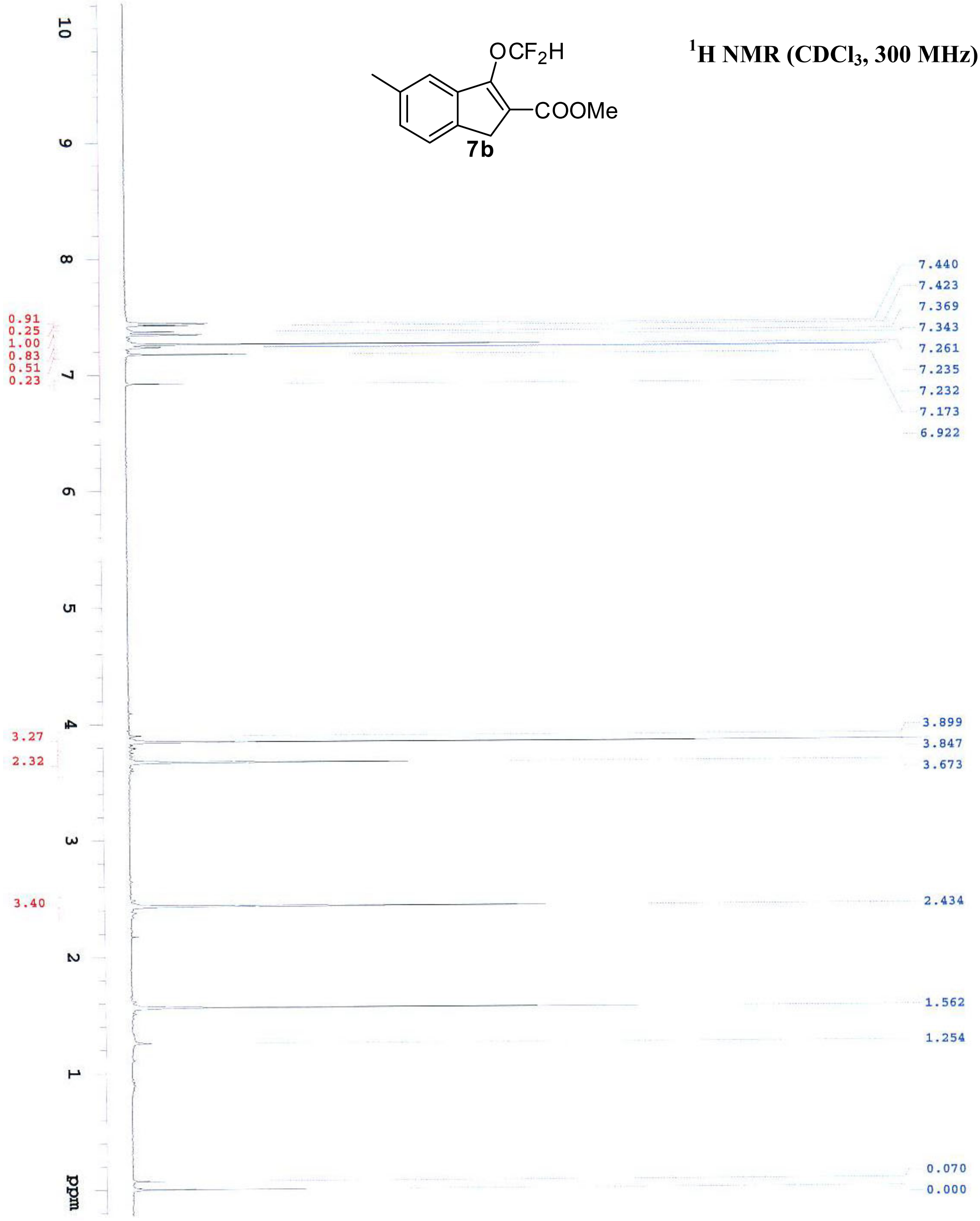

OCF2H
COOMe
7b
1H NMR (CDCl3, 300 MHz)
7.440
7.423
7.369
7.343
7.261
7.235
7.232
7.173
6.922
3.899
3.847
3.673
2.434
1.562
1.254
0.070
0.000
0.91
0.25
1.00
0.83
0.51
0.23
3.27
2.32
3.40
10
9
8
7
6
5
4
3
2
1
ppm

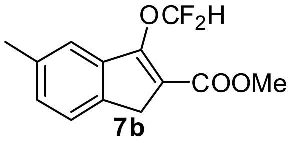

7b

^{19}F NMR ($CDCl_3$, 282 MHz)

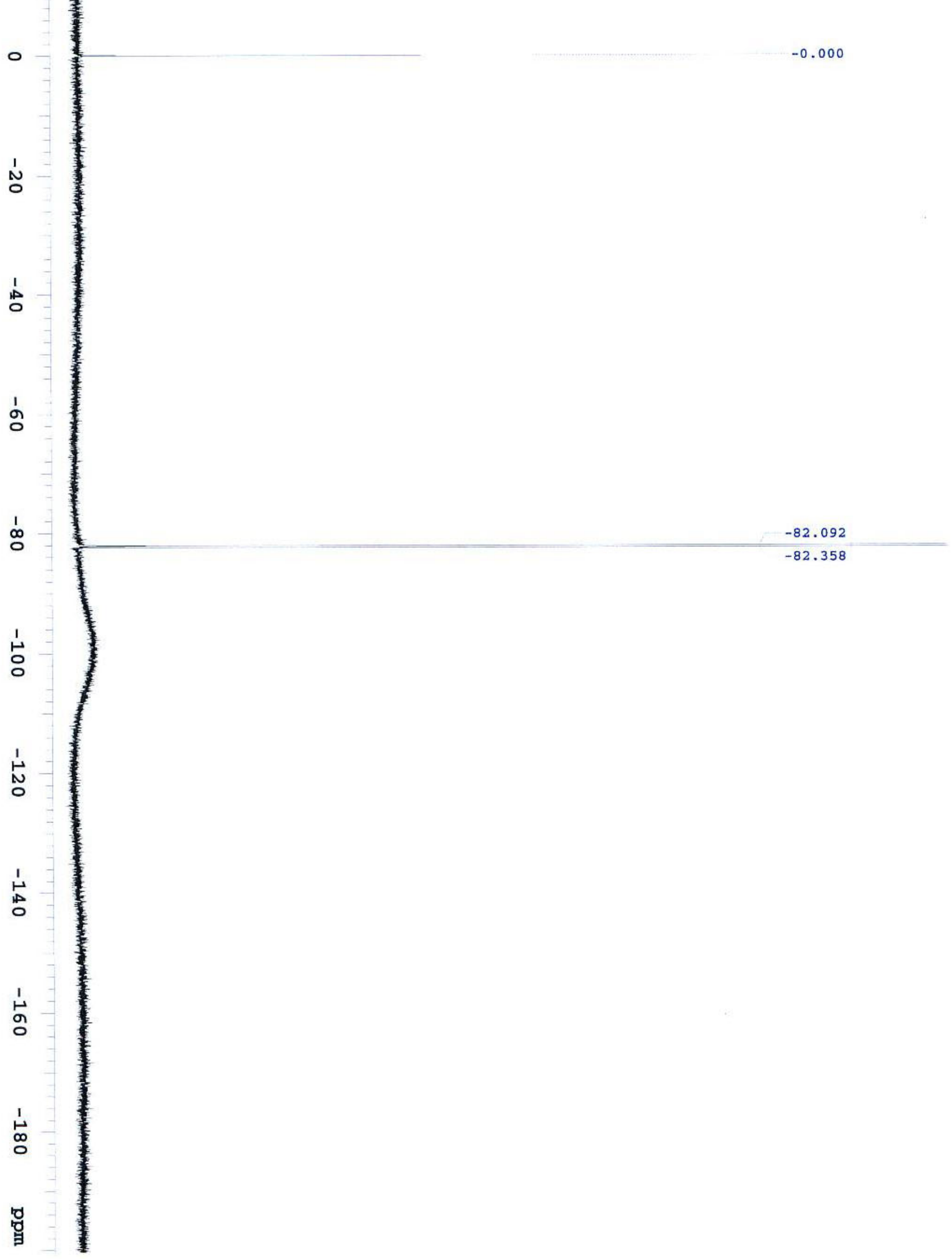

^{13}C NMR ($CDCl_3$, 150.9 MHz)

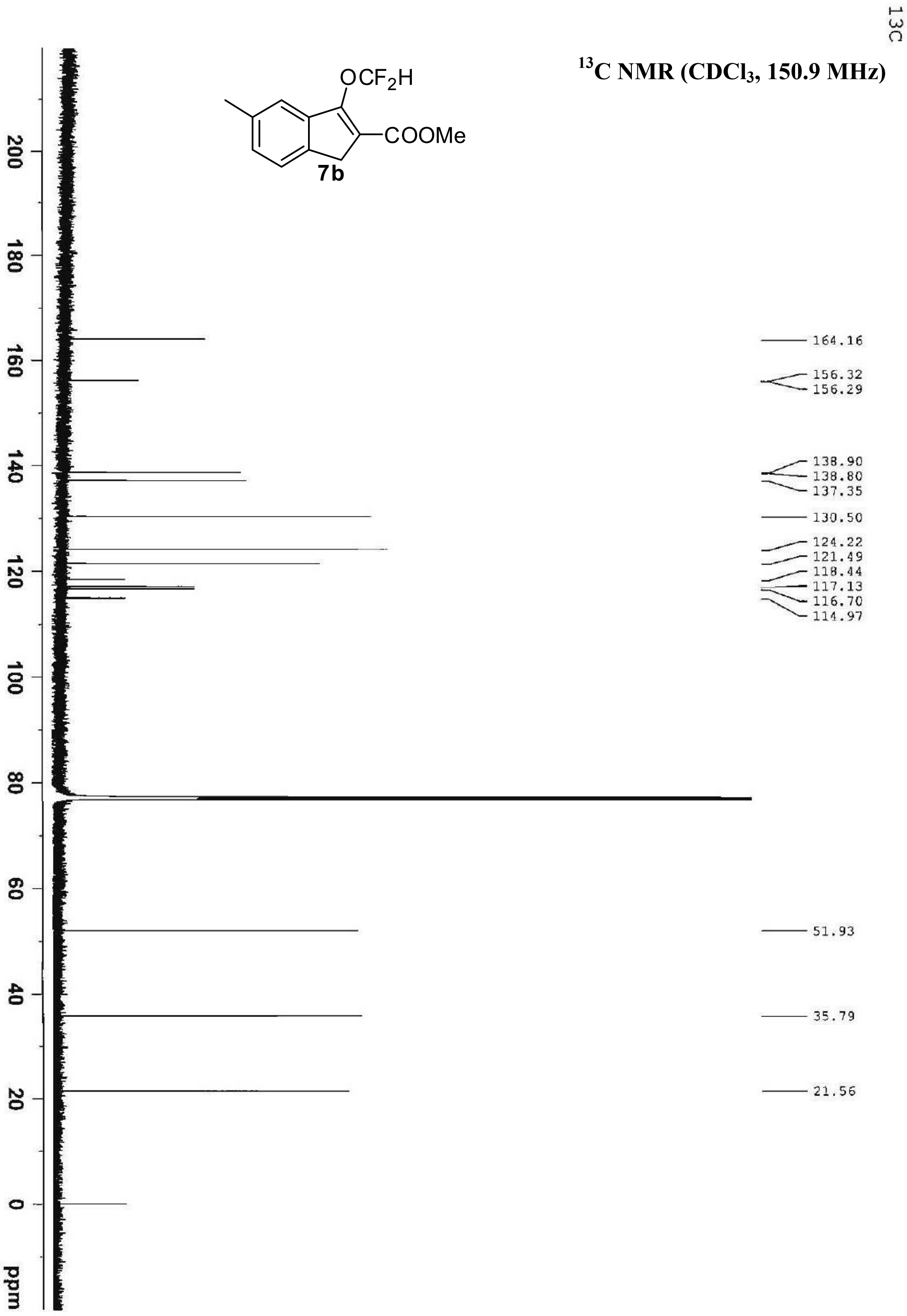

^{1}H NMR ($CDCl_3$, 300 MHz)

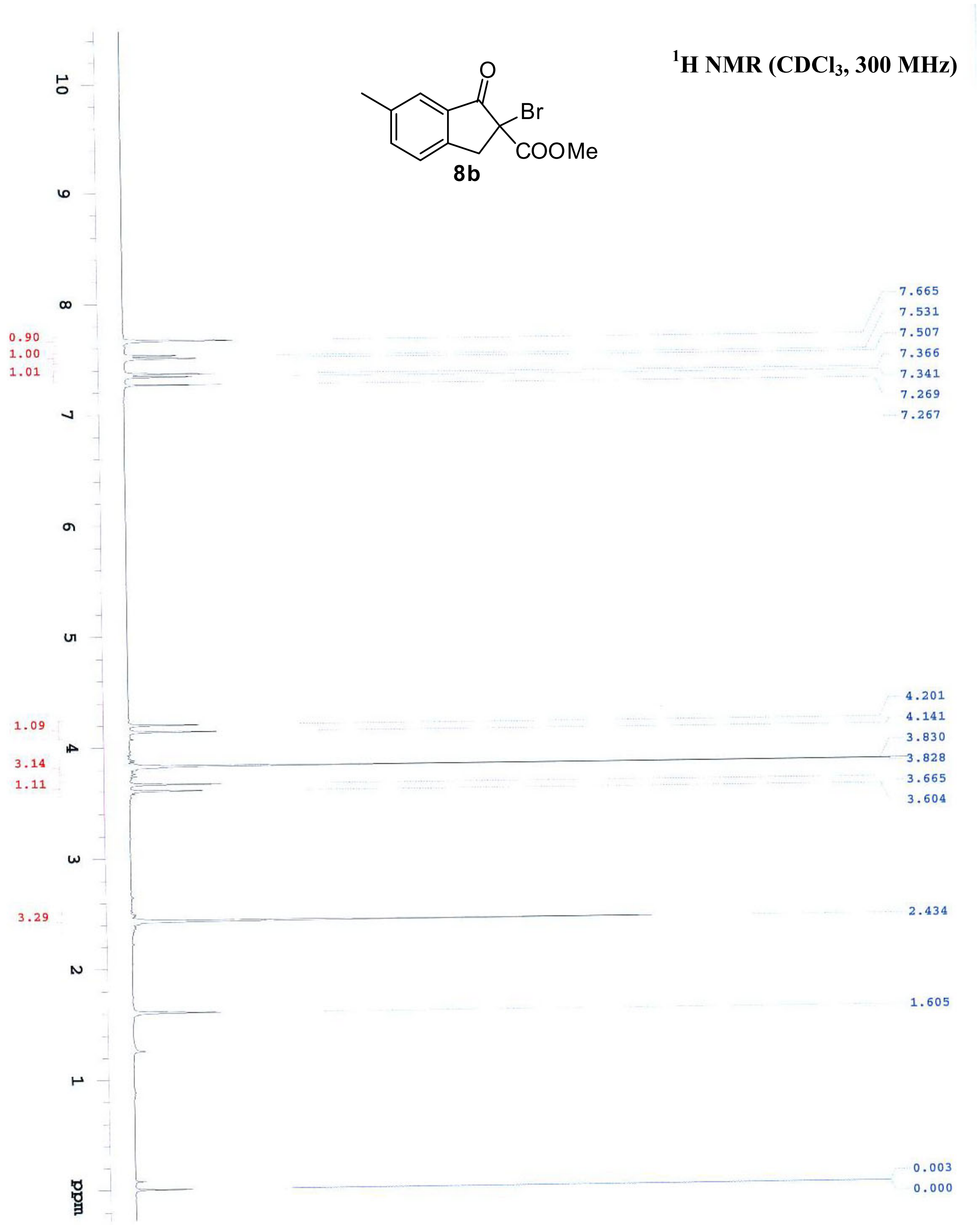

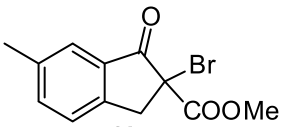

8b

^{13}C NMR ($CDCl_3$, 150.9 MHz)

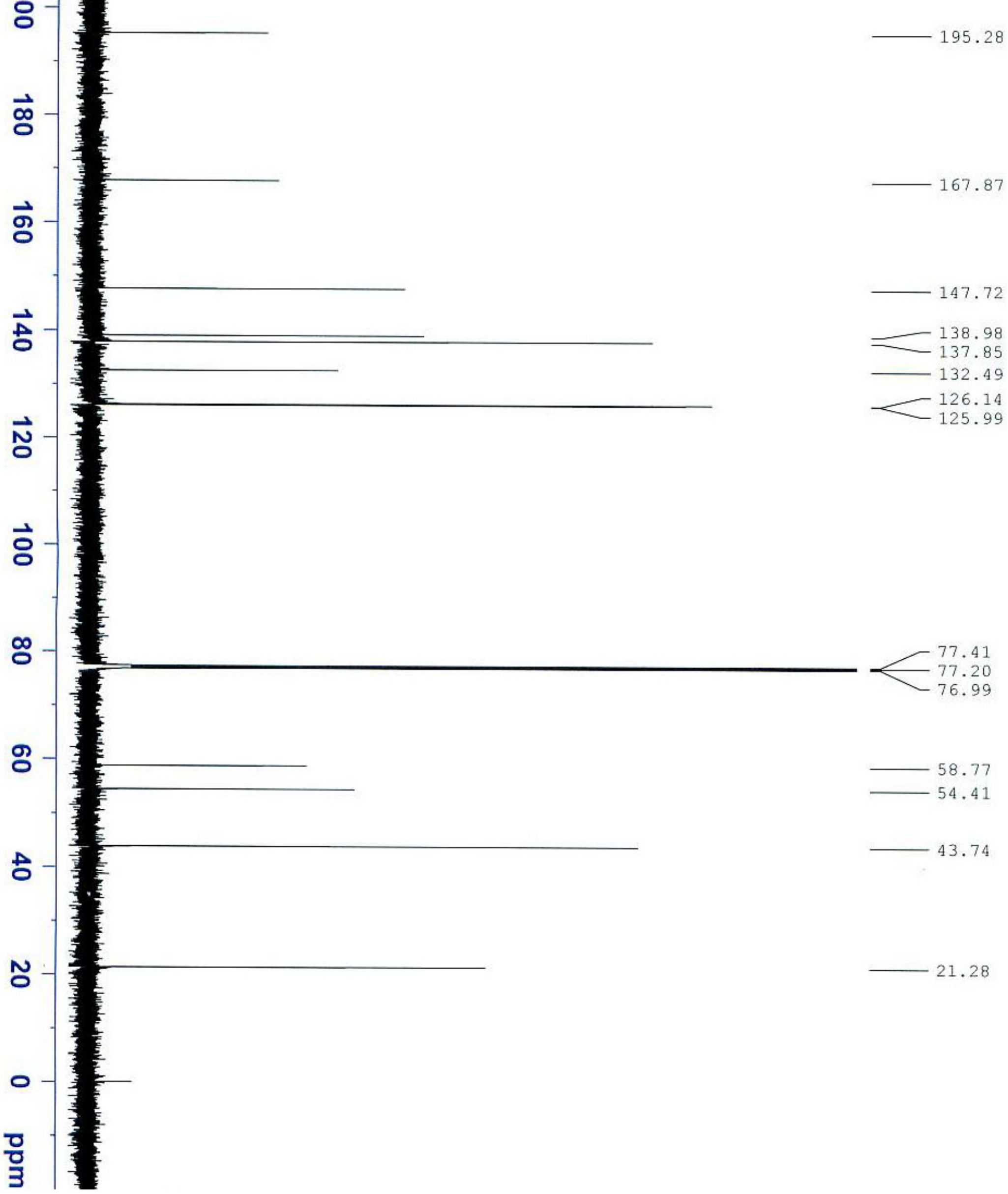

^{1}H NMR ($CDCl_3$, 300 MHz)

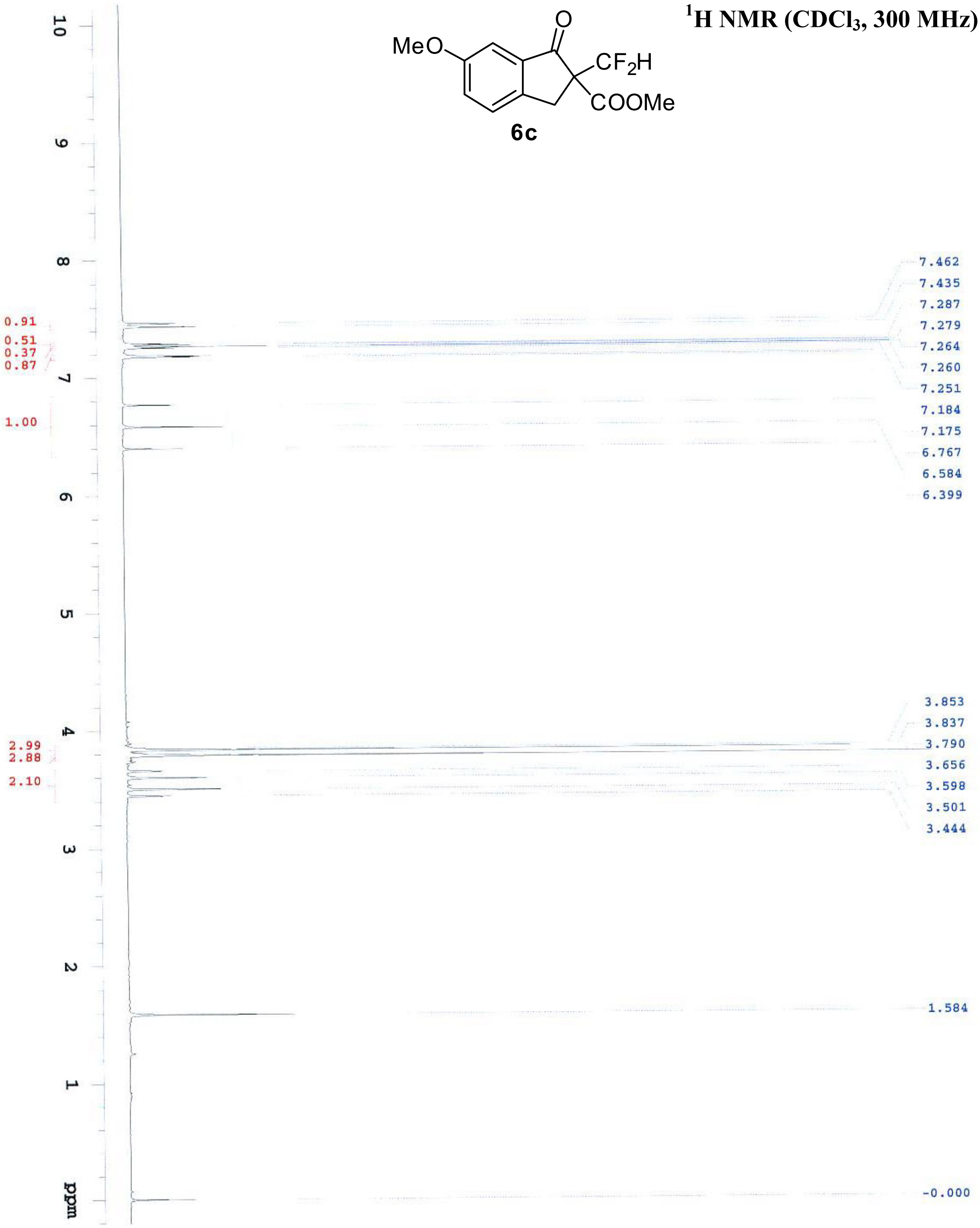

6c

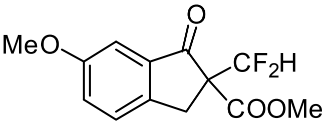

6c

^{19}F NMR ($CDCl_3$, 282 MHz)

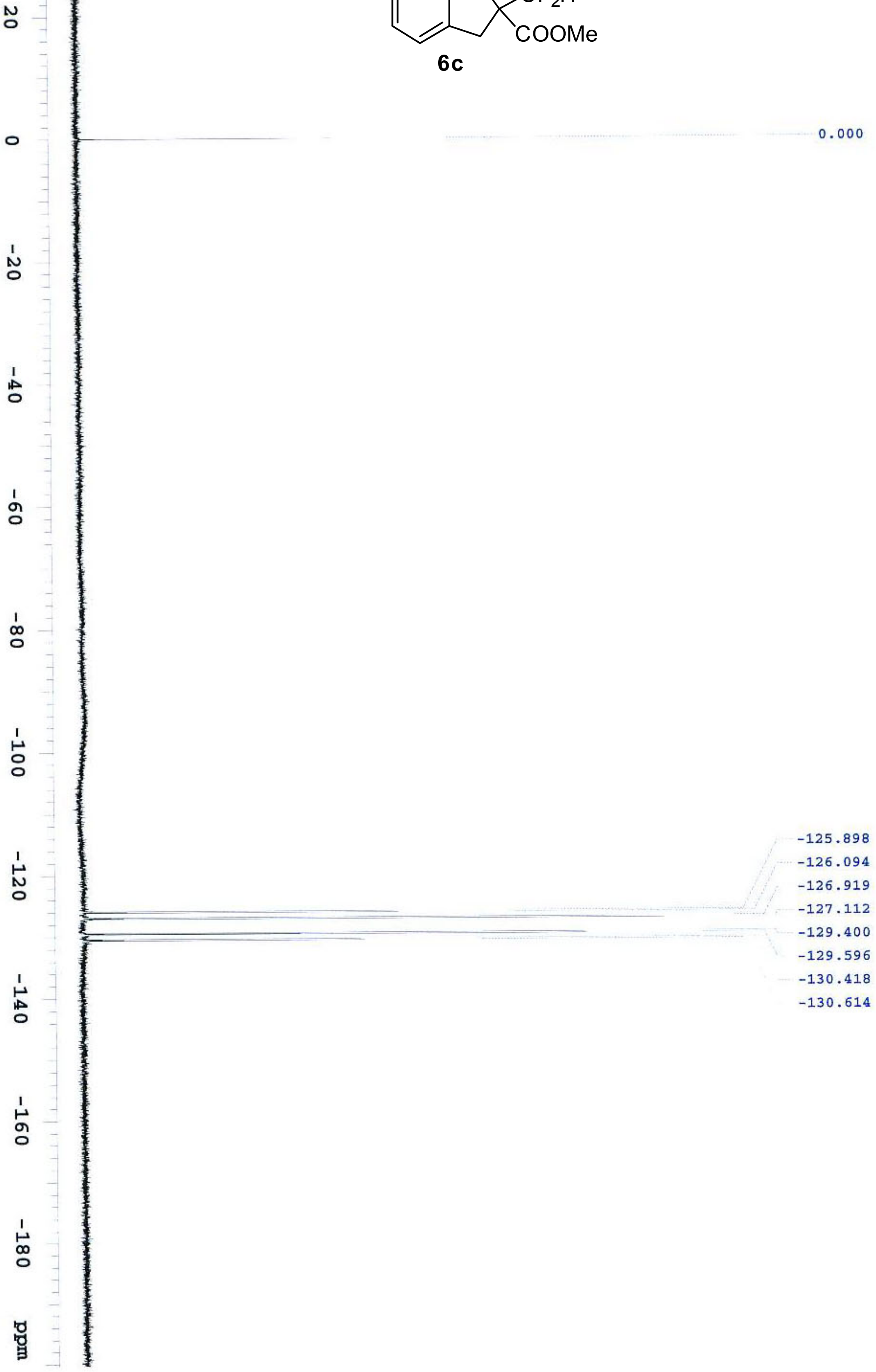

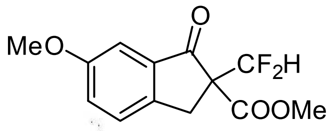

6c

^{13}C NMR ($CDCl_3$, 150.9 MHz)

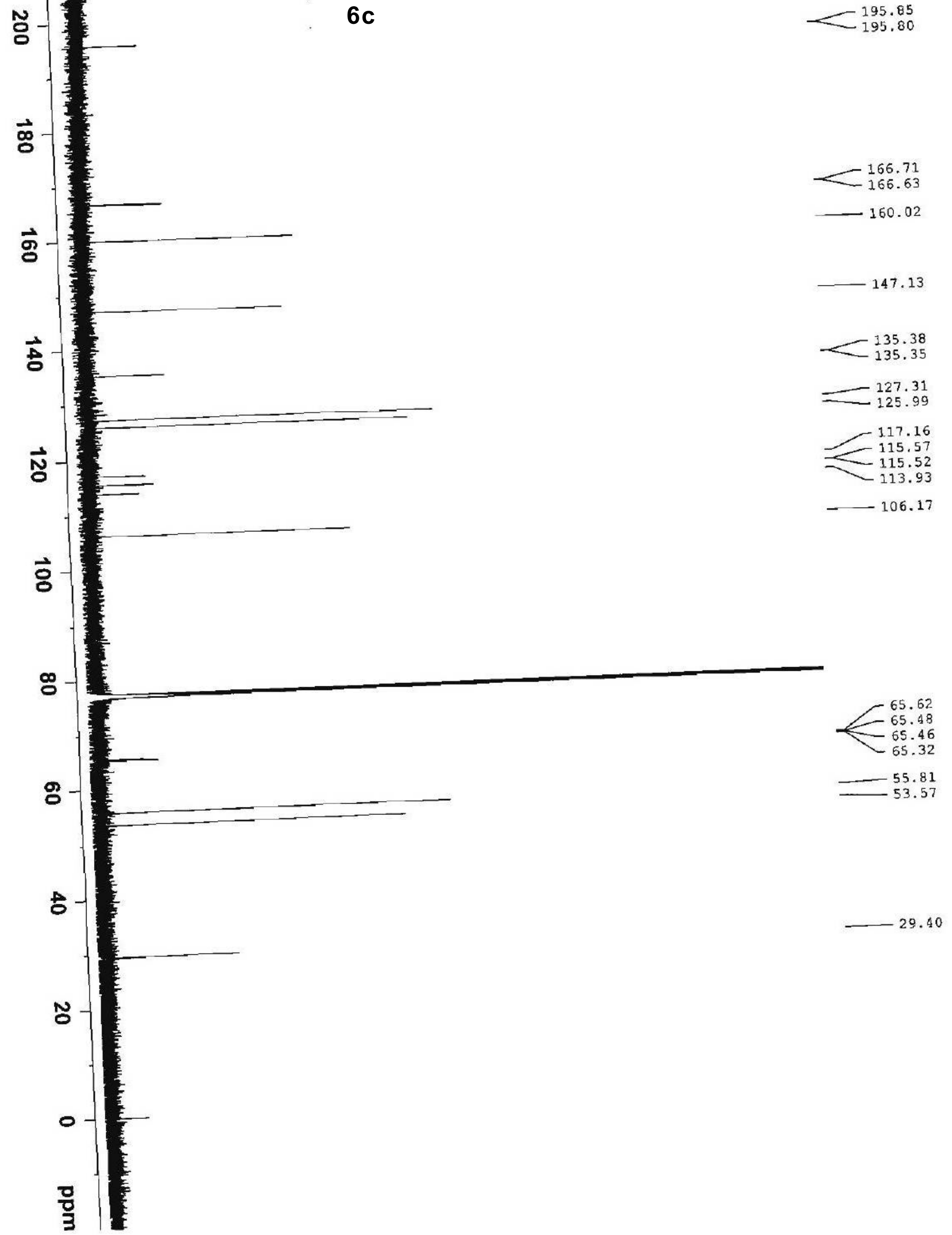

^{1}H NMR ($CDCl_3$, 300 MHz)

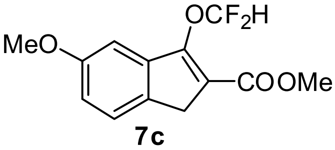

7c

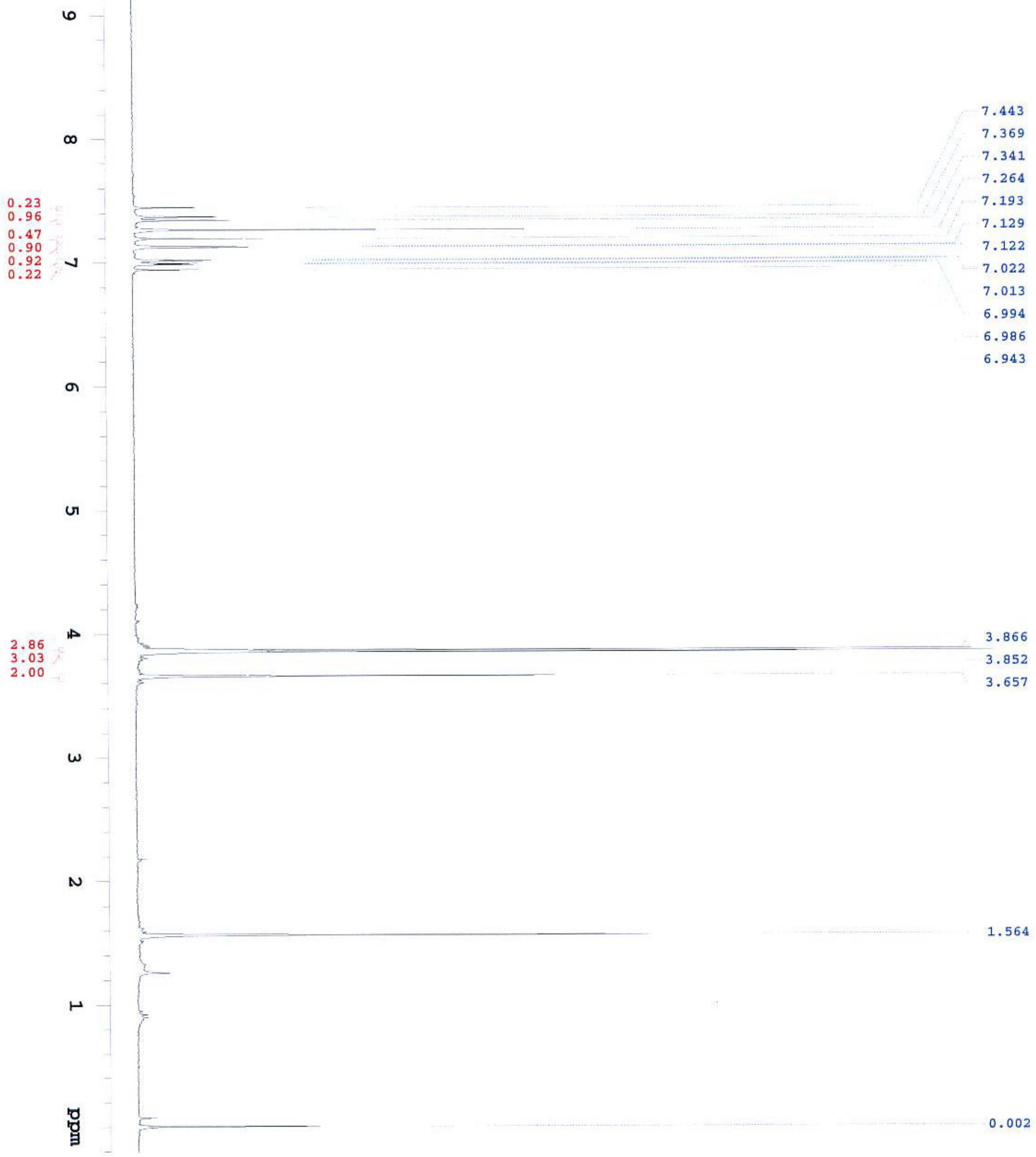

^{19}F NMR ($CDCl_3$, 282 MHz)

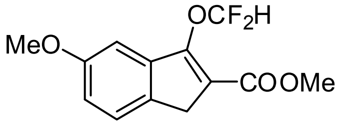

7c

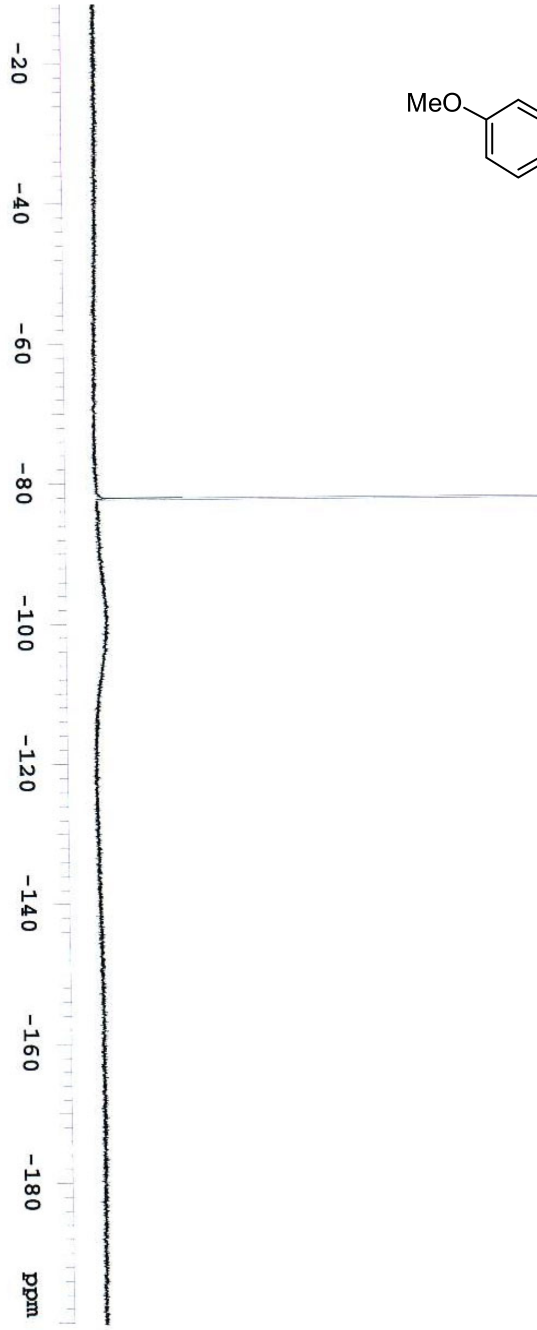

^{13}C NMR ($CDCl_3$, 150.9 MHz)

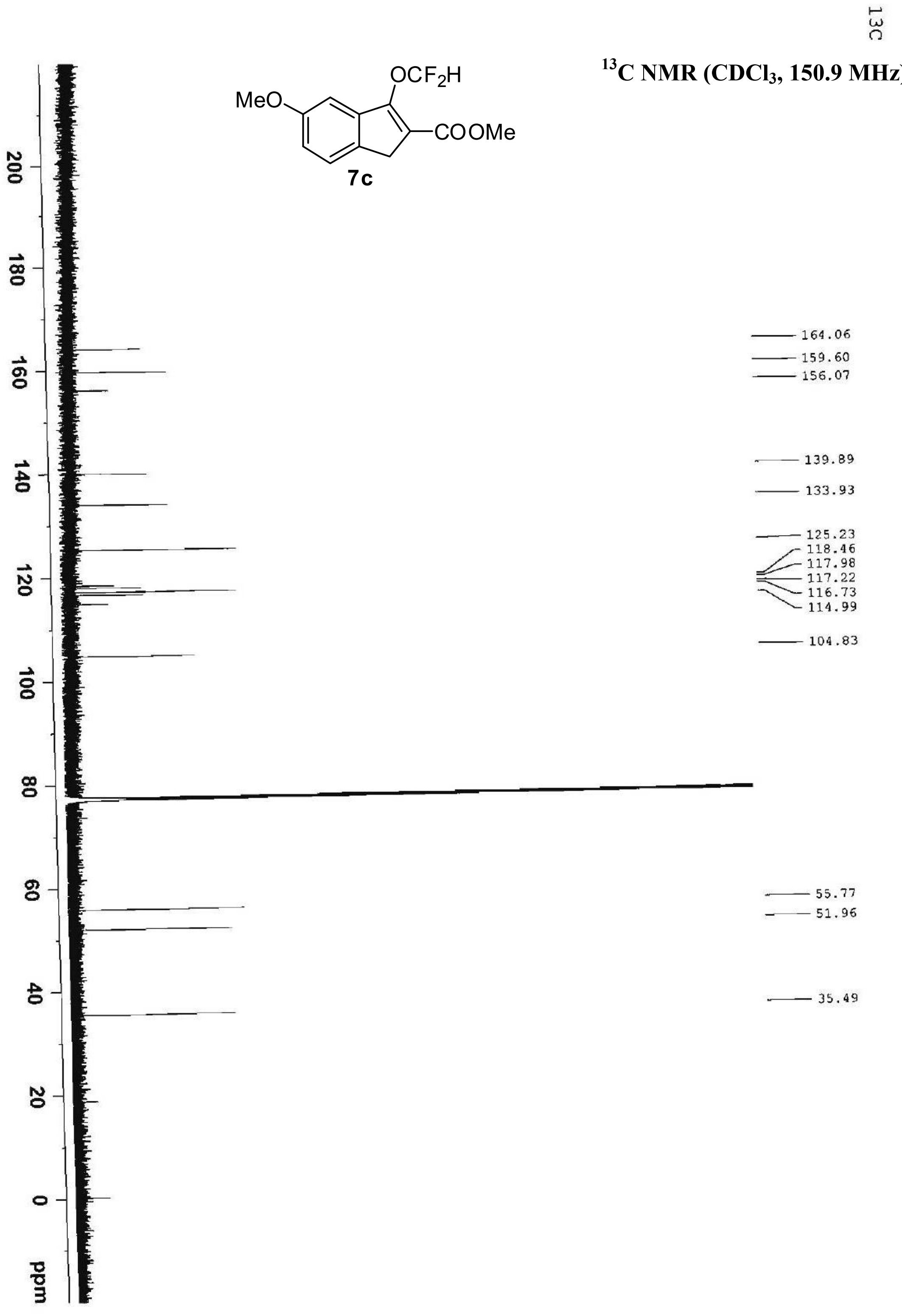

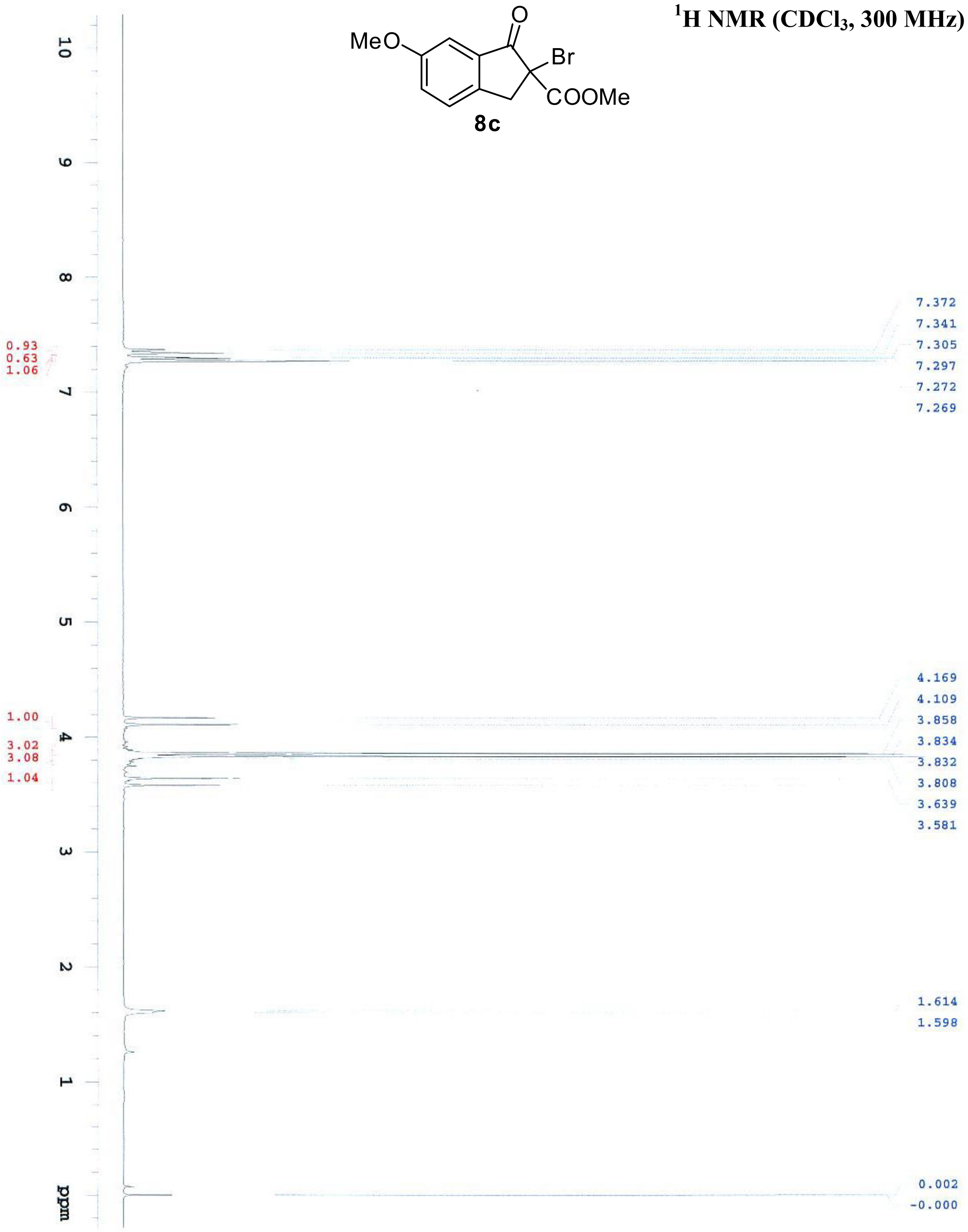
1H NMR (CDCl3, 300 MHz)
MeO
O
Br
COOMe
8c
7.372
7.341
7.305
7.297
7.272
7.269
4.169
4.109
3.858
3.834
3.832
3.808
3.639
3.581
1.614
1.598
0.002
-0.000
0.93
0.63
1.06
1.00
3.02
3.08
1.04
10
9
8
7
6
5
4
3
2
1
ppm

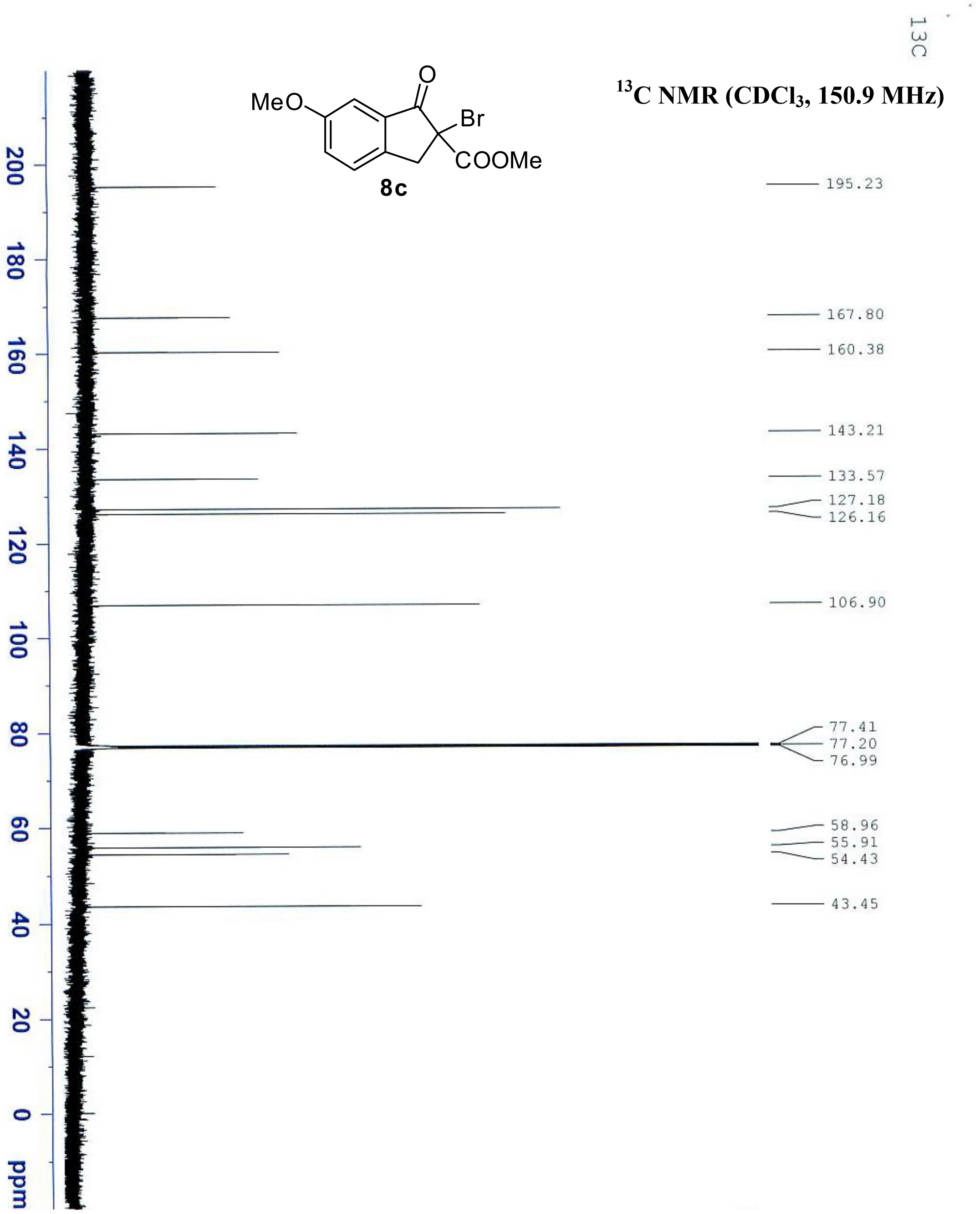
13C
^{13}C NMR ($CDCl_3$, 150.9 MHz)
MeO
O
Br
COOMe
8c
195.23
167.80
160.38
143.21
133.57
127.18
126.16
106.90
77.41
77.20
76.99
58.96
55.91
54.43
43.45
200
180
160
140
120
100
80
60
40
20
0
ppm

^{1}H NMR ($CDCl_3$, 300 MHz)

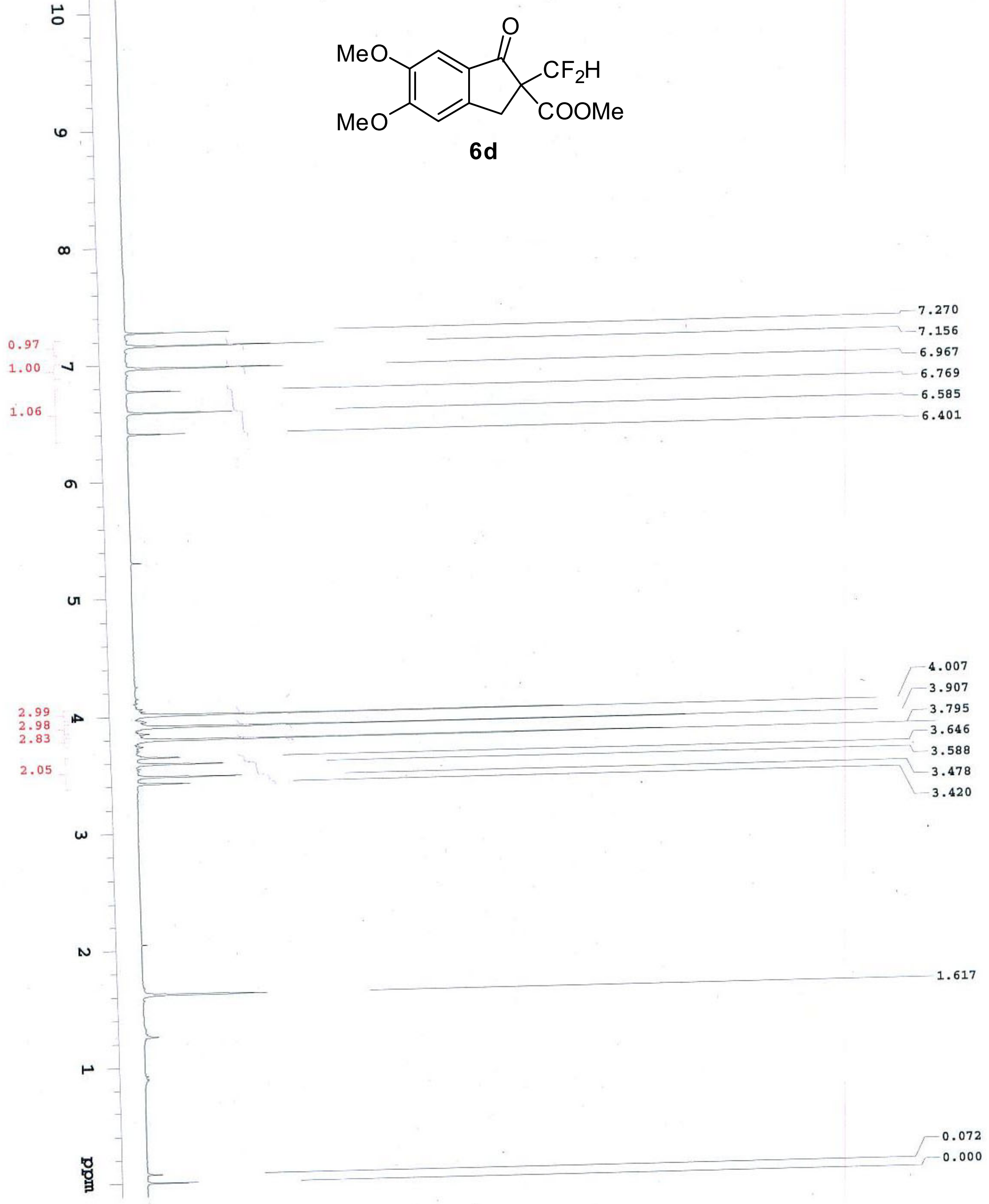

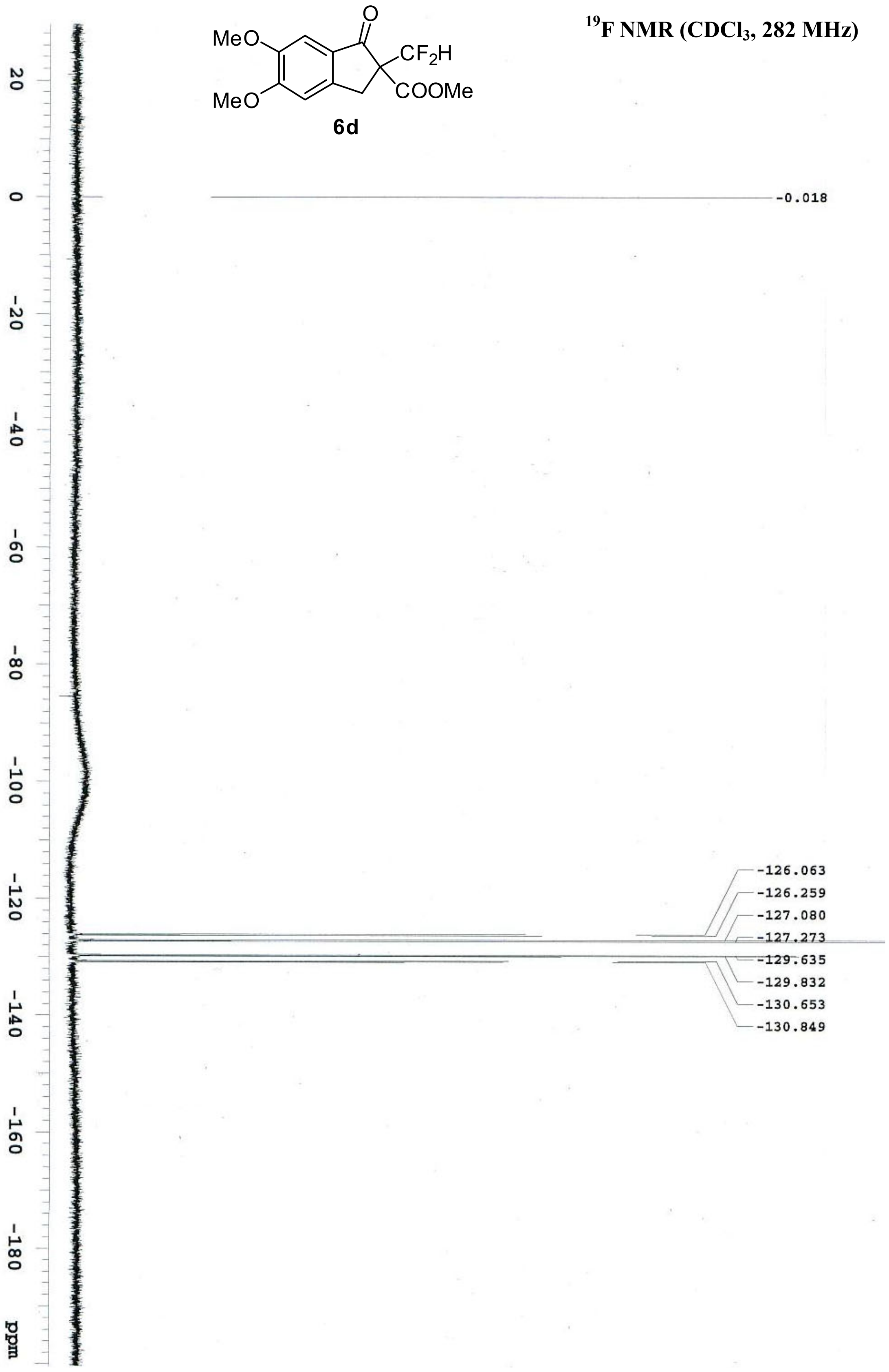

^{19}F NMR ($CDCl_3$, 282 MHz)

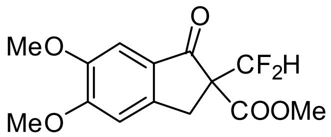

6d

^{13}C NMR ($CDCl_3$, 150.9 MHz)

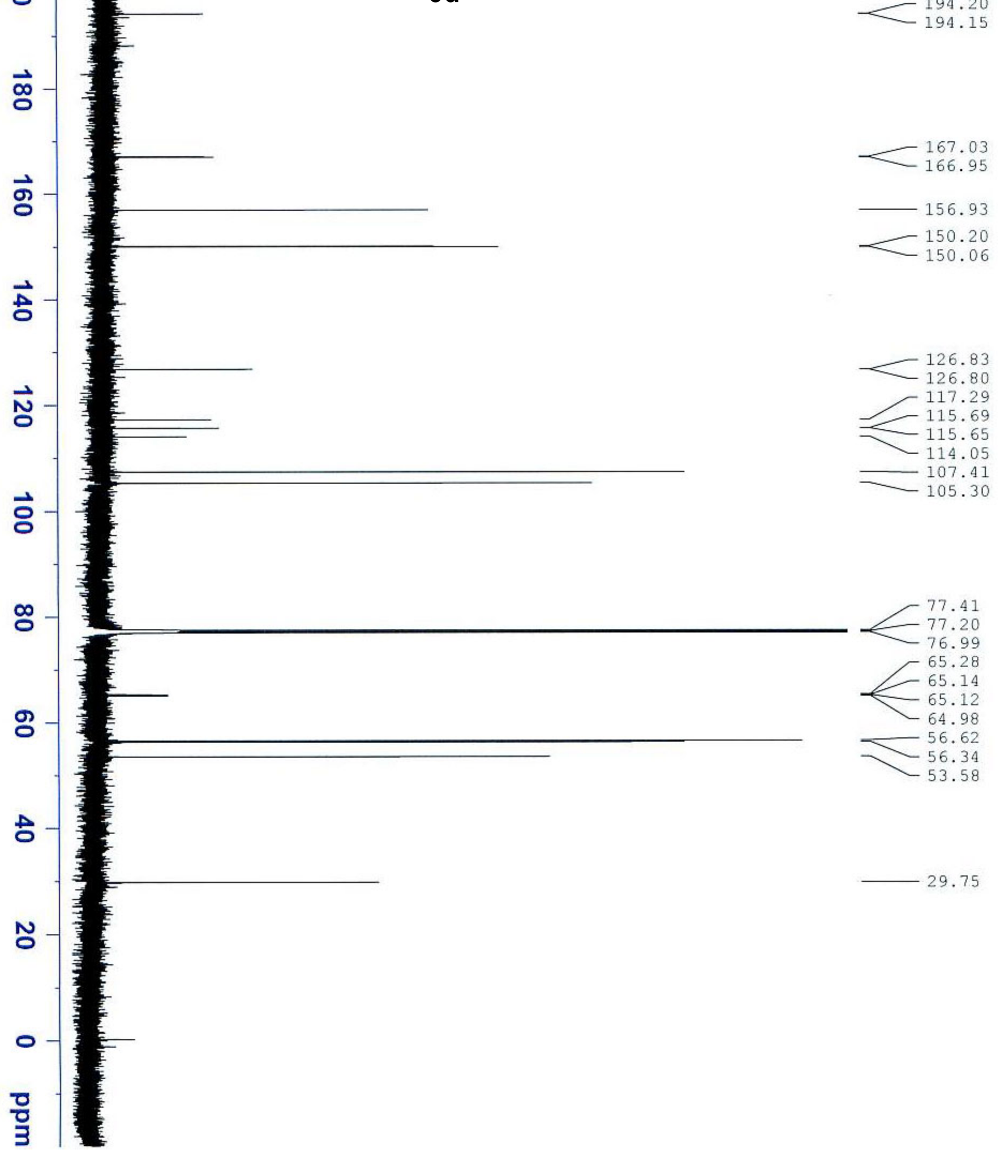

^{1}H NMR ($CDCl_3$, 300 MHz)

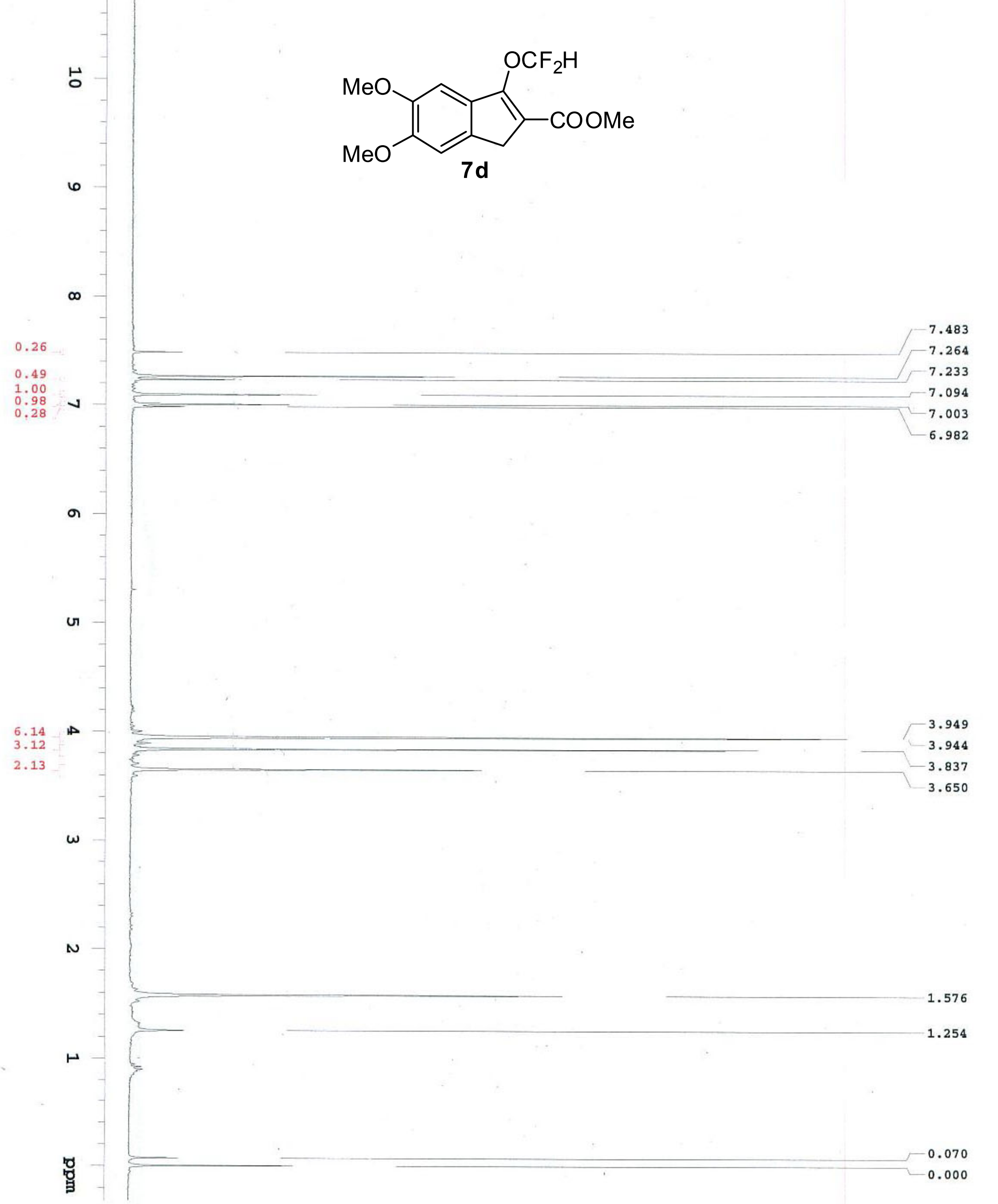

^{19}F NMR ($CDCl_3$, 282 MHz)

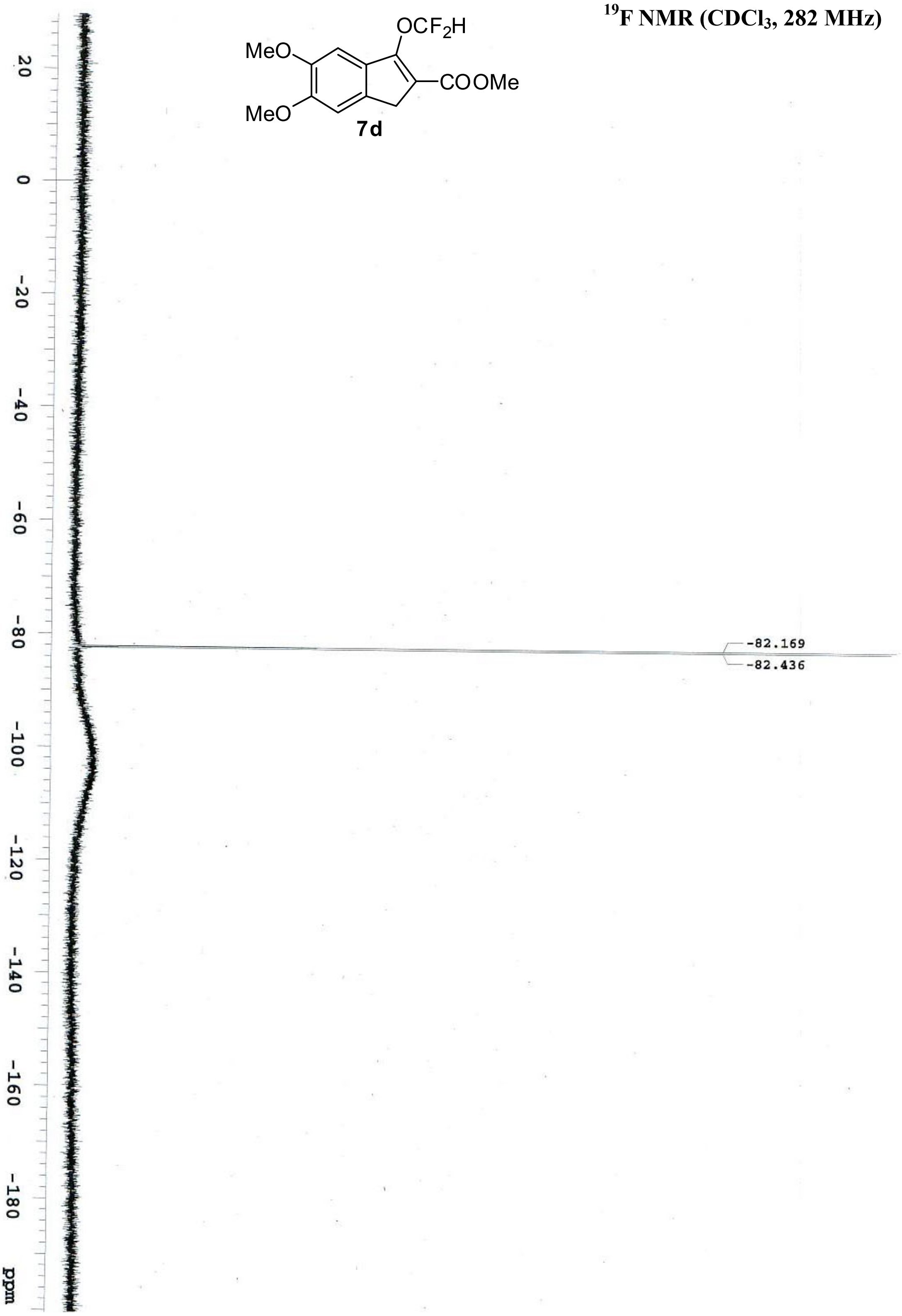

^{13}C NMR ($CDCl_3$, 150.9 MHz)

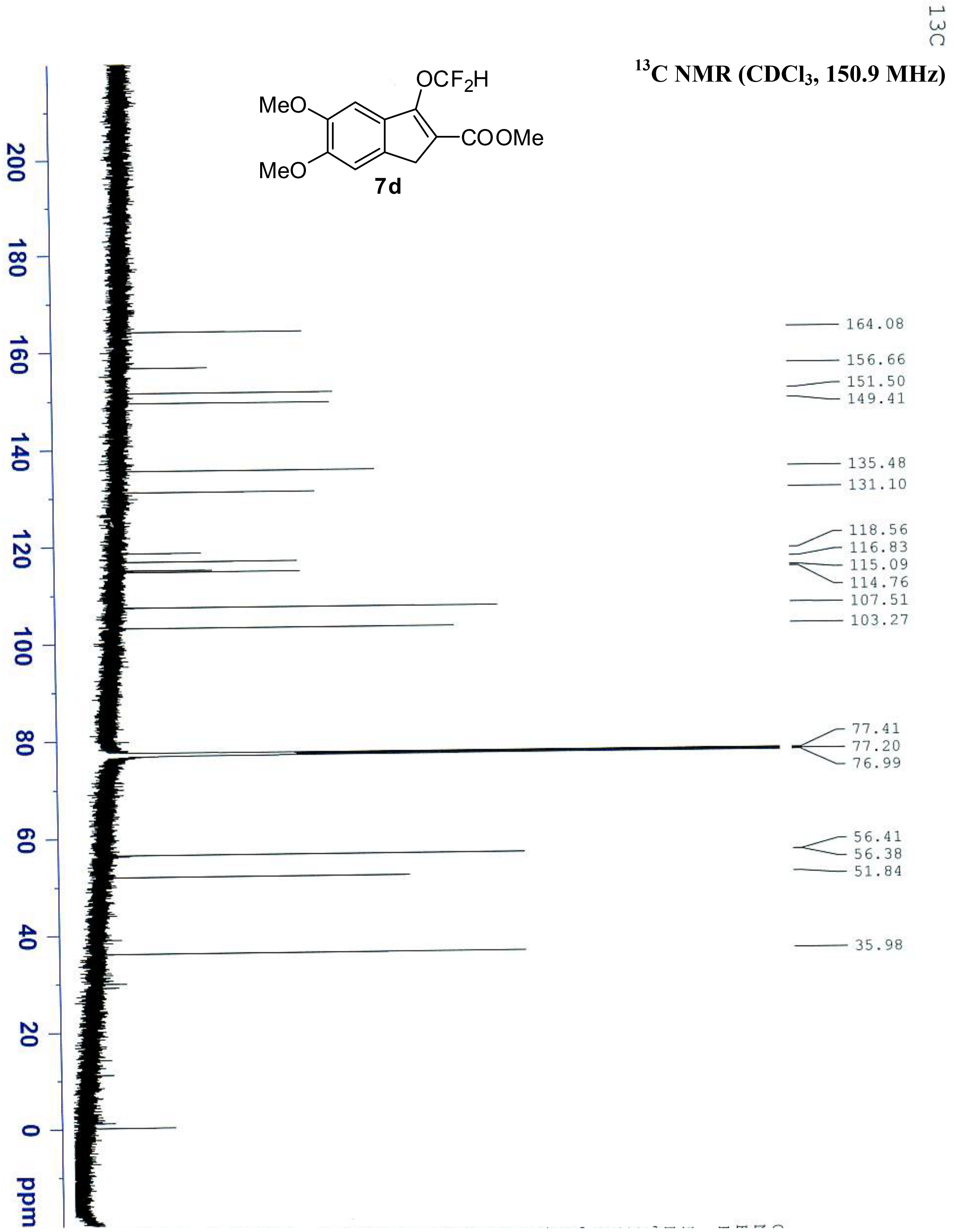

^{1}H NMR ($CDCl_3$, 300 MHz)

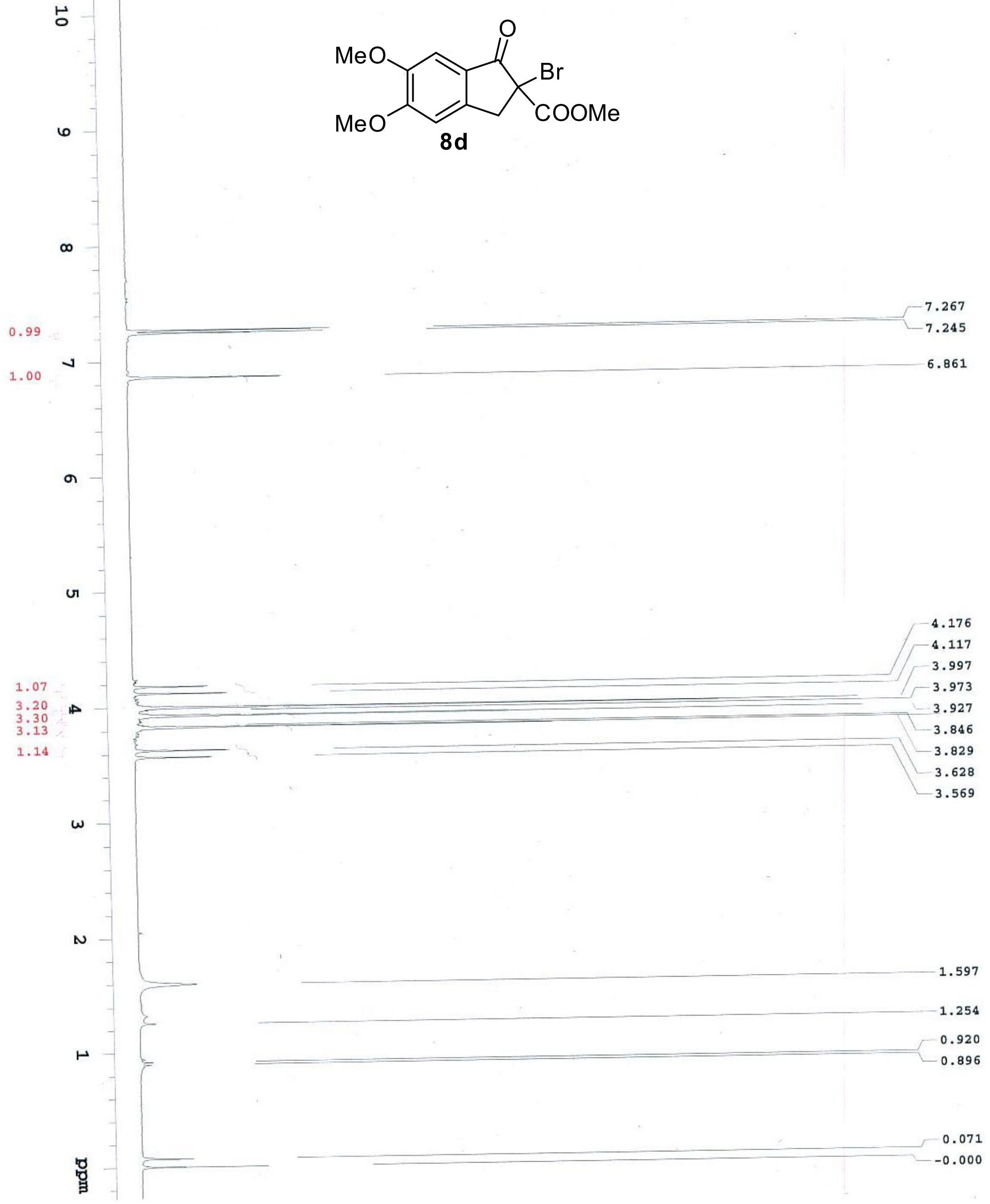

^{13}C NMR (CDCl$_3$, 150.9 MHz)

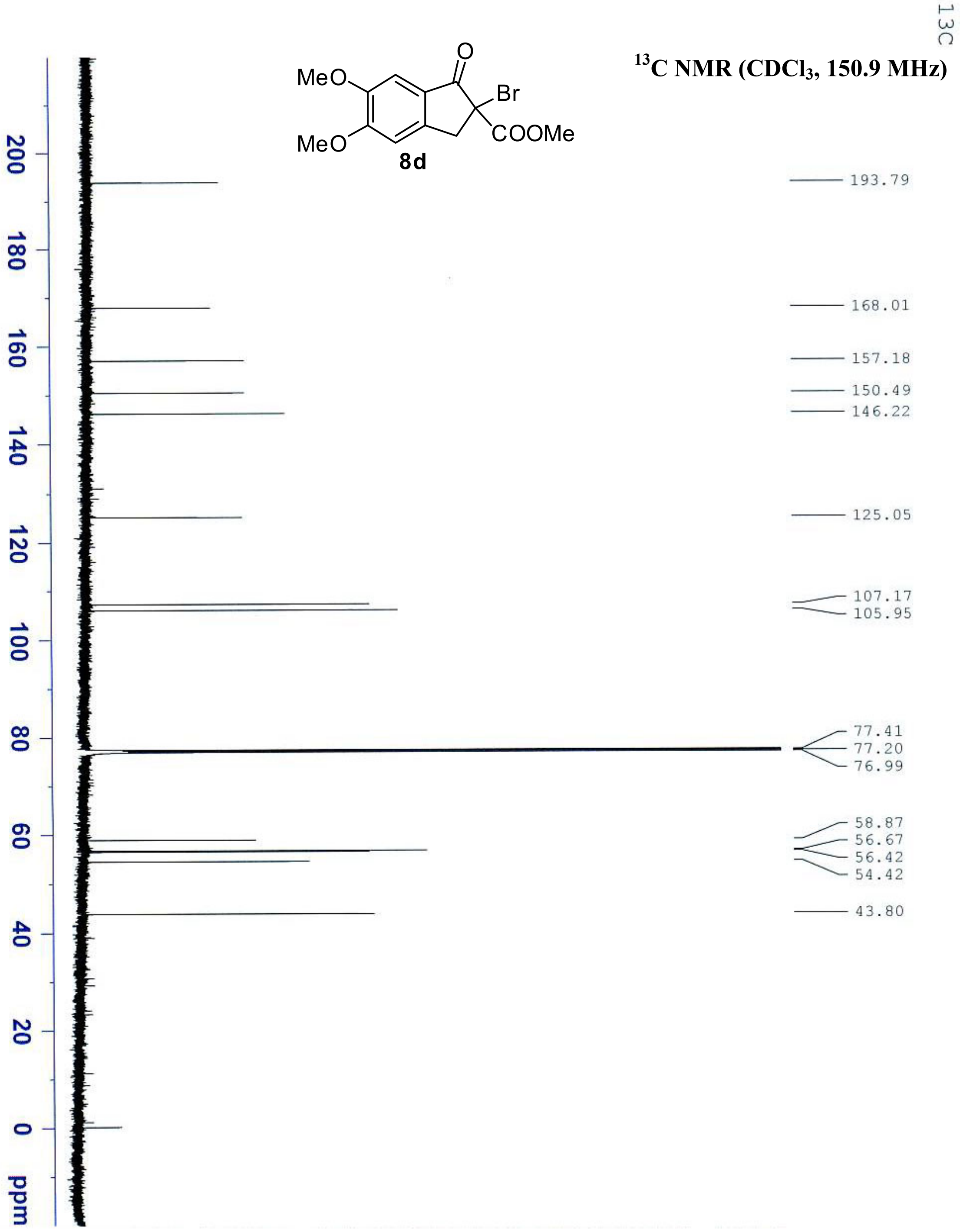

^{1}H NMR ($CDCl_3$, 300 MHz)

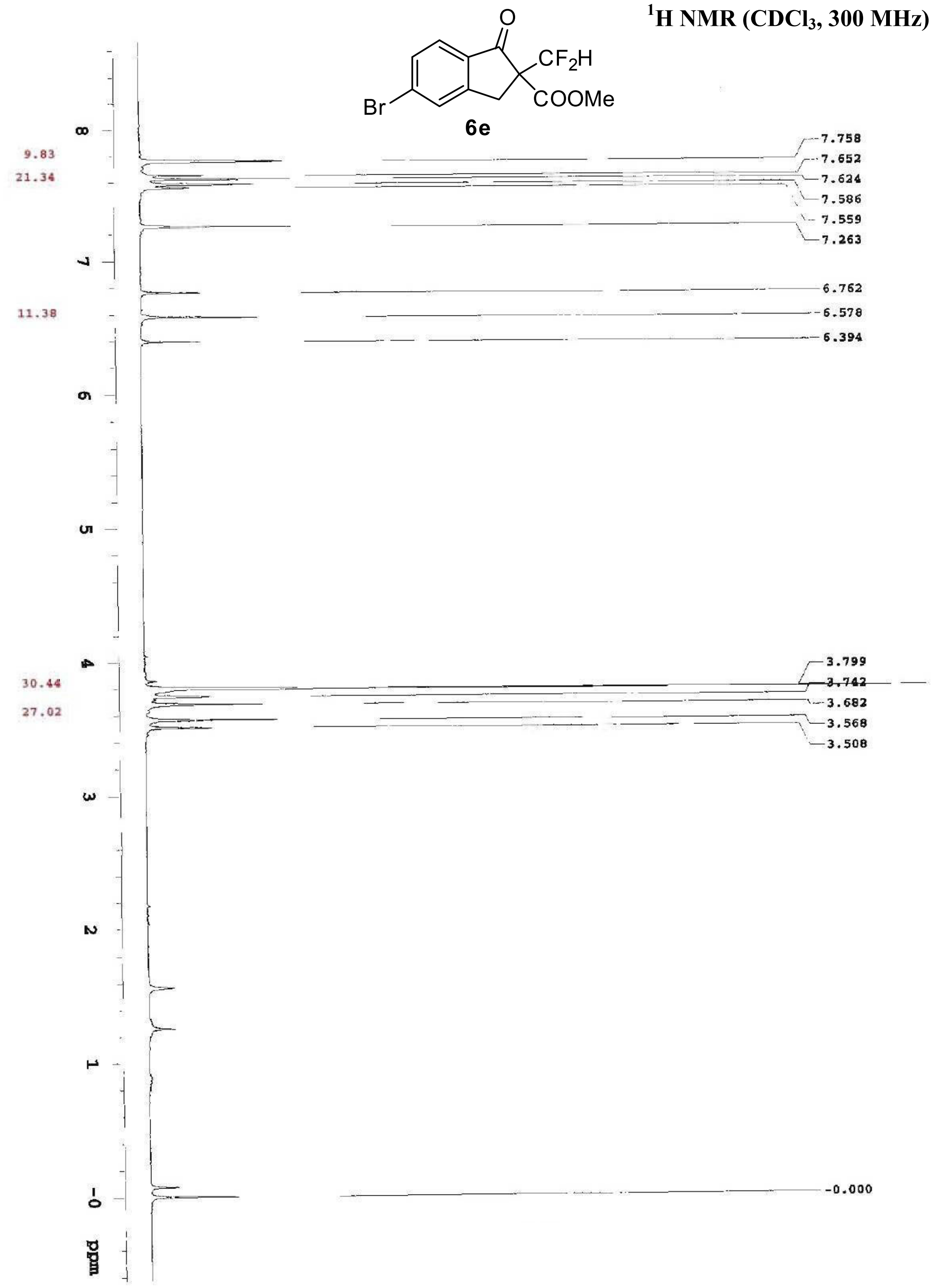

^{19}F NMR ($CDCl_3$, 282 MHz)

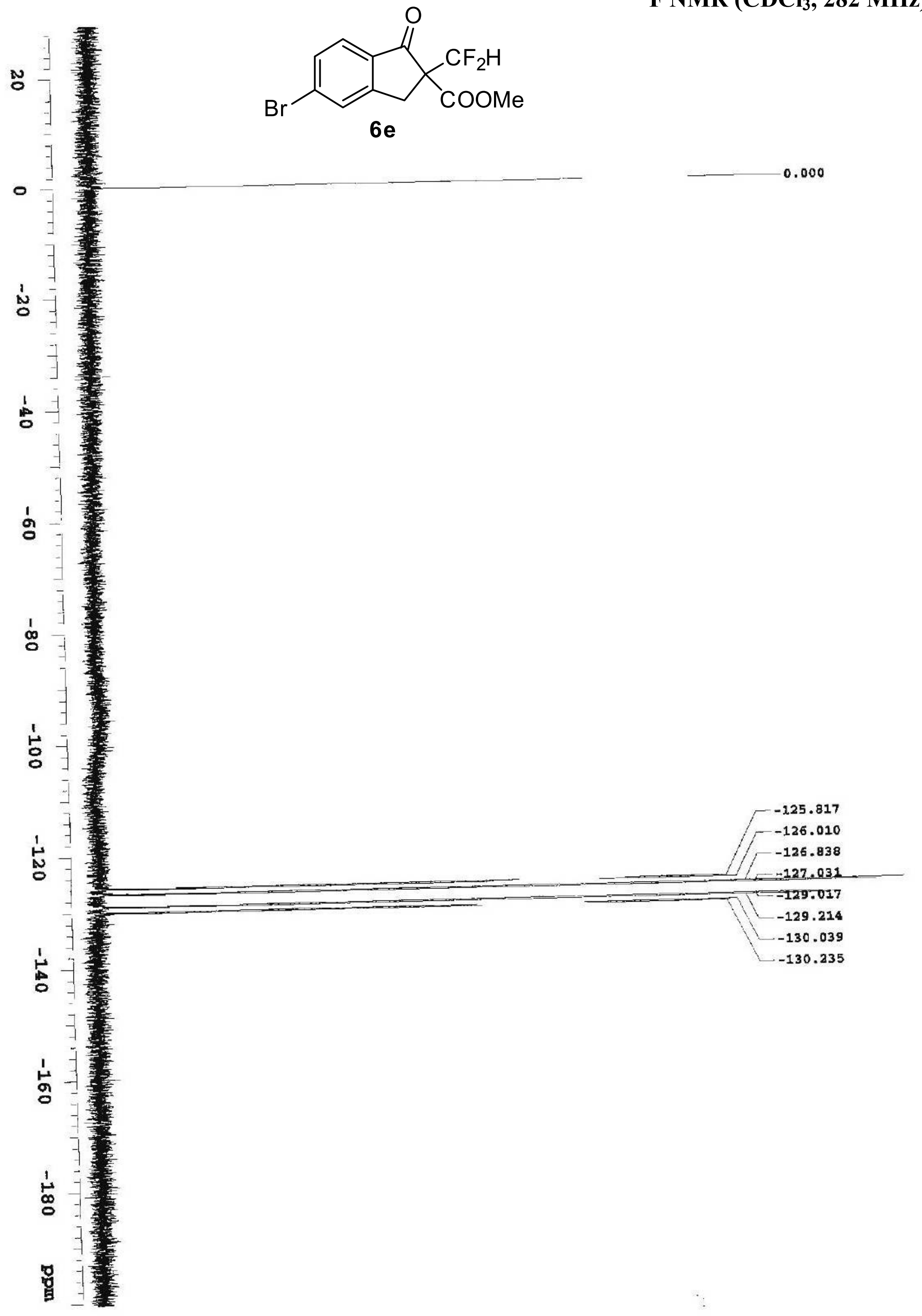

6e

^{13}C NMR ($CDCl_3$, 150.9 MHz)

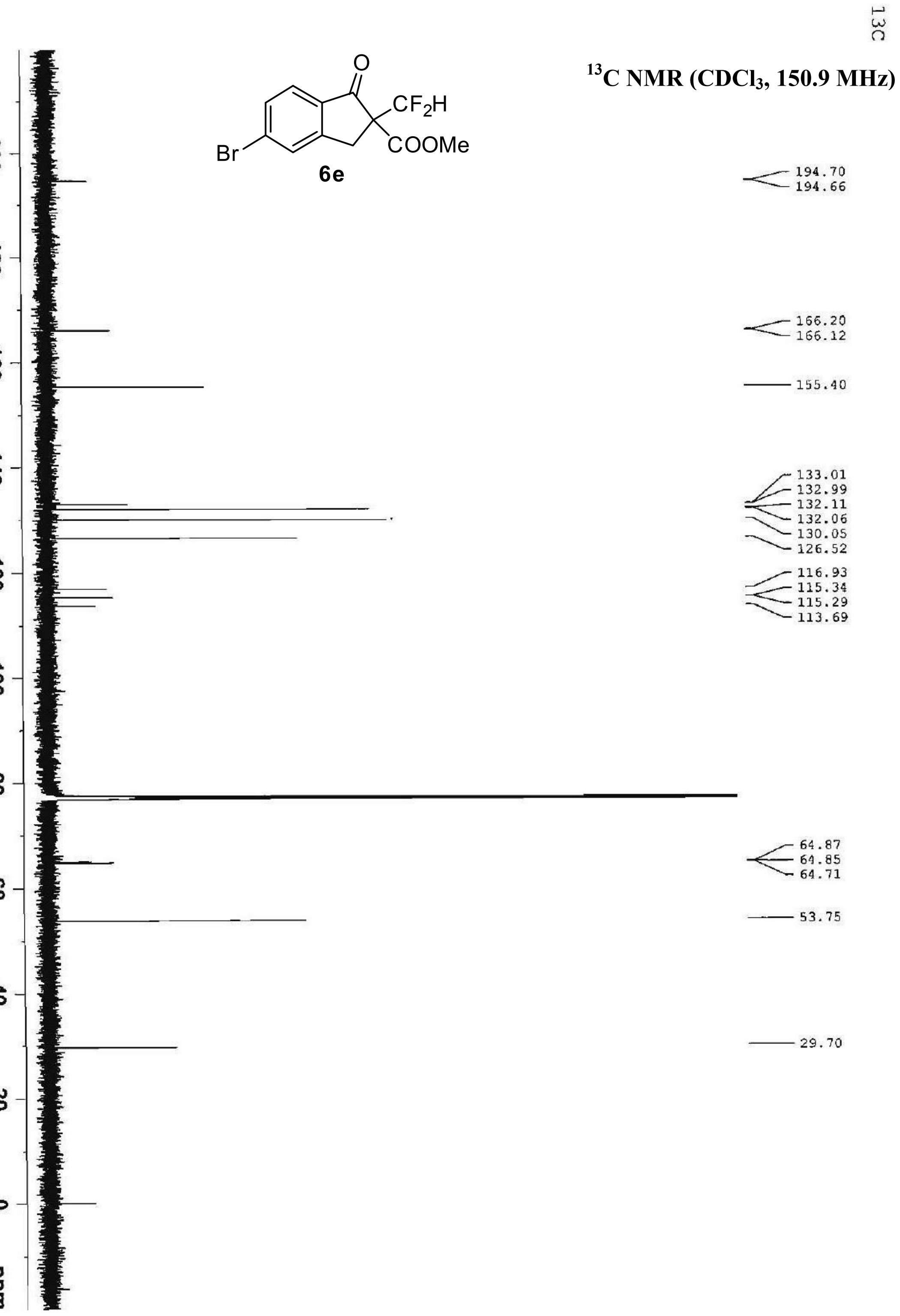

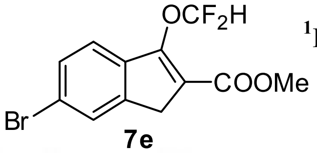

7e

^{1}H NMR (CDCl$_3$, 300 MHz)

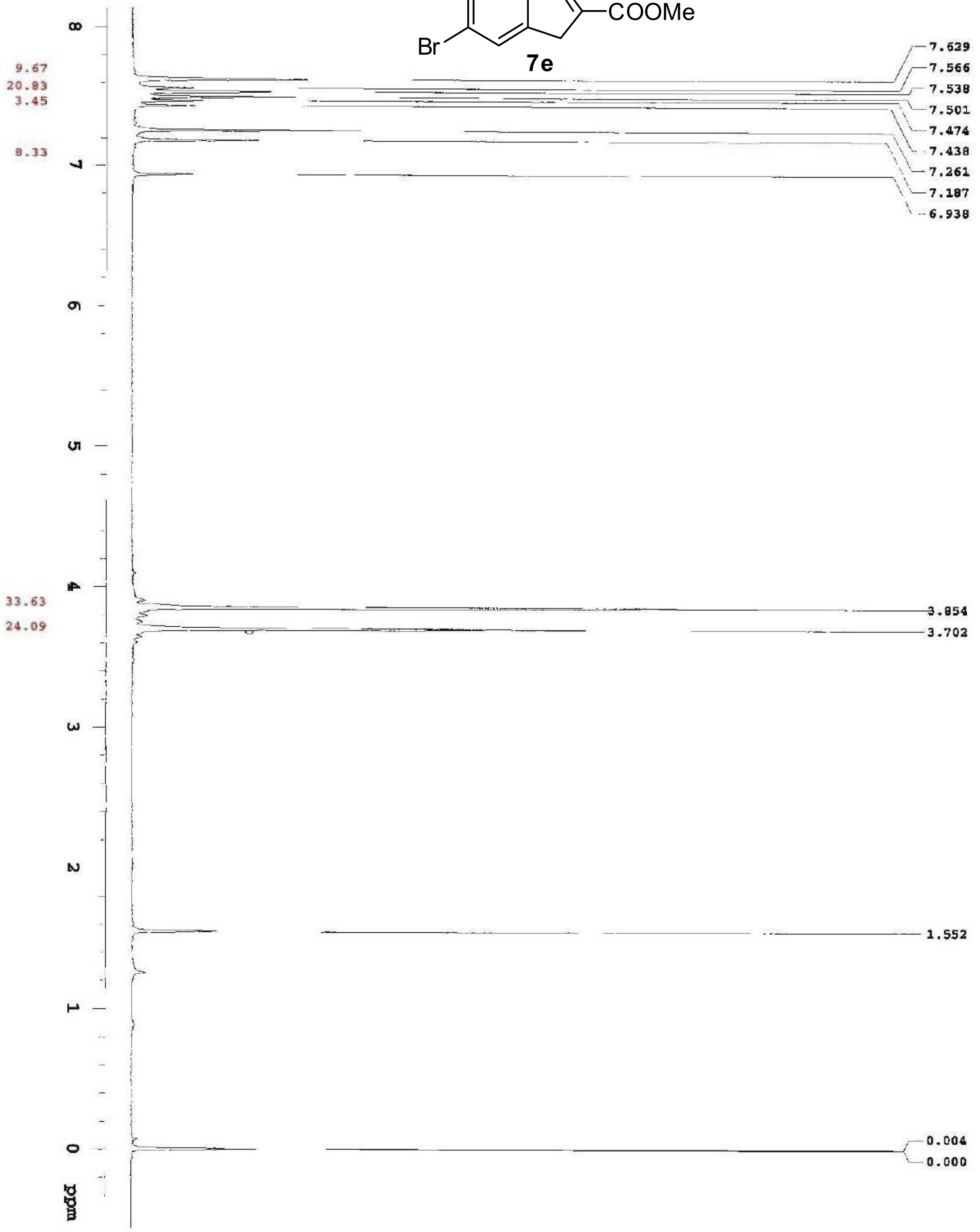

^{19}F NMR ($CDCl_3$, 282 MHz)

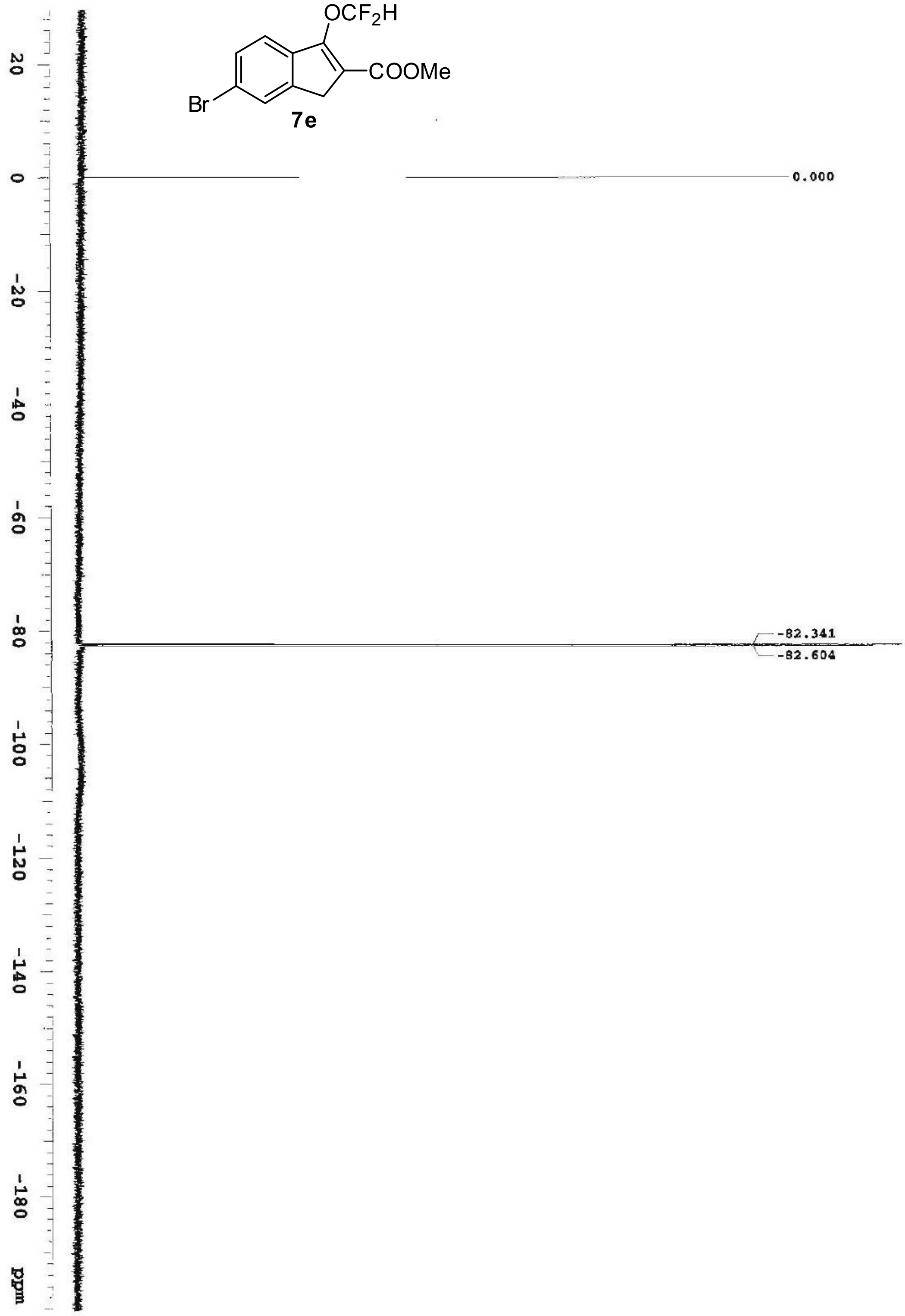

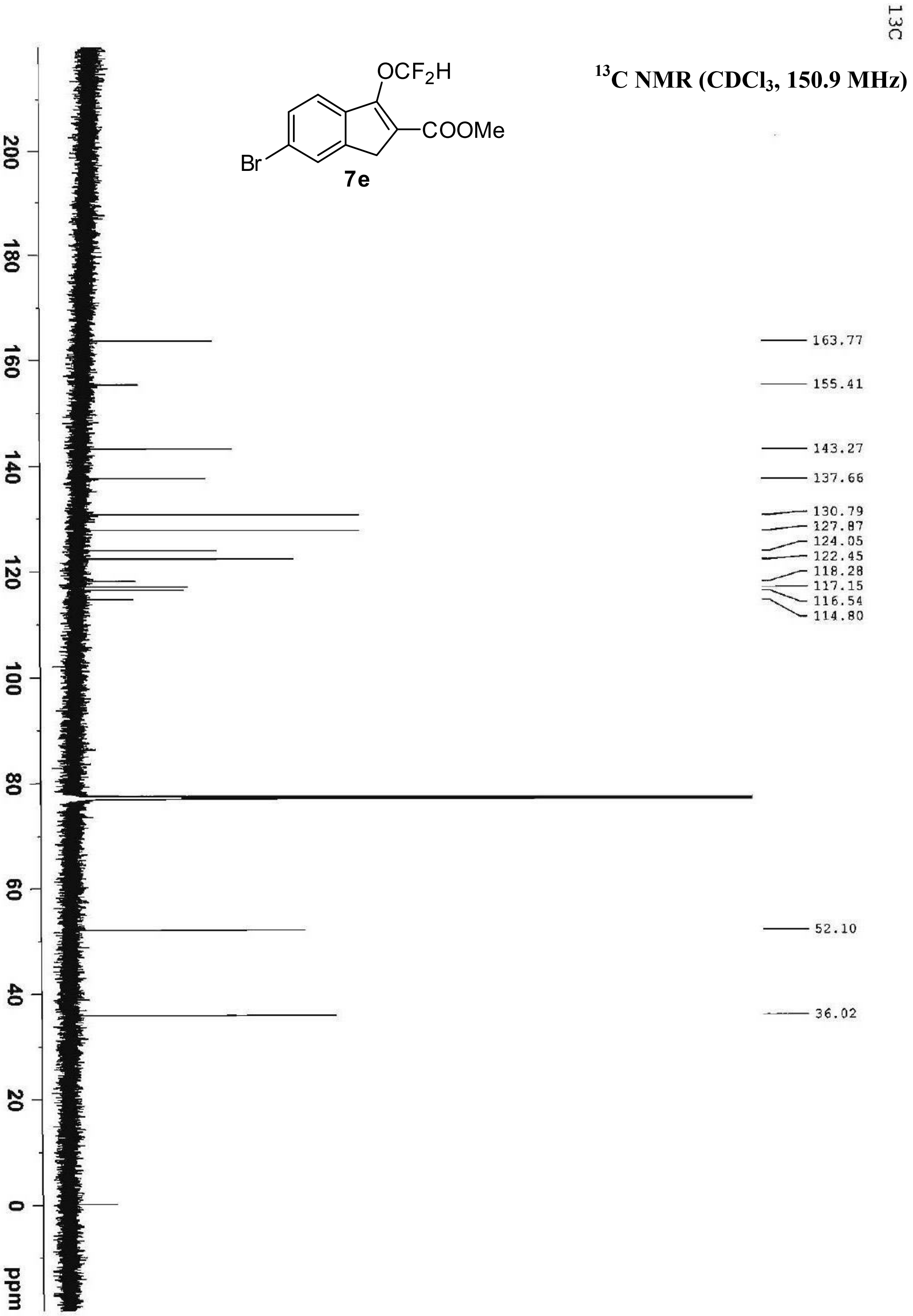
13C
^{13}C NMR ($CDCl_3$, 150.9 MHz)
OCF2H
COOMe
Br
7e
163.77
155.41
143.27
137.66
130.79
127.87
124.05
122.45
118.28
117.15
116.54
114.80
52.10
36.02
200
180
160
140
120
100
80
60
40
20
0
ppm

^{1}H NMR (CDCl$_3$, 300 MHz)

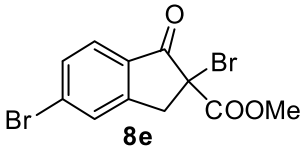

8e

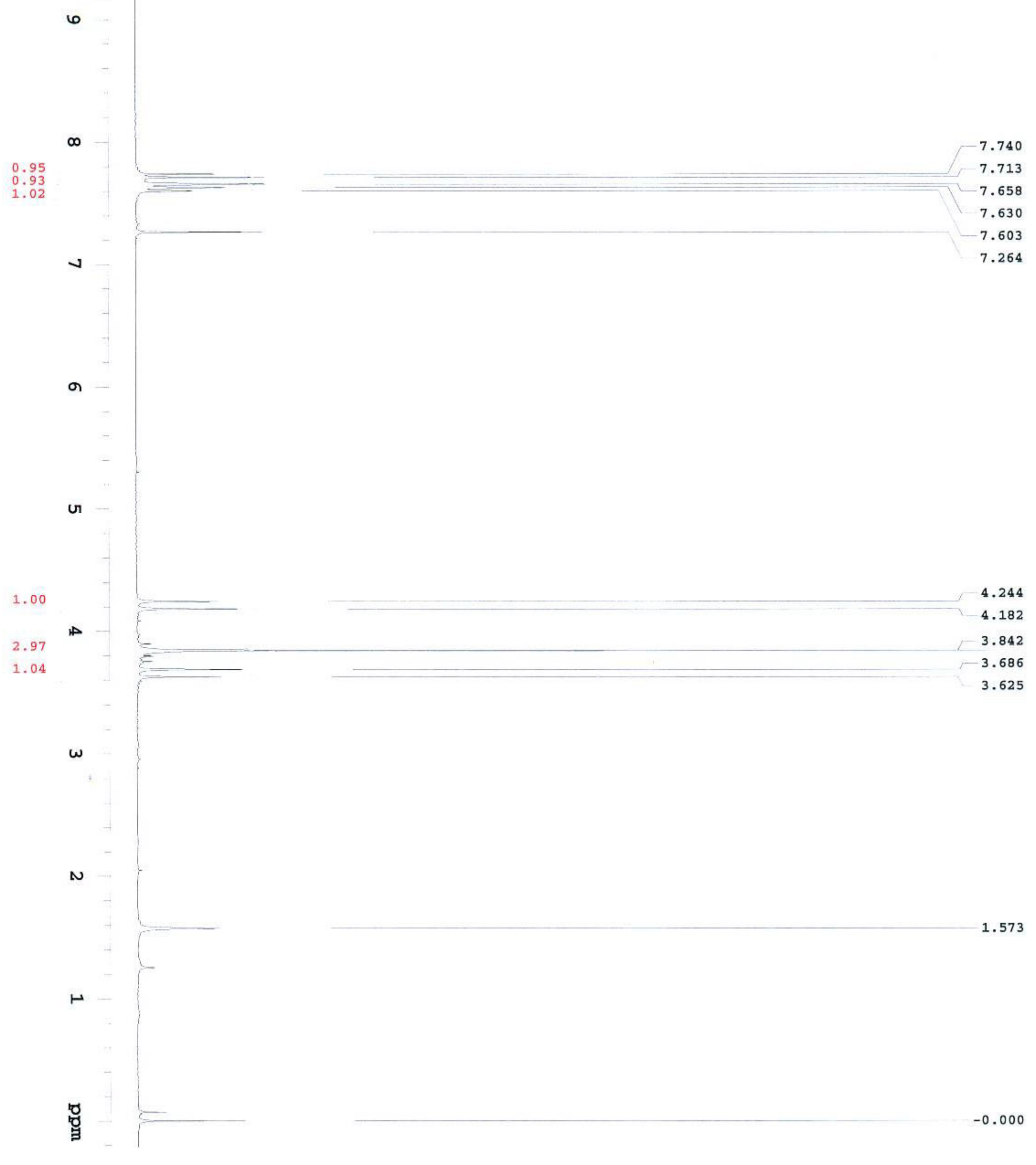

^{13}C NMR ($CDCl_3$, 150.9 MHz)

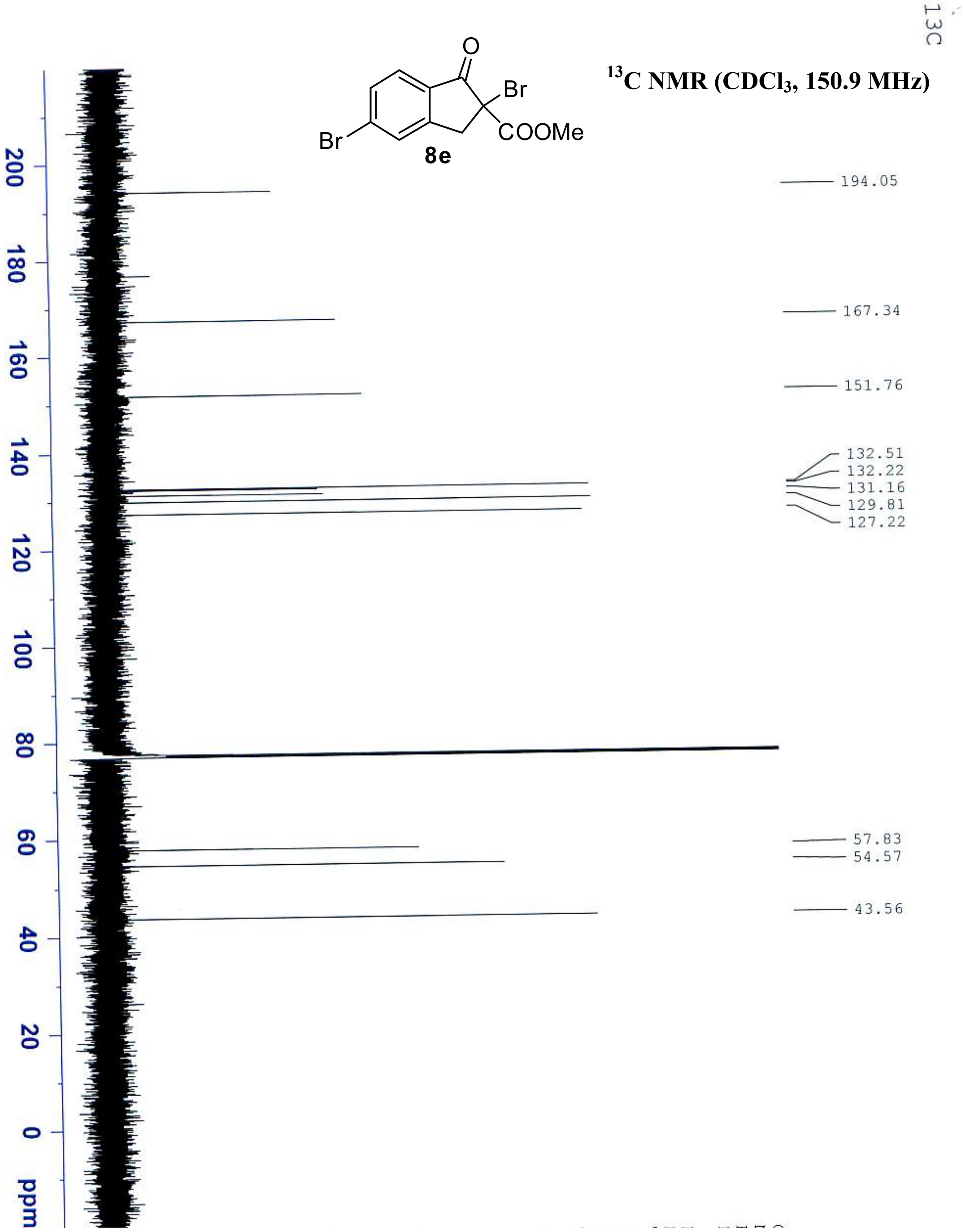

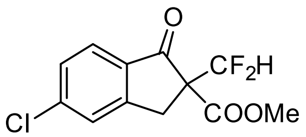

6f

^{1}H NMR (CDCl$_3$, 300 MHz)

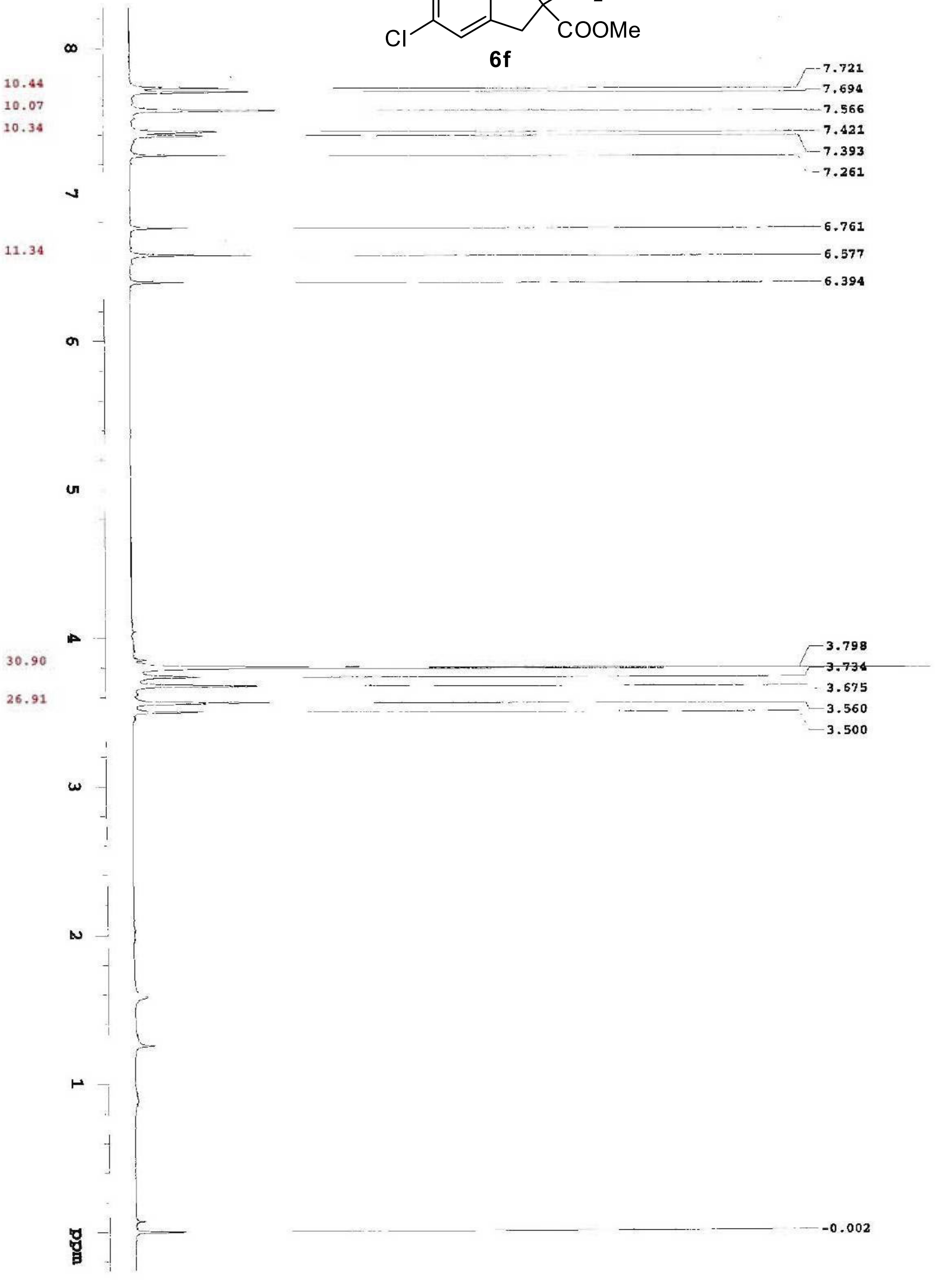

^{19}F NMR ($CDCl_3$, 282 MHz)

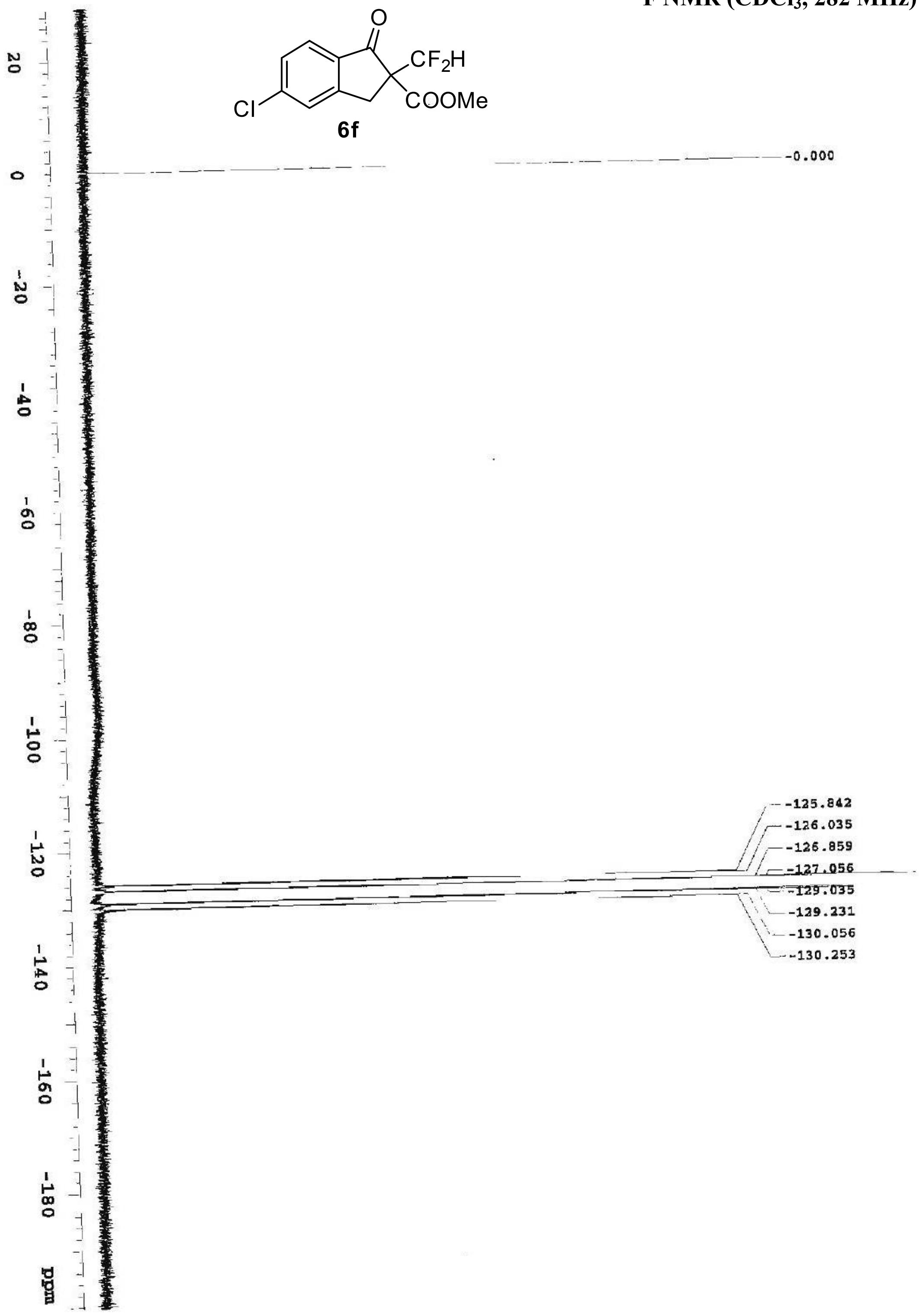

^{13}C NMR ($CDCl_3$, 150.9 MHz)

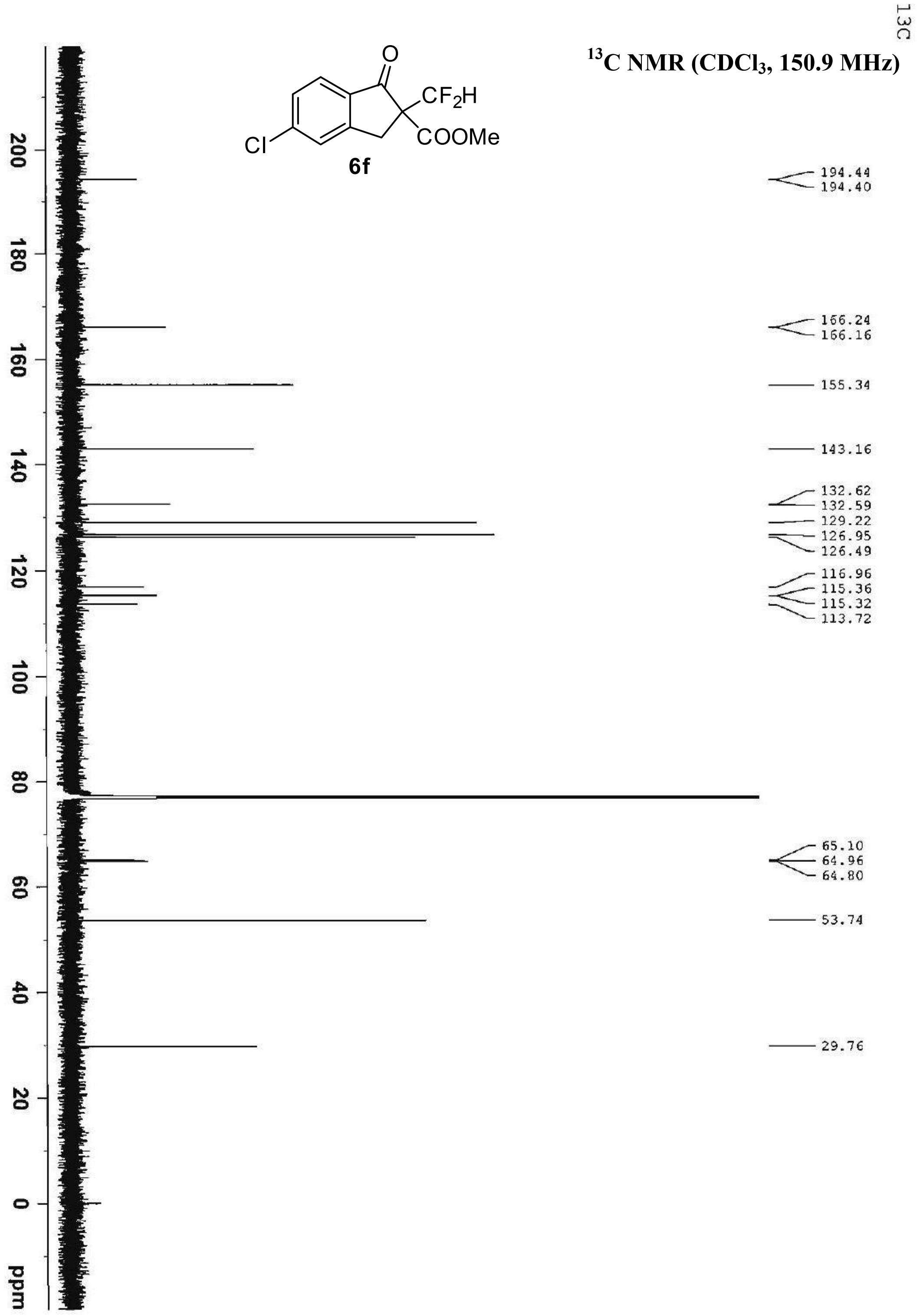

^{1}H NMR ($CDCl_3$, 300 MHz)

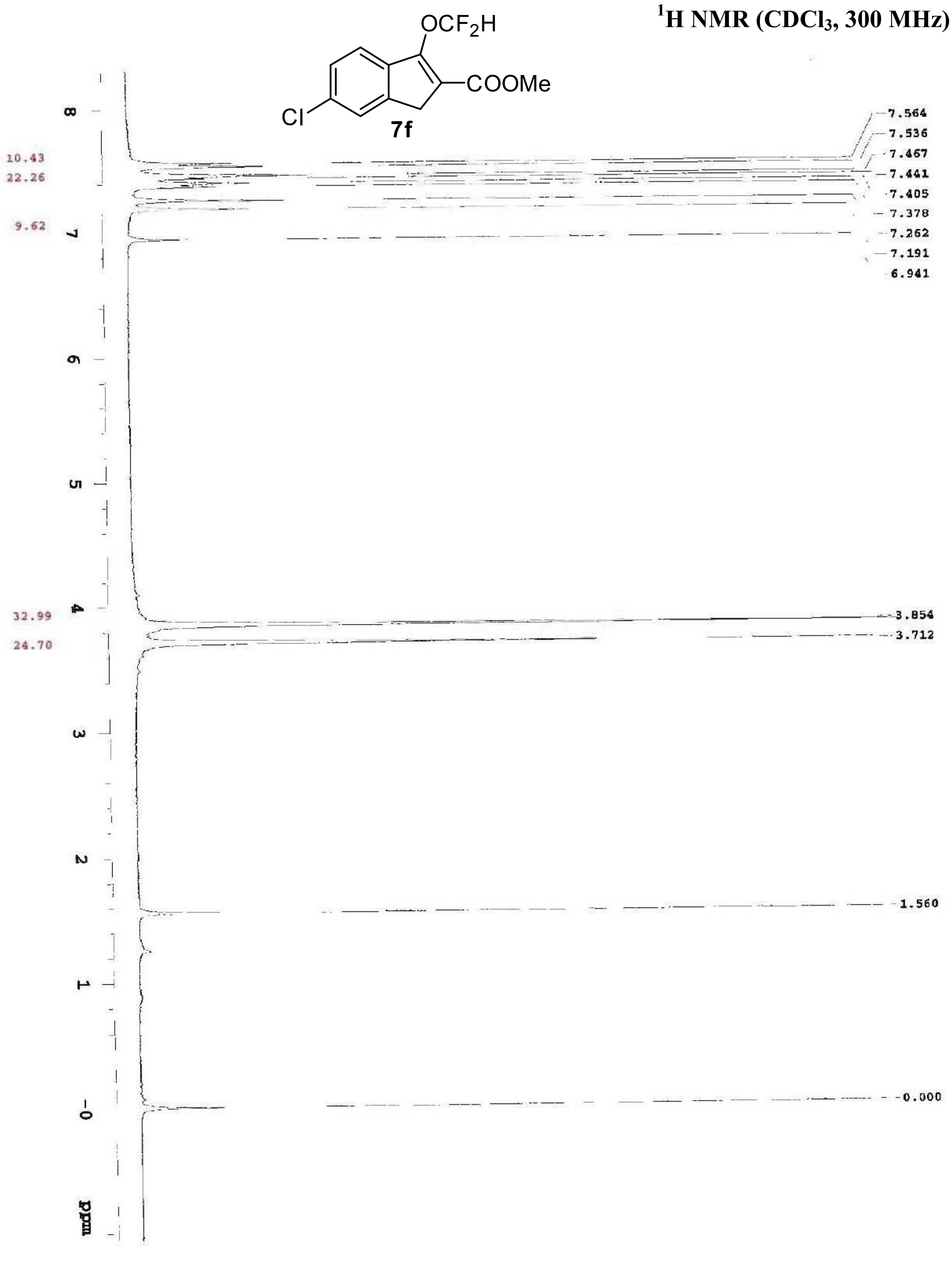

^{19}F NMR ($CDCl_3$, 282 MHz)

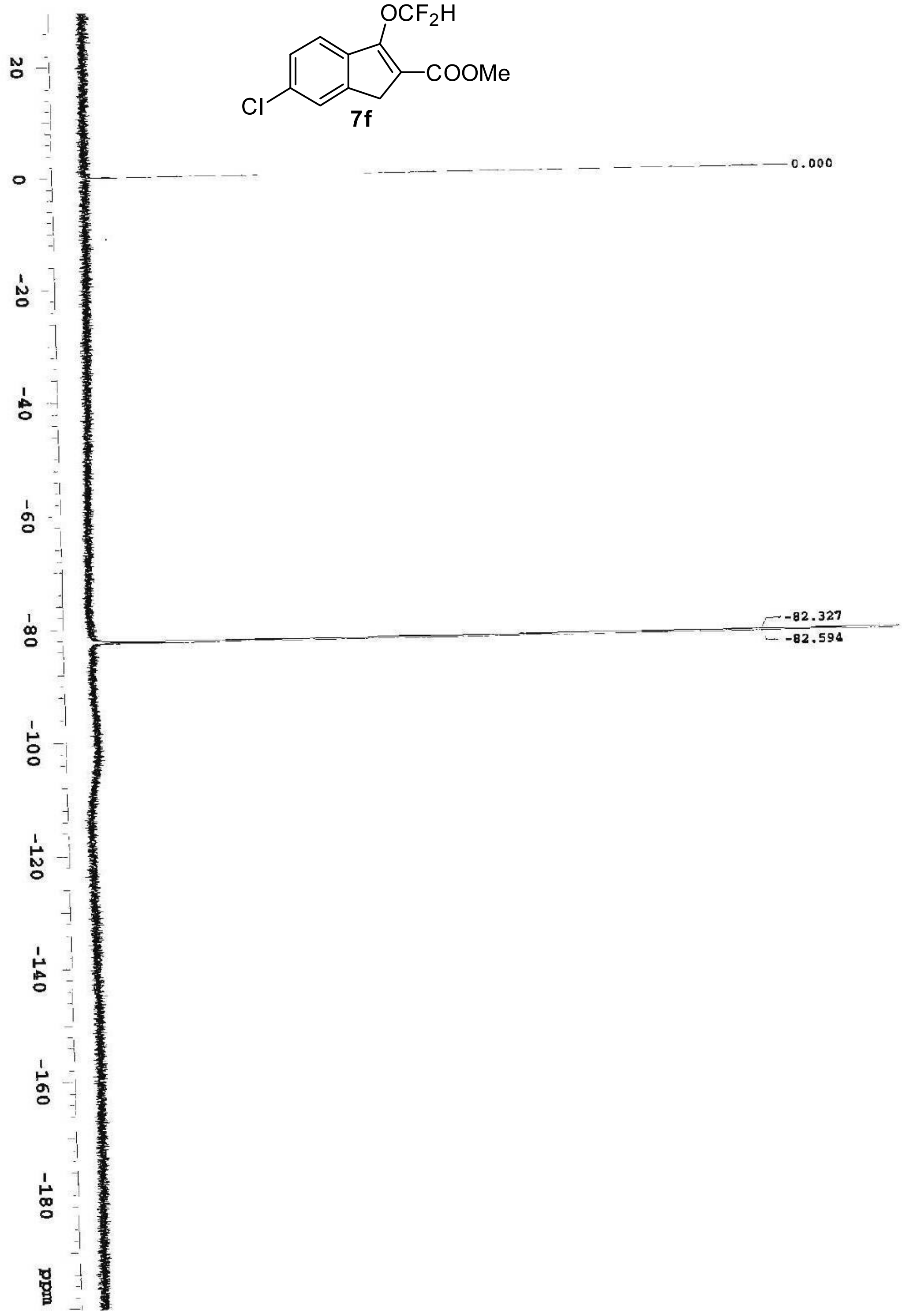

^{13}C NMR ($CDCl_3$, 150.9 MHz)

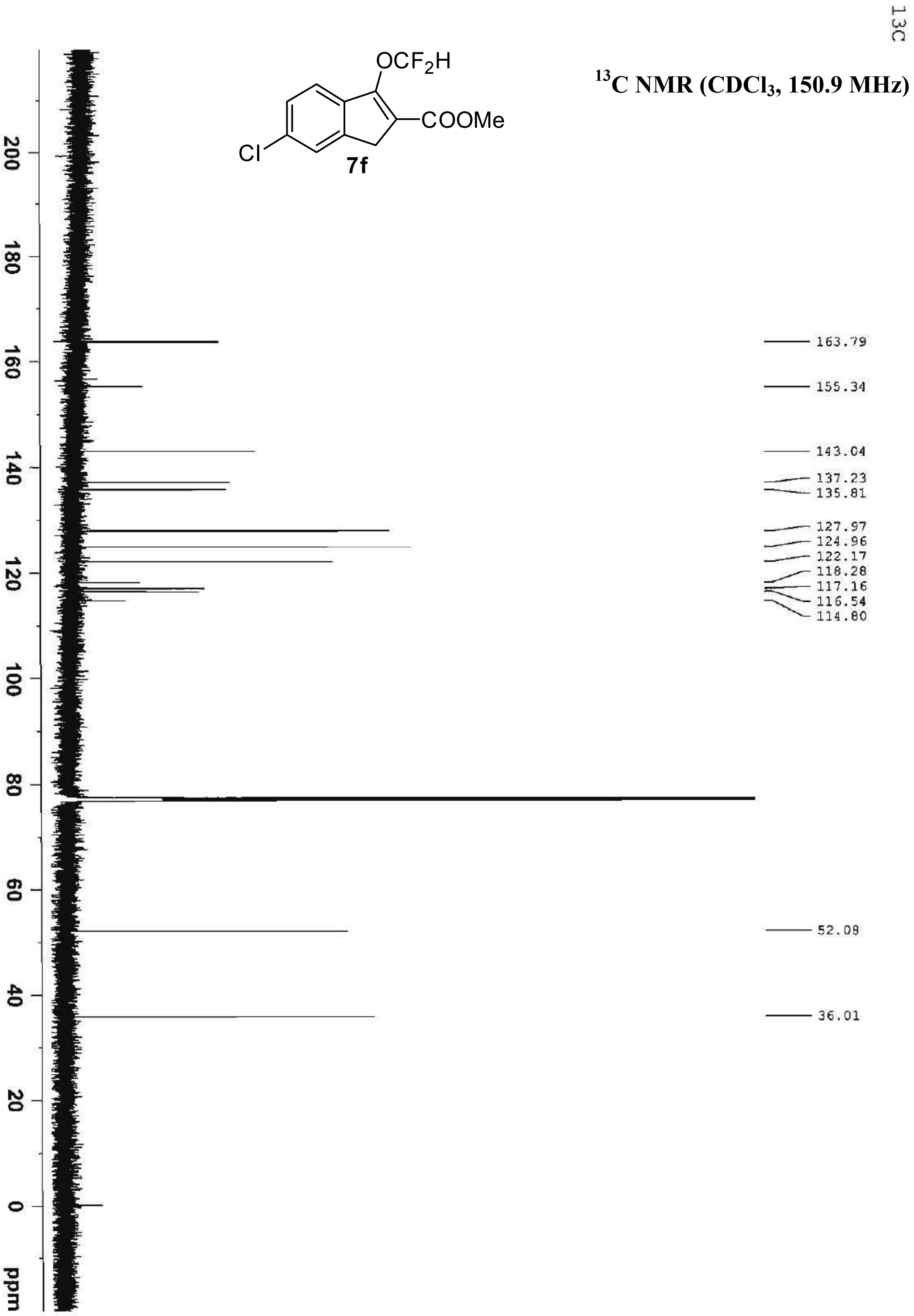

^{1}H NMR ($CDCl_3$, 300 MHz)

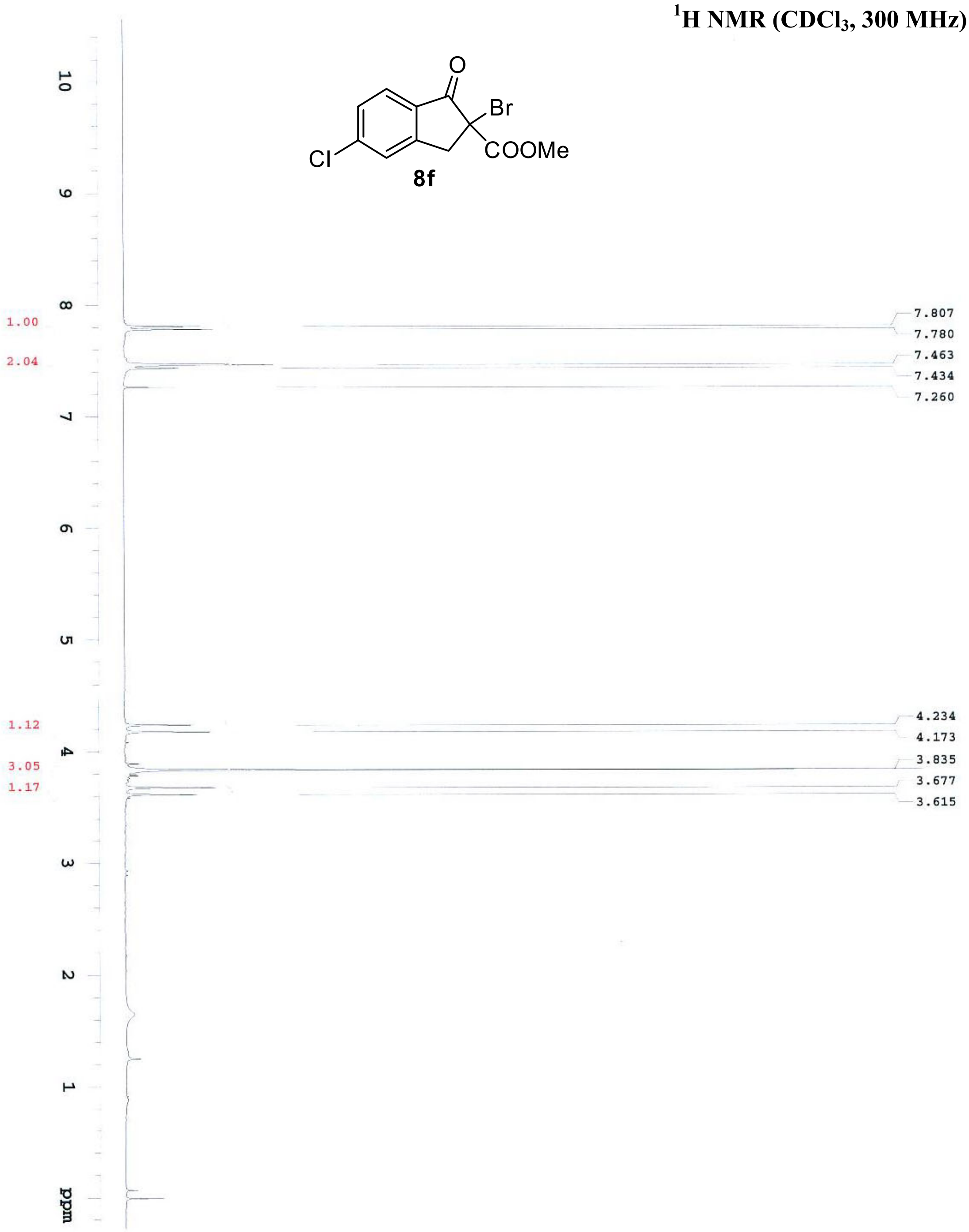

8f

^{13}C NMR ($CDCl_3$, 150.9 MHz)

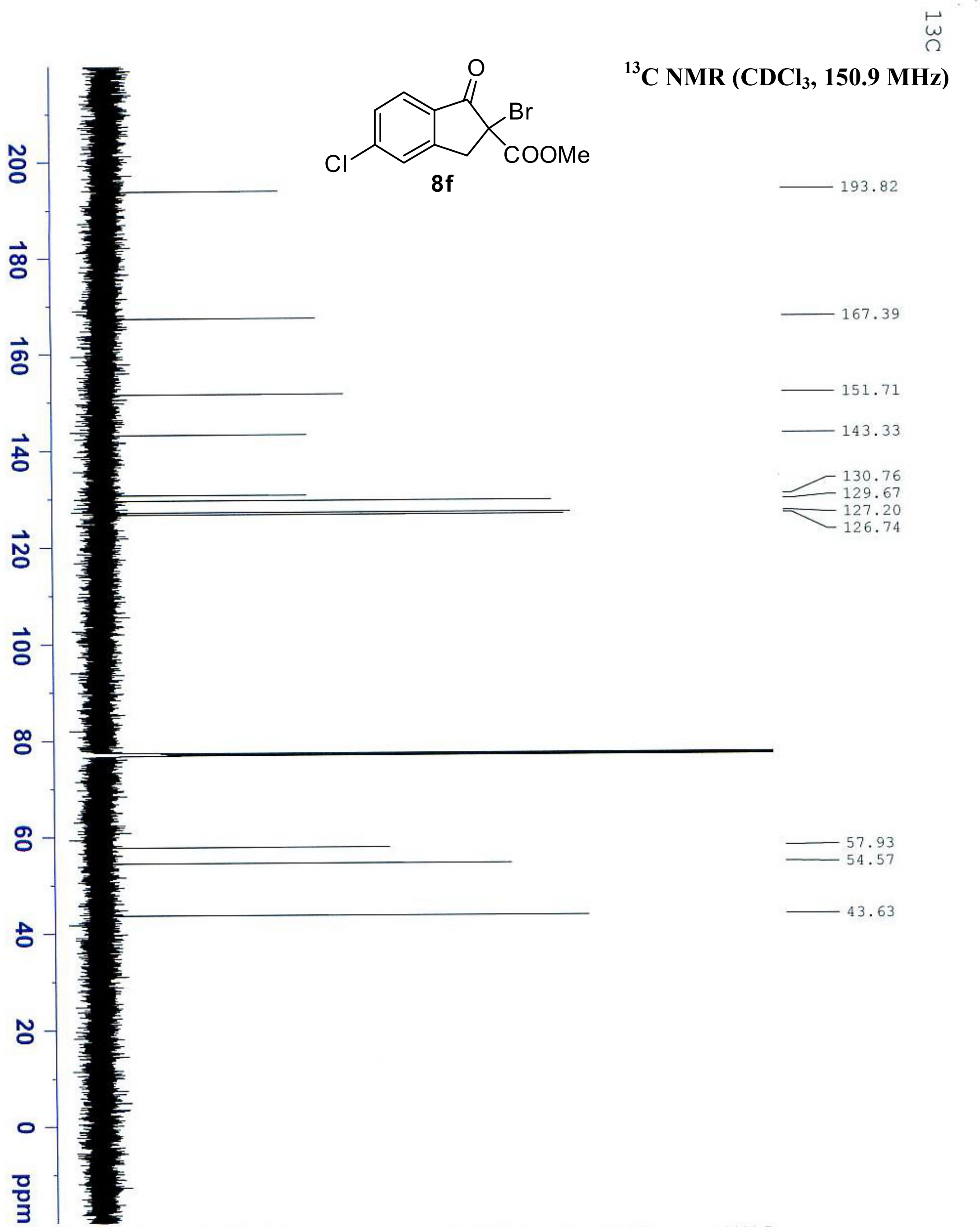

^{1}H NMR ($CDCl_3$, 300 MHz)

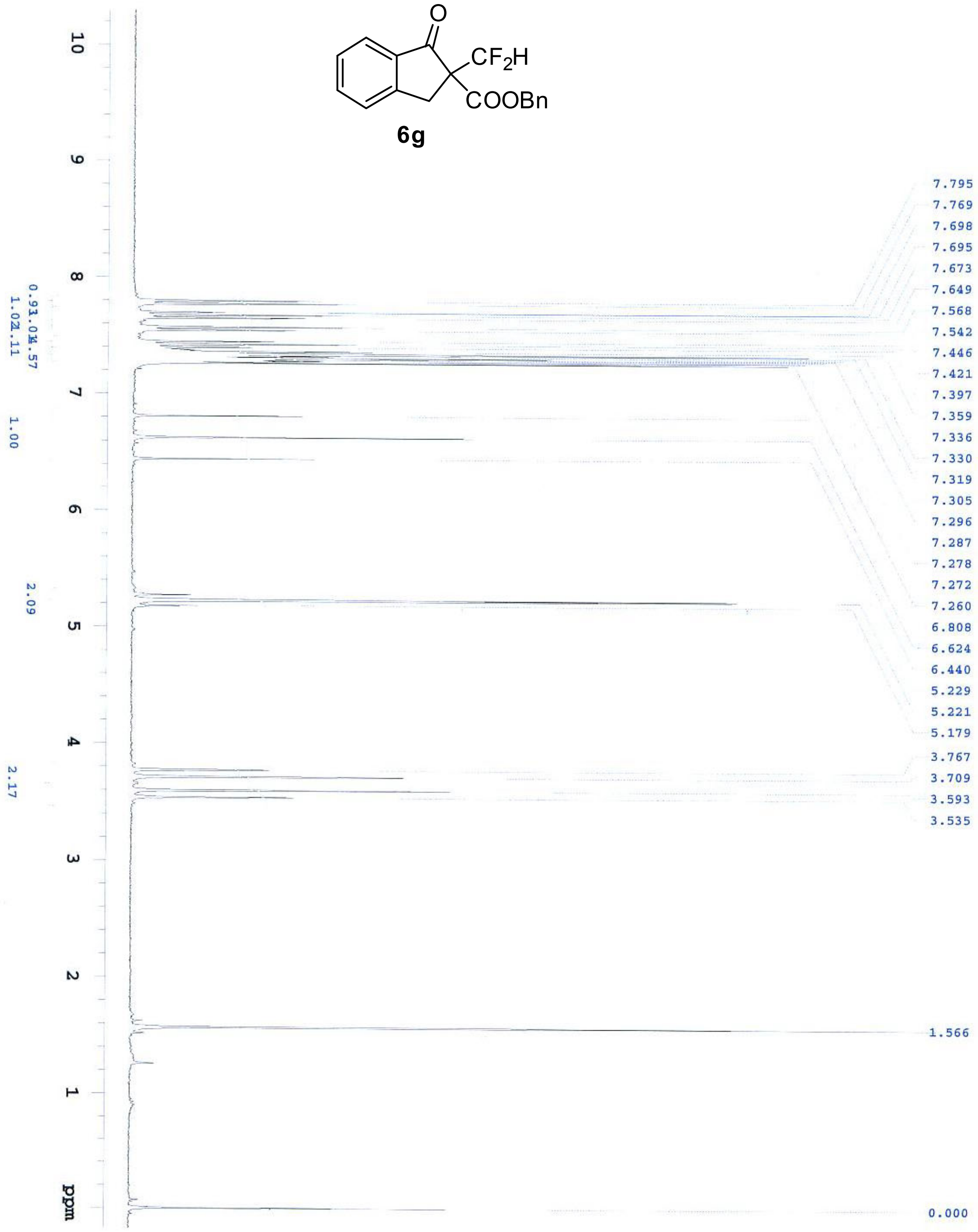

^{19}F NMR ($CDCl_3$, 282 MHz)

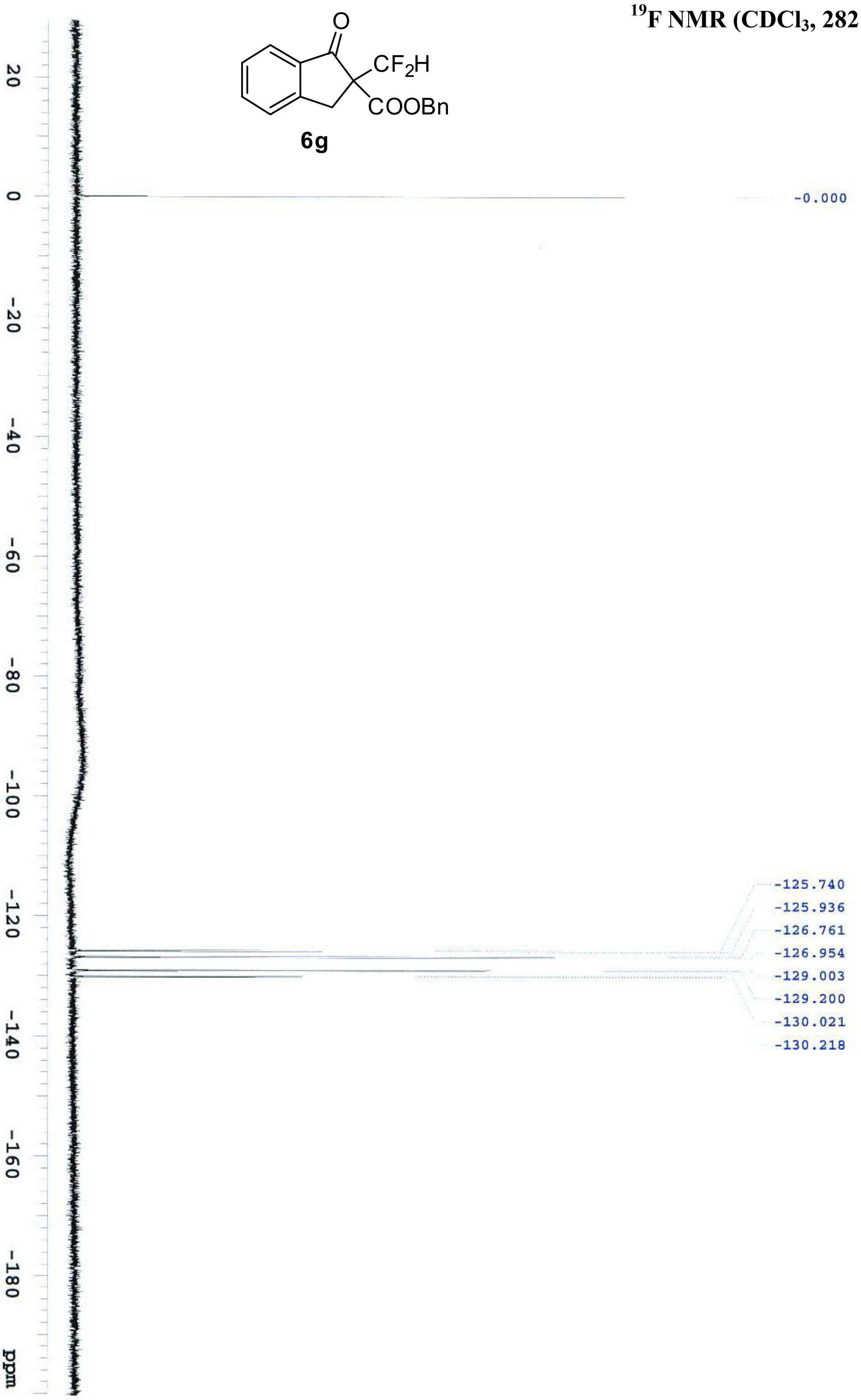

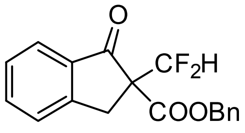

6g

^{13}C NMR ($CDCl_3$, 150.9 MHz)

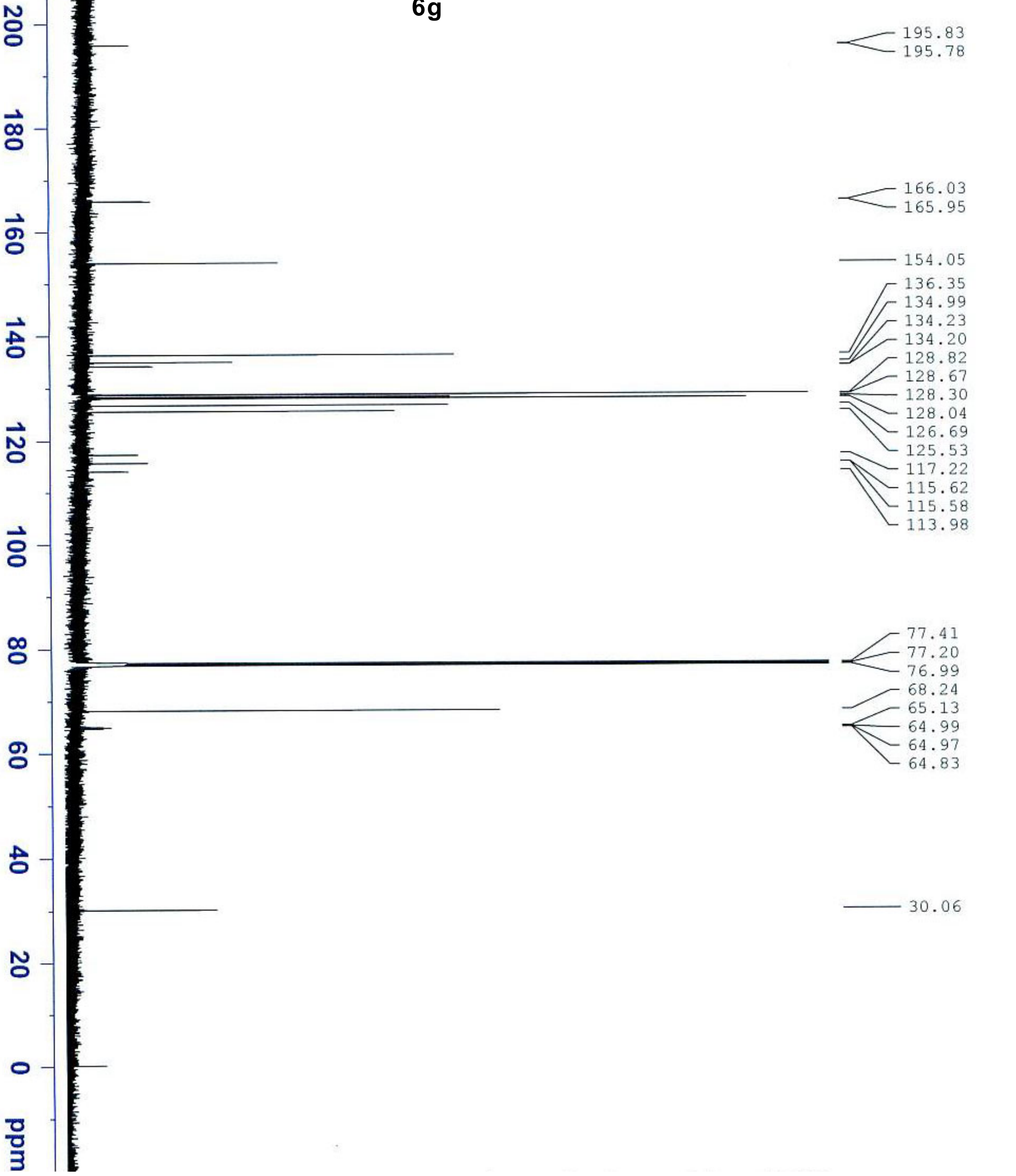

^{1}H NMR ($CDCl_3$, 300 MHz)

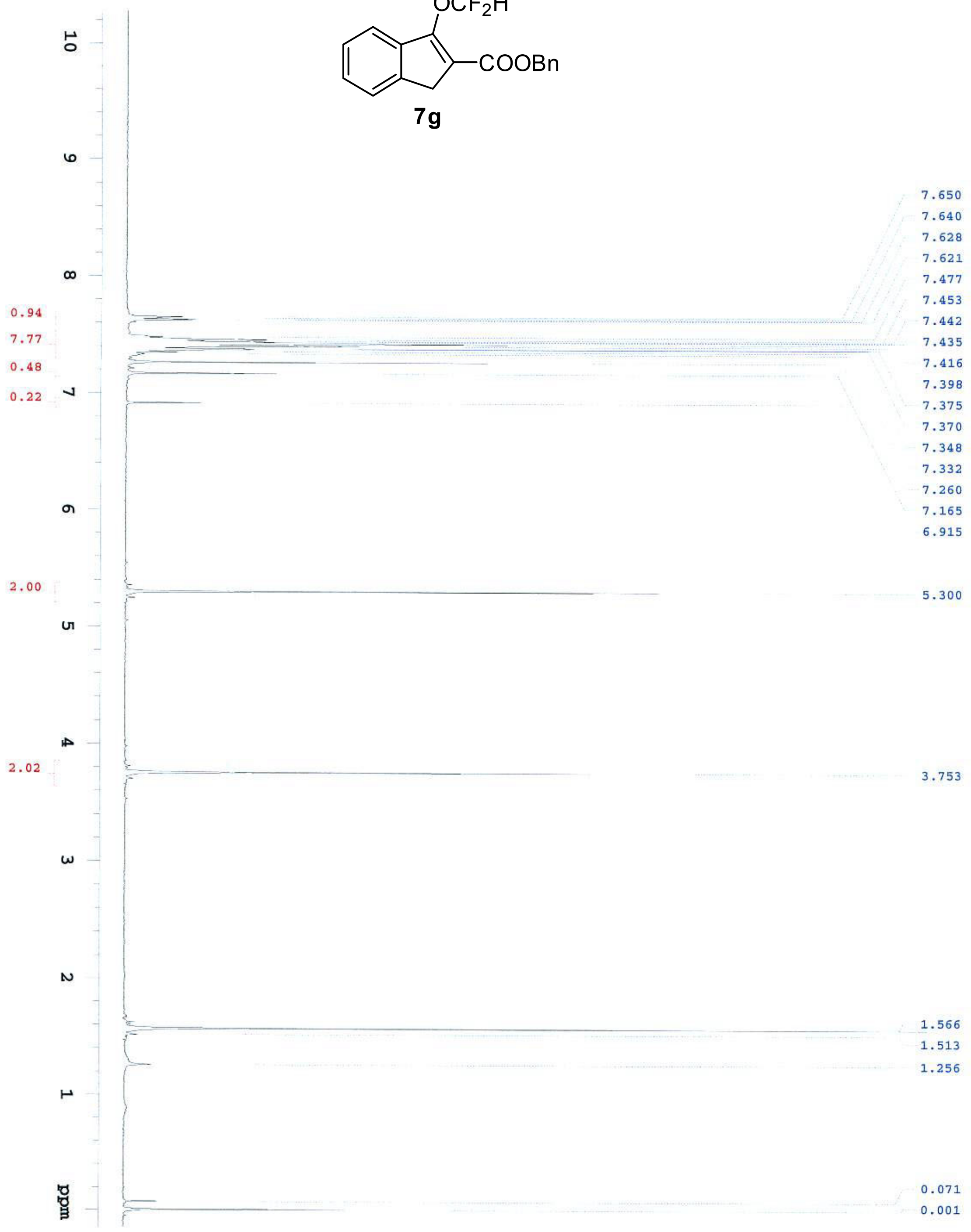

^{19}F NMR ($CDCl_3$, 282 MHz)

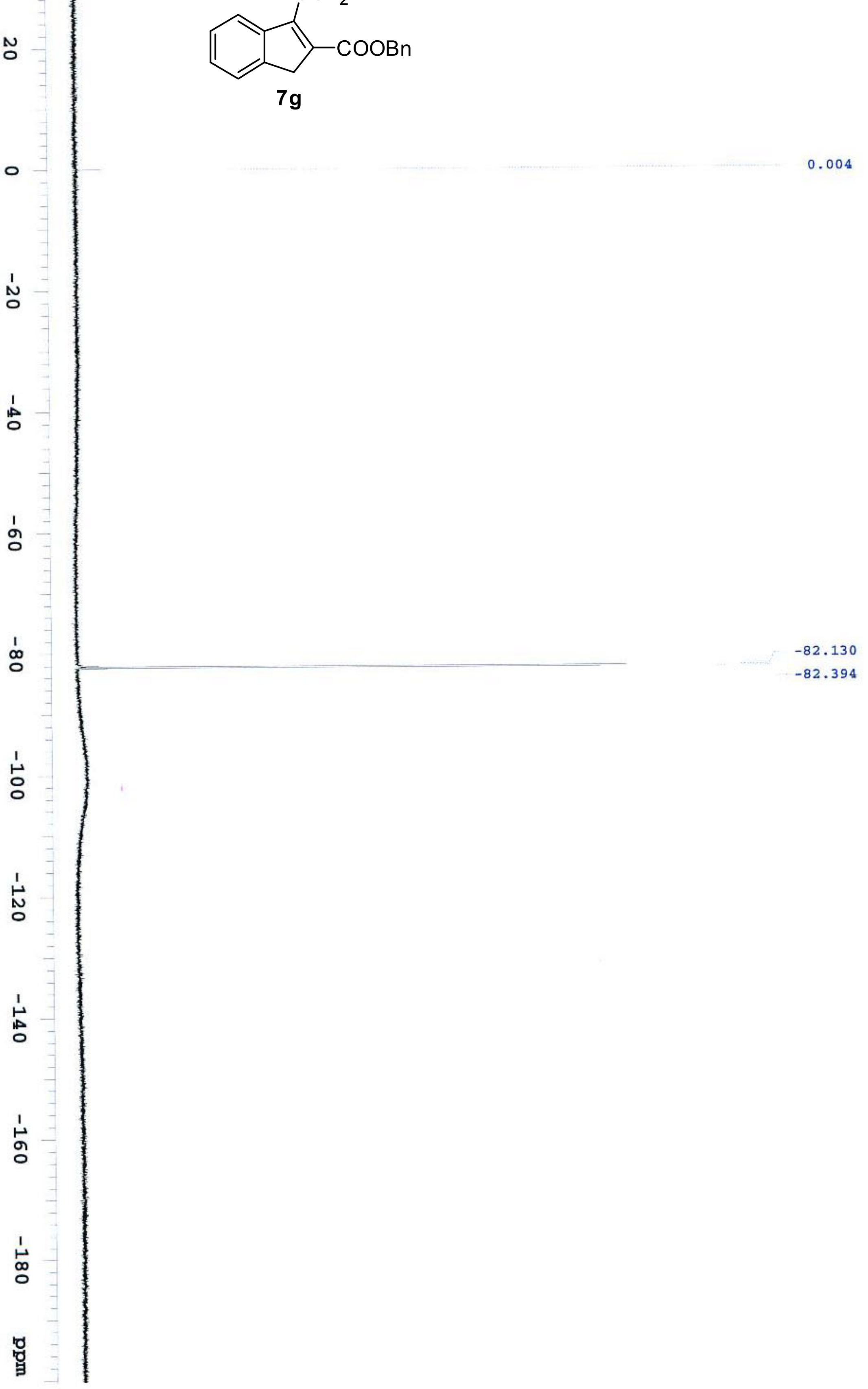

^{13}C NMR (CDCl$_3$, 150.9 MHz)

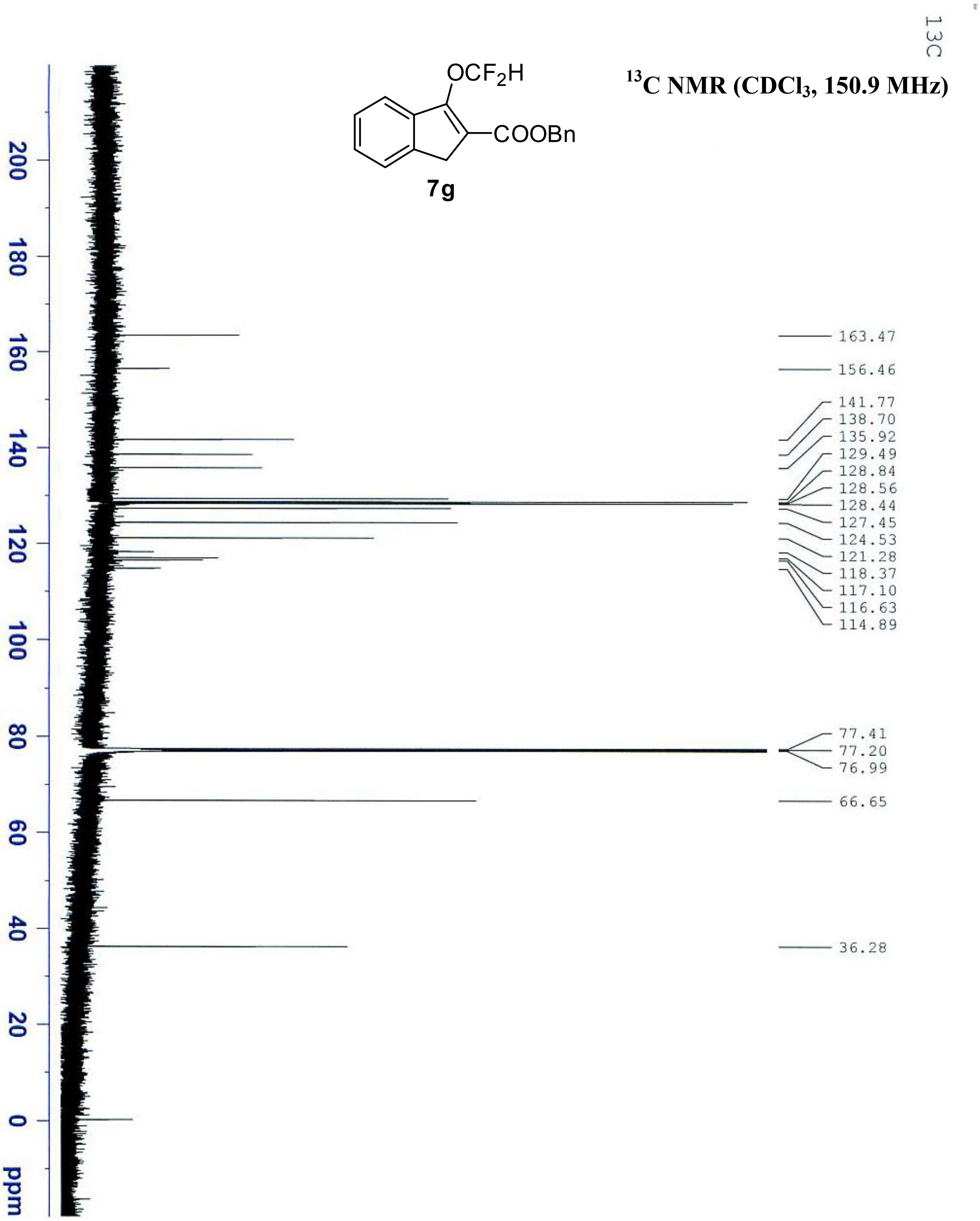

^{1}H NMR ($CDCl_3$, 300 MHz)

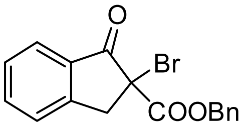

8g

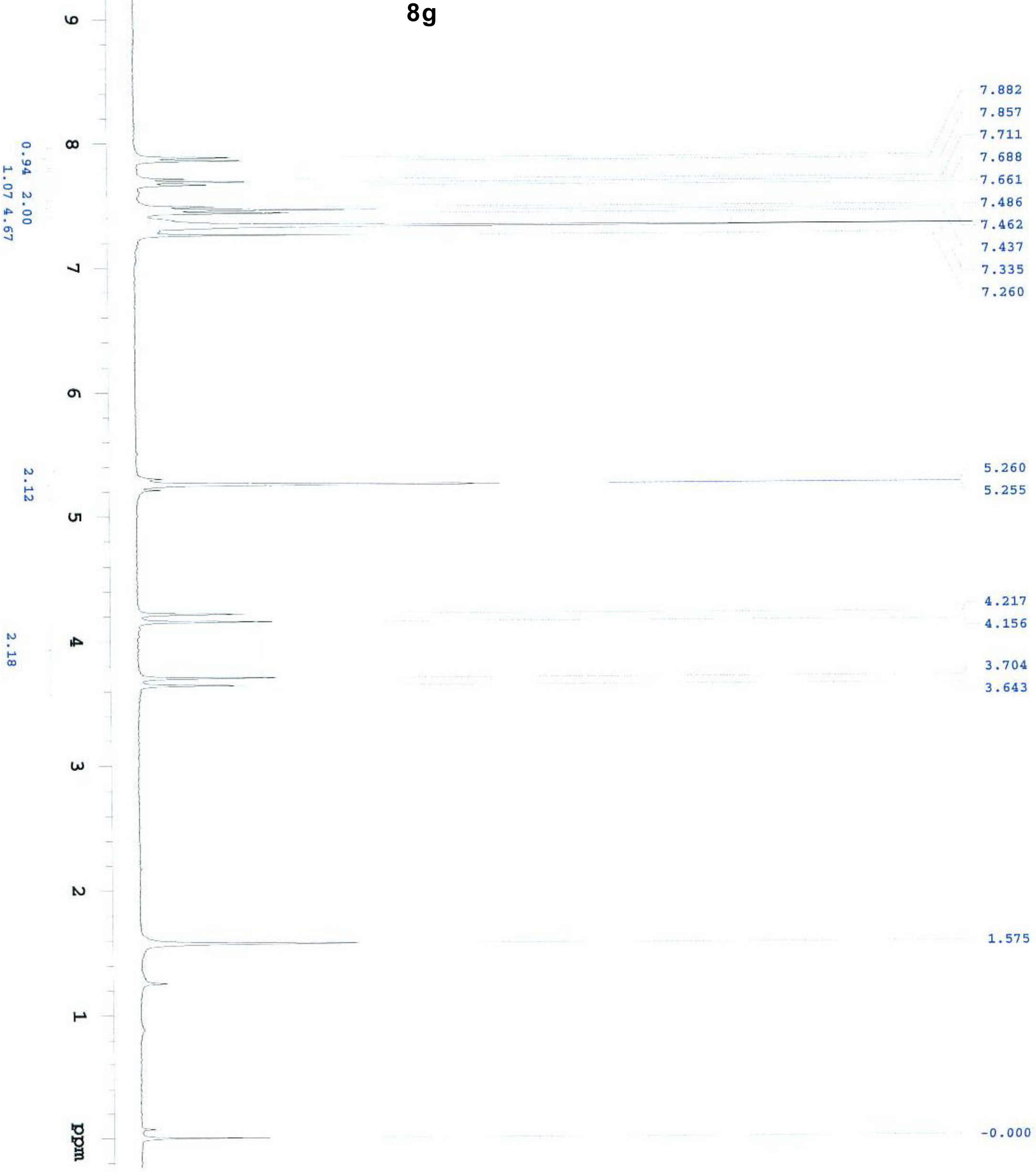

^{13}C NMR ($CDCl_3$, 150.9 MHz)

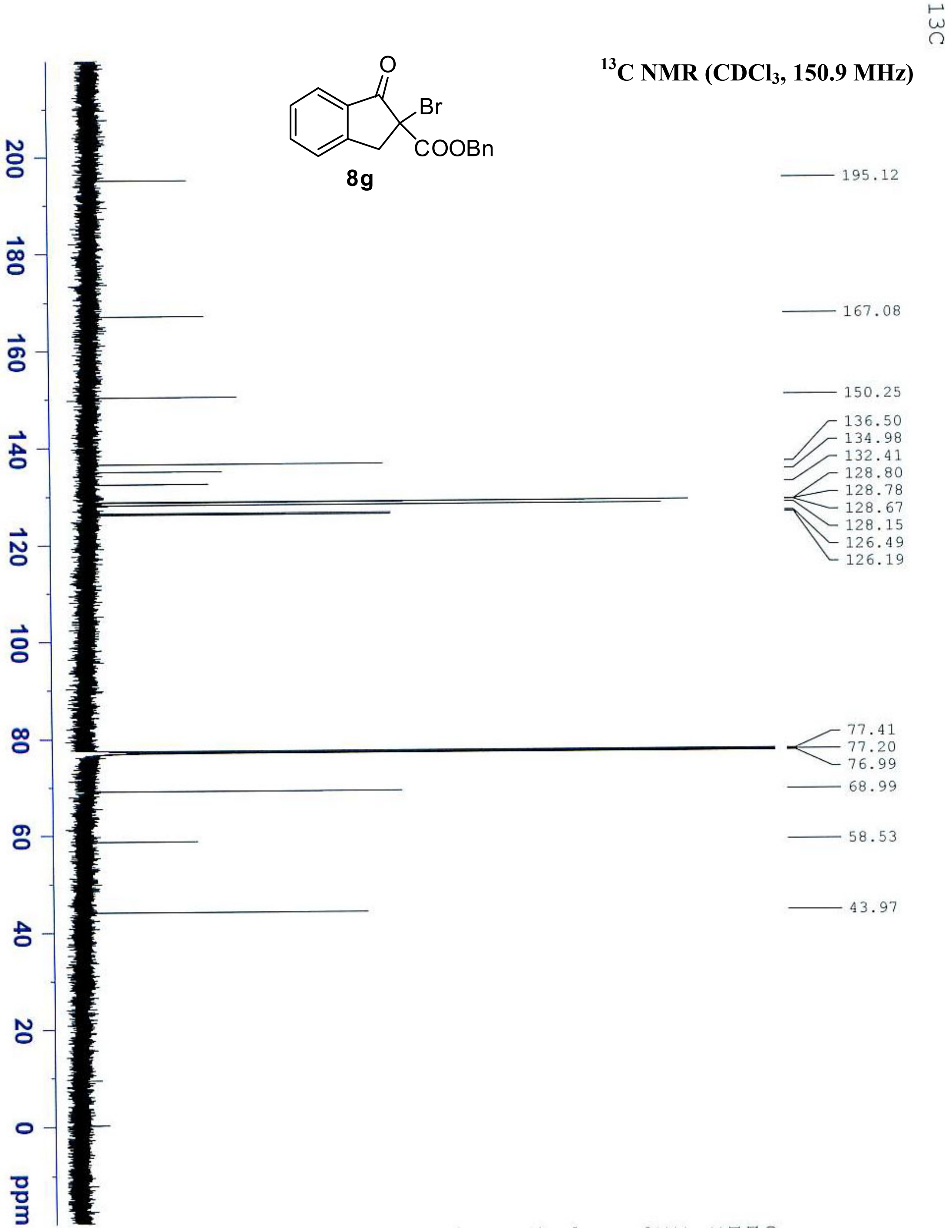

^{1}H NMR (CDCl$_3$, 300 MHz)

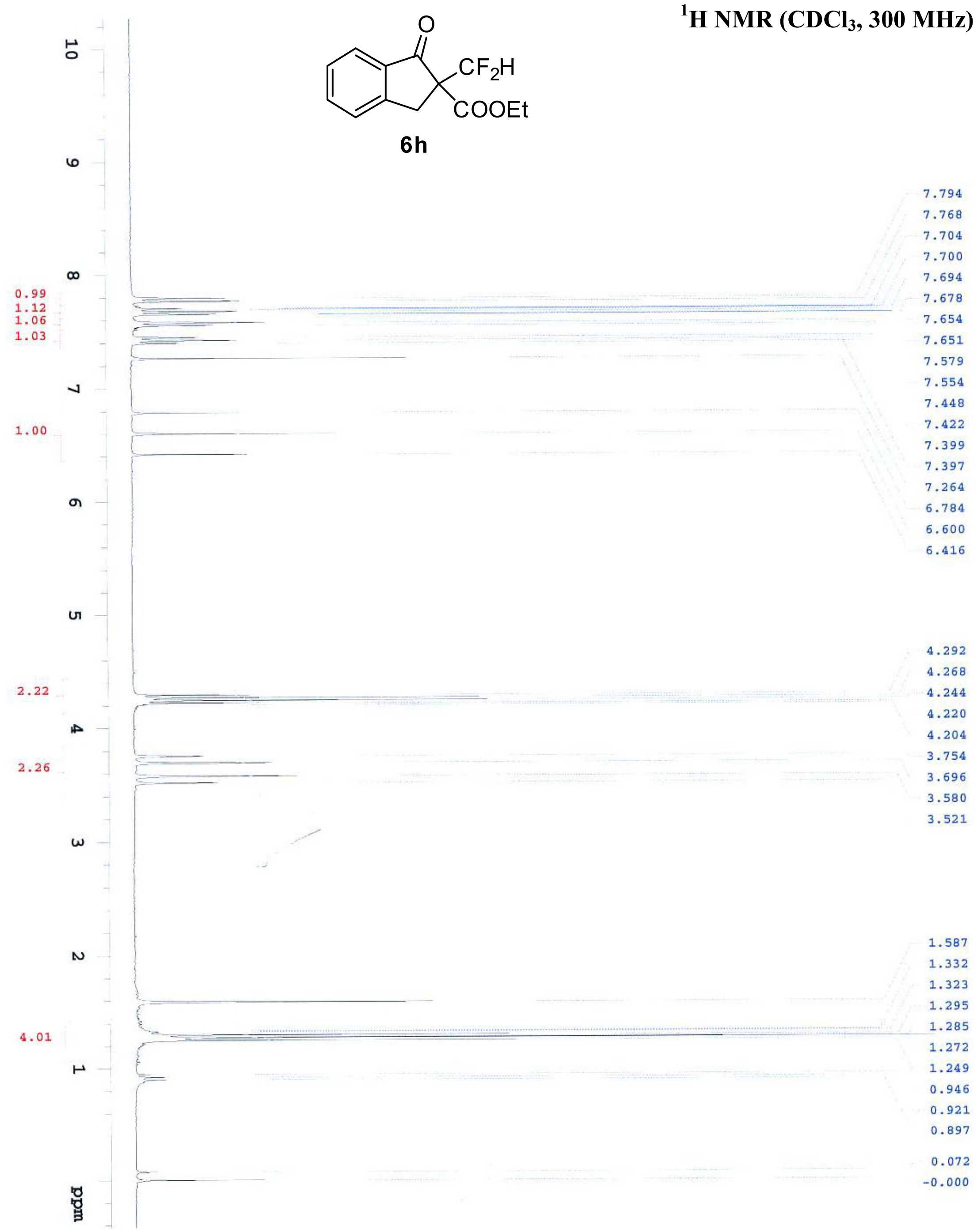

^{19}F NMR ($CDCl_3$, 282 MHz)

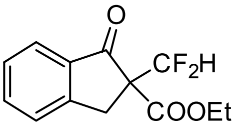

6h

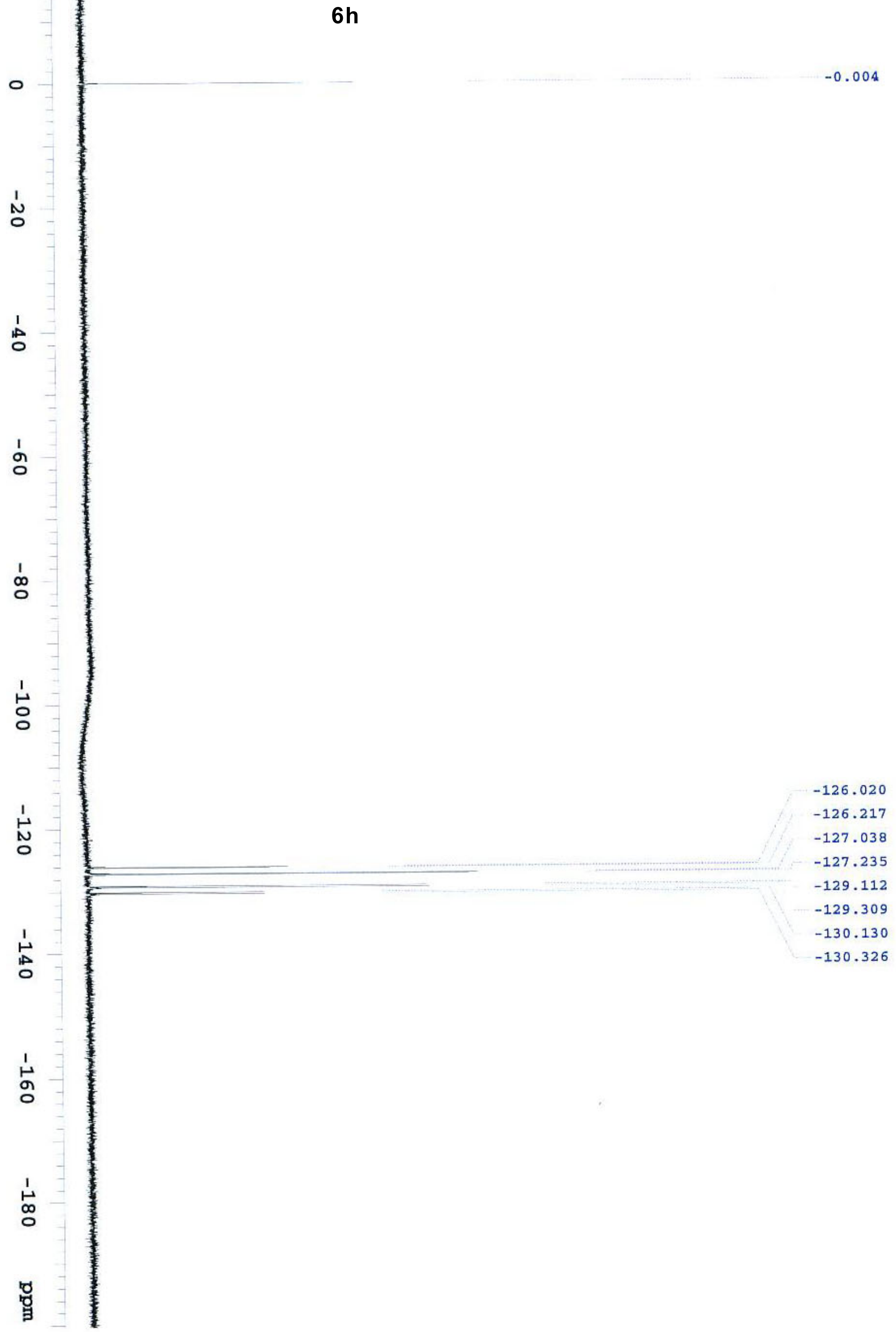

^{13}C NMR ($CDCl_3$, 150.9 MHz)

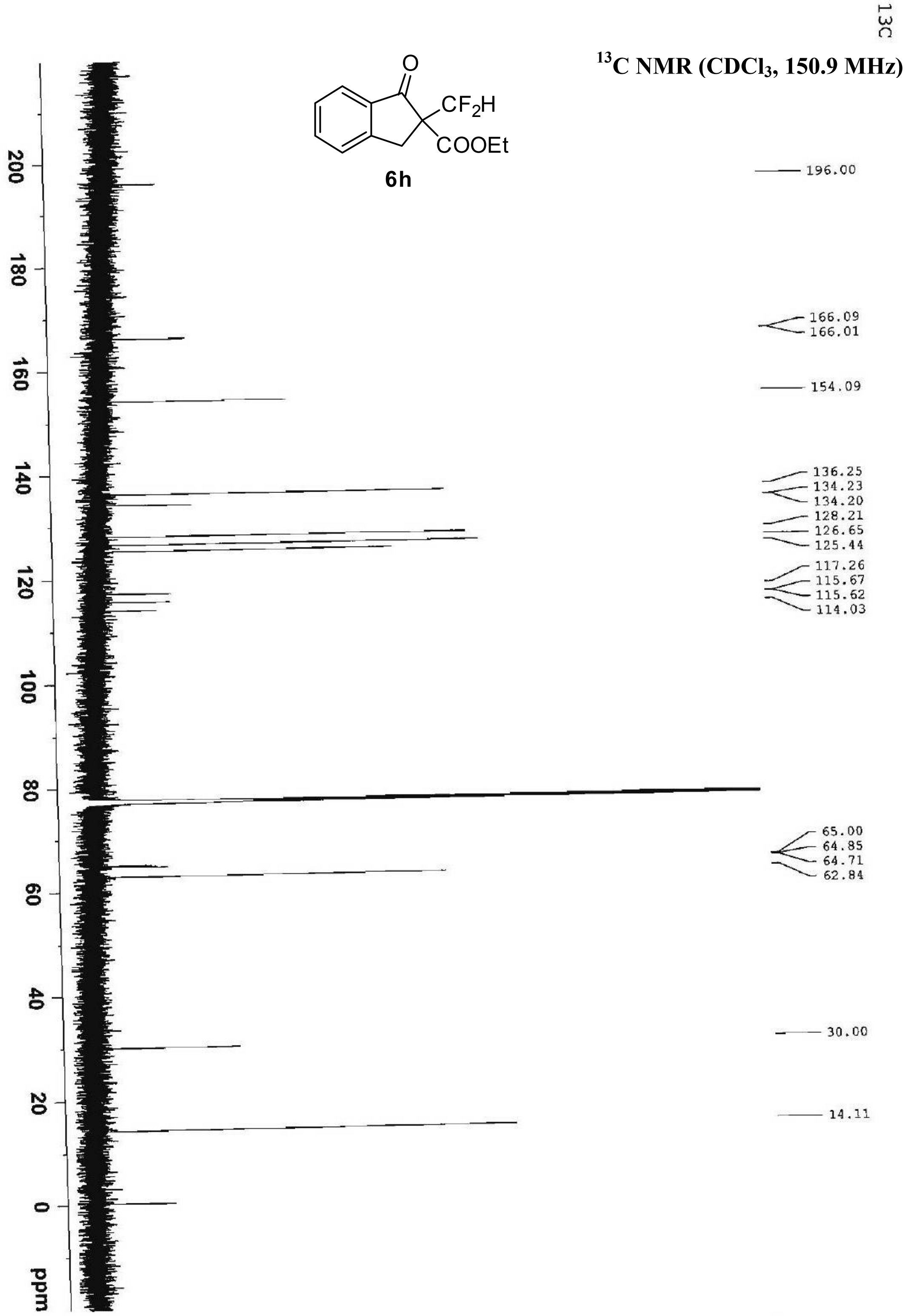

^{1}H NMR ($CDCl_3$, 300 MHz)

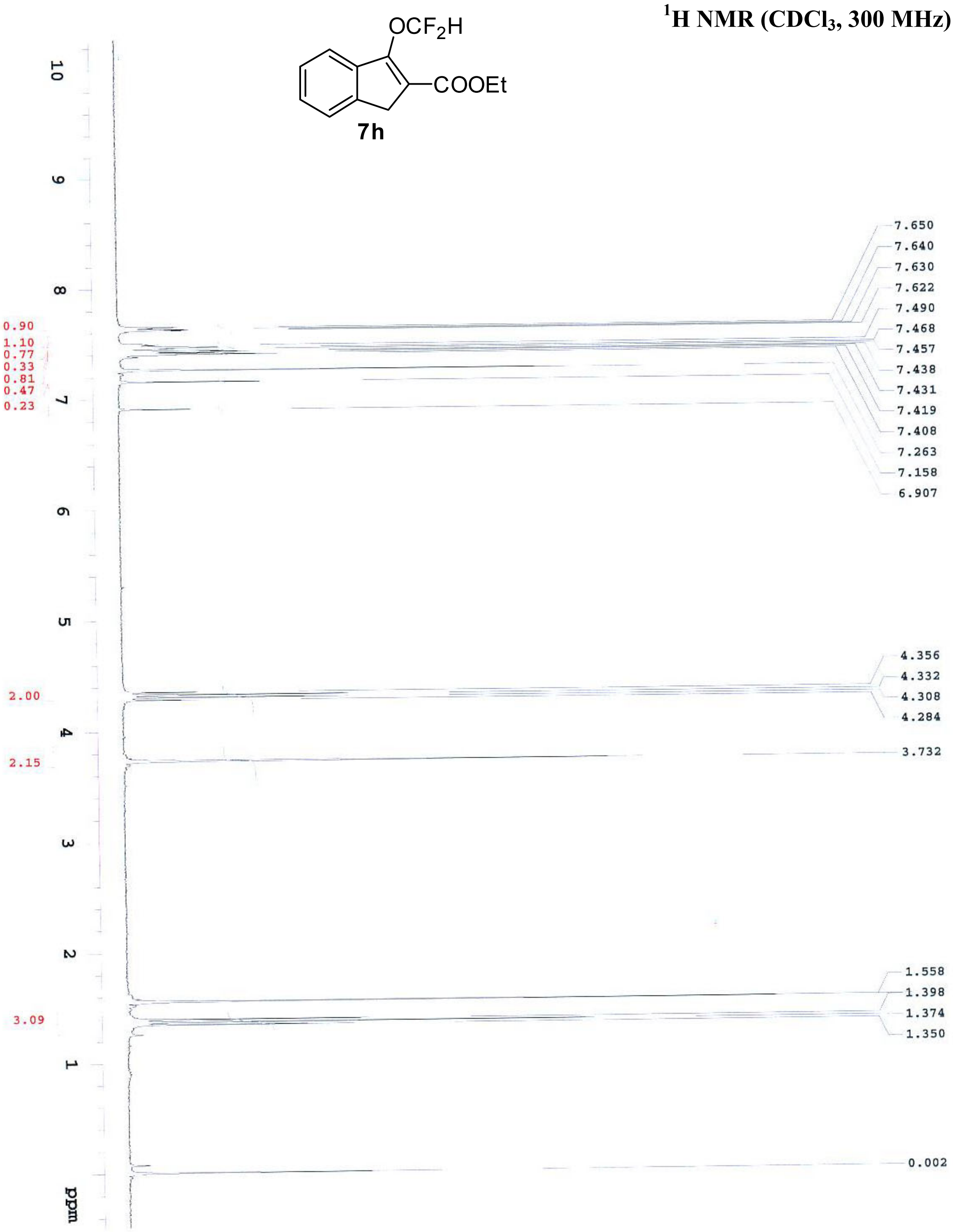

^{19}F NMR ($CDCl_3$, 282 MHz)

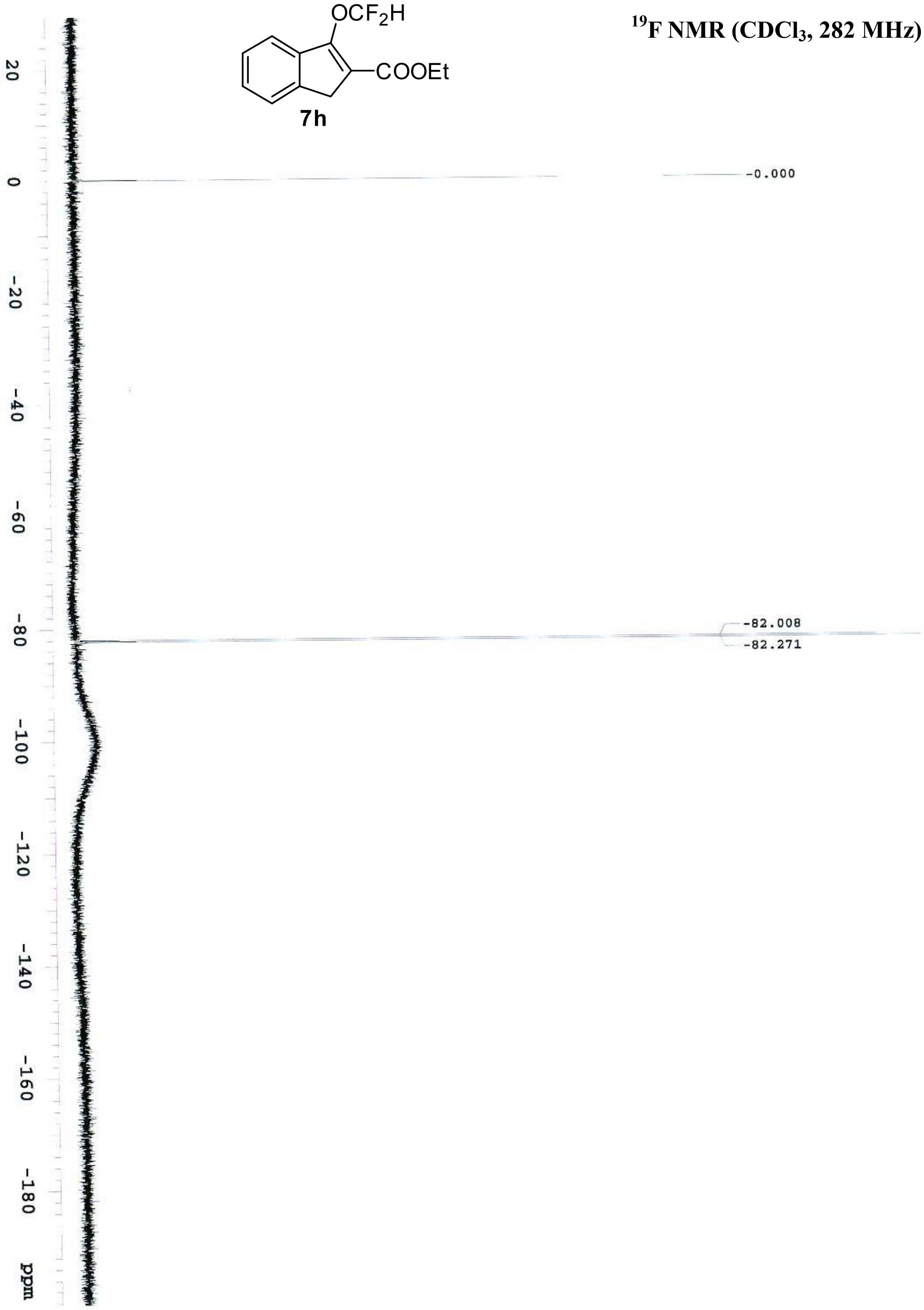

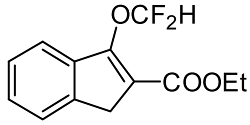

7h

^{13}C NMR (CDCl$_3$, 150.9 MHz)

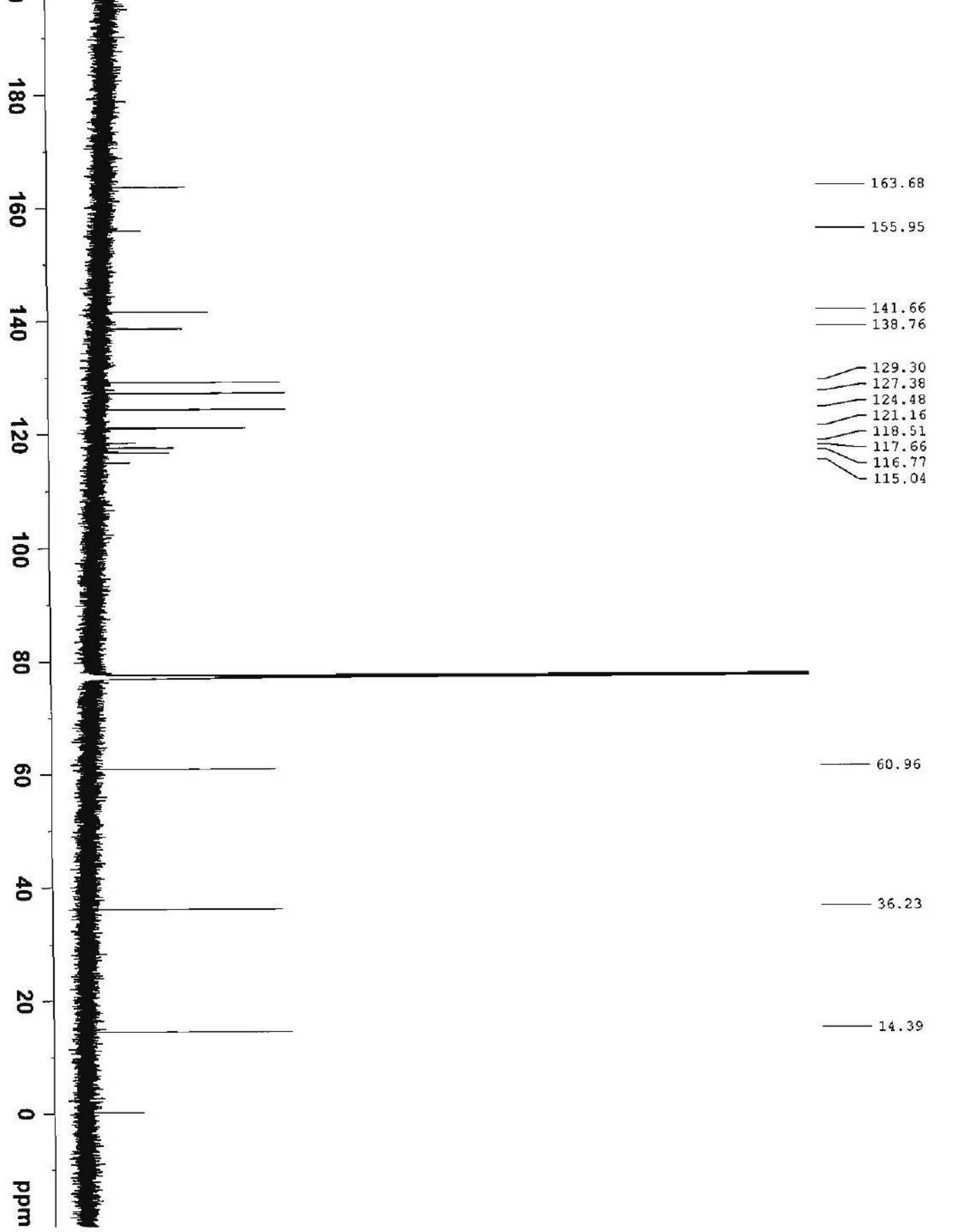

^{1}H NMR ($CDCl_3$, 300 MHz)

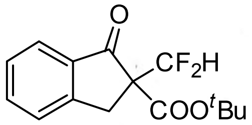

6i

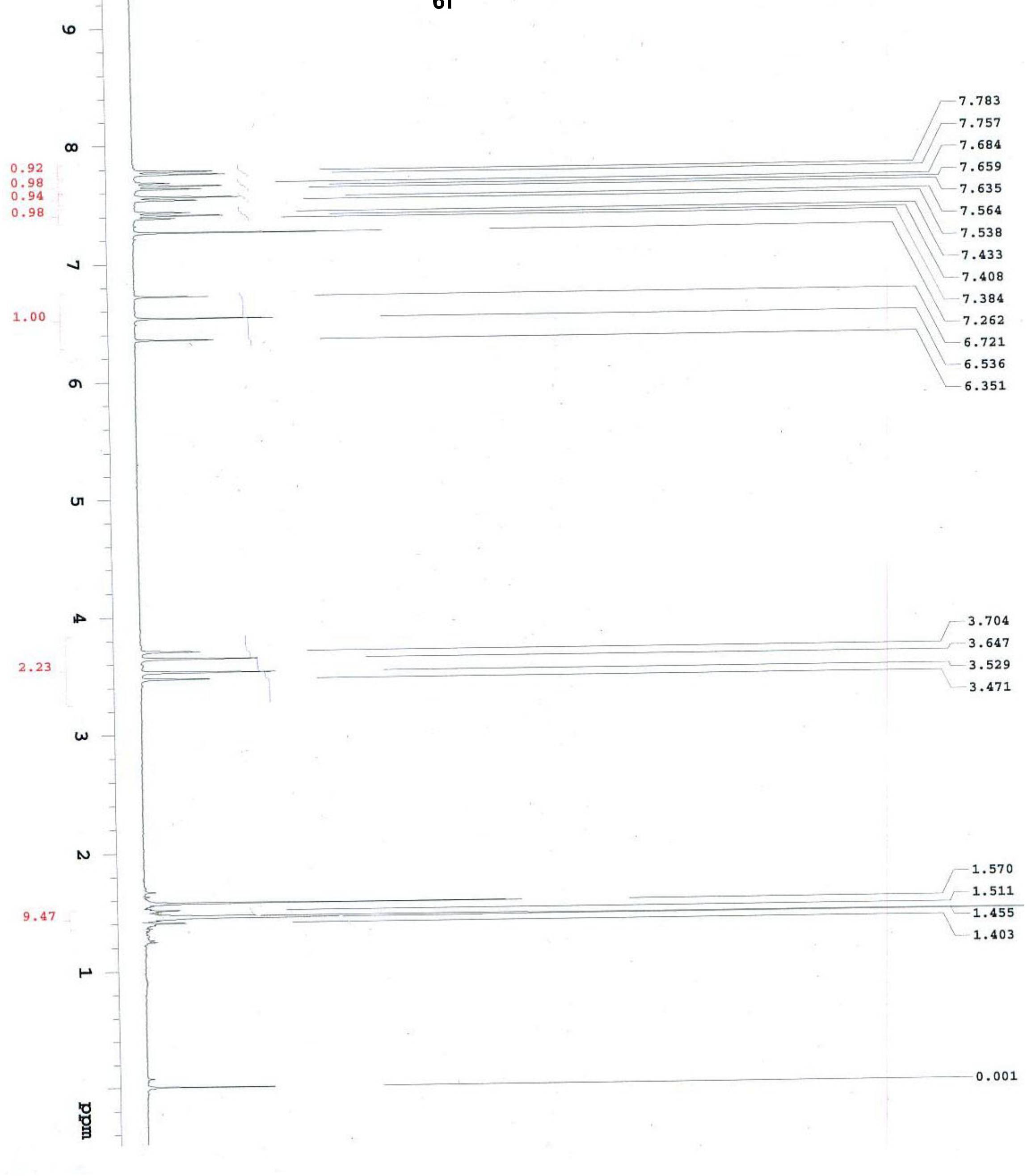

^{19}F NMR (CDCl$_3$, 282 MHz)

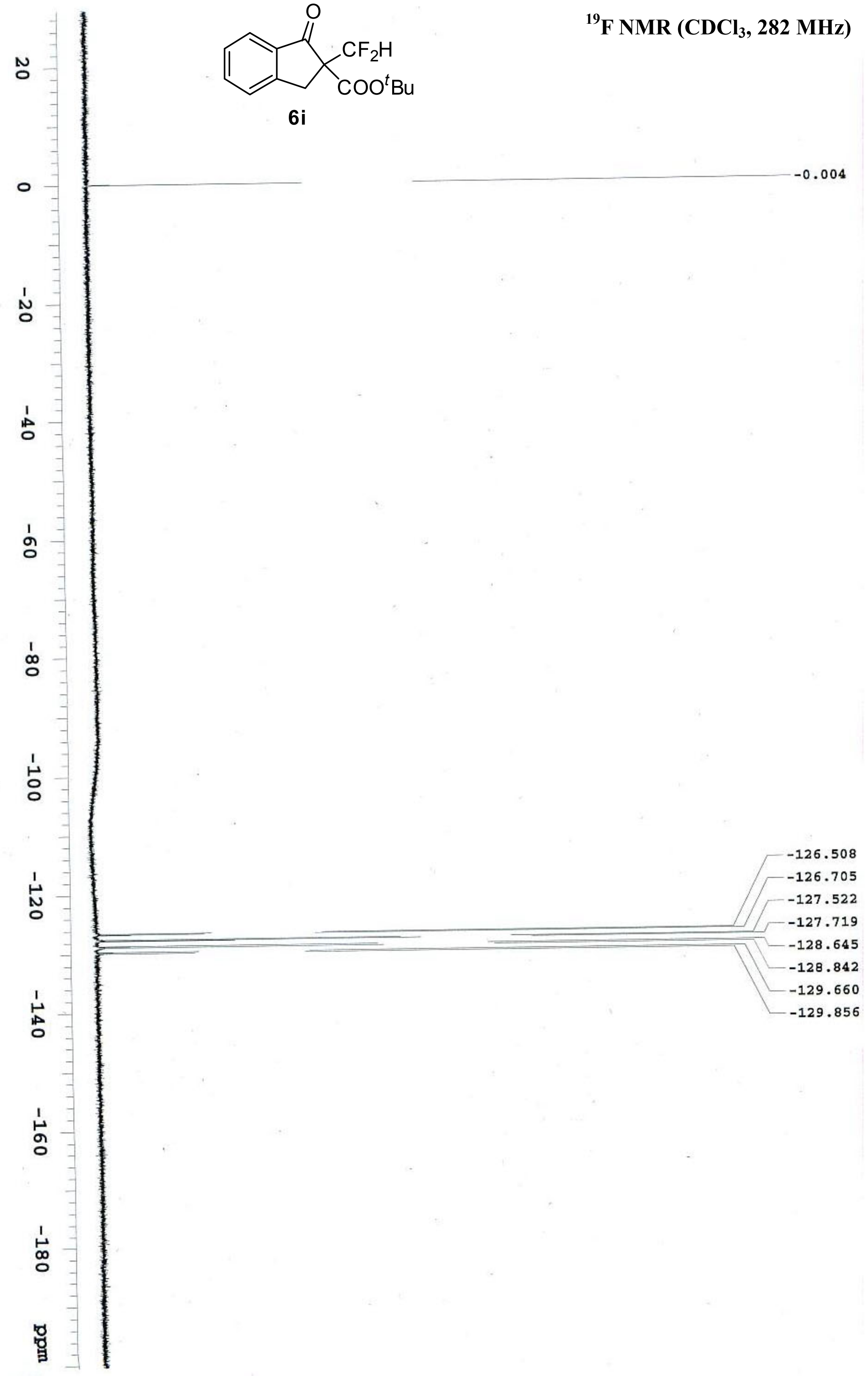

^{13}C NMR ($CDCl_3$, 150.9 MHz)

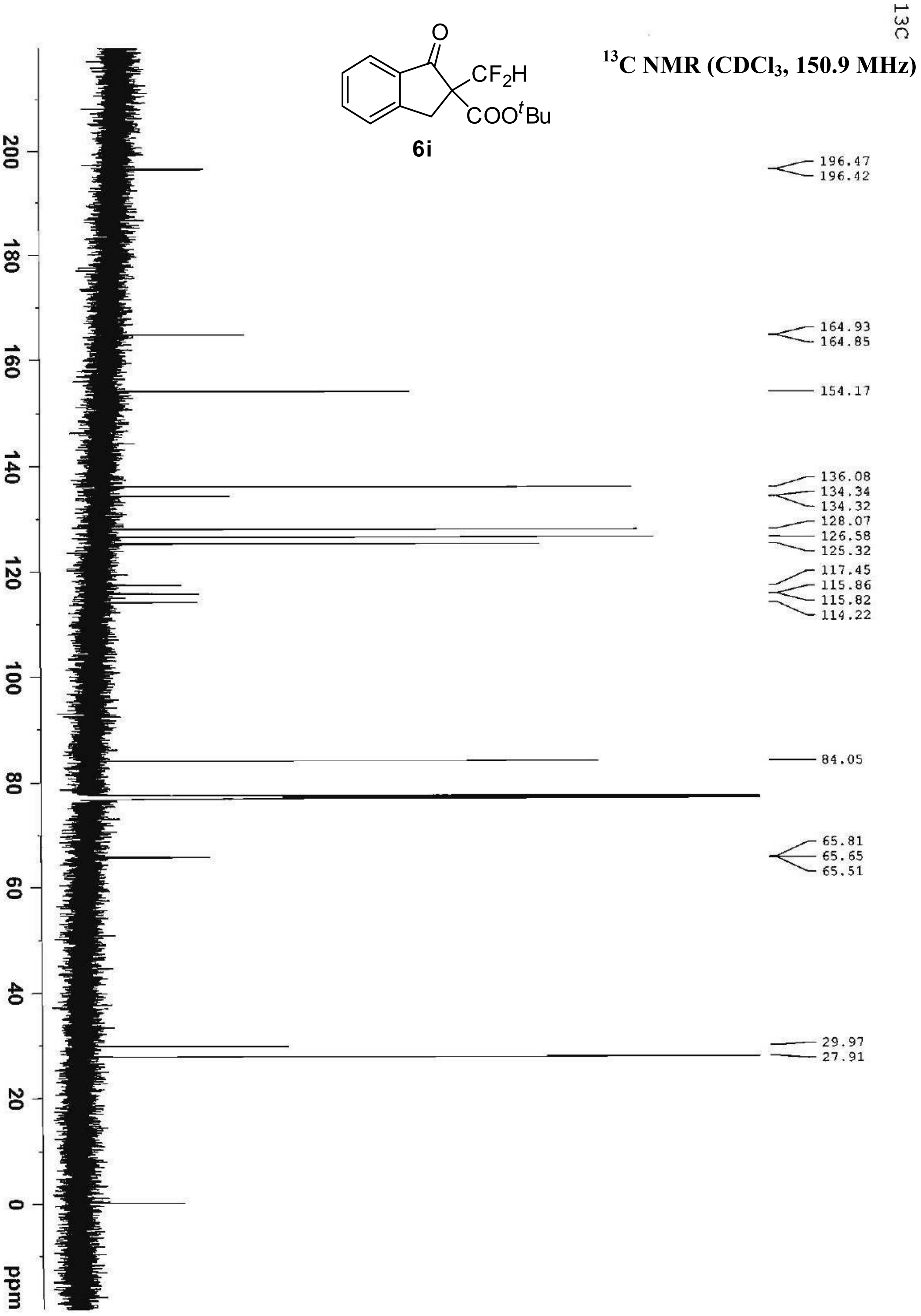

^{1}H NMR ($CDCl_3$, 300 MHz)

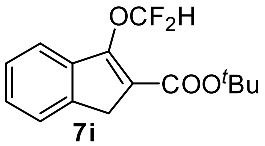

7i

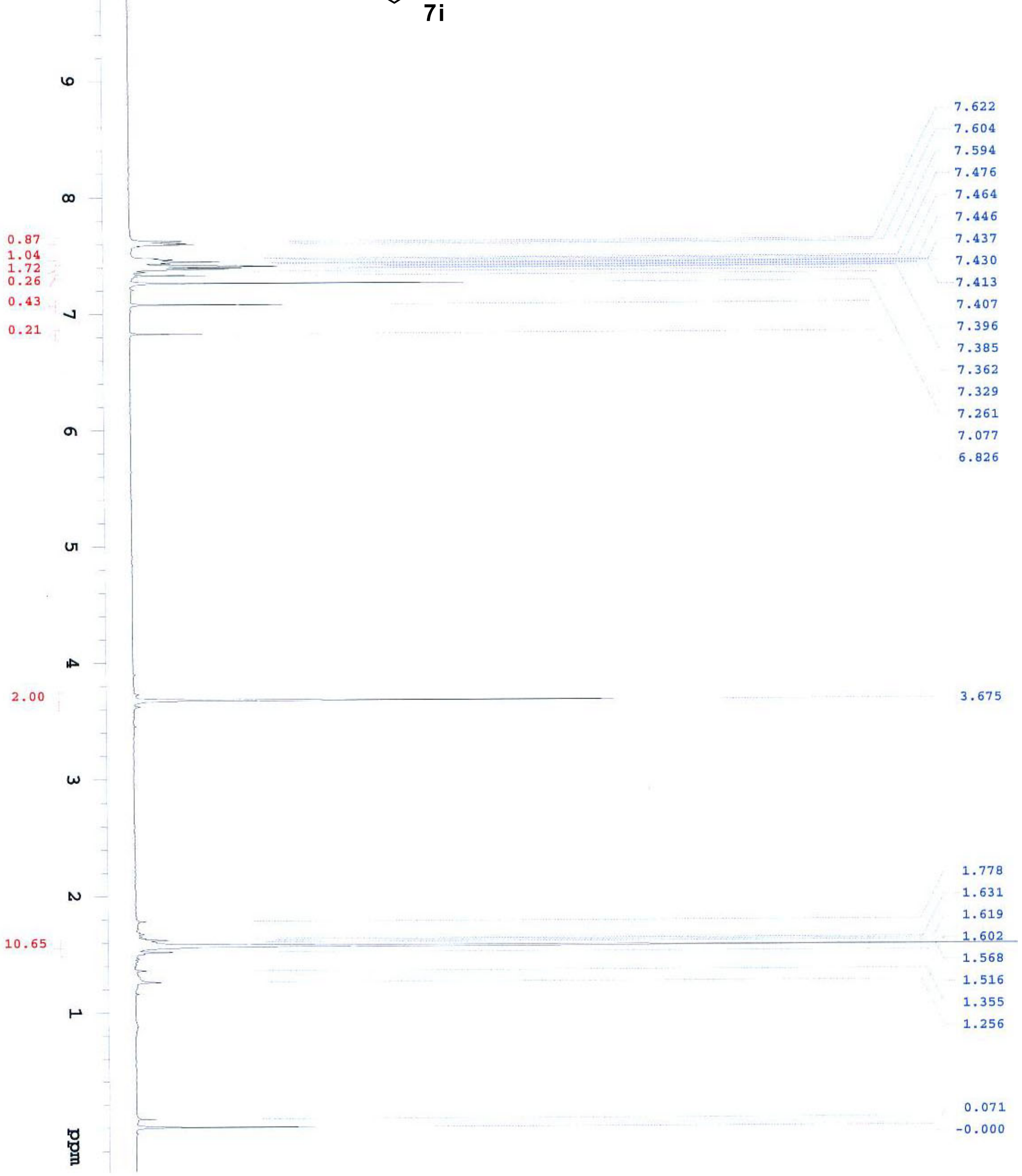

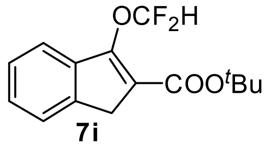

7i

^{19}F NMR ($CDCl_3$, 282 MHz)

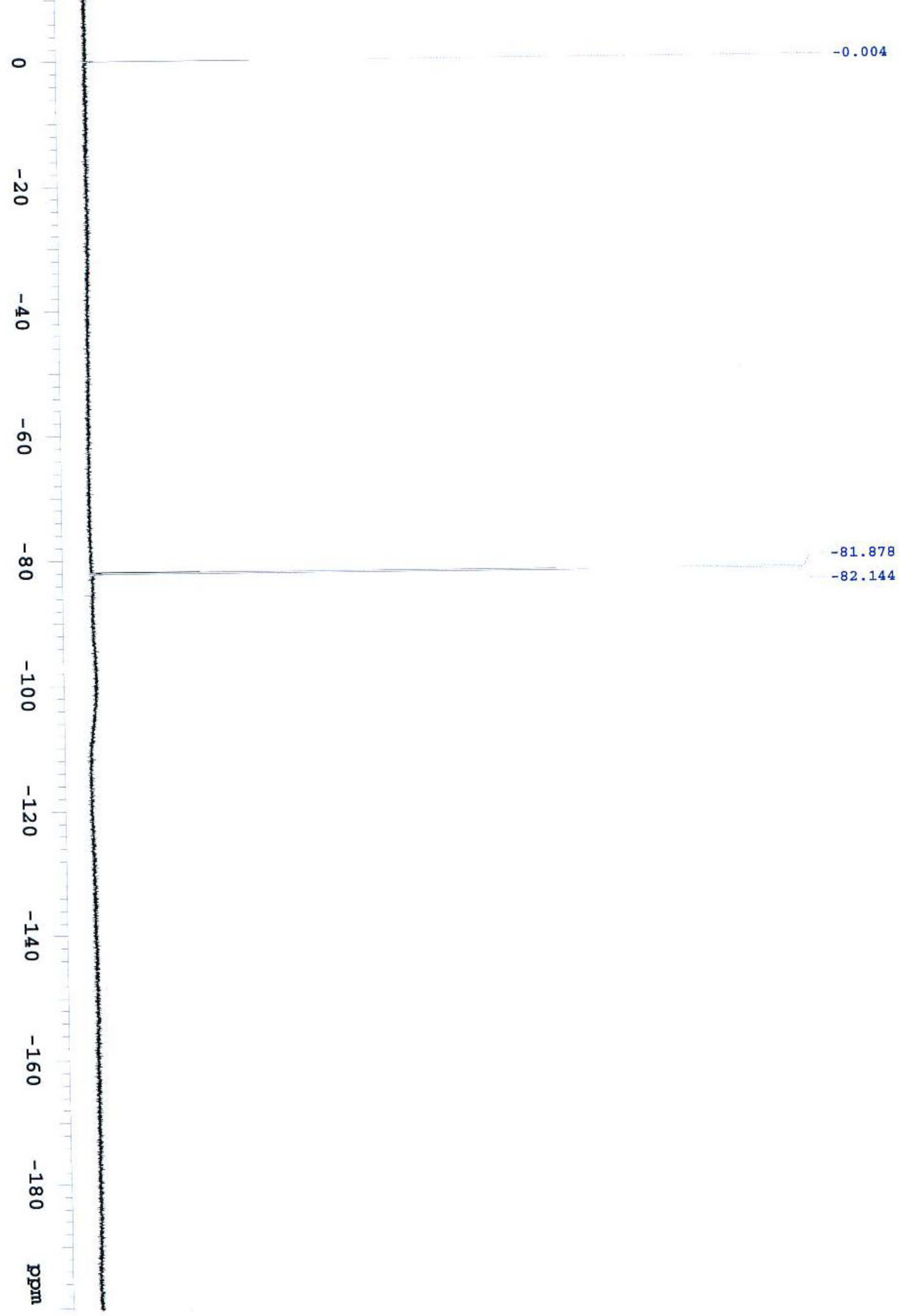

^{13}C NMR ($CDCl_3$, 150.9 MHz)

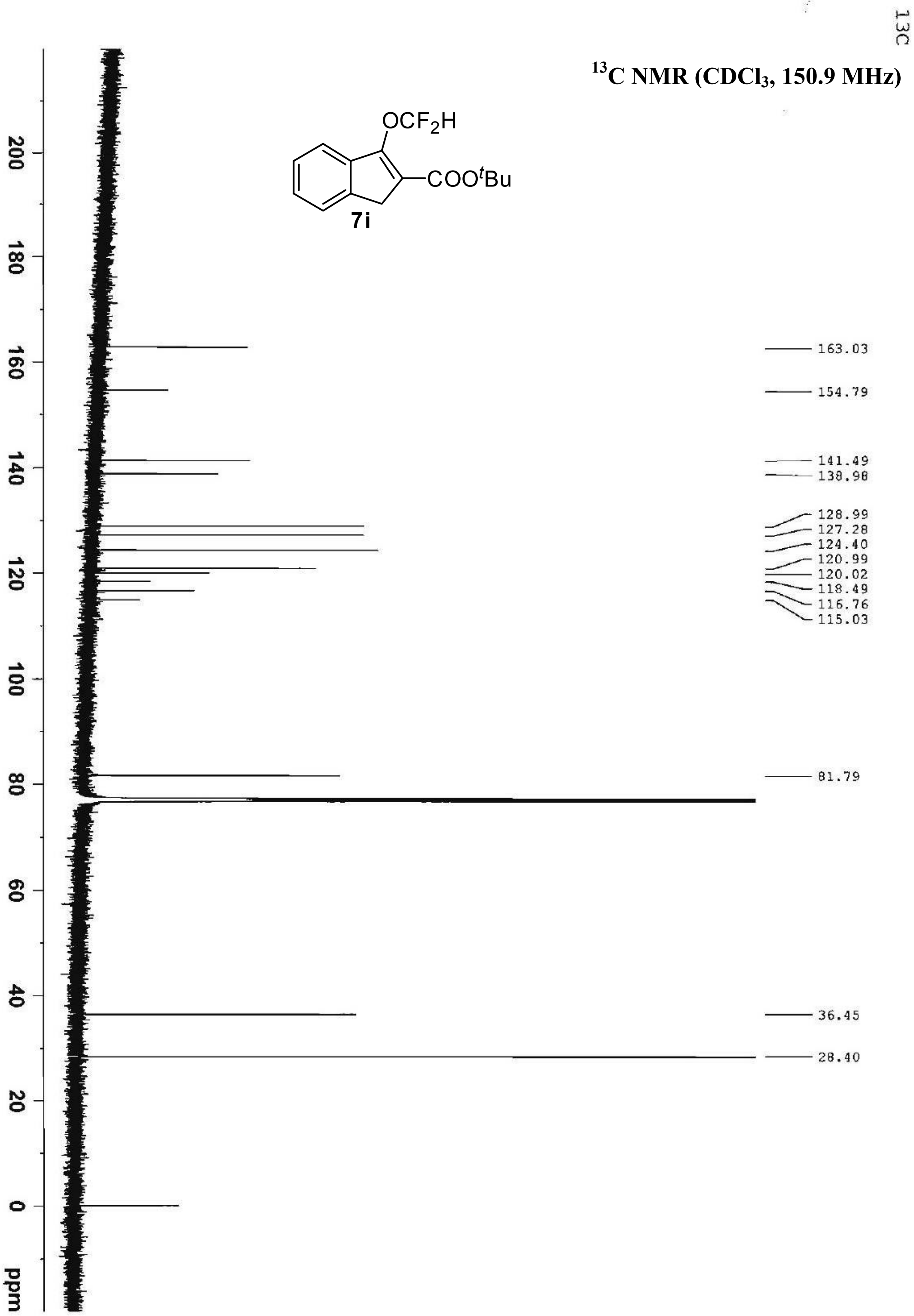

^{1}H NMR ($CDCl_3$, 300 MHz)

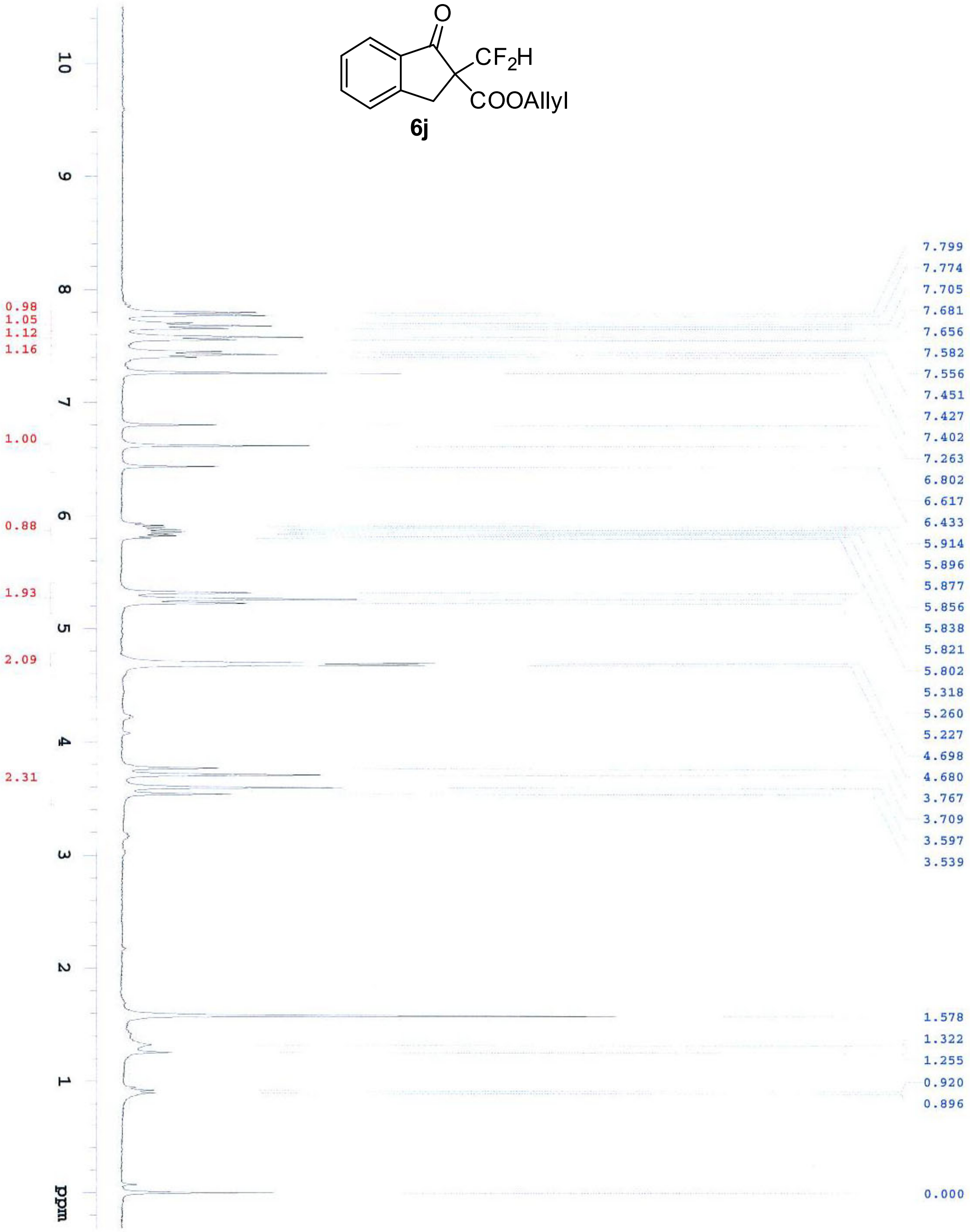

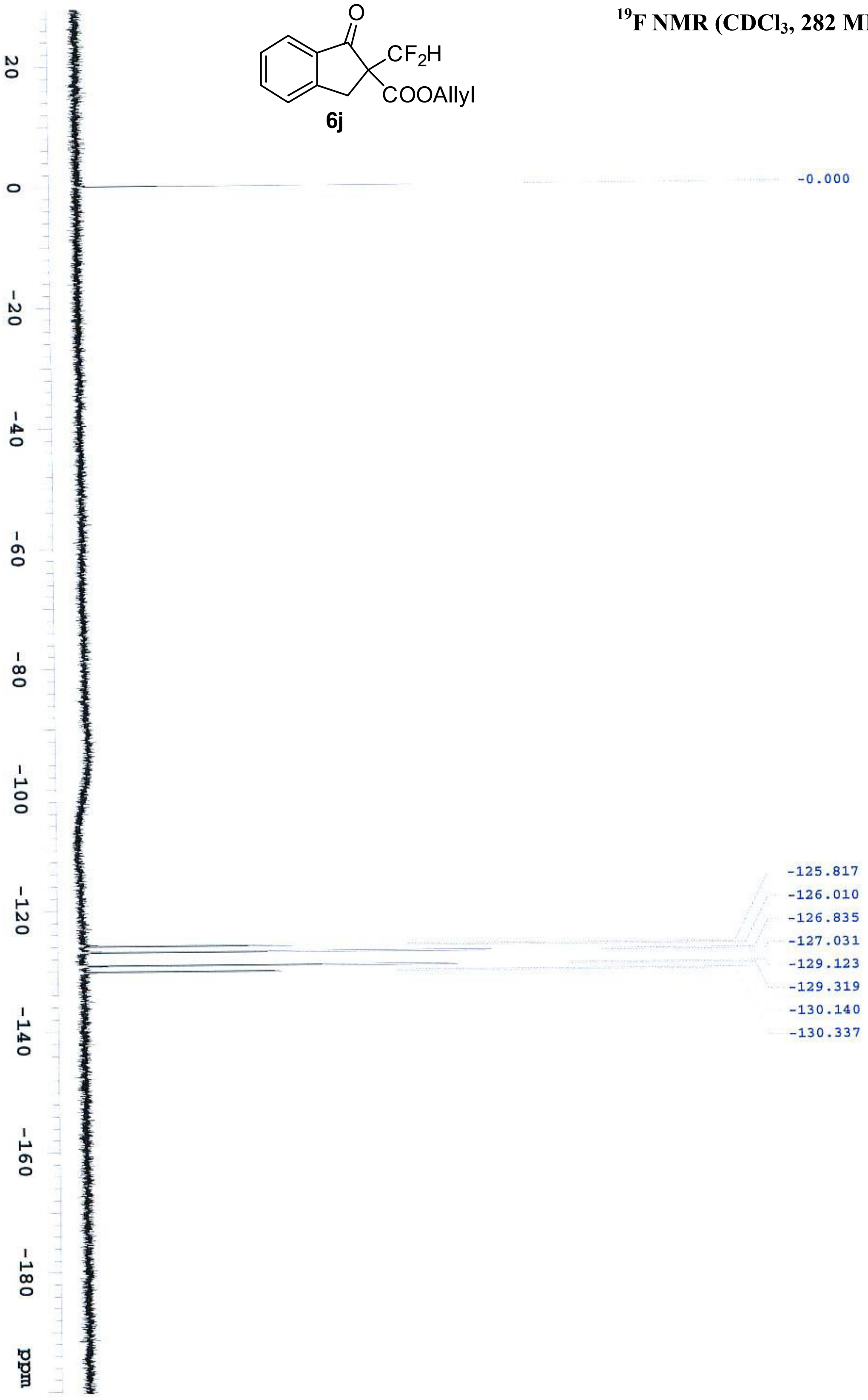

6j

^{19}F NMR ($CDCl_3$, 282 MHz)

^{13}C NMR ($CDCl_3$, 150.9 MHz)

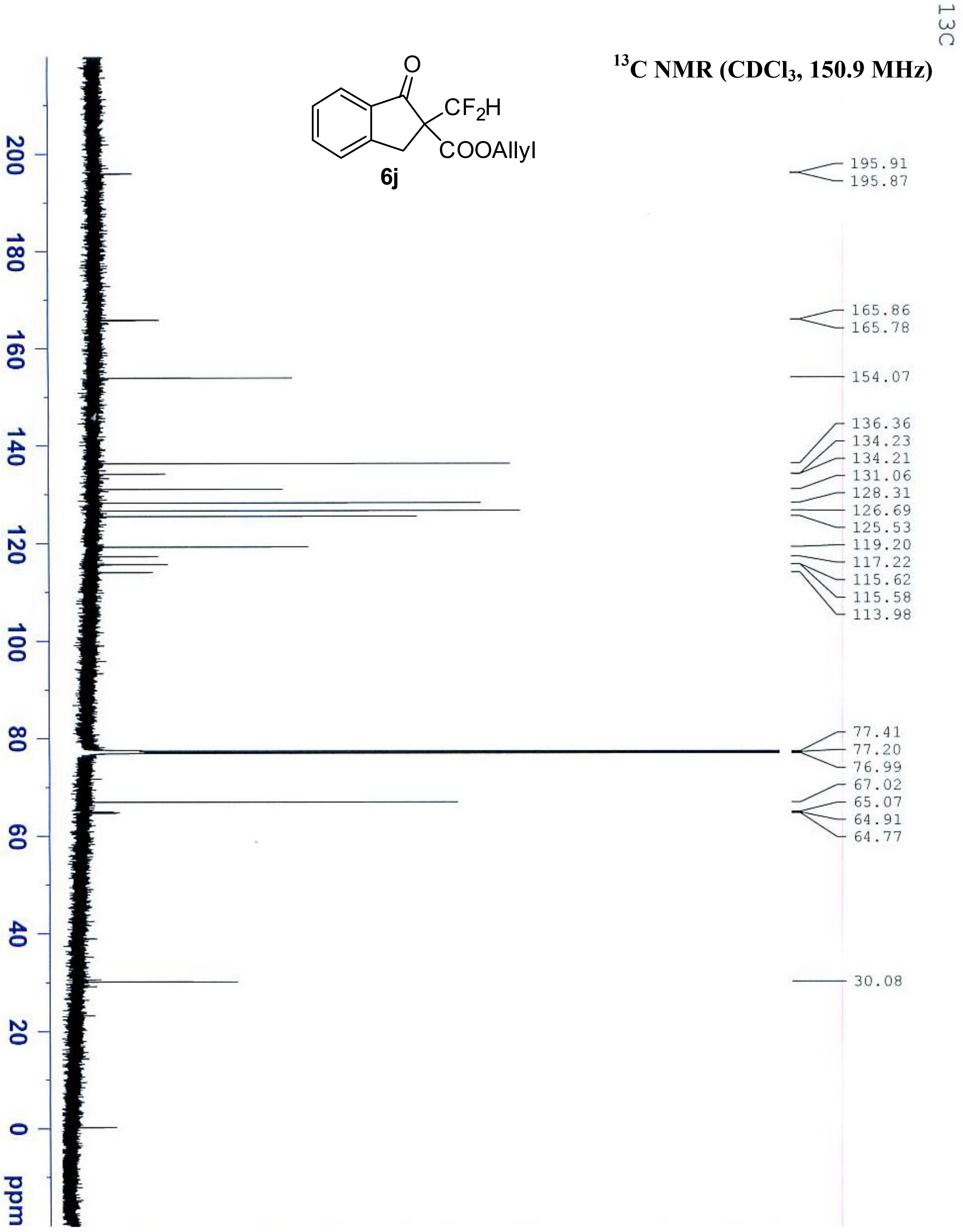

6j

^{1}H NMR ($CDCl_3$, 300 MHz)

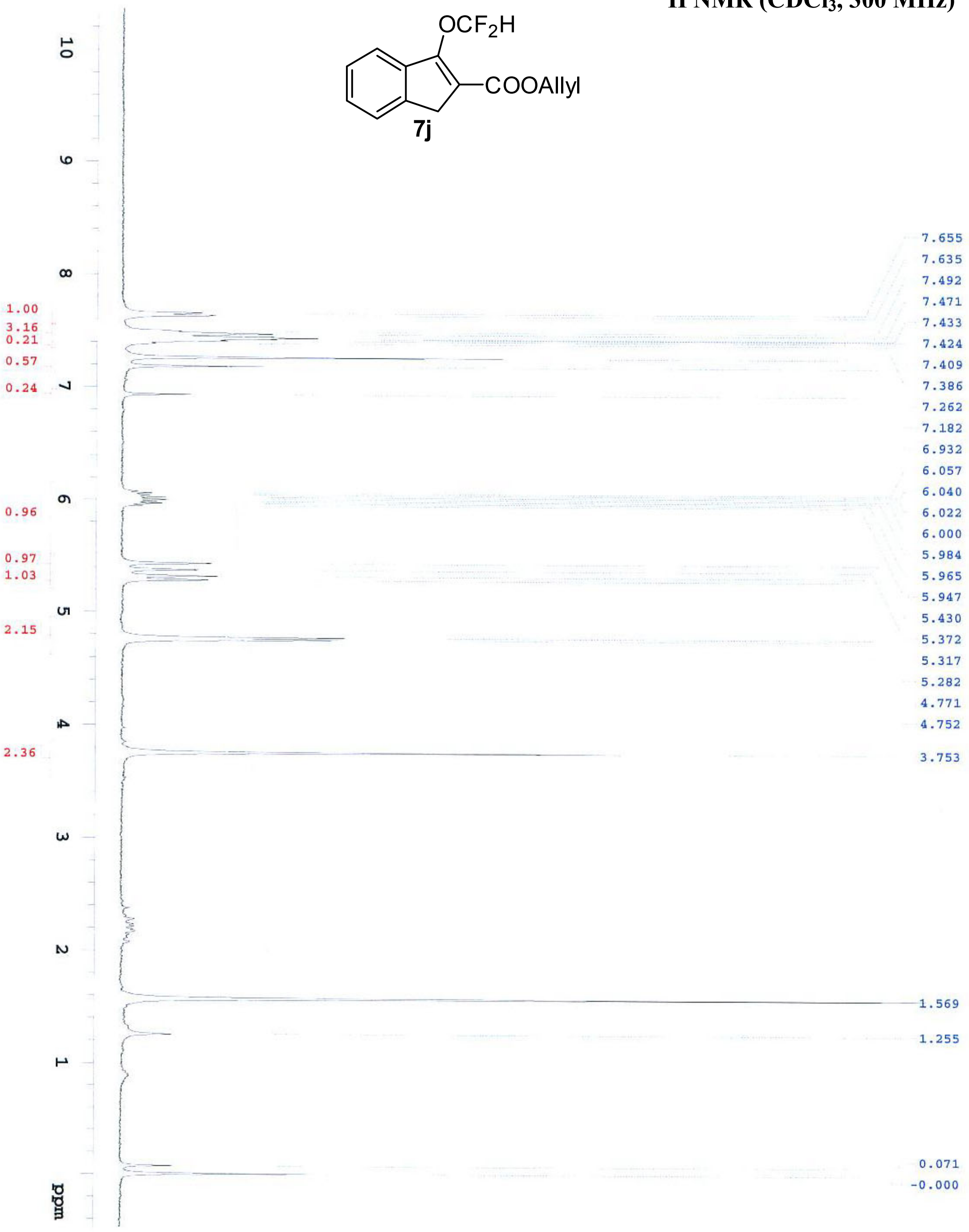

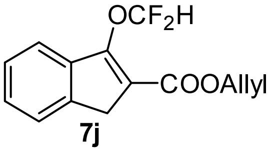

7j

^{19}F NMR (CDCl$_3$, 282 MHz)

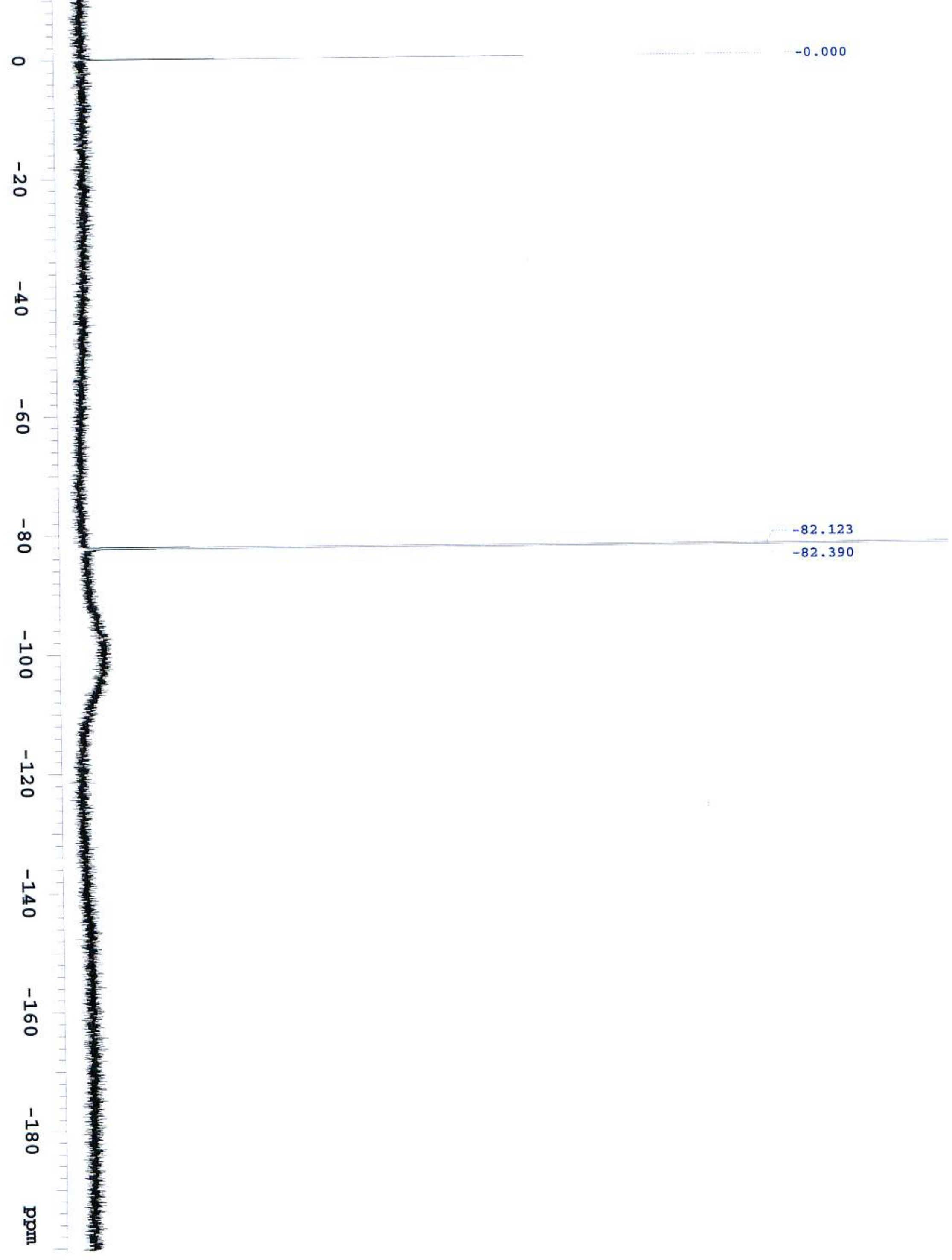

^{13}C NMR ($CDCl_3$, 150.9 MHz)

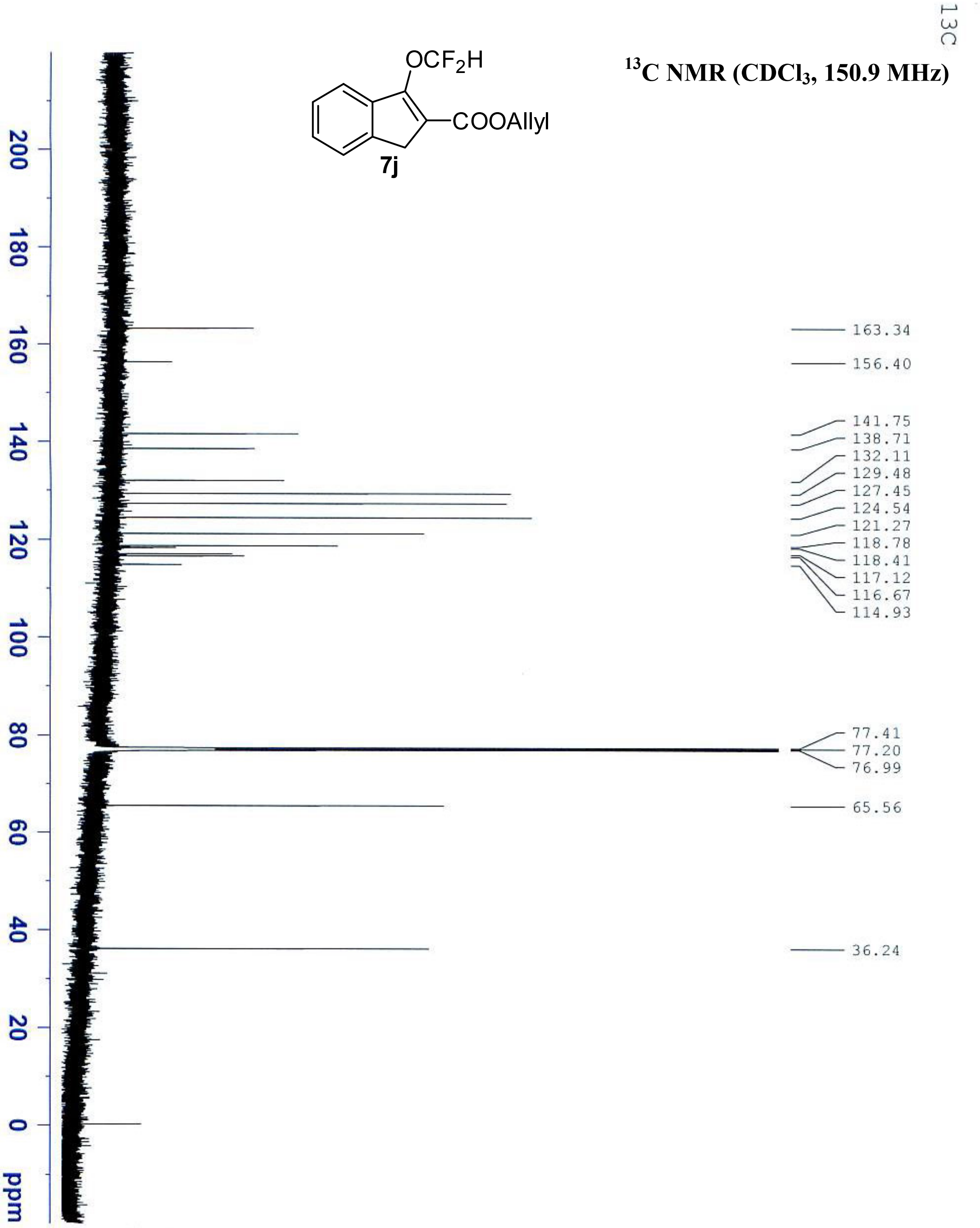

^{1}H NMR ($CDCl_3$, 300 MHz)

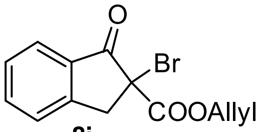

8j

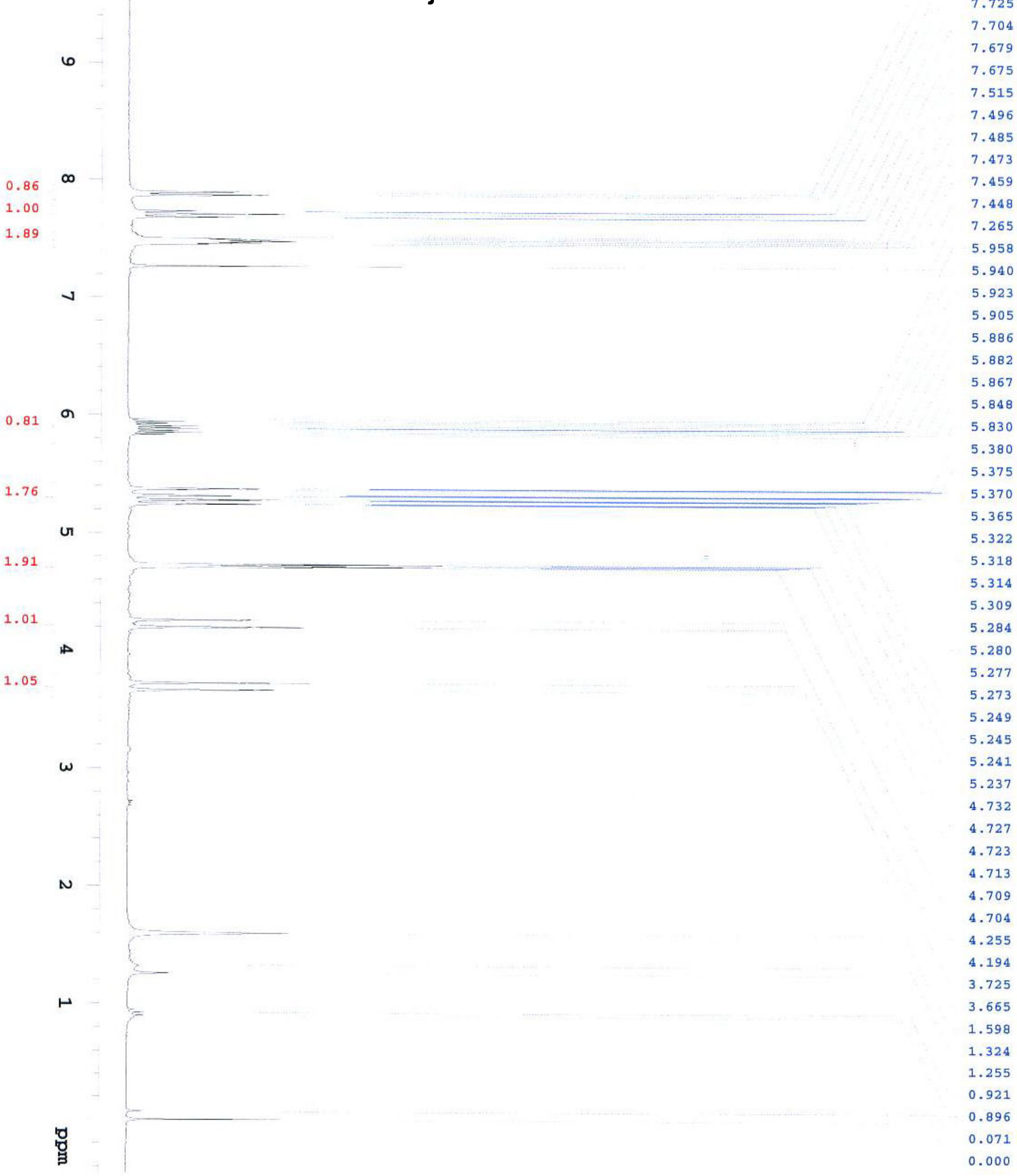

^{13}C NMR ($CDCl_3$, 150.9 MHz)

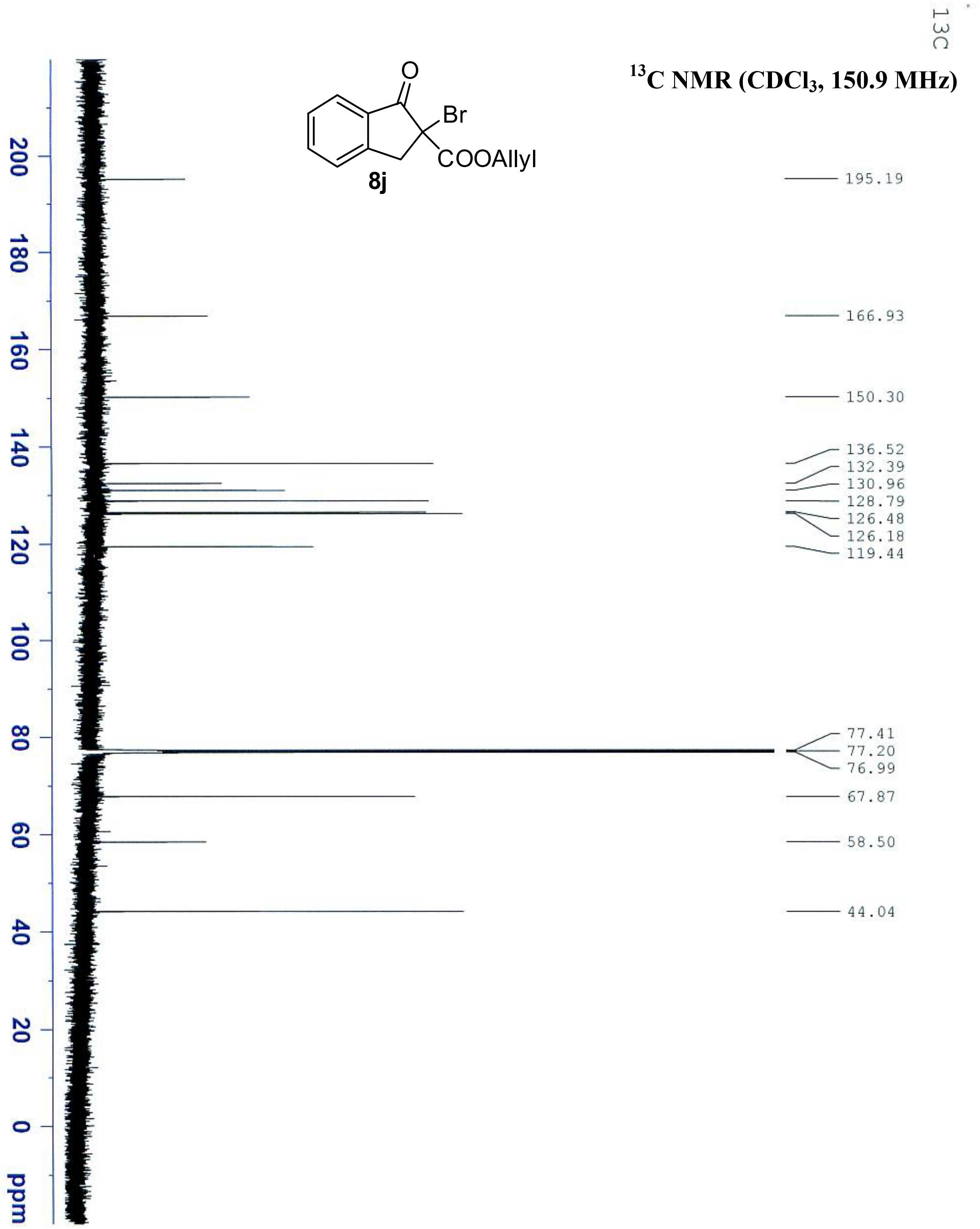

^{1}H NMR ($CDCl_3$, 300 MHz)

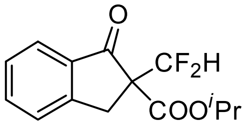

6k

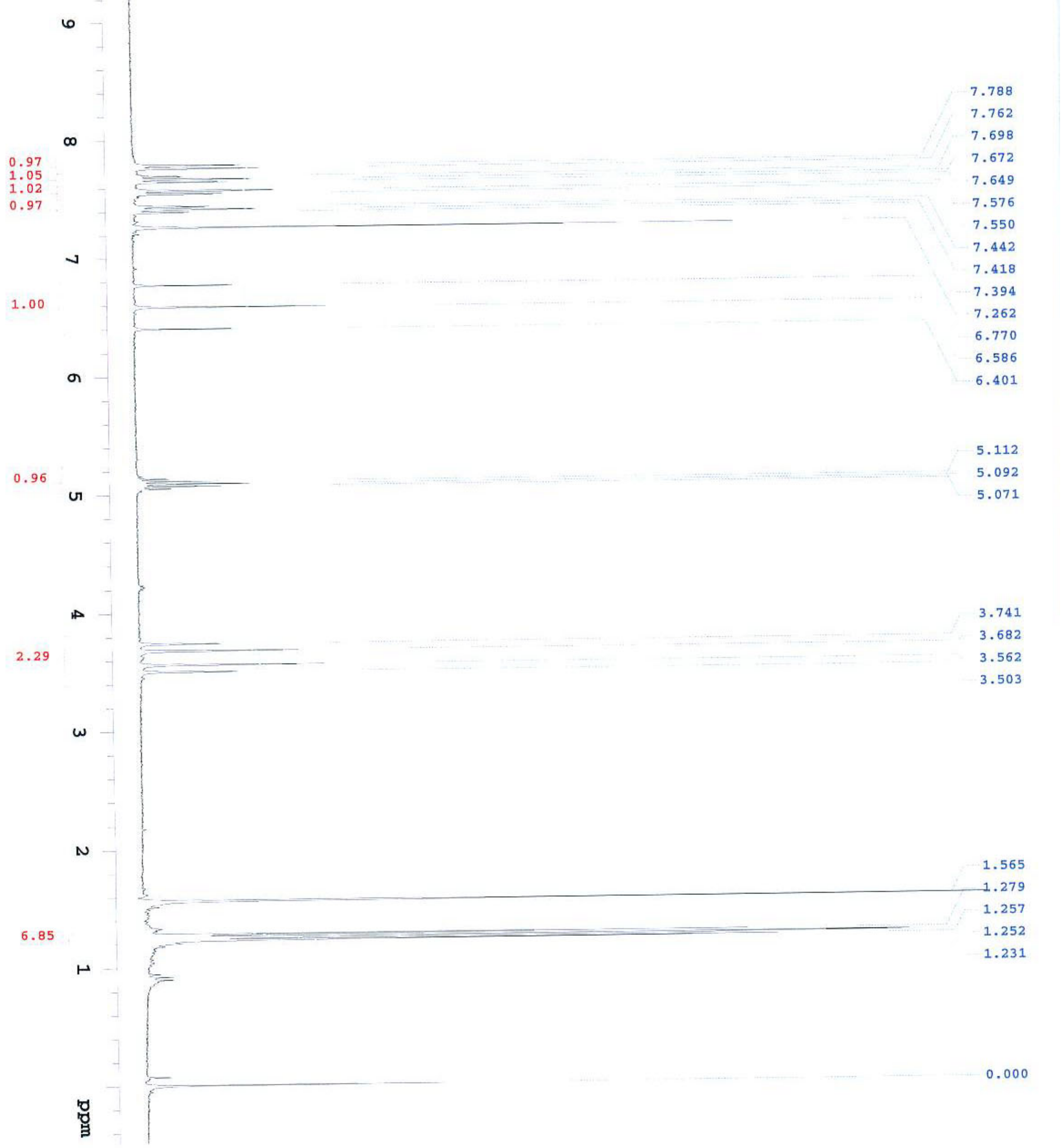

^{19}F NMR ($CDCl_3$, 282 MHz)

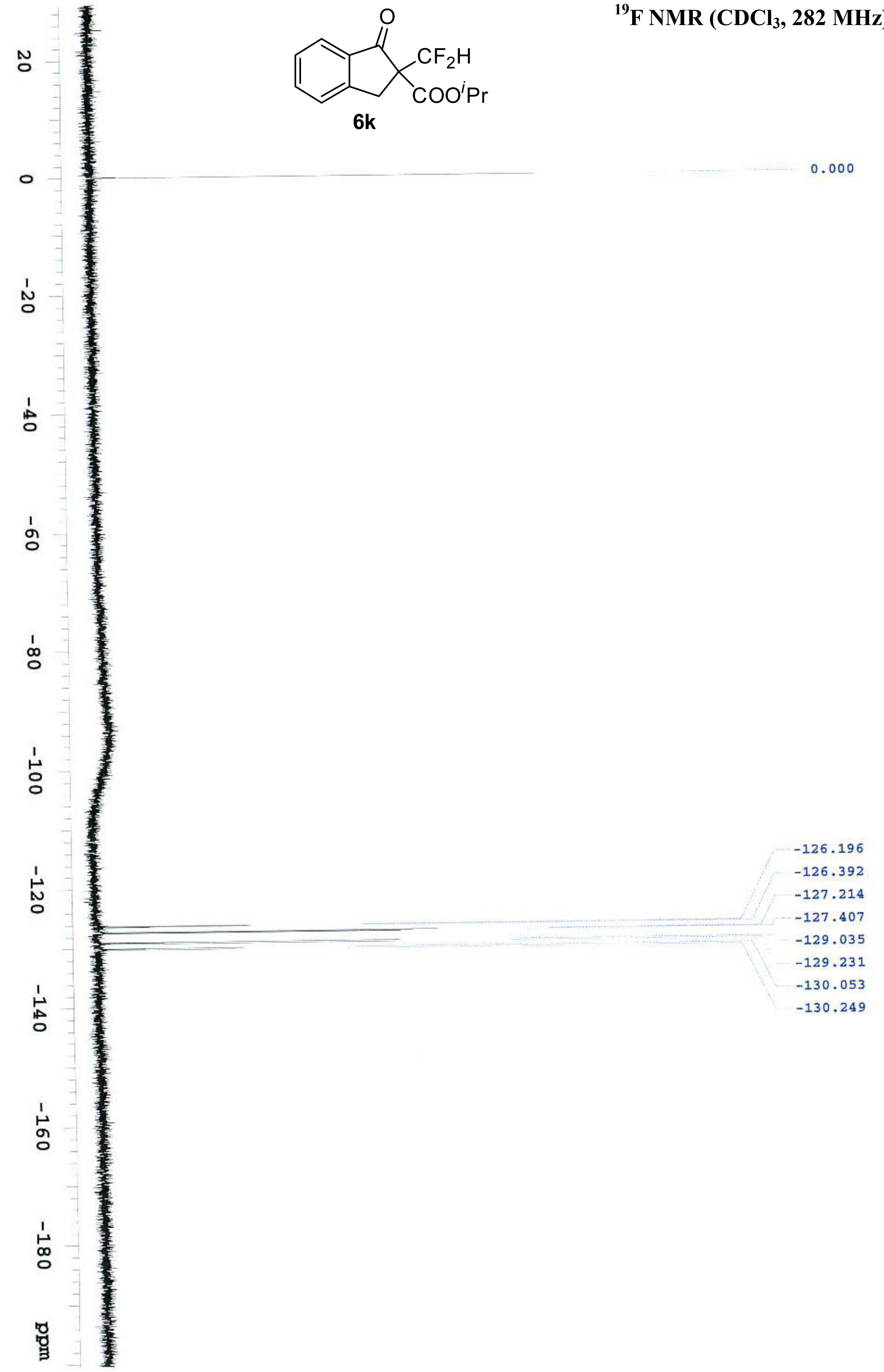

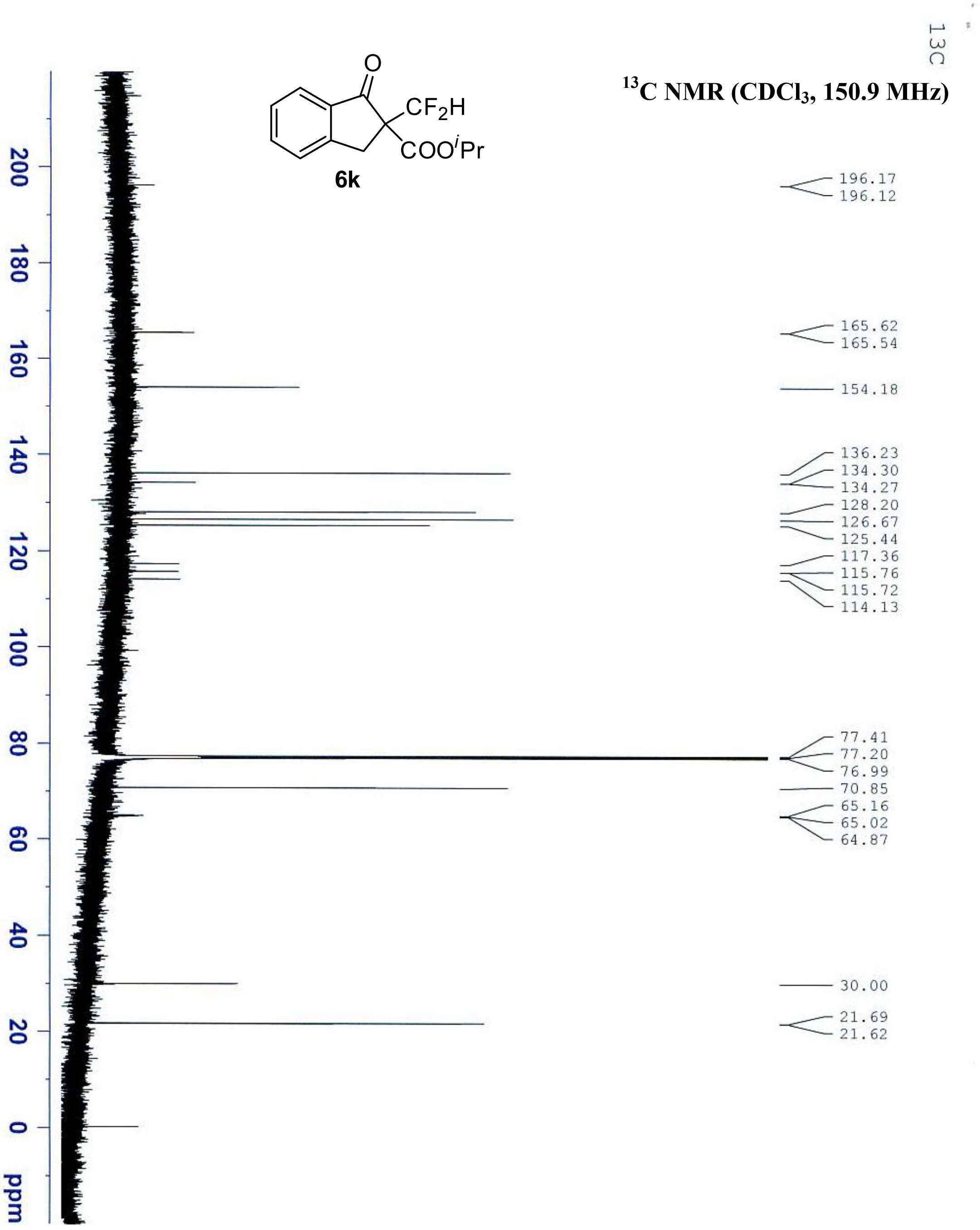
13C
^{13}C NMR ($CDCl_3$, 150.9 MHz)
6k
O
CF_2H
COO^iPr
196.17
196.12
165.62
165.54
154.18
136.23
134.30
134.27
128.20
126.67
125.44
117.36
115.76
115.72
114.13
77.41
77.20
76.99
70.85
65.16
65.02
64.87
30.00
21.69
21.62
200
180
160
140
120
100
80
60
40
20
0
ppm

^{1}H NMR ($CDCl_3$, 300 MHz)

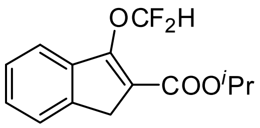

7k

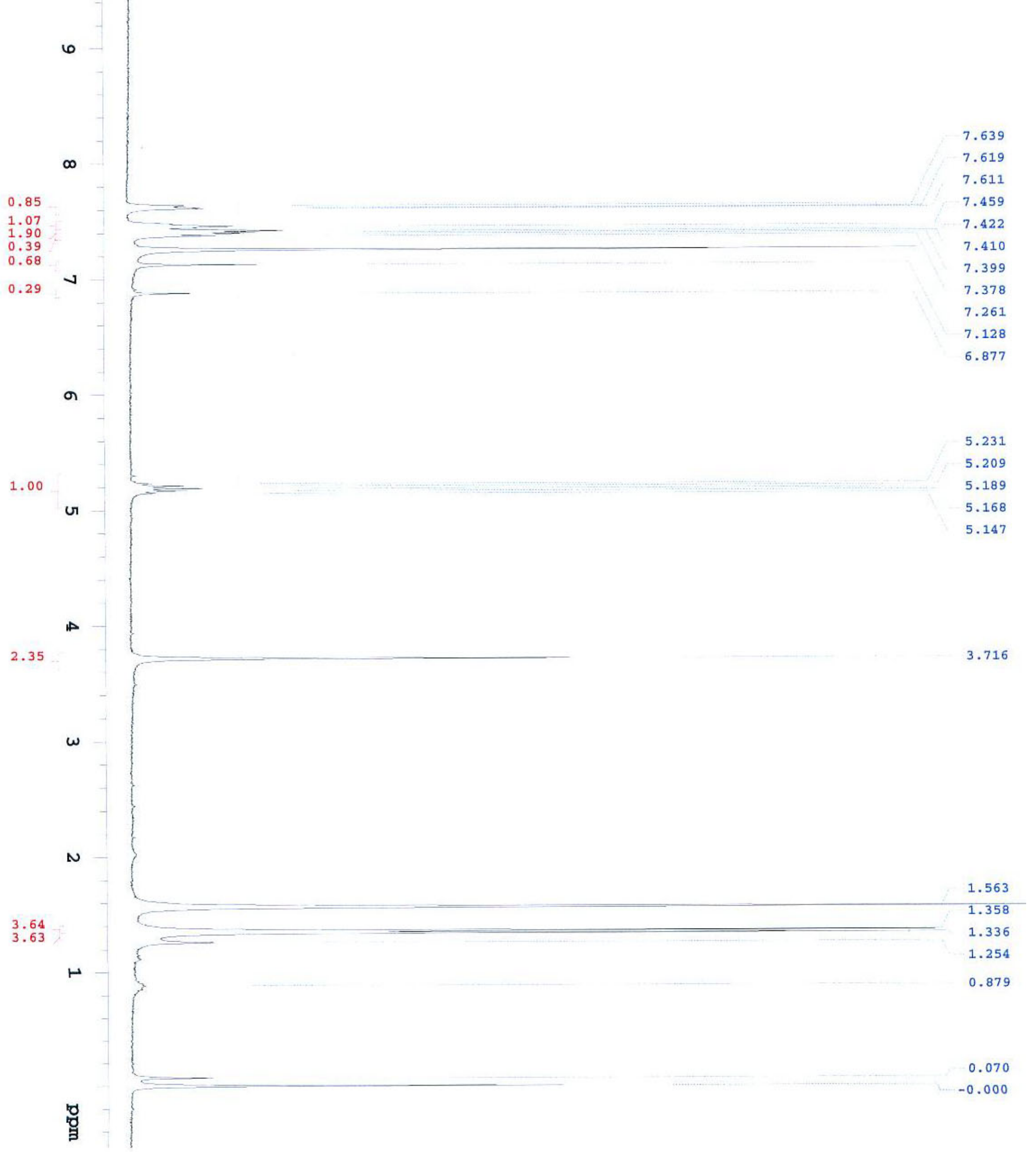

^{19}F NMR ($CDCl_3$, 282 MHz)

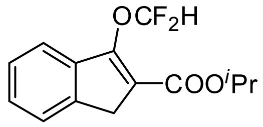

7k

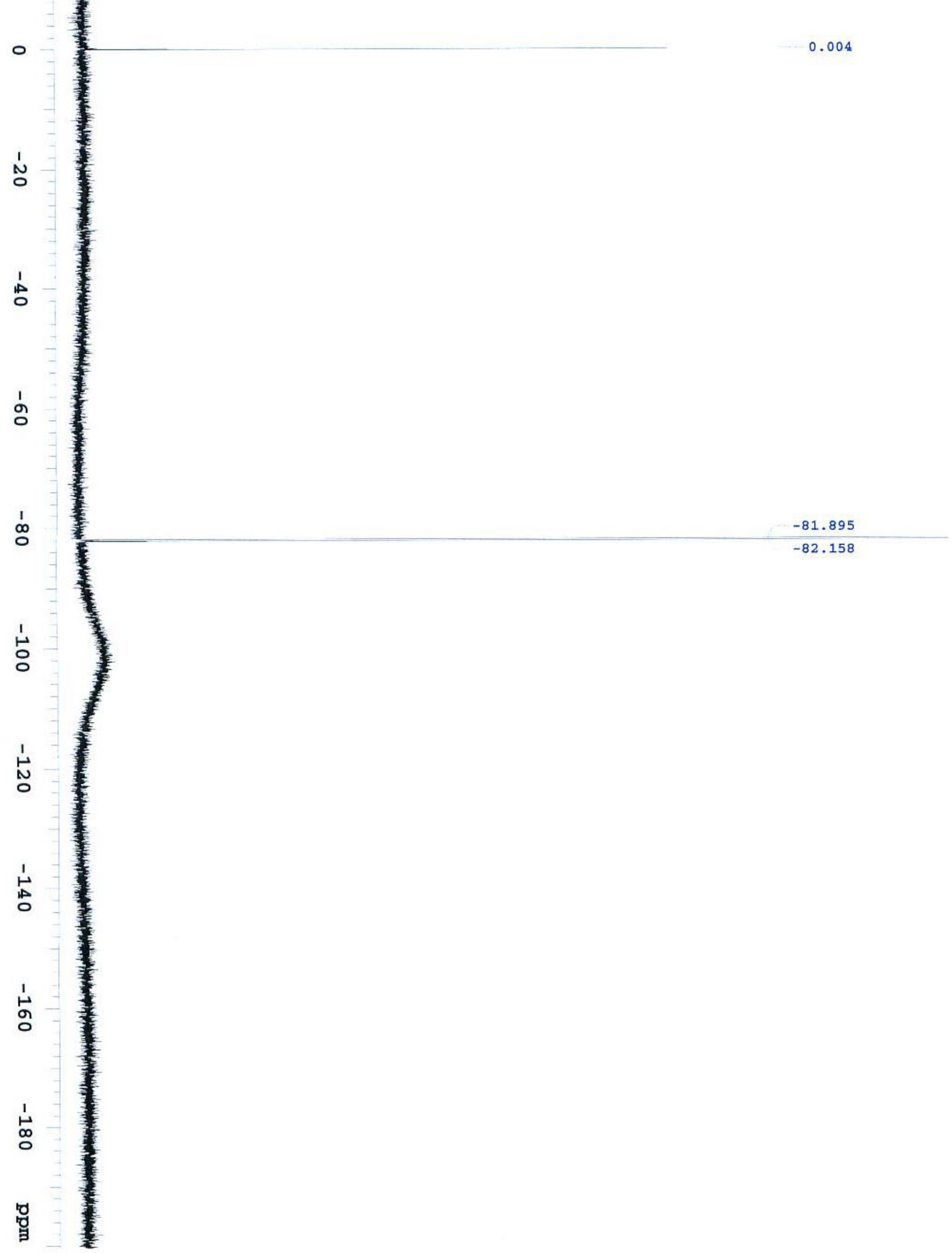

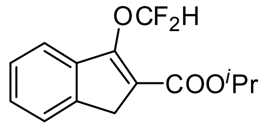

7k

^{13}C NMR (CDCl$_3$, 150.9 MHz)

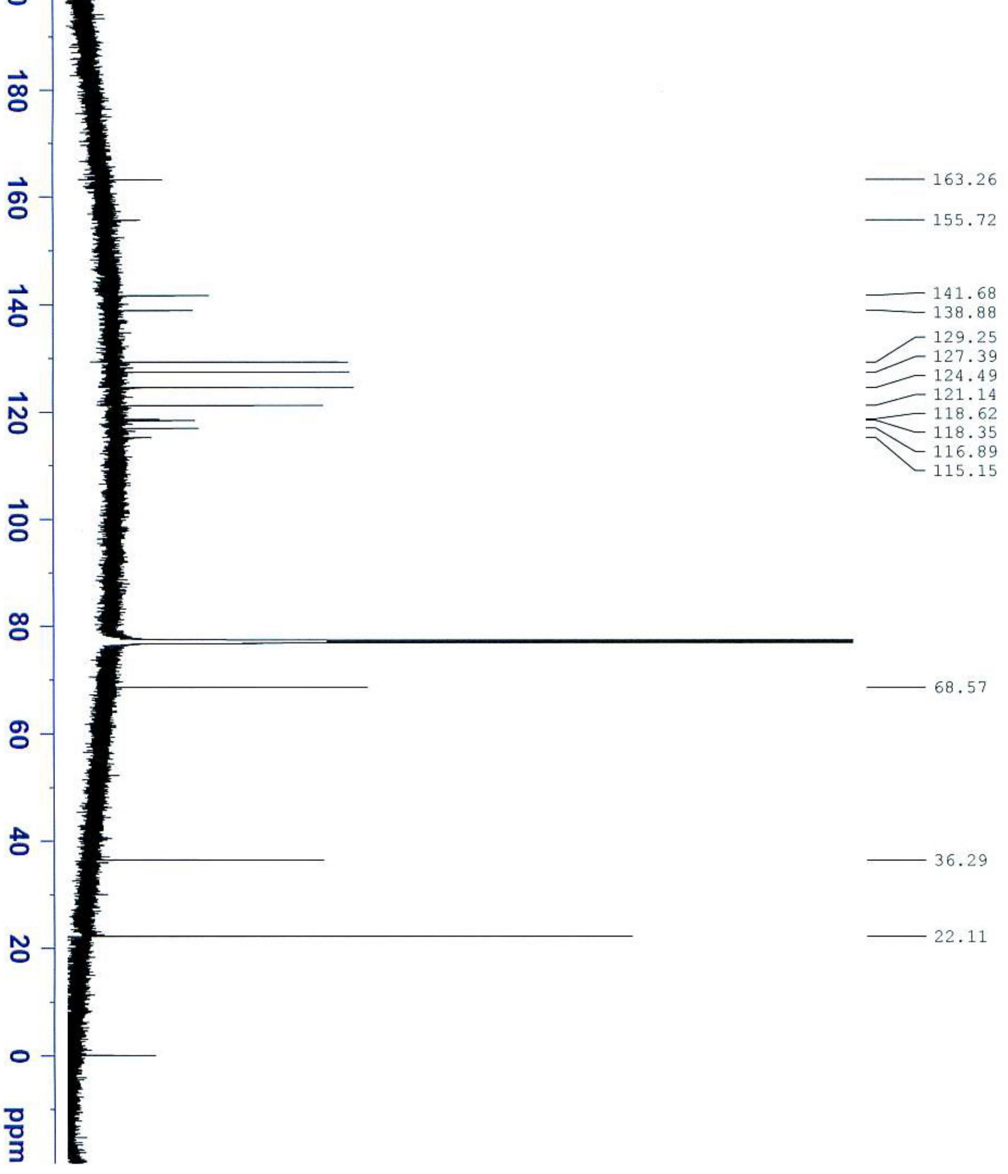

^{1}H NMR ($CDCl_3$, 300 MHz)

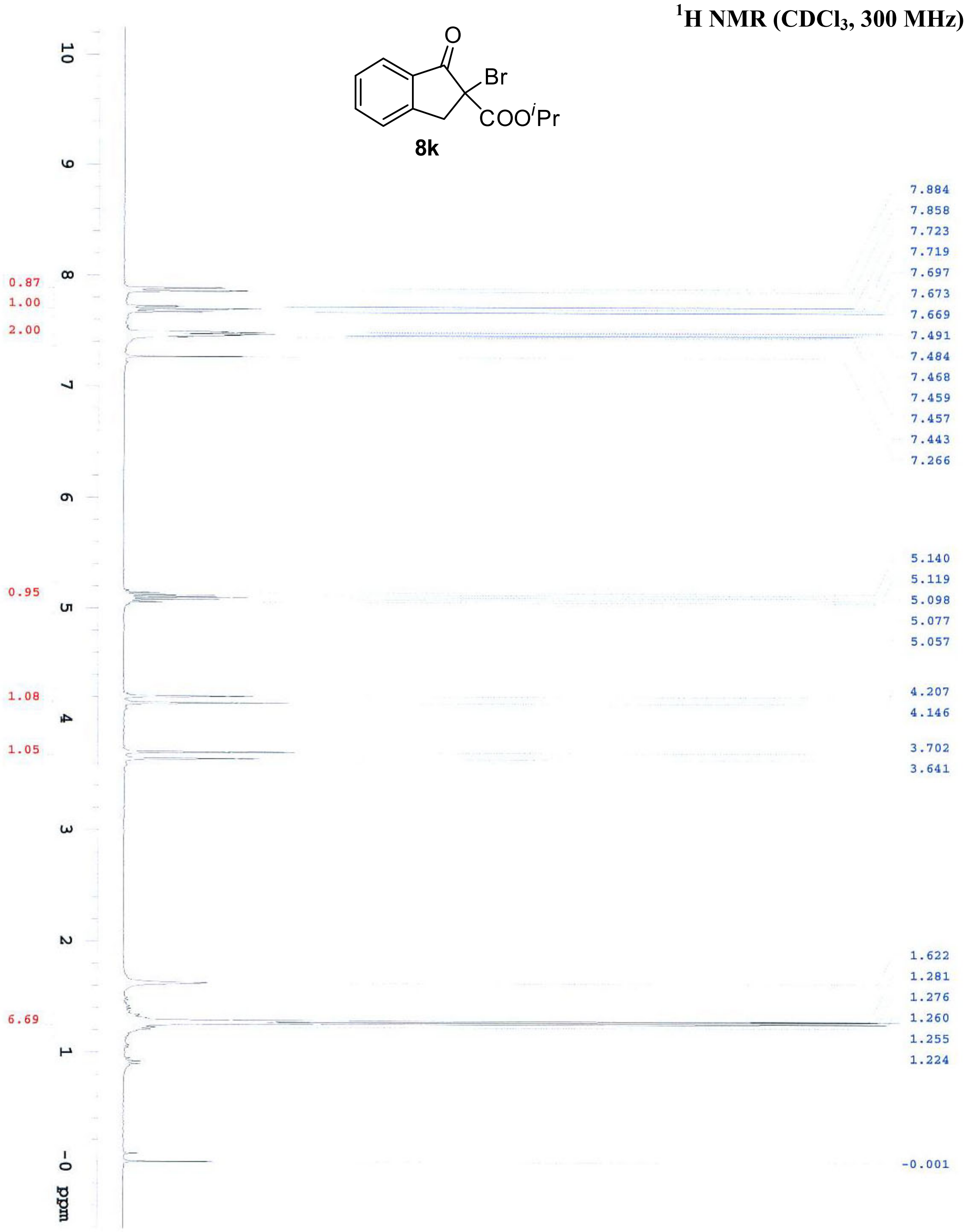

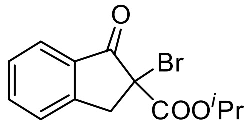

8k

^{13}C NMR ($CDCl_3$, 150.9 MHz)

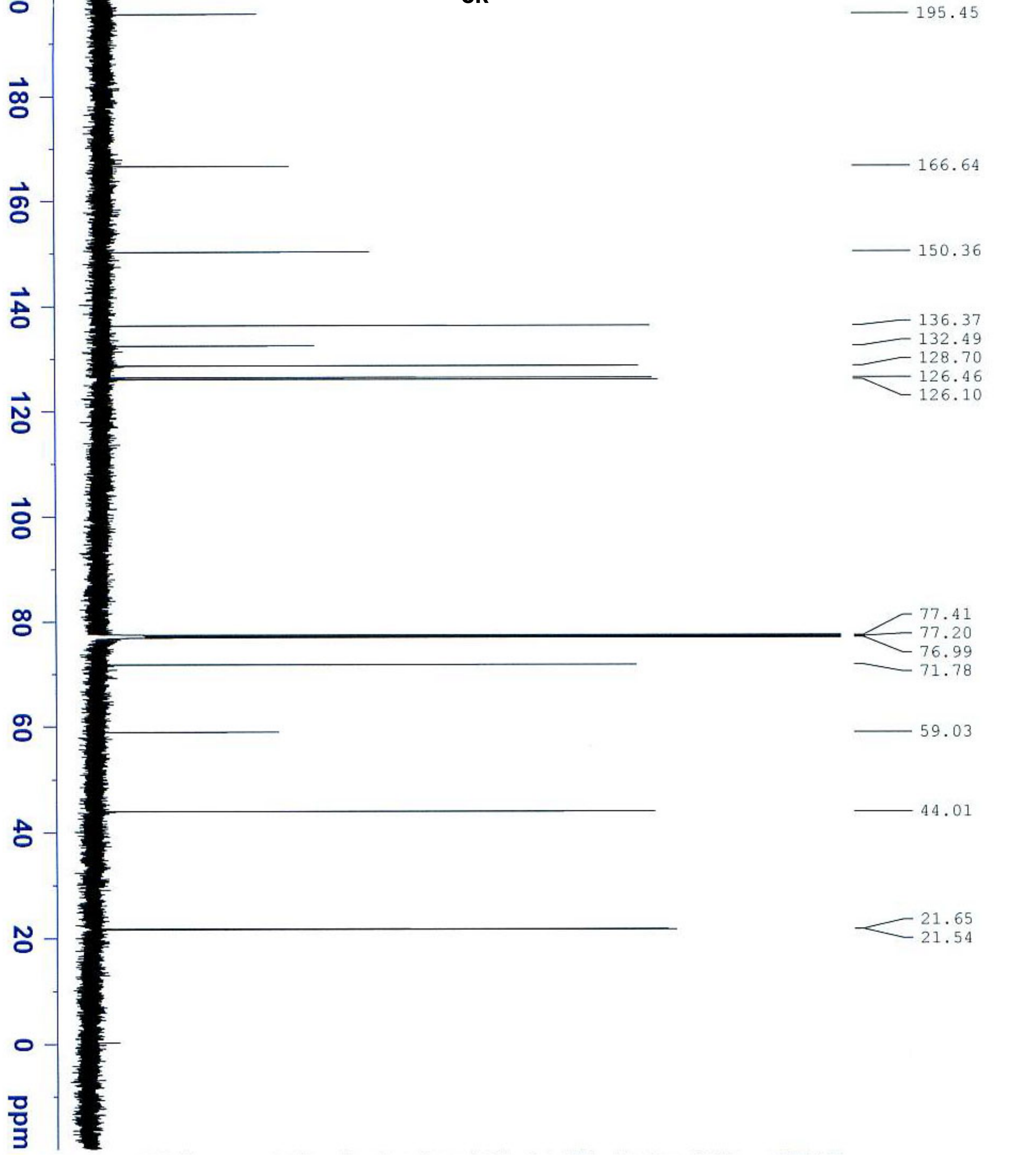

^{1}H NMR ($CDCl_3$, 300 MHz)

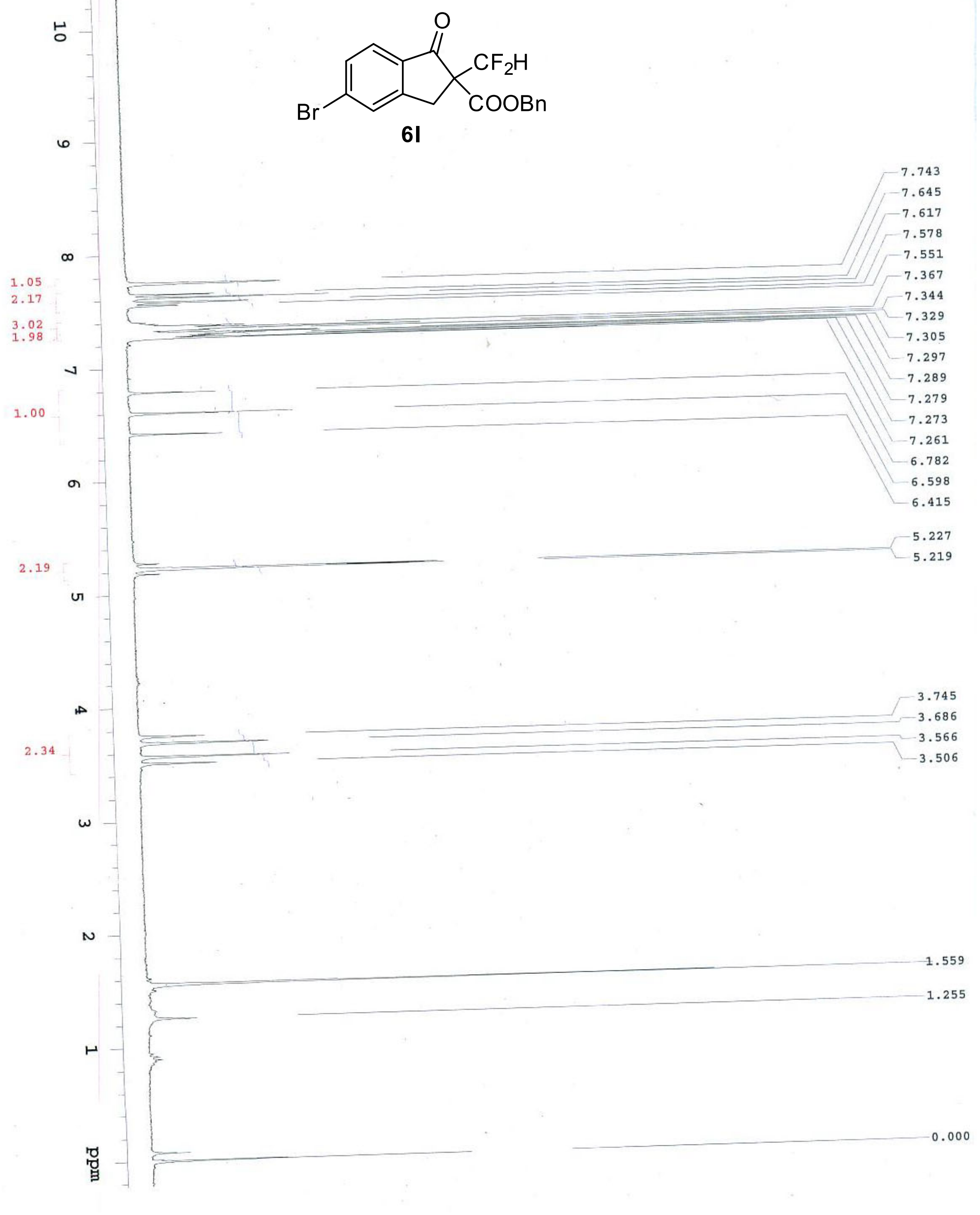

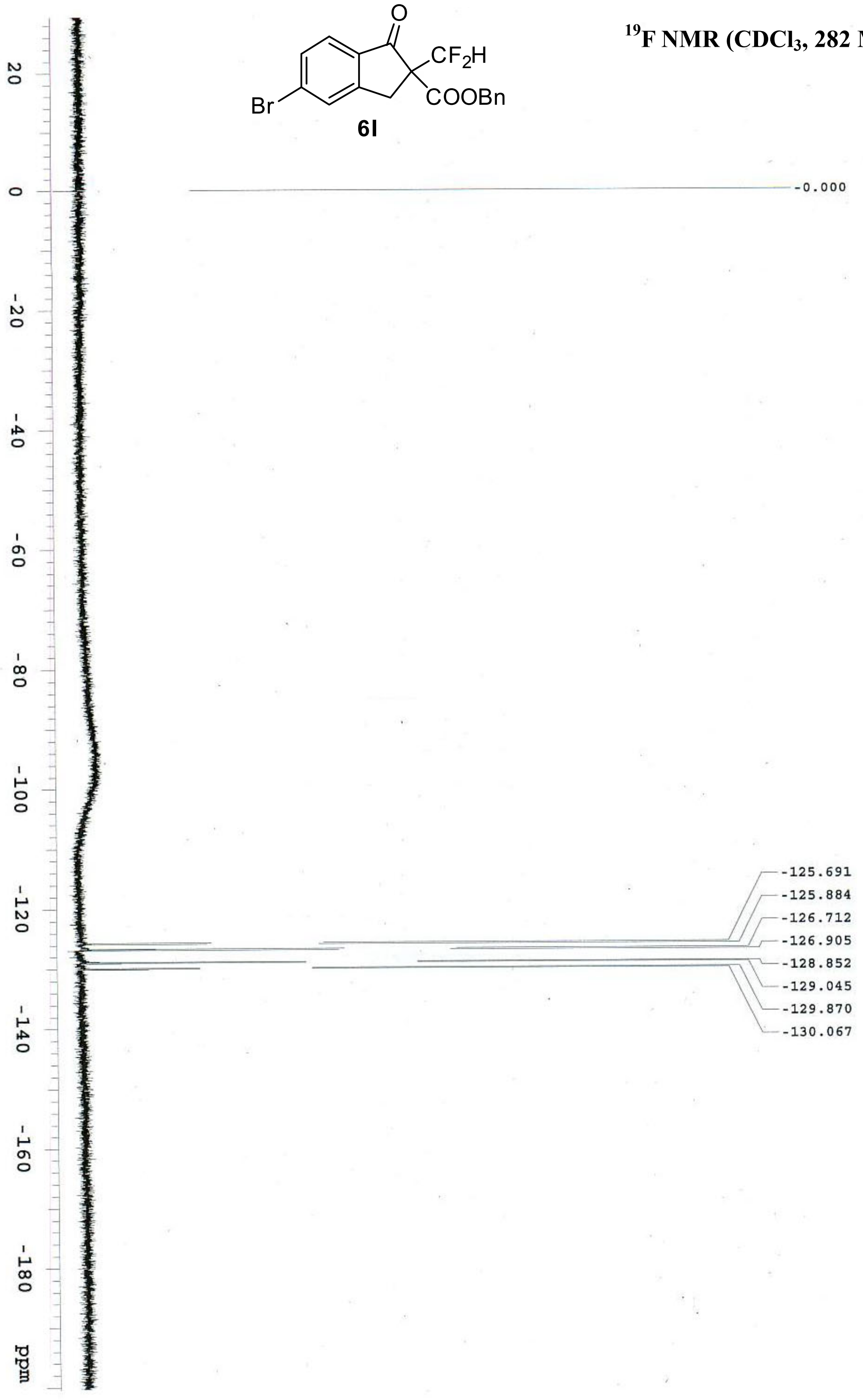

6l

^{19}F NMR (CDCl$_3$, 282 MHz)

^{13}C NMR ($CDCl_3$, 150.9 MHz)

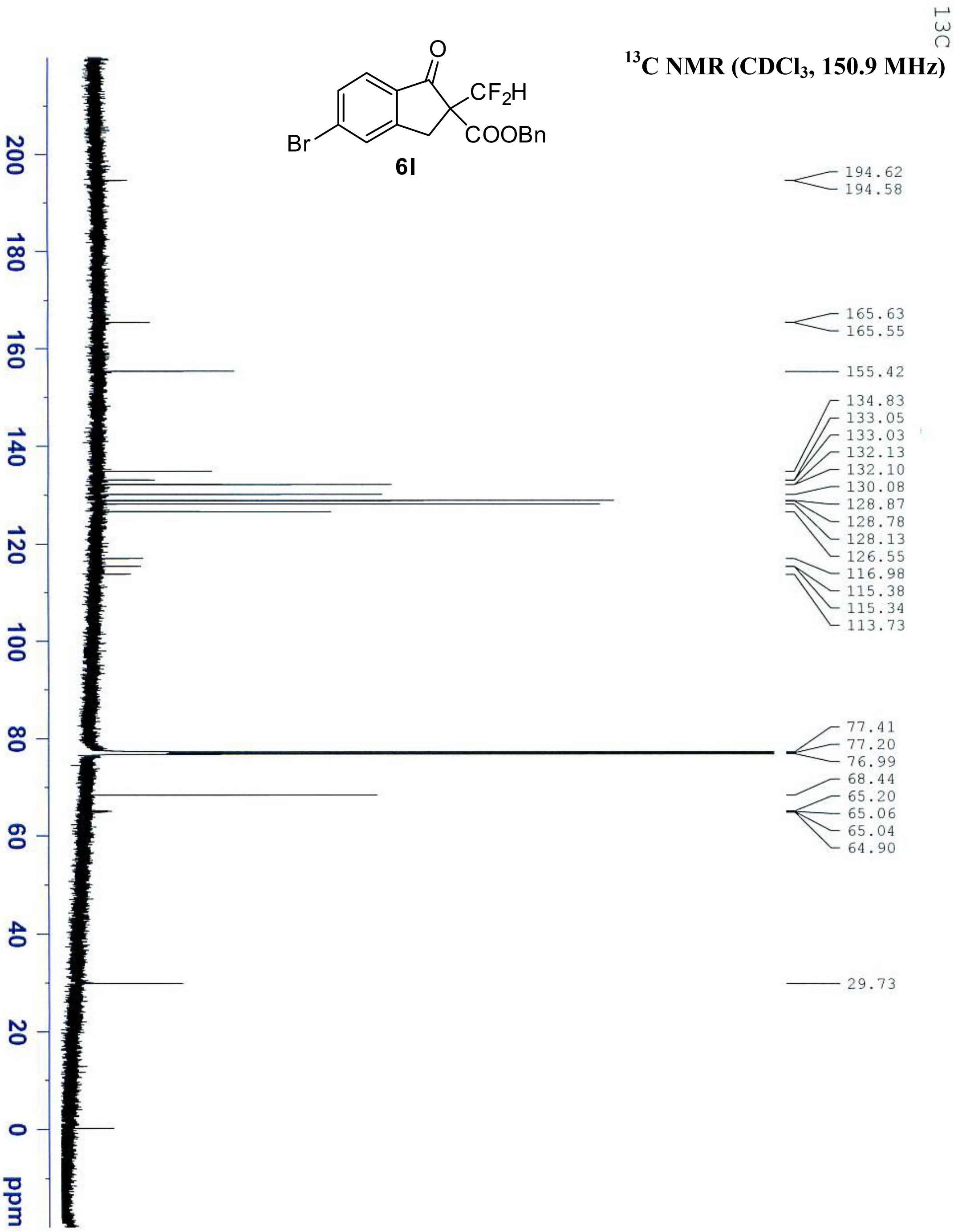

^{1}H NMR ($CDCl_3$, 300 MHz)

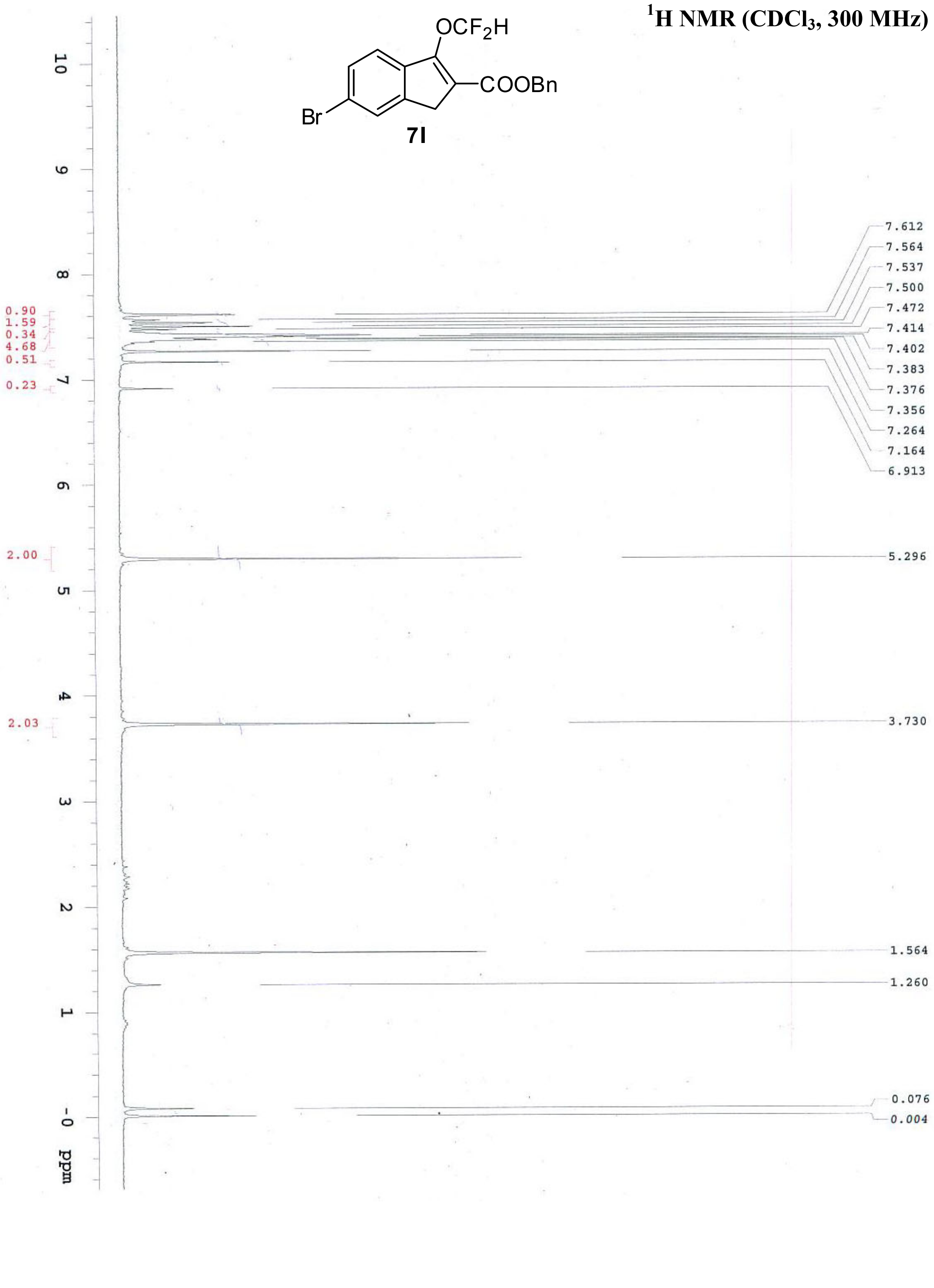

^{19}F NMR ($CDCl_3$, 282 MHz)

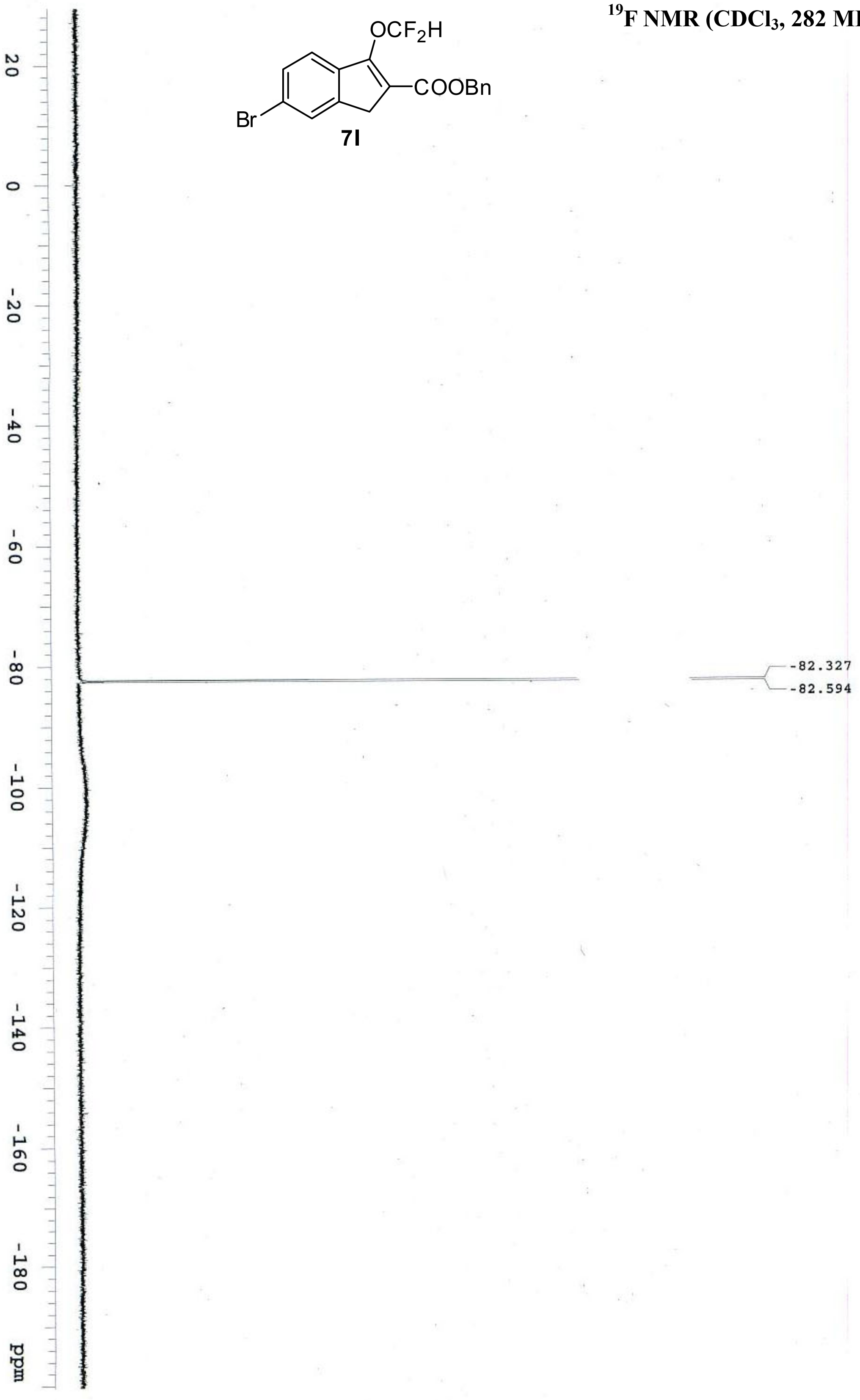

13C

^{13}C NMR ($CDCl_3$, 150.9 MHz)

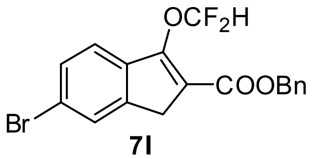

7l

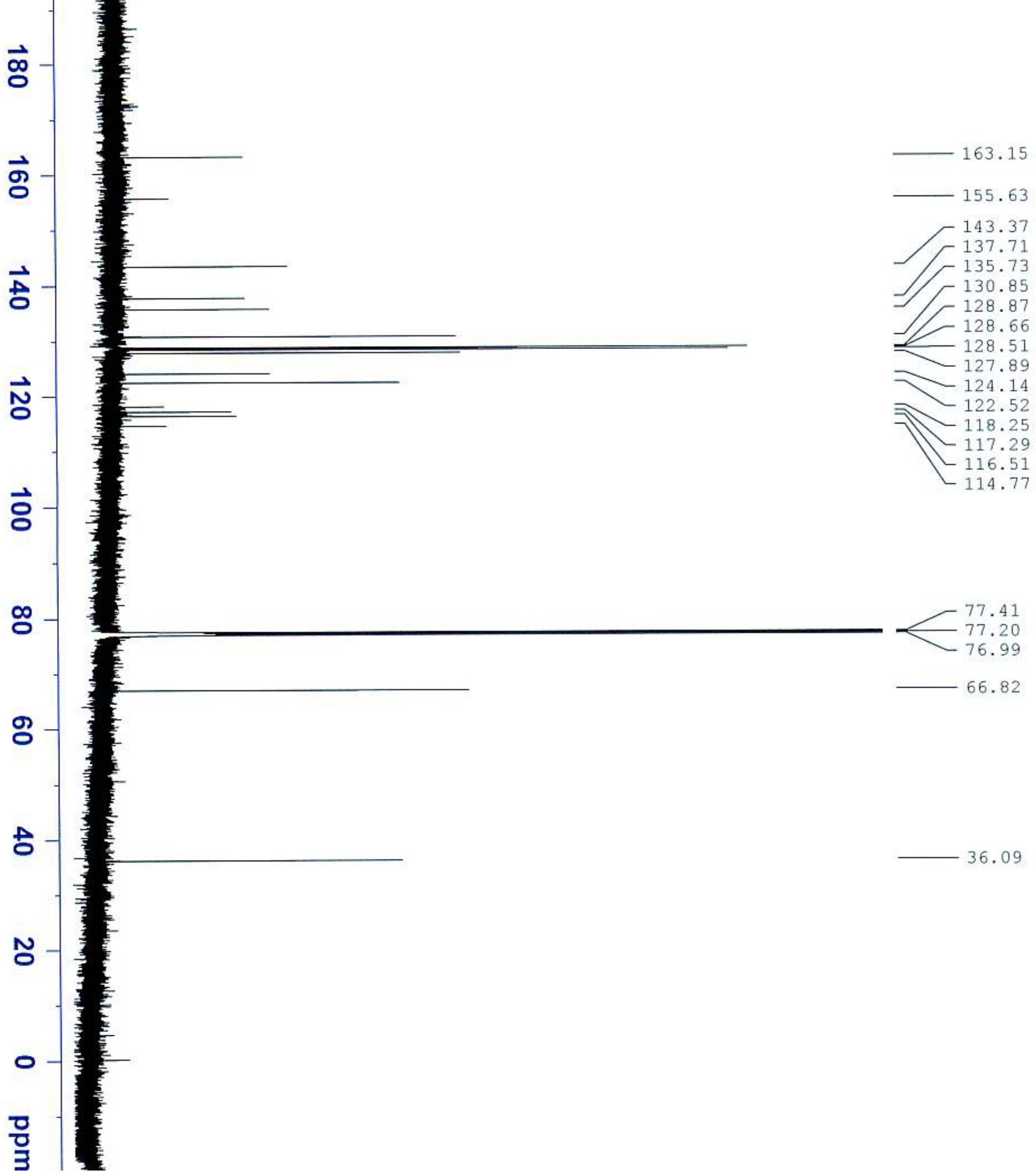

^{1}H NMR ($CDCl_3$, 300 MHz)

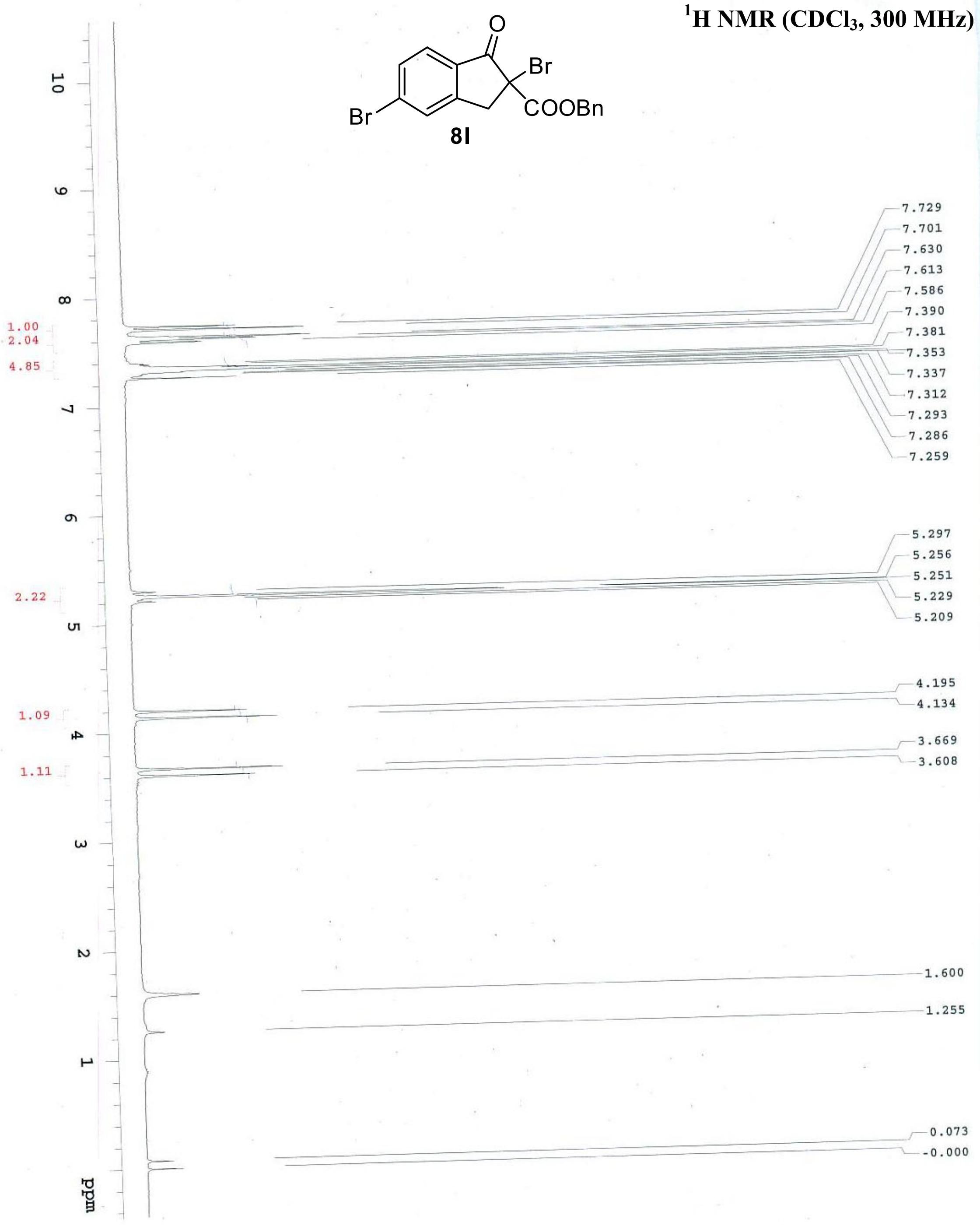

8l

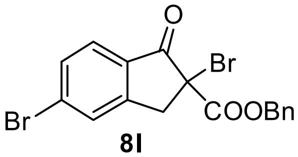

8l

^{13}C NMR (CDCl$_3$, 150.9 MHz)

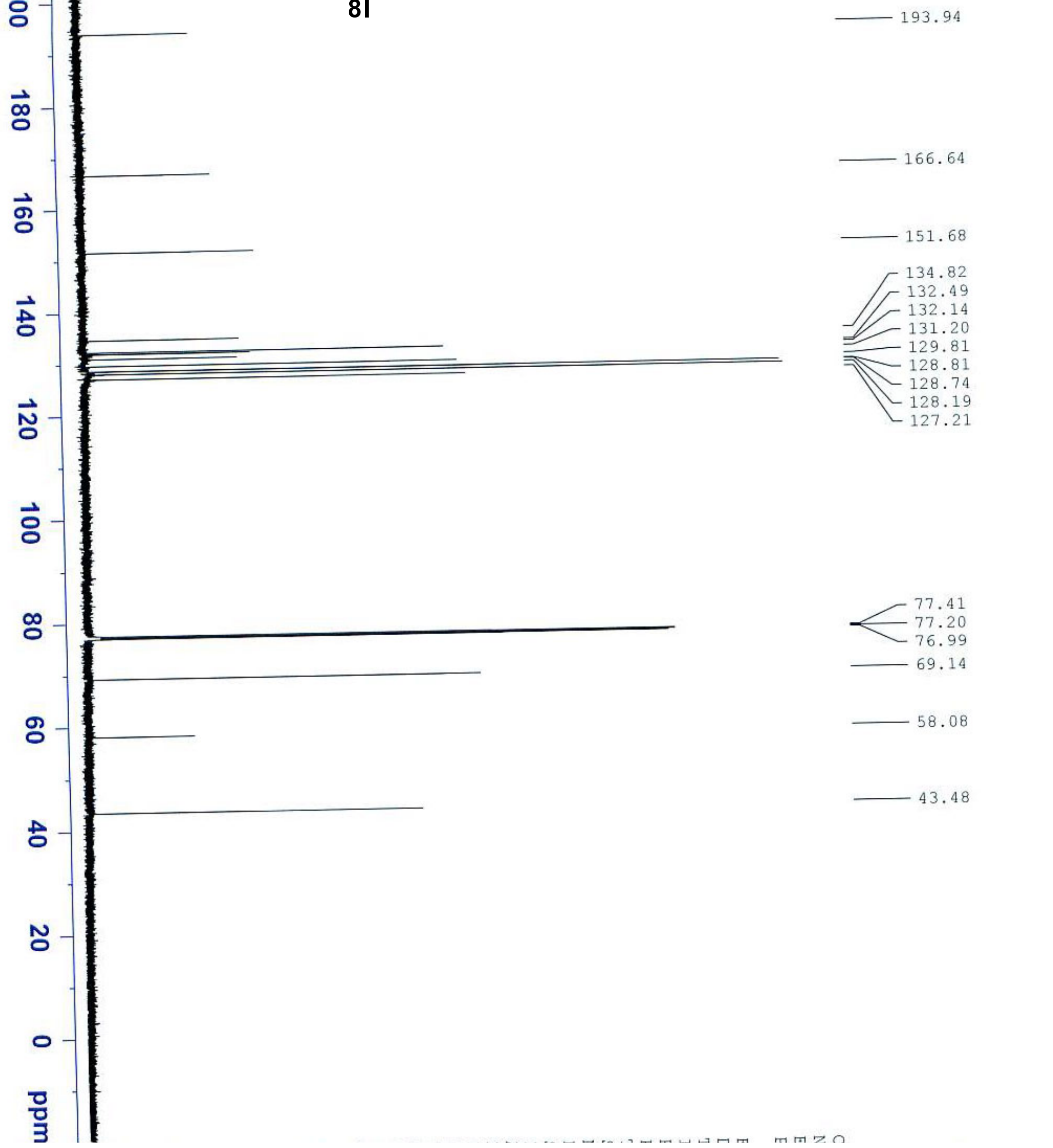

^{1}H NMR ($CDCl_3$, 300 MHz)

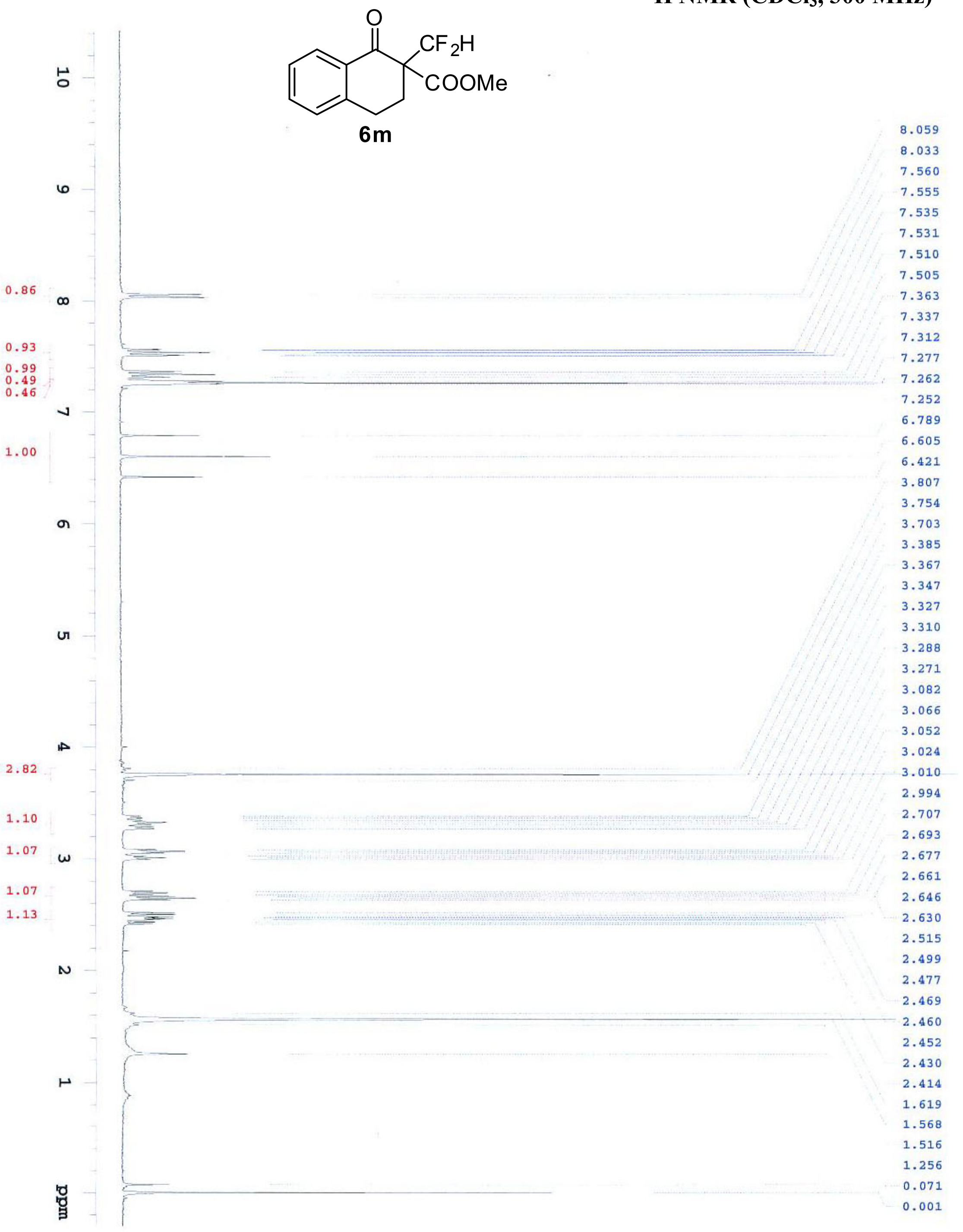

^{19}F NMR ($CDCl_3$, 282 MHz)

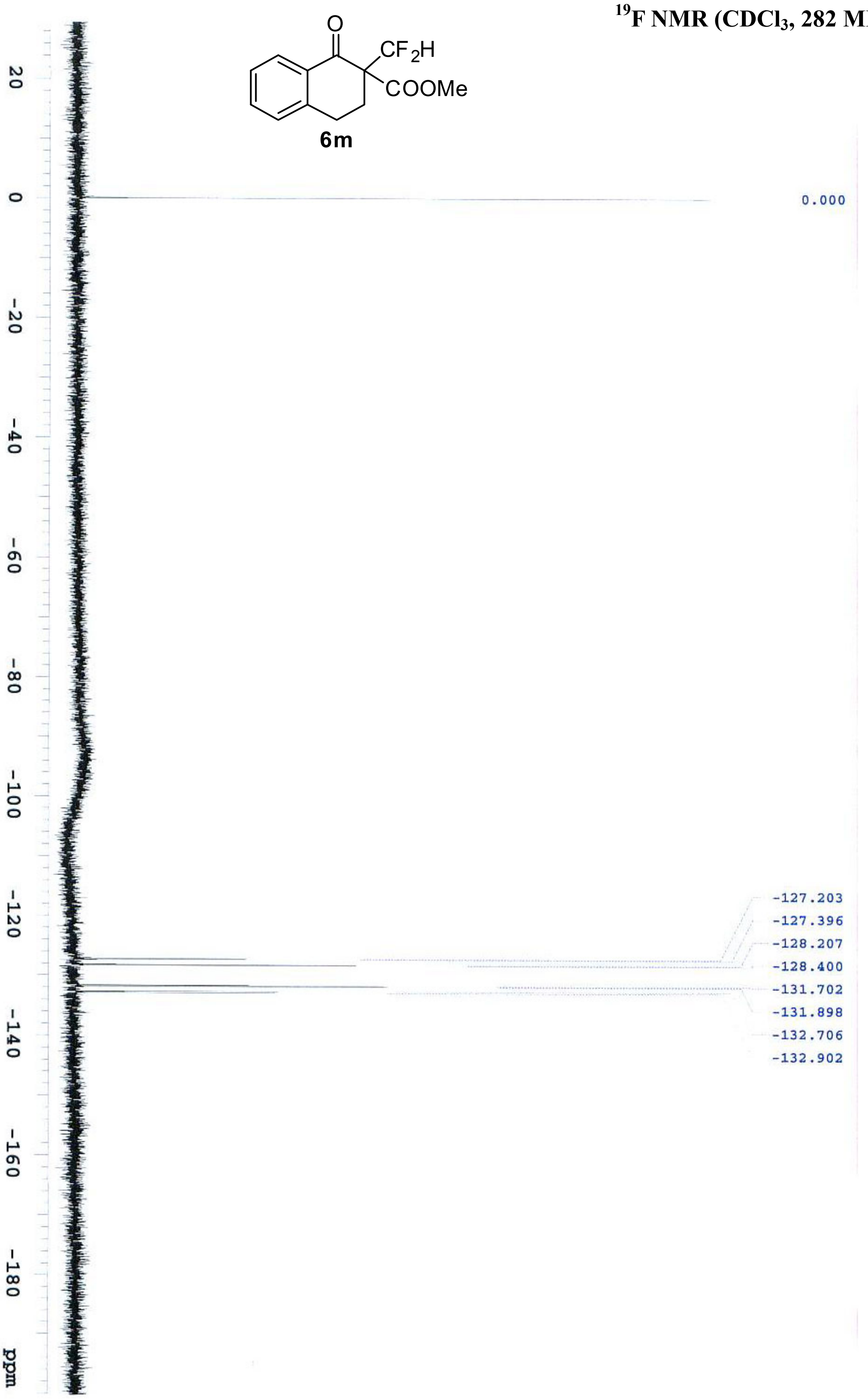

^{13}C NMR ($CDCl_3$, 150.9 MHz)

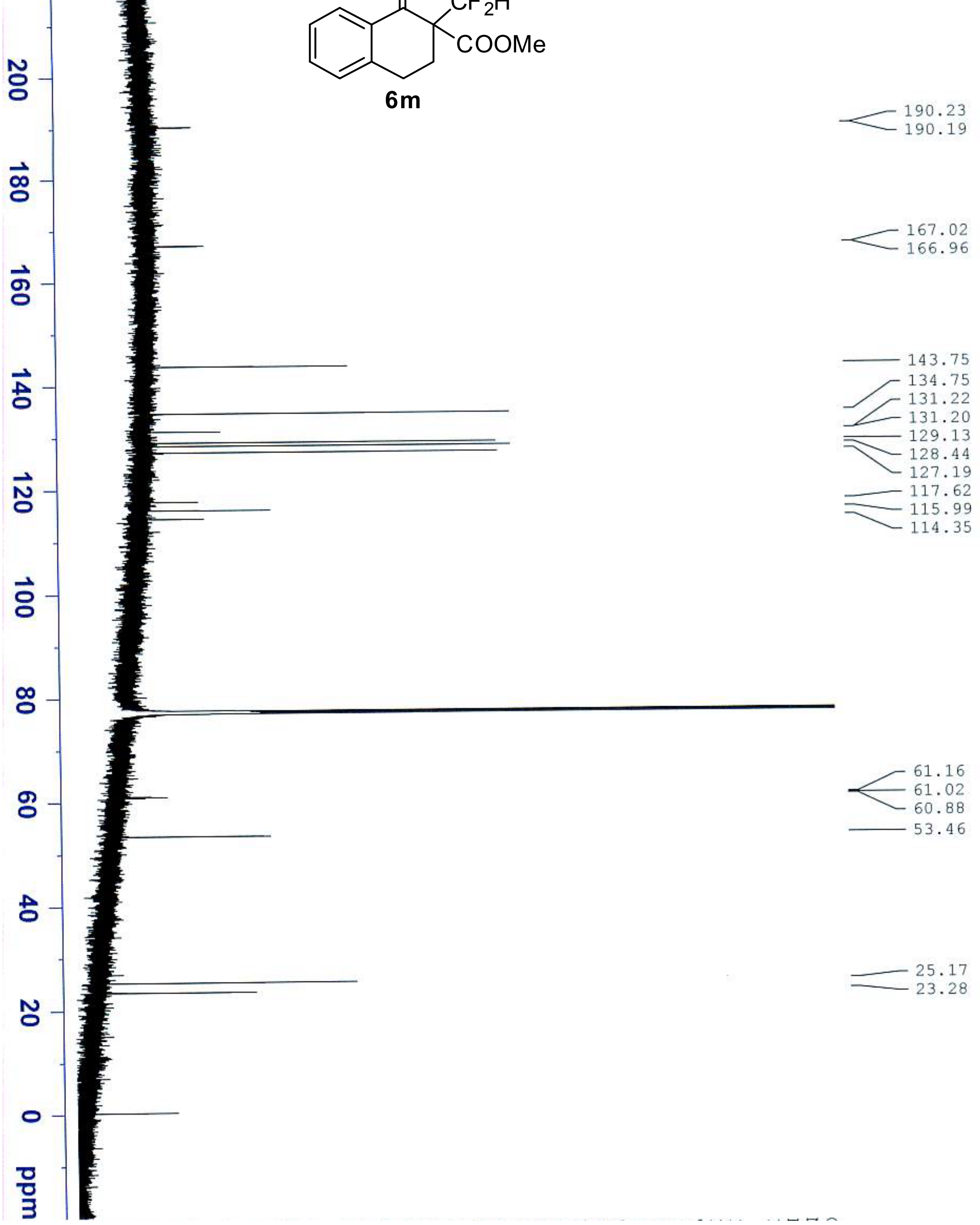

^{1}H NMR ($CDCl_3$, 300 MHz)

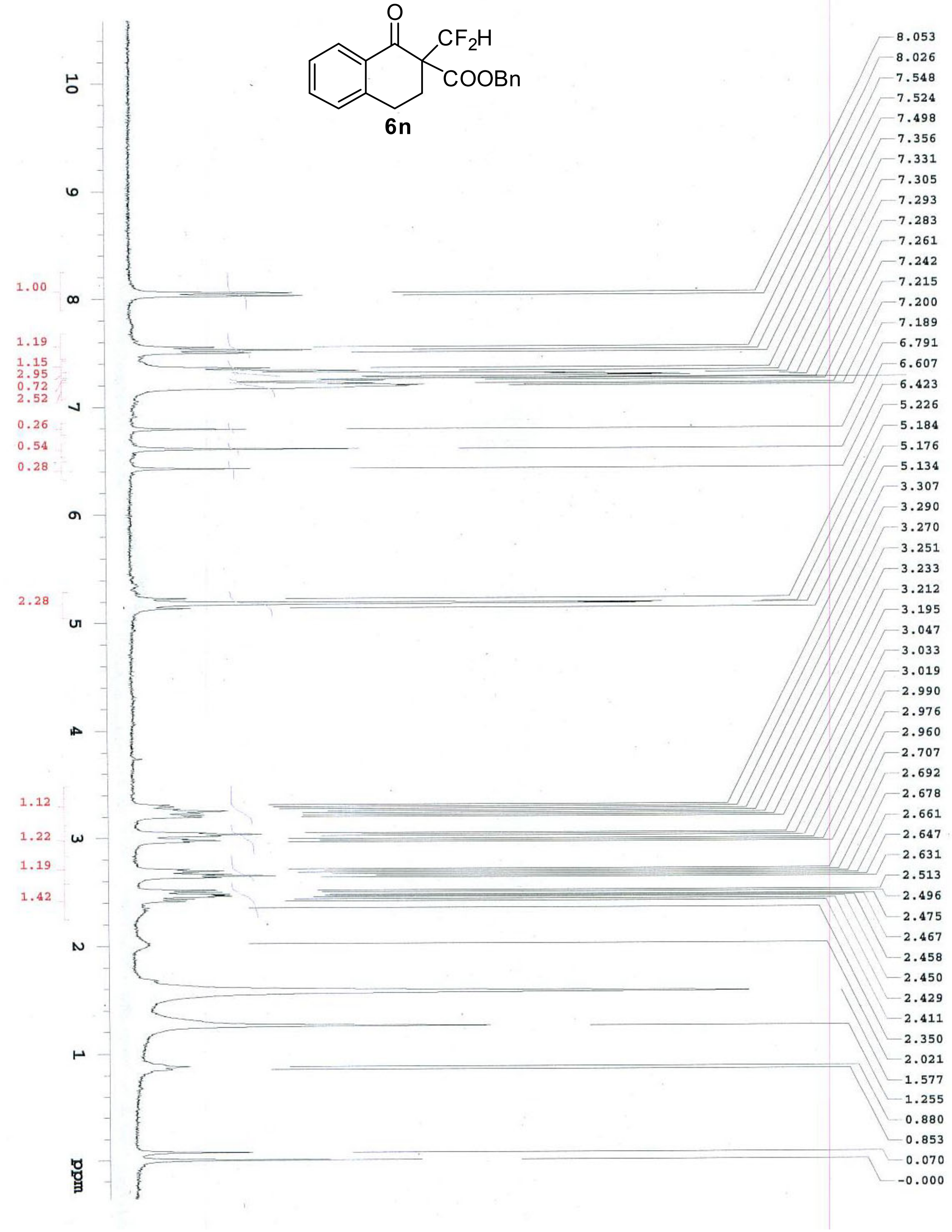

^{19}F NMR ($CDCl_3$, 282 MHz)

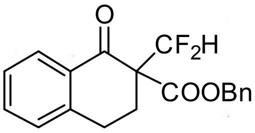

6n

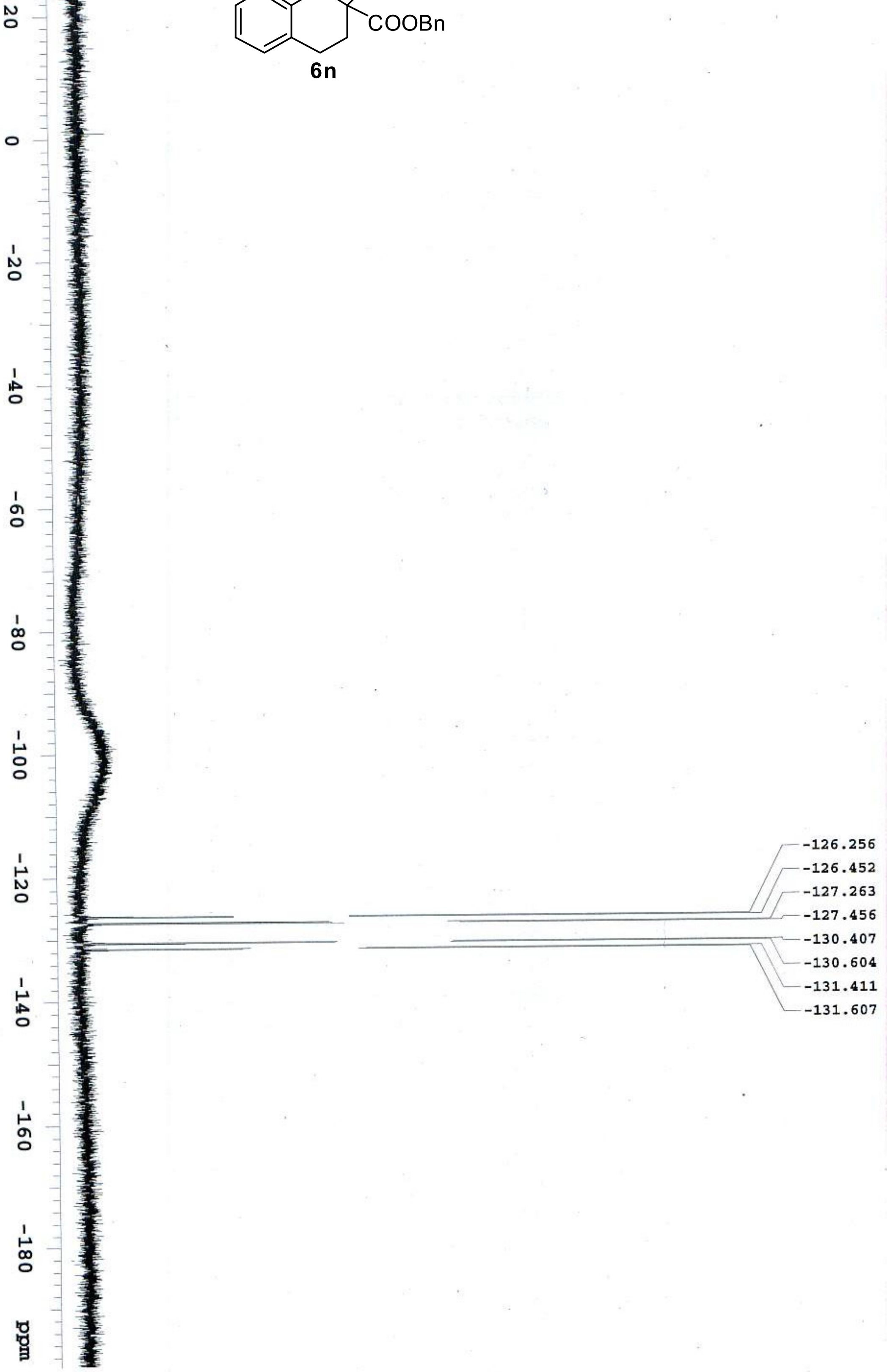

^{13}C NMR ($CDCl_3$, 150.9 MHz)

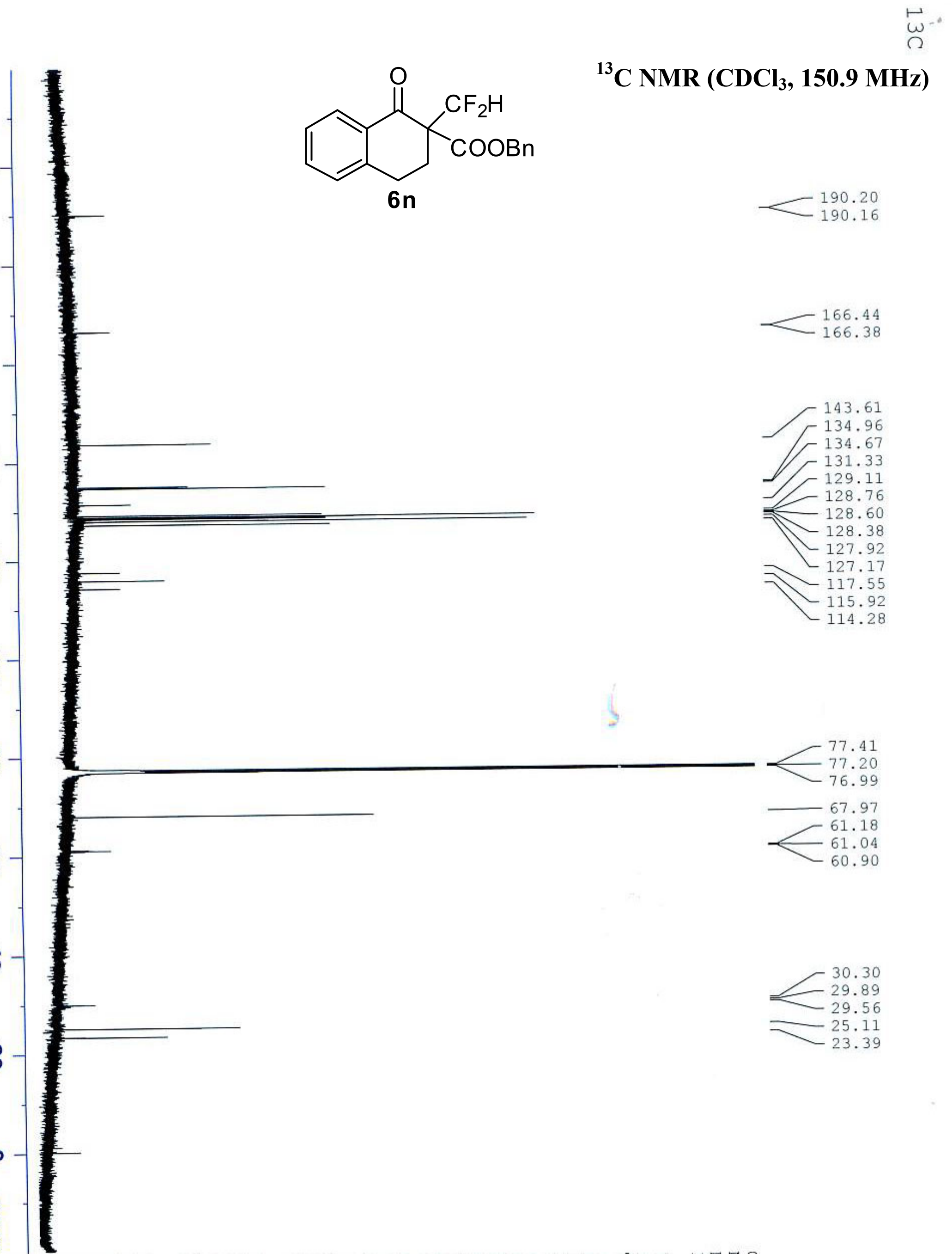

^{1}H NMR ($CDCl_3$, 300 MHz)

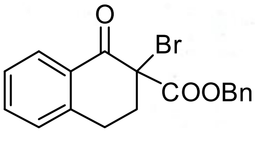

8n

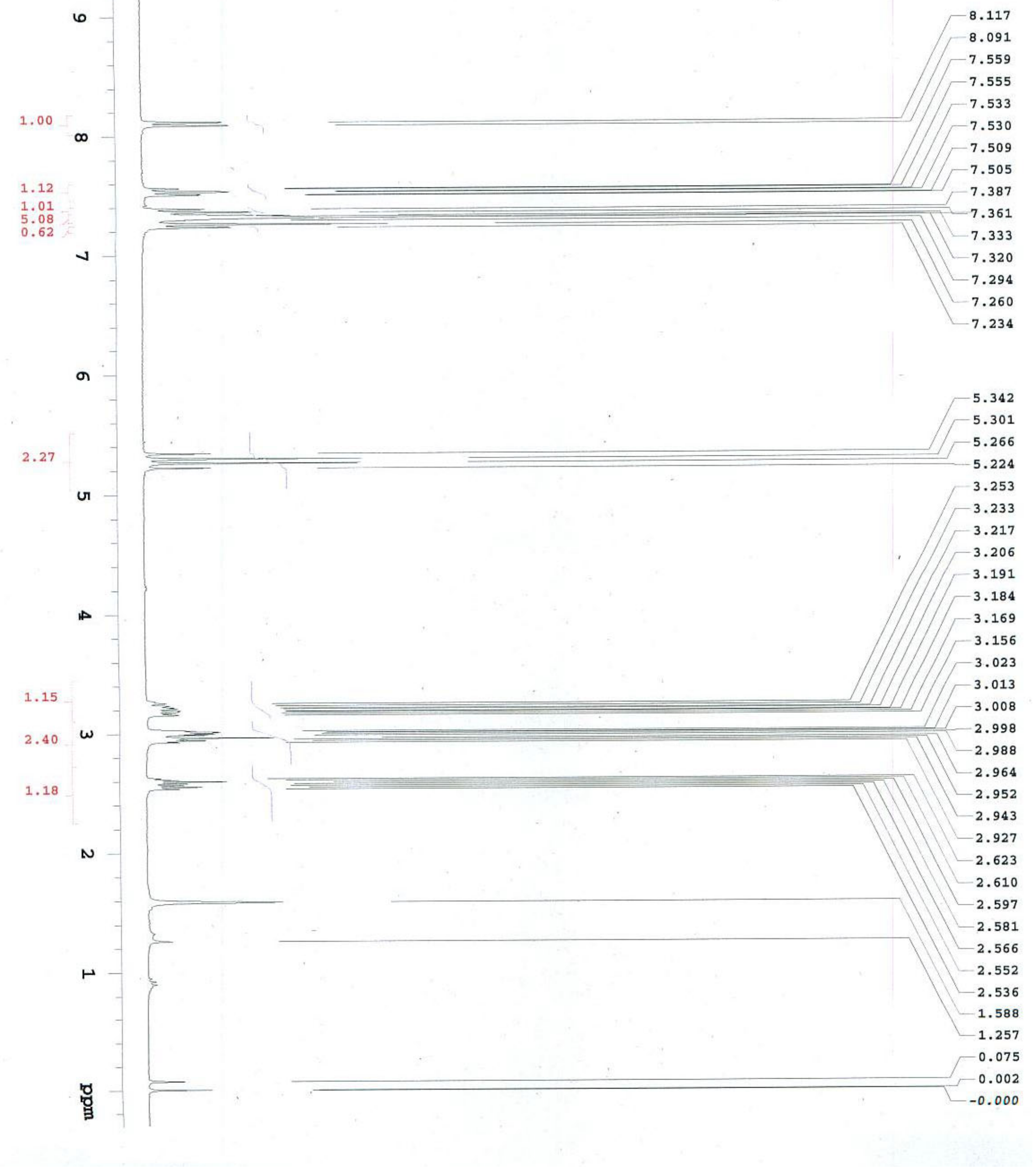

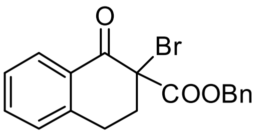

8n

^{13}C NMR ($CDCl_3$, 150.9 MHz)

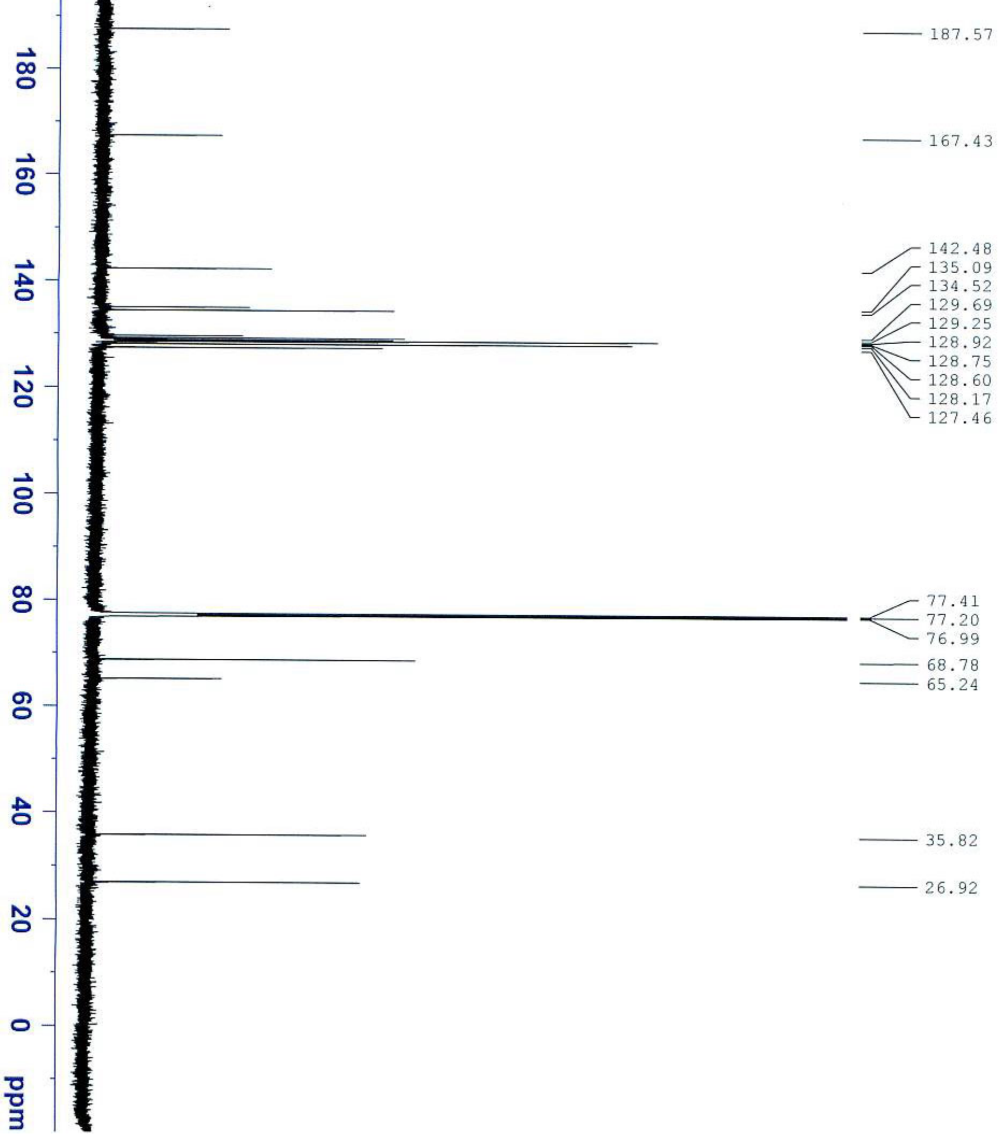

^{1}H NMR ($CDCl_3$, 300 MHz)

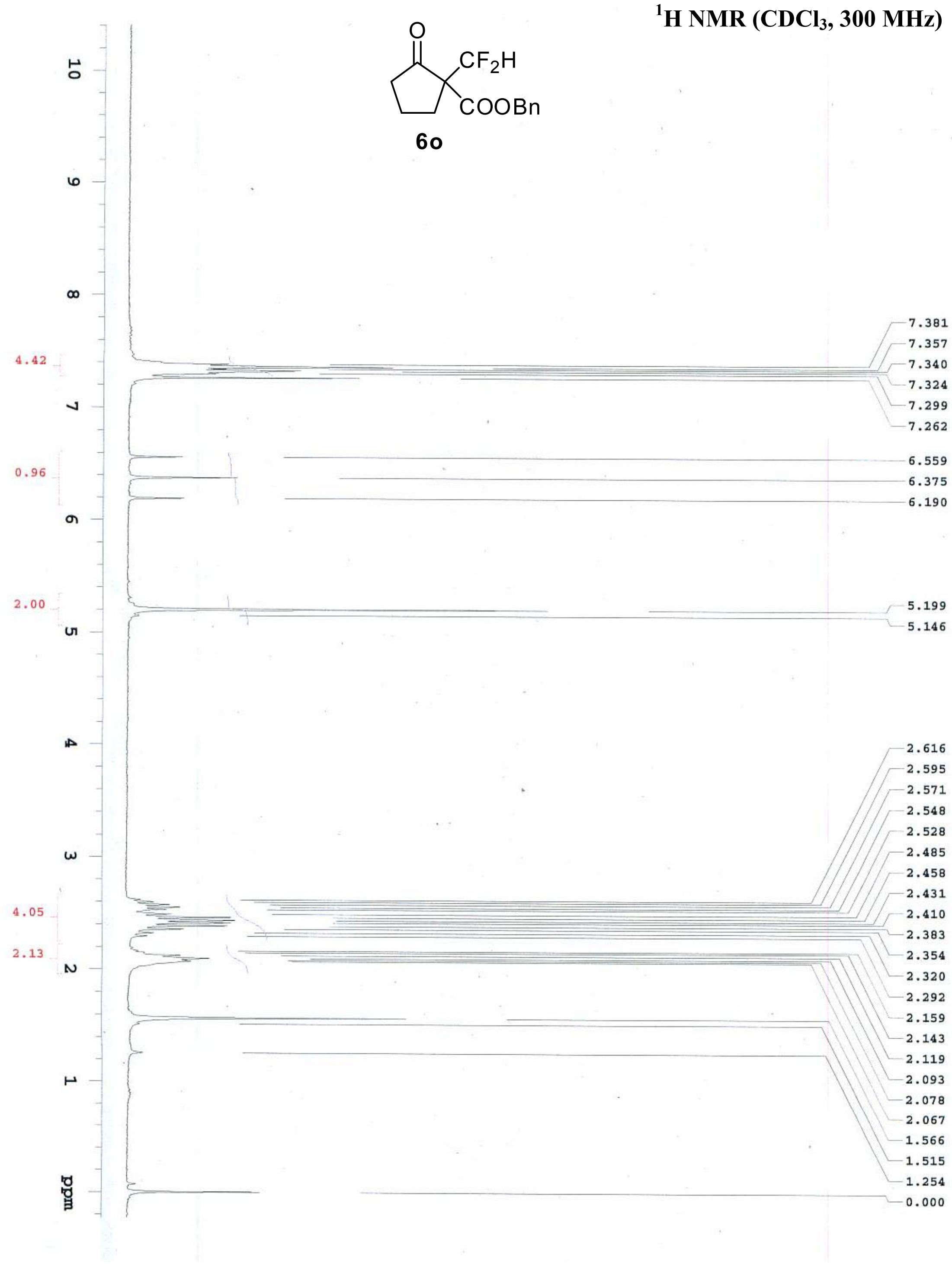

^{19}F NMR ($CDCl_3$, 282 MHz)

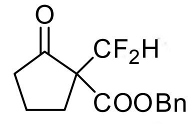

6o

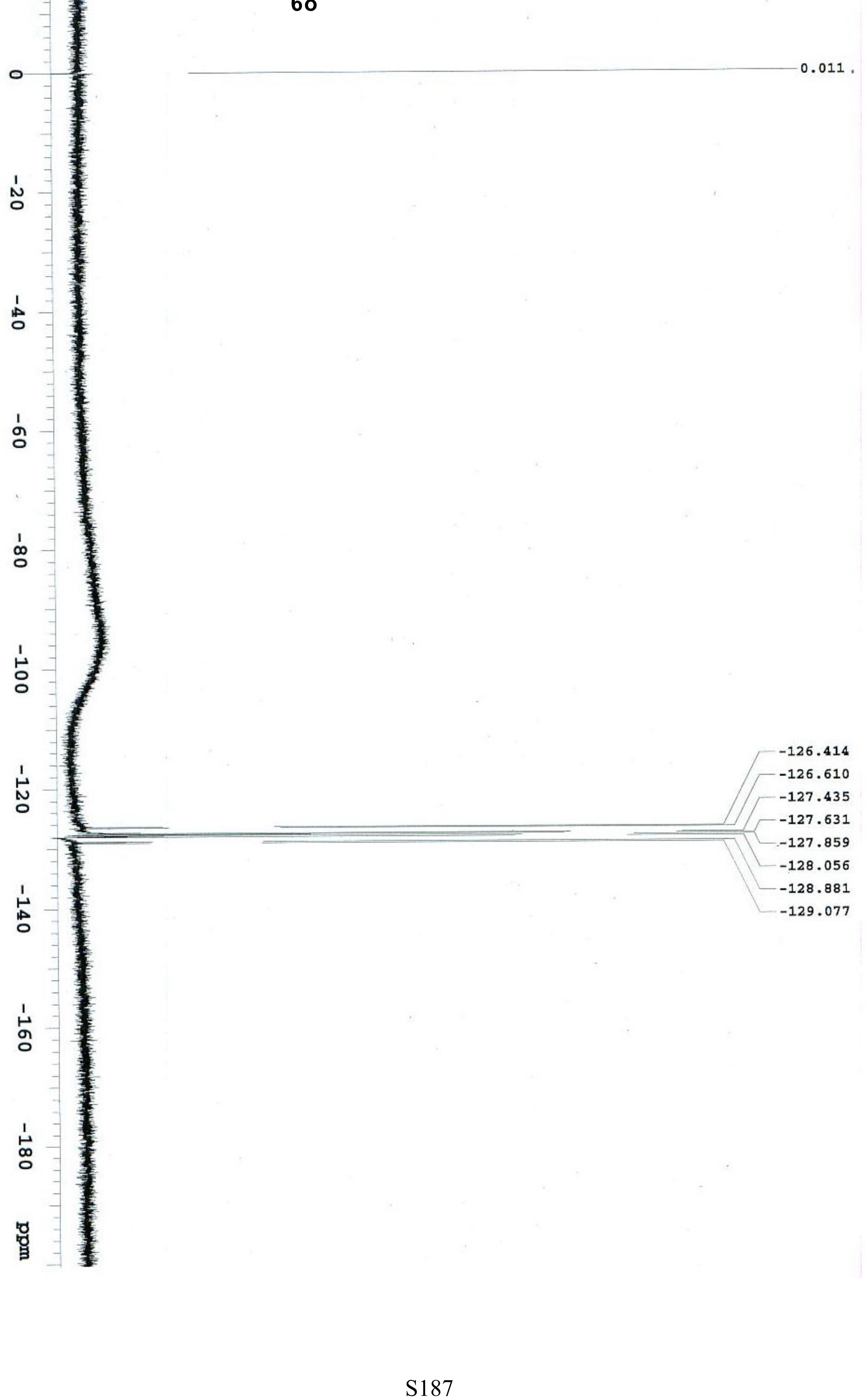

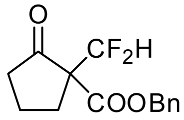

6o

^{13}C NMR ($CDCl_3$, 150.9 MHz)

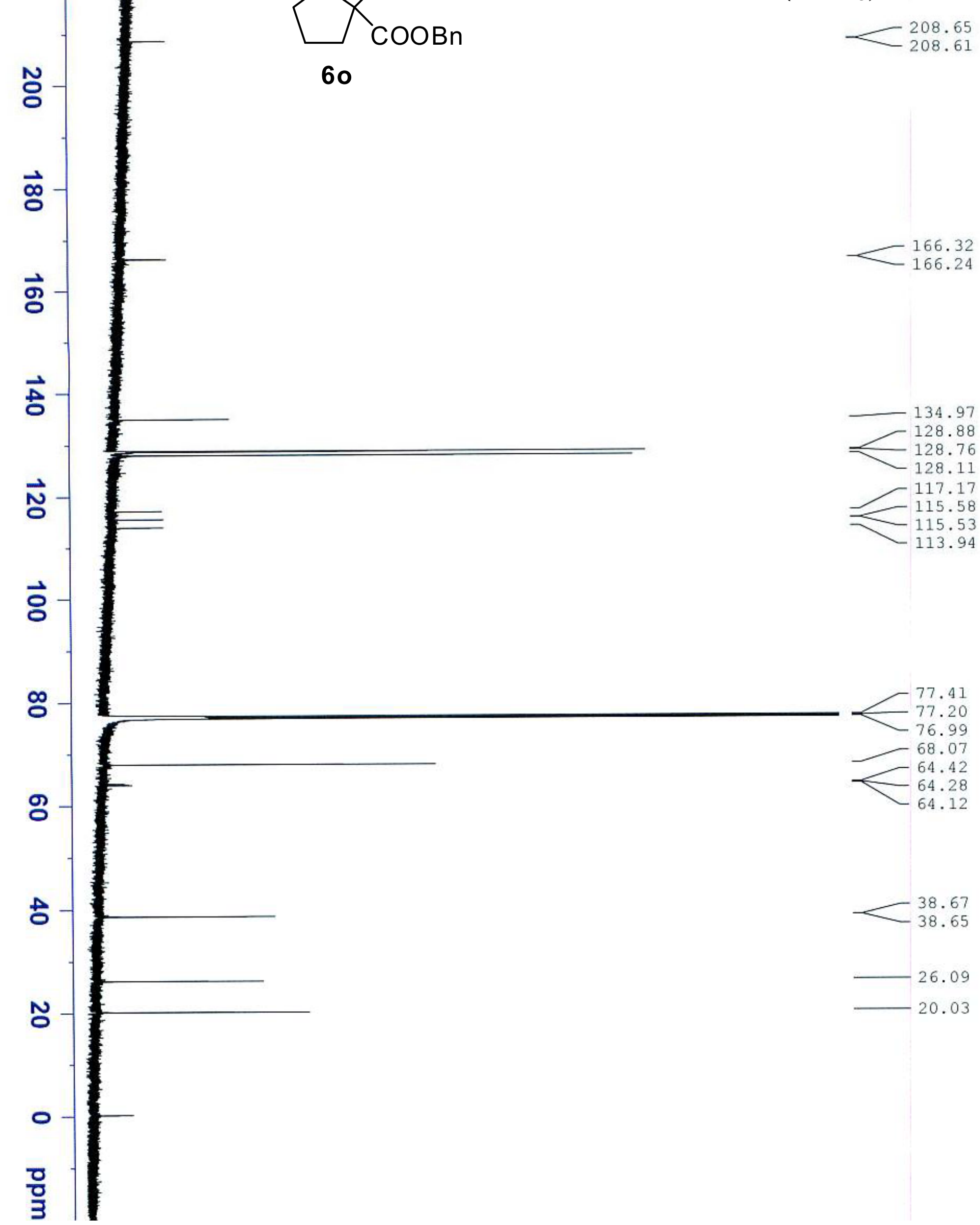

^{1}H NMR ($CDCl_3$, 300 MHz)

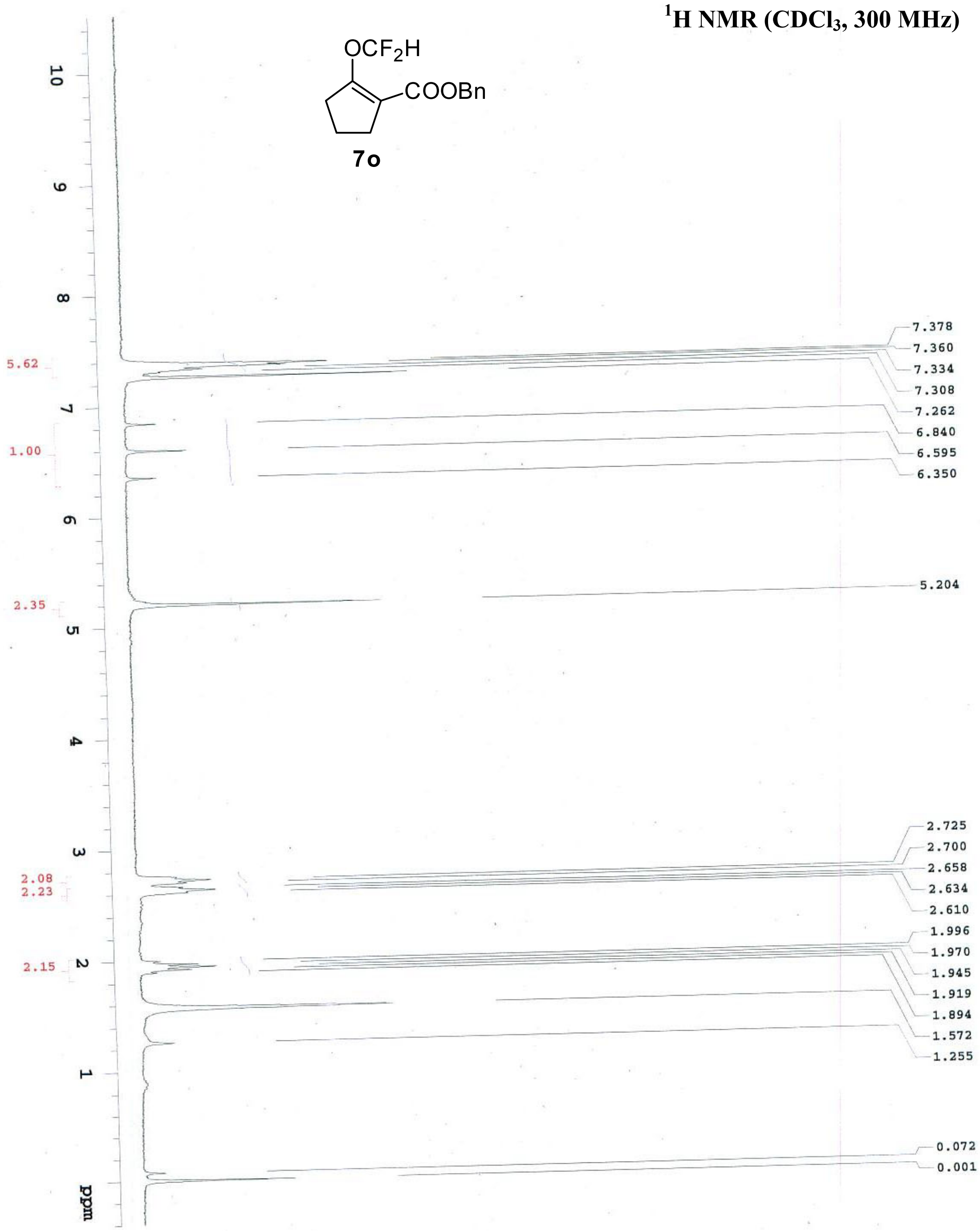

^{19}F NMR ($CDCl_3$, 282 MHz)

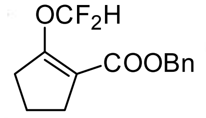

7o

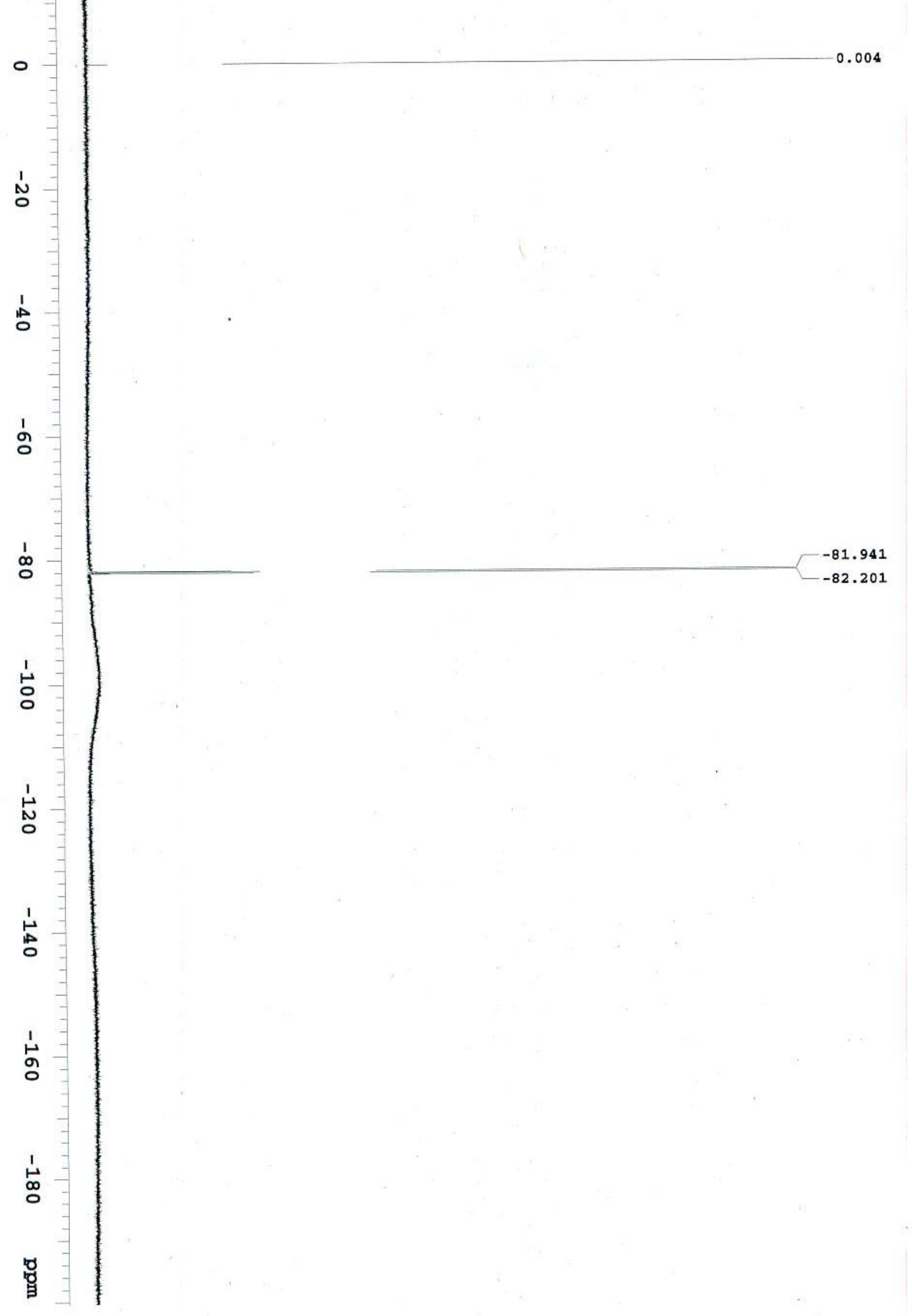

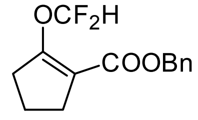

7o

^{13}C NMR ($CDCl_3$, 150.9 MHz)

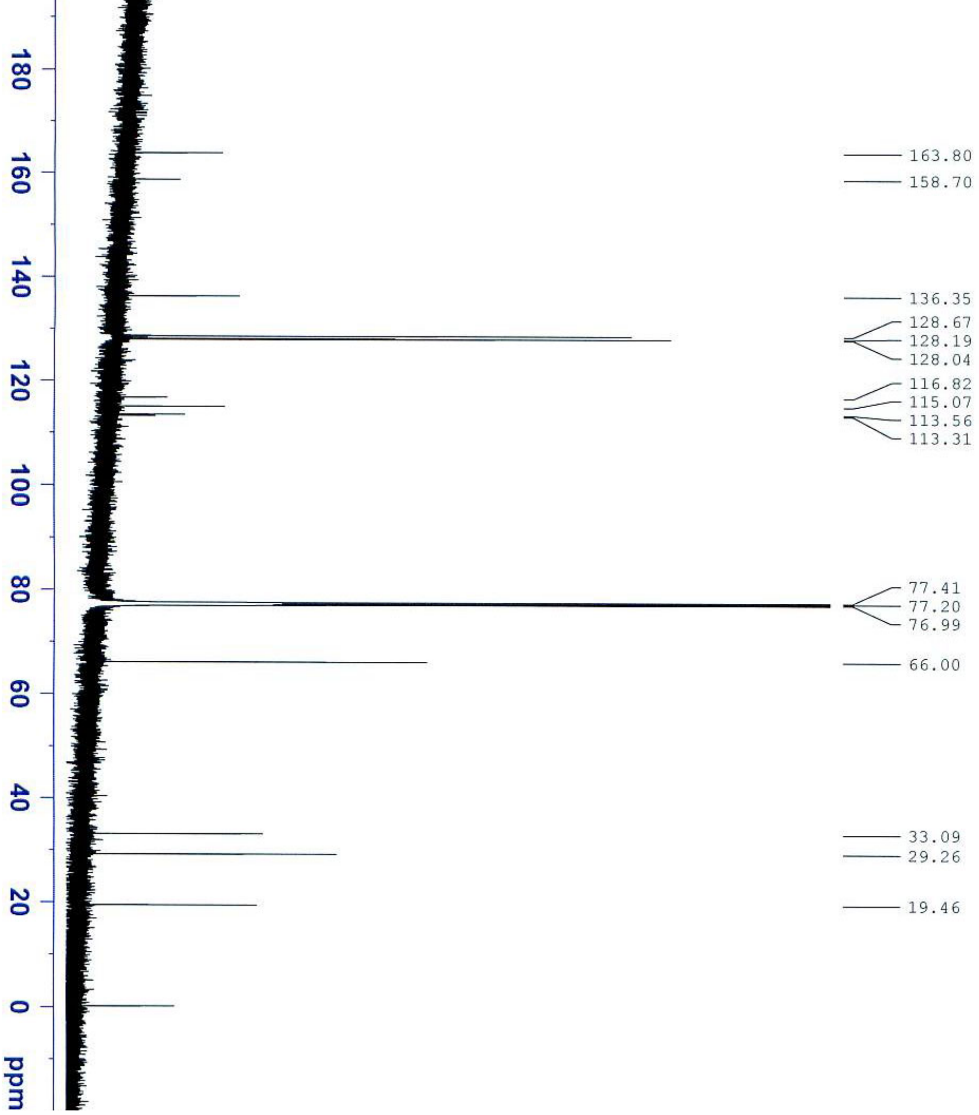

^{1}H NMR (CDCl$_3$, 300 MHz)

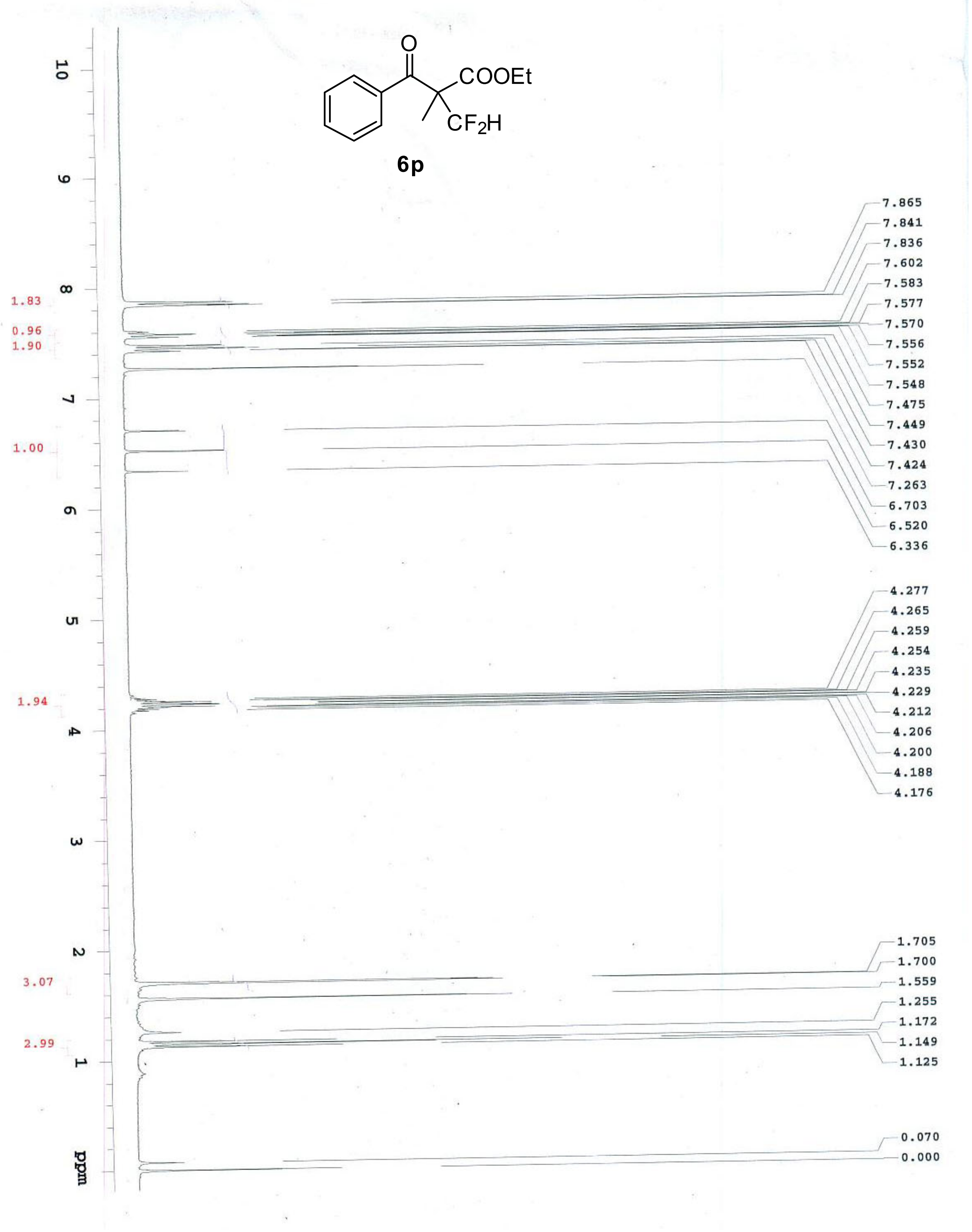

6p

^{19}F NMR (CDCl$_3$, 282 MHz)

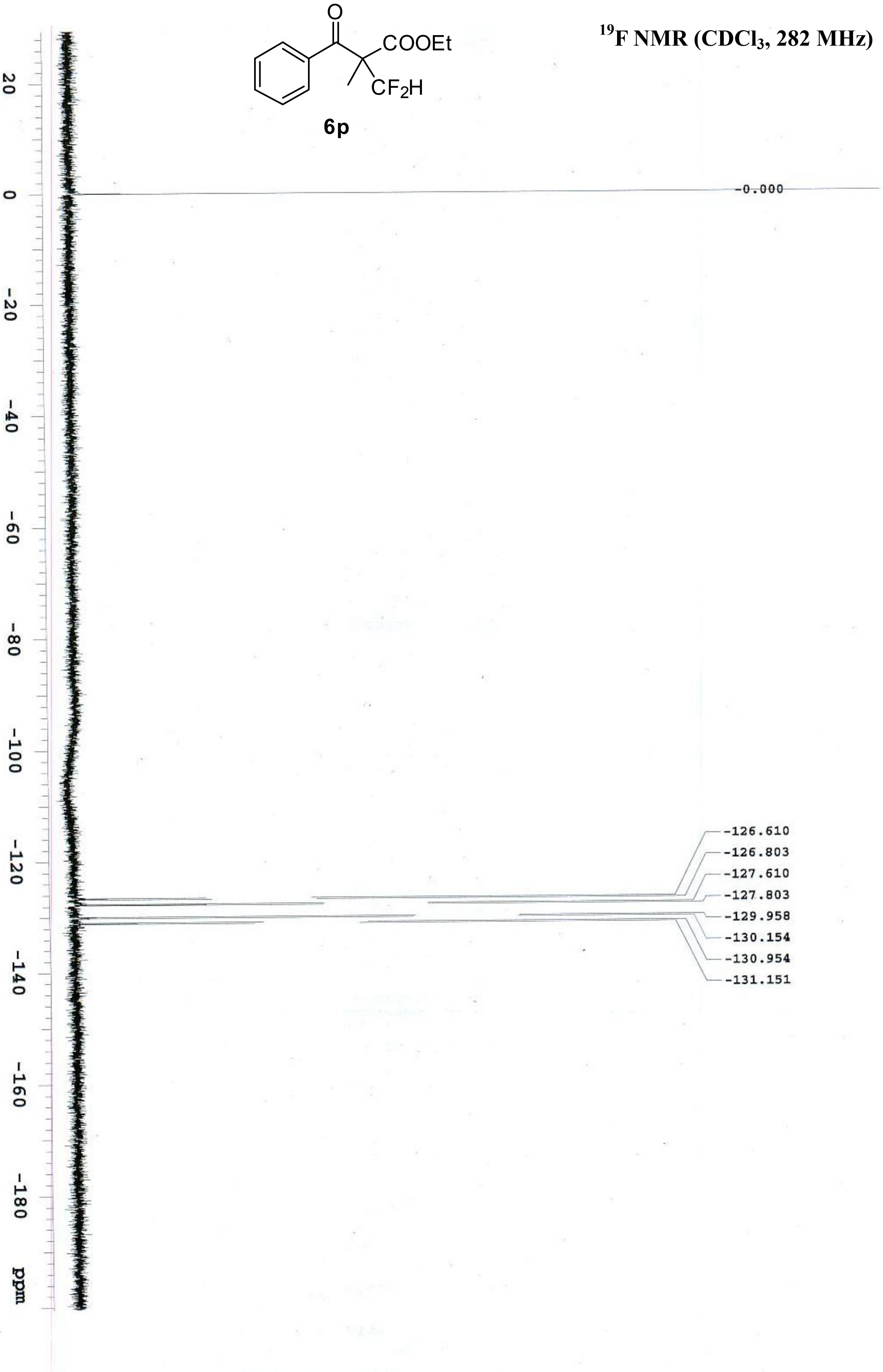

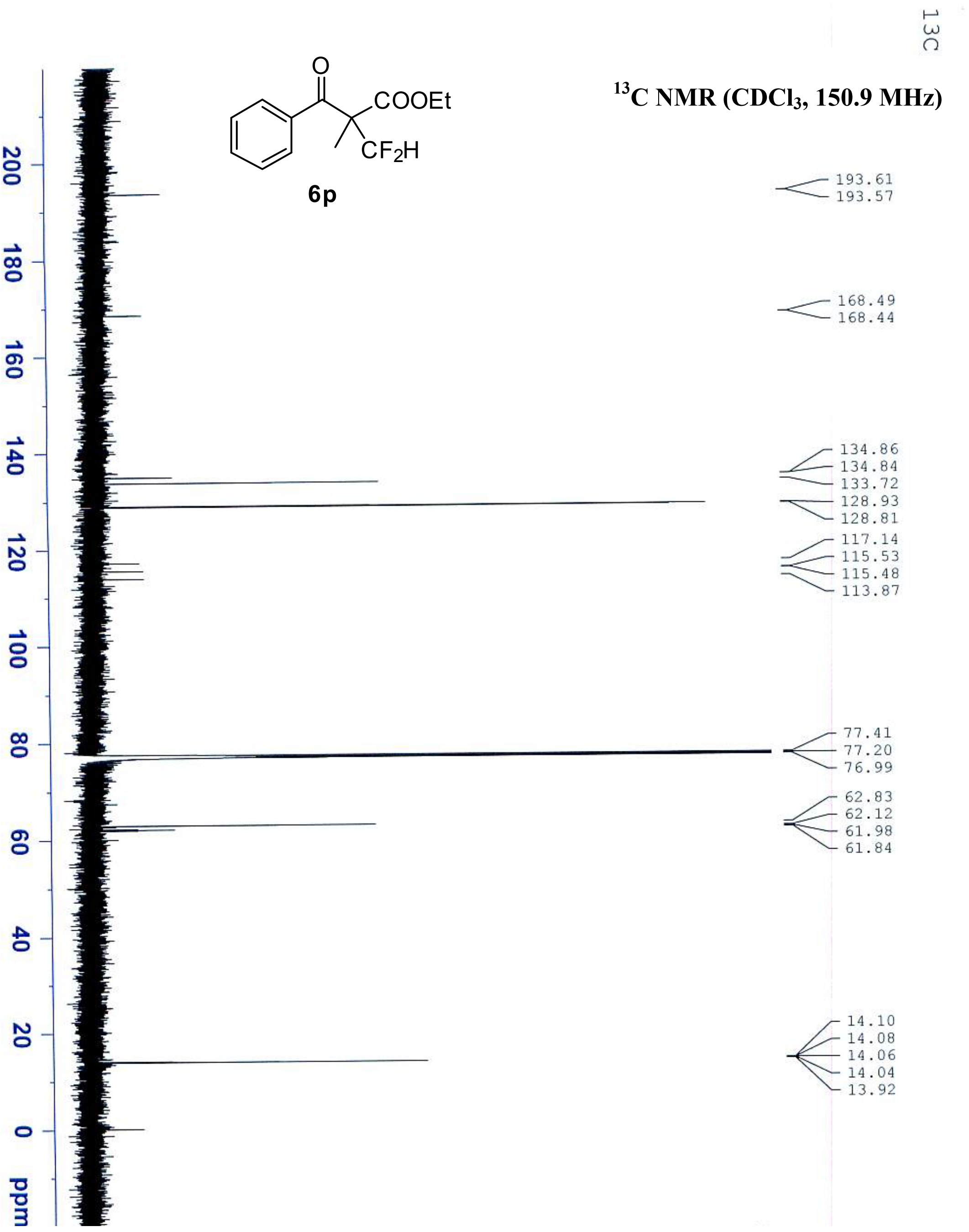
13C
^{13}C NMR ($CDCl_3$, 150.9 MHz)
COOEt
CF_2H
6p
193.61
193.57
168.49
168.44
134.86
134.84
133.72
128.93
128.81
117.14
115.53
115.48
113.87
77.41
77.20
76.99
62.83
62.12
61.98
61.84
14.10
14.08
14.06
14.04
13.92
200
180
160
140
120
100
80
60
40
20
0
ppm

^1H NMR ($CDCl_3$, 300 MHz)

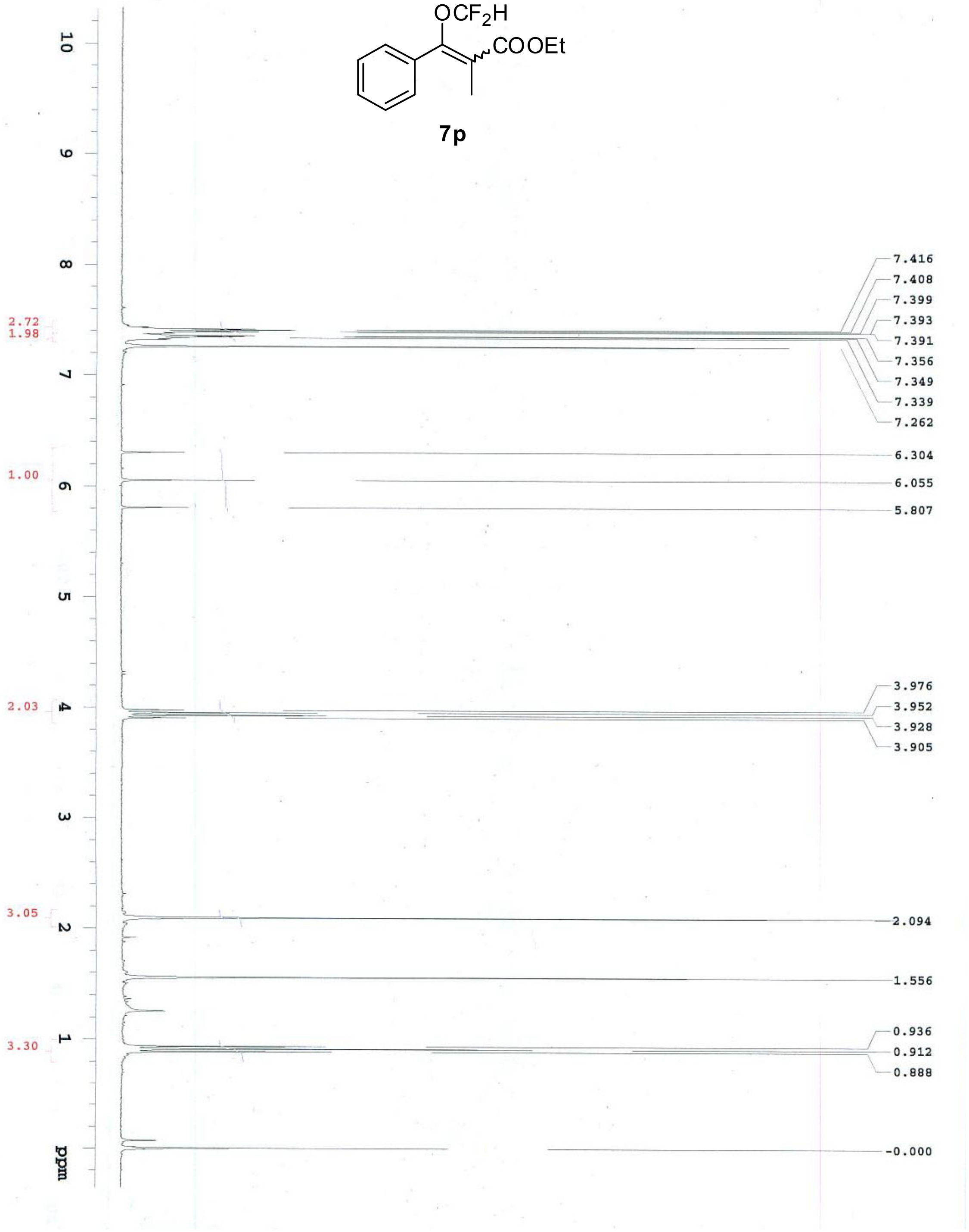

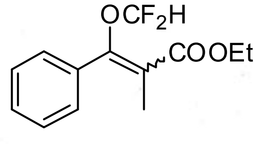

7p

^{19}F NMR ($CDCl_3$, 282 MHz)

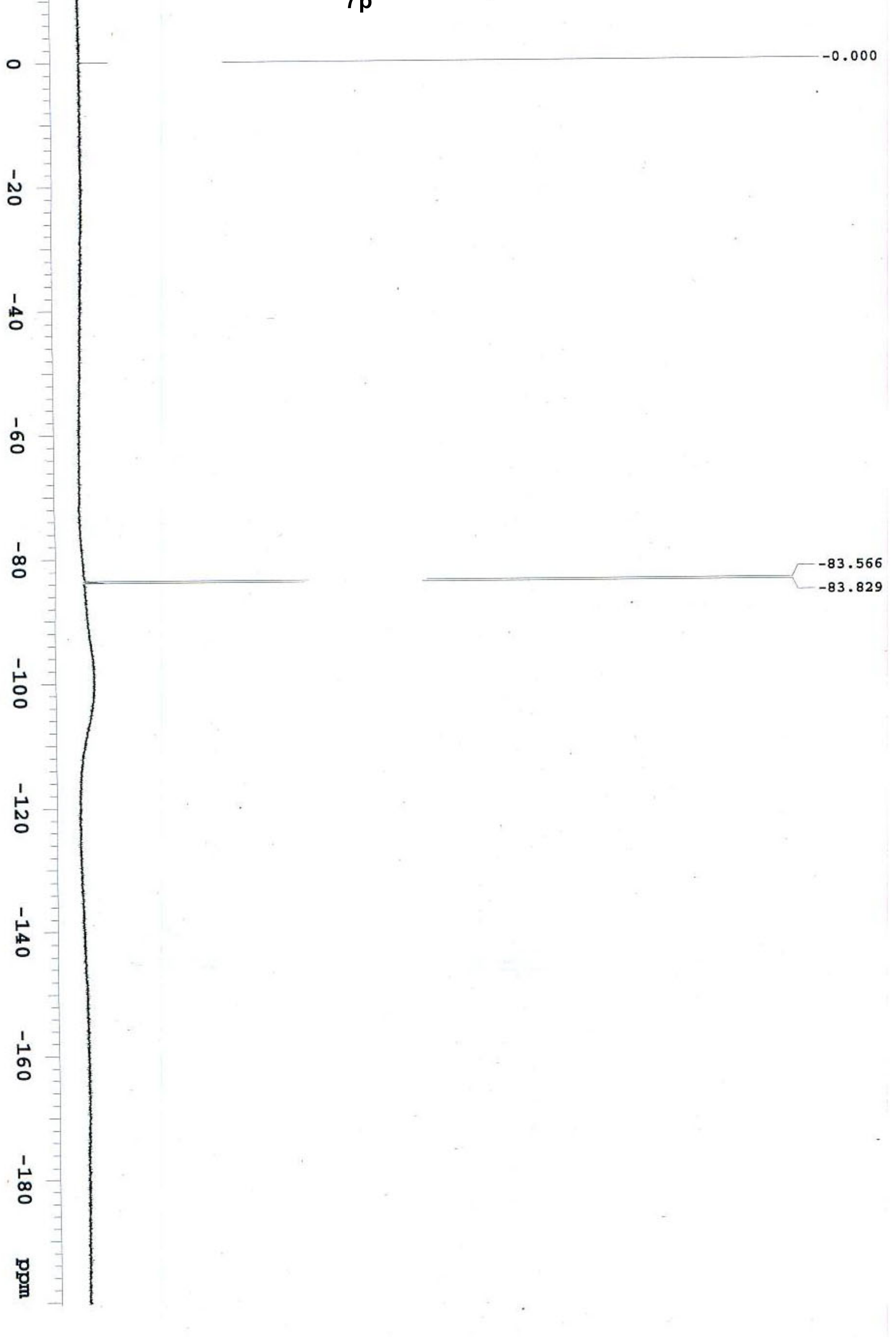

^{13}C NMR (CDCl$_3$, 150.9 MHz)

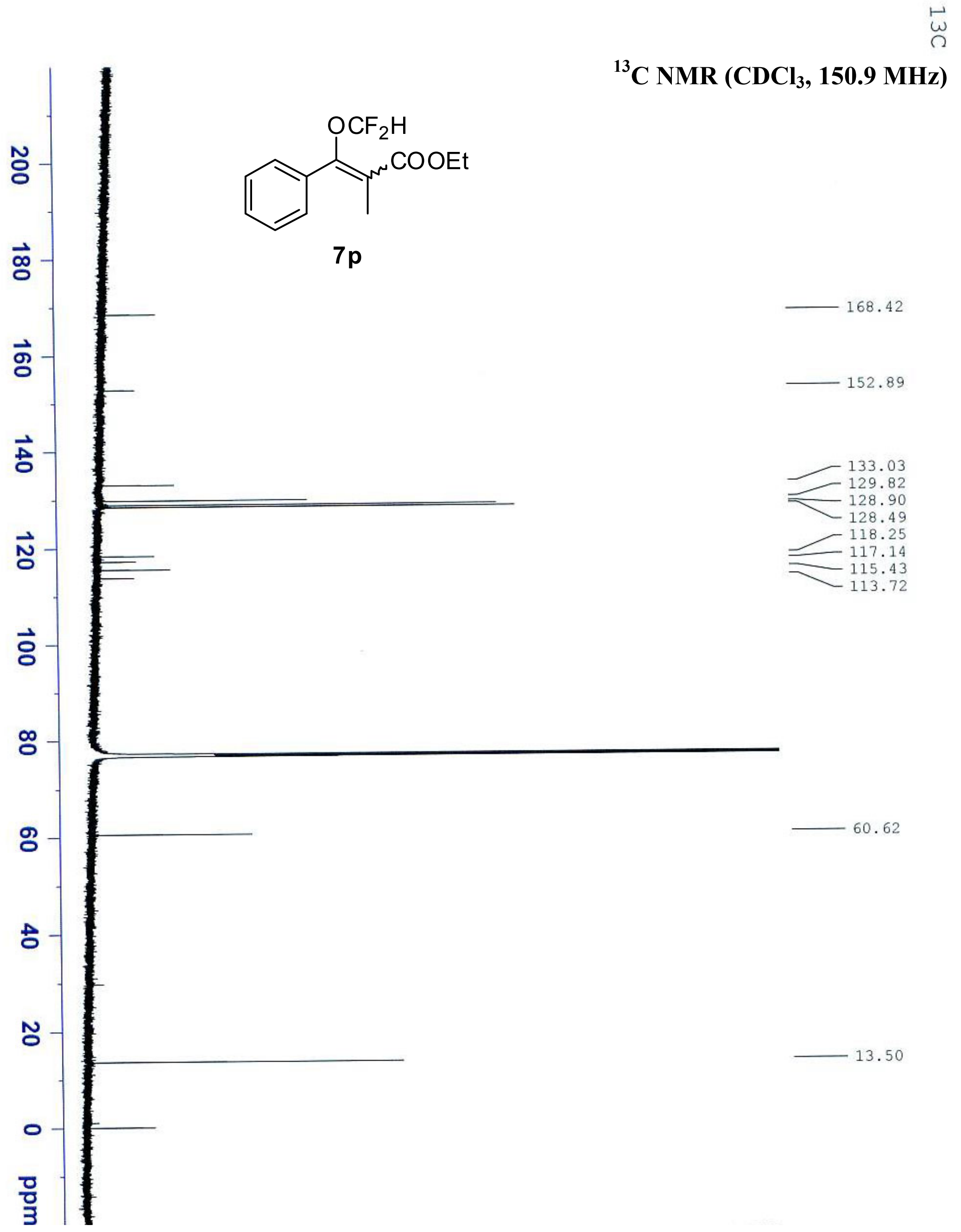

Supplement: Supplementary file 1 [file open0001-0227-SD1.pdf]
